# Puzzling Out the Structure of Novofumigatamide: Total Synthesis of Constitutional Isomers. Part II

**DOI:** 10.1021/acs.joc.2c01228

**Published:** 2022-09-21

**Authors:** Patricia García-Domínguez, Angel R. de Lera

**Affiliations:** CINBIO, Universidade de Vigo, 36310Vigo, Spain

## Abstract

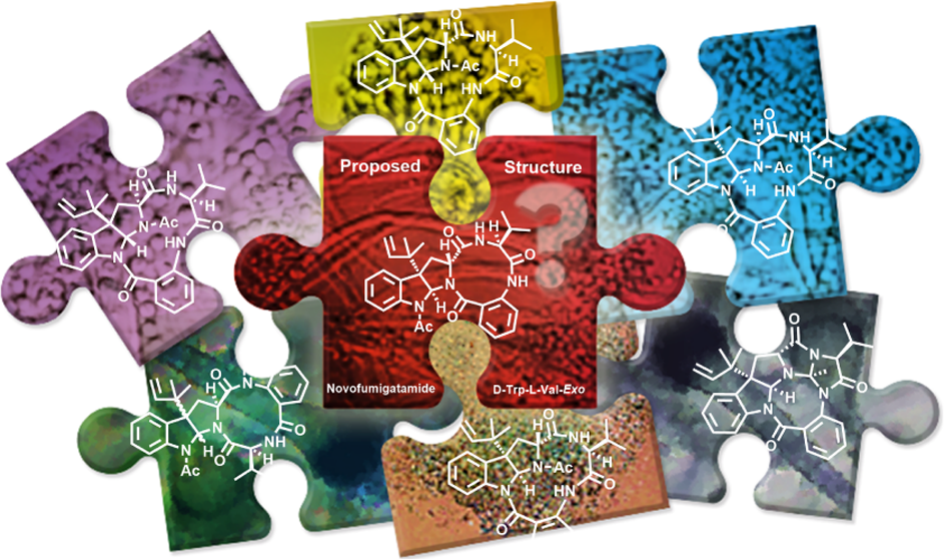

The total synthesis of several constitutional isomers showing a different connectivity of the macrolactam ring with the hexahydropyrrolo[2,3-*b*]indole core, as well as those arising from the positional exchange of the valine and the anthranilate units of the structure originally proposed for (−)-novofumigatamide, has been carried out. The constitutional isomers with 12-membered ring macrolactam connected with the pyrroloindoline framework through the indole nitrogen, and the acetyl group at the pyrrole nitrogen, of *endo* relative configuration, were prepared through the condensation between the tryptophan and valine edges derived from l- or d-tryptophan and l-valine amino acids. The corresponding *exo* products are highly unstable structures difficult to isolate and characterize. A second group of isomeric structures synthesized considered the positional exchange between the valine and the anthranilate residues within the macrolactam ring in the originally proposed macrocyclic structure. Comparison of the spectroscopic data allowed us to discard these alternative structures for the natural product.

## Introduction

**Figure 1:**
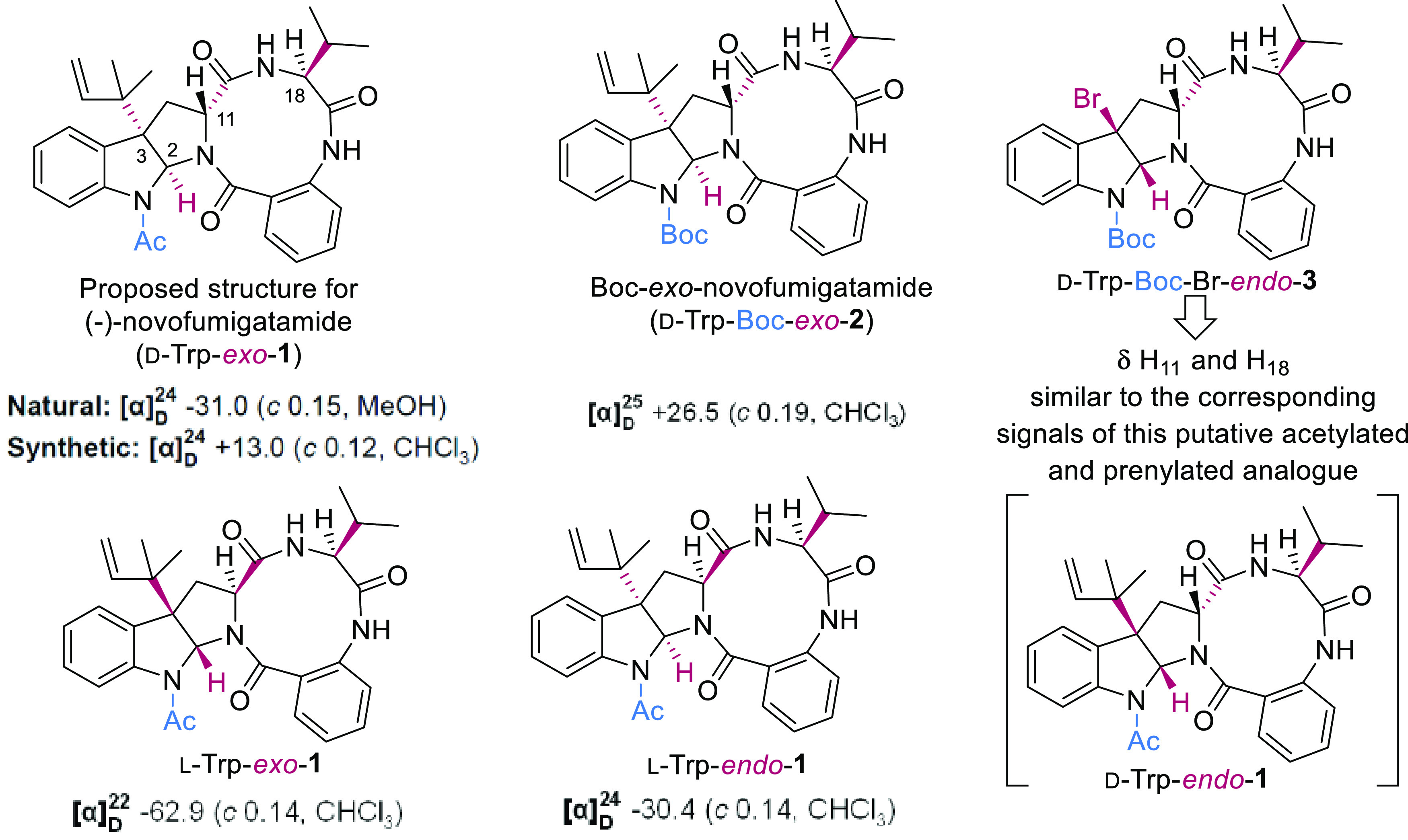
Proposed structure of novofumigatamide (**1**), diastereomers, and *N*-Boc-analogues (**2** and **3**) prepared in a previous manuscript.

We have recently reported the total synthesis of the proposed structure of novofumigatamide (d-Trp-*exo*-**1**),^1^ a Boc-analogue (d-Trp-*exo*-**2**), an *N*-Boc-brominated precursor with *endo* relative configuration (d-Trp-Br-*endo*-**3**), all of them arising from d-tryptophan and l-valine amino acids, as well as the two diastereomers (l-Trp-*exo*-**1** and l-Trp-*endo*-**1**) of the purported natural product built from l-tryptophan and the same enantiomer of valine (Figure 1).^2^ None of the spectroscopic data of these products matched those provided in the original publication describing the isolation of this naturally occurring alkaloid.^1^ In the previous manuscript (DOI: 10.1021/acs.joc.2c01127), we reported that the values of the ^1^H NMR chemicals shifts for H11 and H18 key signals remained unaltered after replacing a bromine atom at C3 by a reverse prenyl group, or an *N*-acetyl group on the indole nitrogen by an *N*-Boc group. Furthermore, the very characteristic chemical shifts exhibited by these protons in *endo* and *exo* diastereomeric compounds make these values very useful for the straightforward assignment of the relative configuration of bromine-containing synthetic precursors and final products. Herein, we describe the total synthesis of several constitutional isomers of the original structure proposed for novofumigatamide that display either a different connection between the macrolactam ring and the hexahydropyrrolo[2,3-*b*]indole core^3,4^ or are derived from the positional exchange of the valine and the anthranilate moieties.

## Results and Discussion

**Figure 2:**
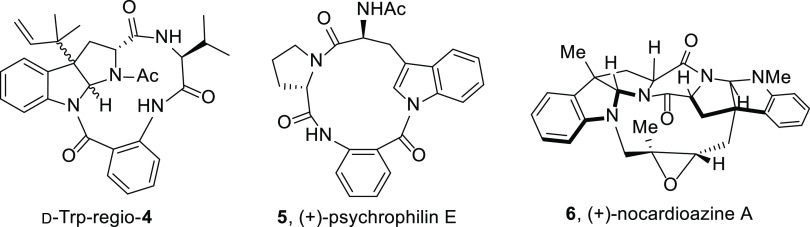
New proposed structure for novofumigatamide (d-Trp-regio-**4**), inspired by those of (+)-psychrophilin E (**5**) and (+)-nocardioazine A (**6**).

**Scheme 1:**
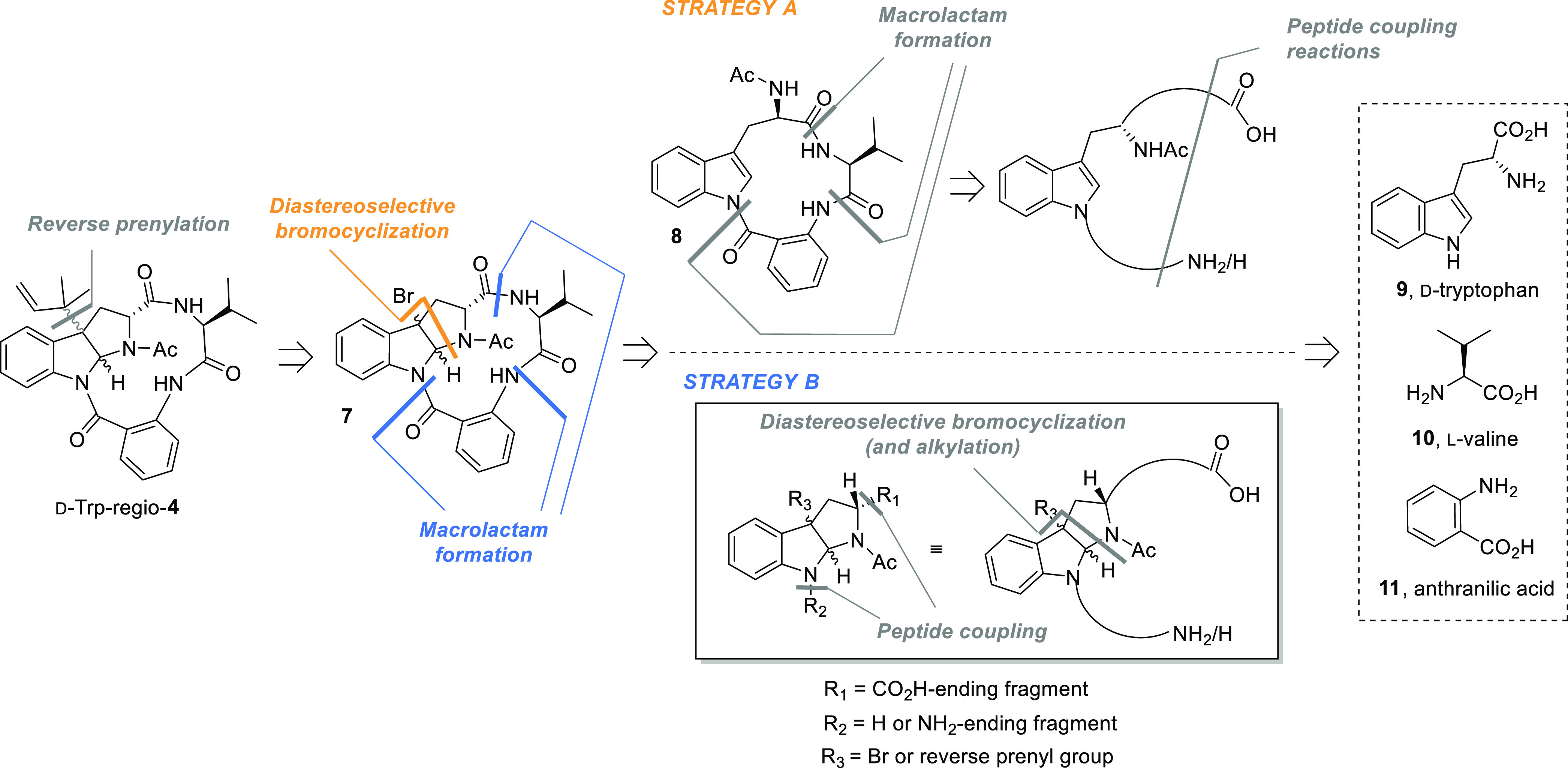
General Synthetic Strategies toward Putative Structures of Novofumigatamide (d-Trp-regio-**4**)

After completion of the synthetic targets of the first part of this project,^2^ we were convinced that the correct structure of novofumigatamide would display a connectivity between the atoms different from the one proposed for the original structure. Then, our next efforts were focused on the quest of new molecular skeletons that fulfilled the most representative two-dimensional (2D) NMR and ROESY correlations which guided the structural elucidation of the molecular skeleton of novofumigatamide.^1^ Most likely, the relevant ROESY correlation between the proton at C2 and the acetyl group would also be observed in the regioisomer depicted in Figure 2 (d-Trp-regio-*exo*-**4**), which shows a 12-membered ring macrocycle anchored to the hexahydropyrrolo[2,3-*b*]indole core through the indole nitrogen. Importantly, in this new skeleton proposal, in which the acetyl group and the macrolactam linking point have exchanged their positions within the molecule, most of the remaining ROESY, HMBC, and ^1^H-^1^H COSY correlations reported in the original manuscript would be maintained. As done in the preceding work,^2^ the different routes explored were named according to the last step selected to assemble the polycyclic structure of novofumigatamide, being type A strategies the ones based on diastereoselective bromocyclization–reverse prenylation reactions as last steps of the synthesis and type B strategies those based on macrolactam formation reactions. In addition, in both groups of routes, the reverse prenylation step could be accomplished at different stages of the synthetic sequences (Scheme 1).

The proposed constitutional isomer (d-Trp-regio-**4**) holds a unique structure possessing a pyrrolidinoindoline framework embedded in a larger macrolactam ring. In a literature search, we could find only two alkaloid natural products bearing a macrocyclic ring attached *via* the indole nitrogen of a tryptophan moiety. One of these natural structures is (+)-psychrophilin E (**5**), a cyclic tripeptide formed by tryptophan, proline, and anthranilic acid residues, the total synthesis of which was published in 2016 by Brimble and co-workers (Figure 2).^5^ To the best of our knowledge, this structure represents the only natural product containing a macrolactam ring attached through the indole nitrogen of the tryptophan moiety. We envisioned that the biosynthetic pathway toward this naturally occurring compound would be similar to the biosynthetic pathway toward the new structure proposed for novofumigatamide (d-Trp-regio-**4**), with an additional last step for our synthetic target involving the simultaneous formation of the hexahydropyrrolo[2,3-*b*]indole core and the prenylation at position C3.^6^ The second is (+)-nocardioazine A (**6**), whose first total synthesis was published by the Reisman group in recent years,^7^ which displays also a macrocycle, but connecting instead two subunits of pyrroloindolines fused through a diketopiperazine central framework (Figure 2). This macrocycle, which is not a macrolactam, links these subunits through the indole nitrogen of one of the hexahydropyrrolo[2,3-*b*]indole segments and the C3a bridged carbon of the other pyrroloindoline framework.

### Type A Strategies toward the *Exo* and *Endo* Diastereomers of d-Trp-regio-**4**

#### Route A.1. Formation of a 13-Membered Ring Macrolactam and a Subsequent Diastereoselective Bromocyclization

**Scheme 2:**
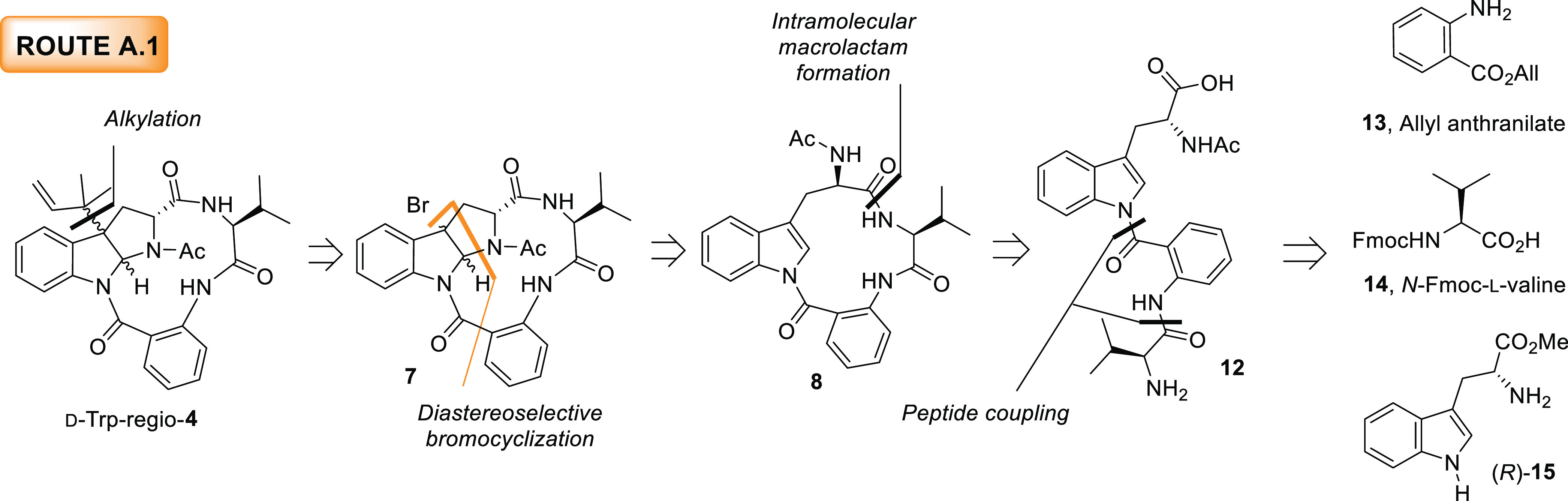
Retrosynthetic Analysis for the New Proposed Structure of Novofumigatamide (d-Trp-regio-**4**) following Route A.1

The chemical shift displayed by H11 in novofumigatamide suggests that the most likely relative configuration of the natural product is *endo*.^2^ Nevertheless, all of the routes were designed to provide access to both *exo* and *endo* diastereomers of the final product (d-Trp-regio-**4**). In the first route explored, route A.1, the attachment of the reverse prenyl group was delayed to the last step of the synthesis (Scheme 2). The immediate brominated precursor **7** could be obtained through a diastereoselective bromocyclization from 13-membered ring macrocycle **8**, an unprecedented transformation in the literature to the best of our knowledge, which, in turn, was expected to derive from intermediate **12***via* a cyclization reaction between the tryptophan and the valine edges. Finally, the latter acyclic compound would result from the assembly of d-tryptophan methyl ester (*R*)-**15** and commercially available allyl anthranilate (**13**) and *N*-Fmoc-l-valine (**14**). As explained above, this synthetic proposal is biosynthetically meaningful since (+)-psychrophilin E (**5**), a counterpart of macrocycle **8**, can be regarded as similar to a putative biosynthetic precursor of novofumigatamide in which the valine residue has been replaced by proline.

**Scheme 3:**
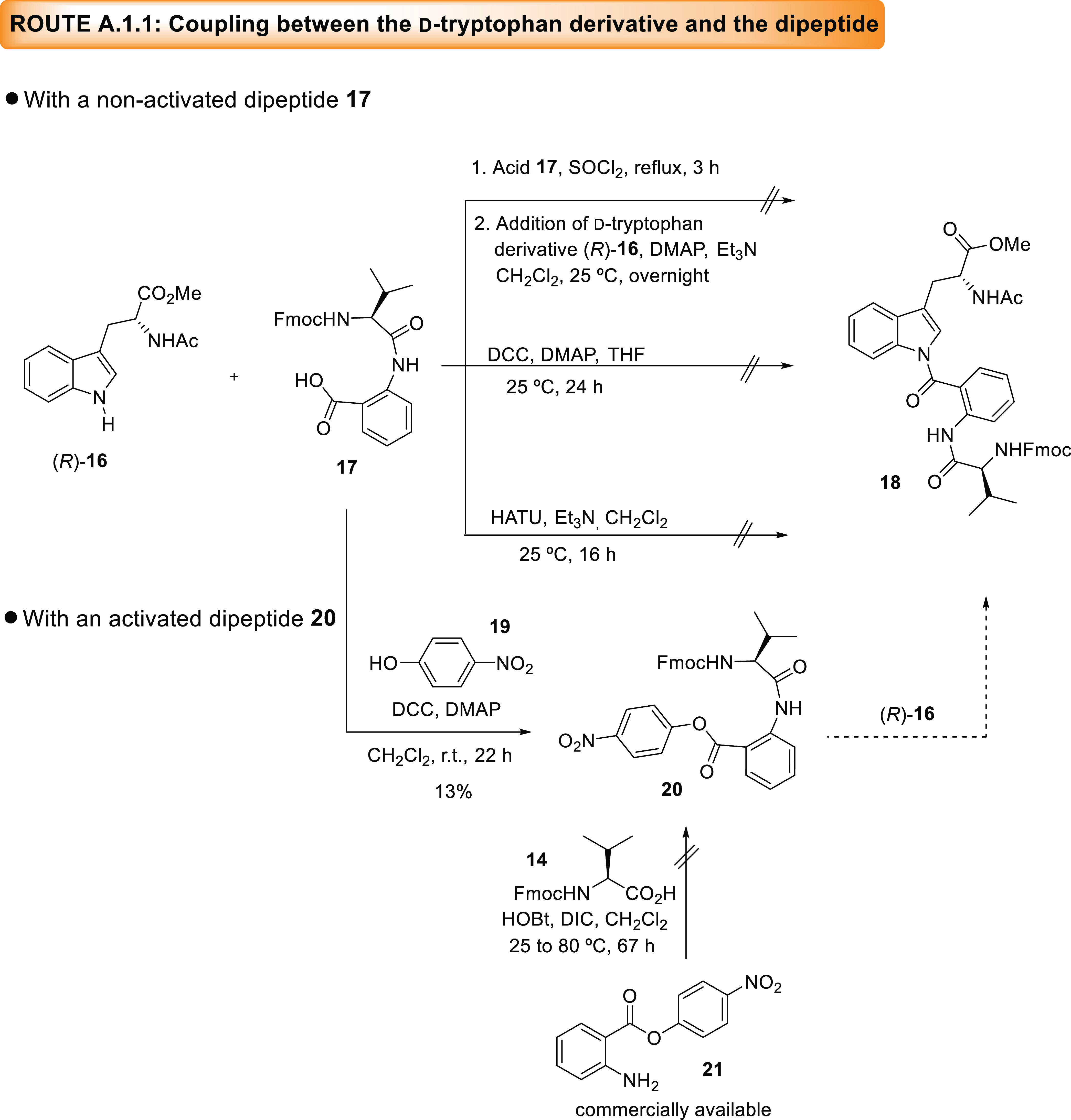
Synthetic Approaches to Acyclic Tripeptide Fragment **18**, Precursor of the New Proposed Structure for (-)-Novofumigatamide (d-Trp-regio-**4**) following Route A.1.1

Route A.1 started with the acetylation of the primary amine on d-tryptophan methyl ester (*R*)-**15** in the presence of acetic anhydride and Et_3_N at 80 °C, which afforded the corresponding product (*R*)-**16**^8^ (Scheme 3) in excellent yield (see the SI). To progress toward the synthesis of acyclic intermediate **12**, we envisioned that the attachment of the anthranilic acid and the valine units to this tryptophan derivative could be alternatively attained in two different ways. On the one hand, a dipeptide between these two units could be formed in advance and be later condensed with (*R*)-**16** after removal of the appropriate protecting group. On the other hand, both units could be added separately and sequentially to the tryptophan derivative (*R*)-**16**. The more convergent route, named Route A.1.1, began with the preparation of the *N*-Fmoc-protected dipeptide **17** from the fully protected precursor prepared in our previous manuscript (see the SI). With the two fragments (*R*)-**16** and **17** in hand the following condensation was assayed using different reaction conditions (Scheme 3).^5,9^ However, only decomposition products or recovered starting materials were obtained from the reaction mixture.

In a quest for methods to condense an anthranilic carboxylic acid and the indole nitrogen, we found out that the literature on the topic is scarce with only four reports dealing with the amide coupling between these two functionalities.^5,9−11^ An indirect way to attain this coupling is based on the use of a more activated *o*-nitrobenzoic acid and a further reduction of the nitro group to the corresponding aniline.^9^ Alternatively, isatoic anhydride could be used as a masked form of anthranilic acid since it is very reactive toward coupling thanks to the driving force provided by the release of both the ring strain and CO_2_ gas.^10,11^ When our attention was specifically focused on the coupling between the indole nitrogen of a tryptophan amino acid and anthranilic acid, we realized this transformation had been unprecedented until the publication of the total synthesis of (+)-psychrophilin E (**5**, Figure 2),^5^ where isatoic anhydride was employed as anthranilate surrogate. Other reports described the condensation of tryptophan derivatives with alanine or valine amino acids using the prior conversion of the carboxylic acid moieties onto the more activated *p*-nitrophenyl esters.^12^ Then, all of the examples reported in the literature, both for indoles and for tryptophan derivatives, highlight the relevance of converting the anthranilic acid into a more activated substrate.

**Scheme 4:**
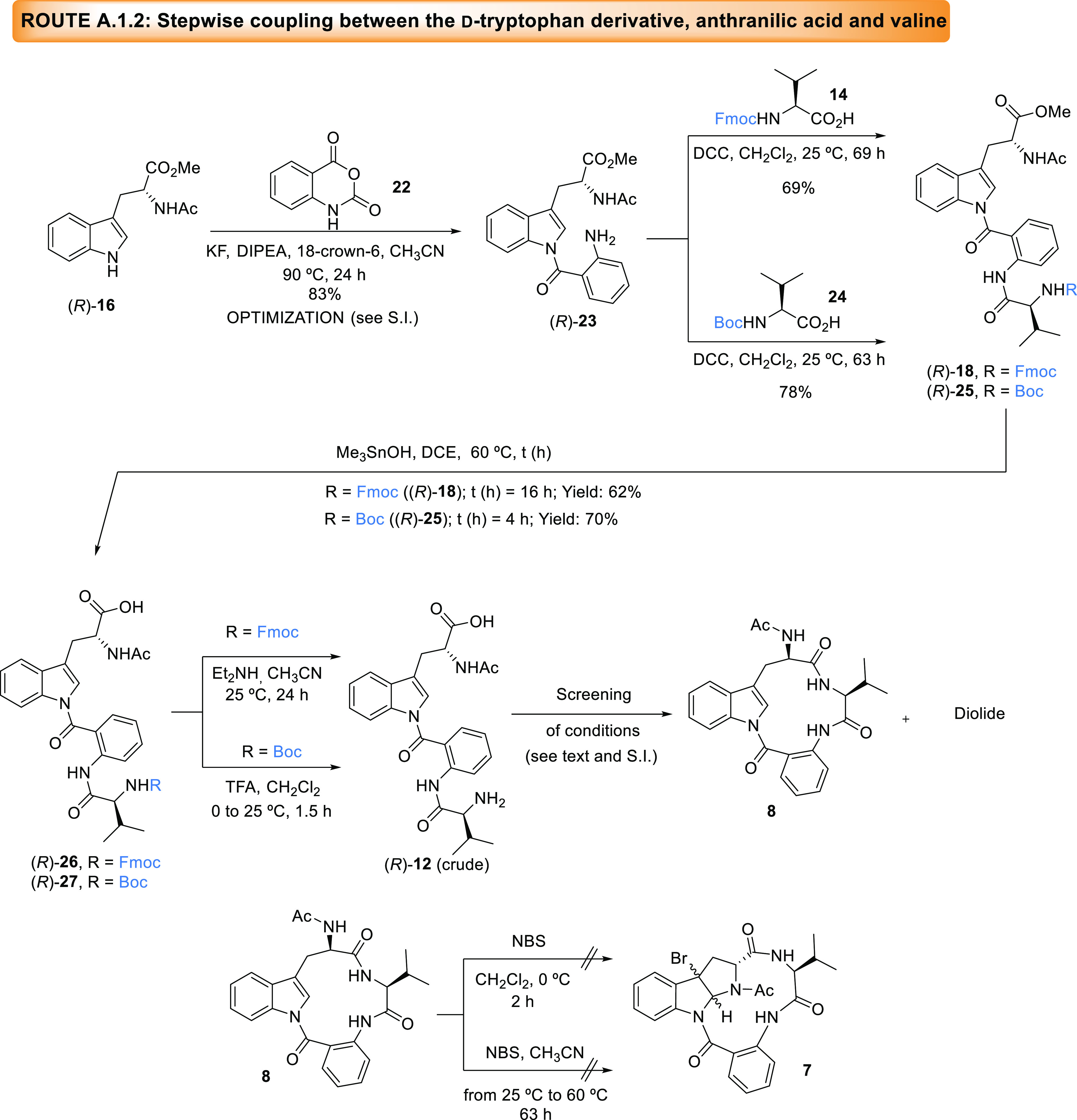
Synthesis of Macrolactam **8**, Precursor of the New Proposed Structure for (−)-Novofumigatamide (d-Trp-regio-**4**) following Route A.1.2

Given these literature precedents and the lack of success in the direct condensation of the *N*-Fmoc-protected dipeptide **17** and tryptophan derivative (*R*)-**16**, we decided to transform this intermediate **17** into a more activated substrate. The conversion of **17** into the corresponding *p*-nitrophenyl ester derivative **20** was first addressed. Unfortunately, with the conditions described in the literature for this purpose (*p*-nitrophenol **19**, DCC, and DMAP),^13^ the activated ester **20** was isolated in a very low yield. The alternative condensation of commercially available *p*-nitrophenyl anthranilate **21**(14) and *N*-Fmoc-valine **14** in the presence of two peptide coupling reagents failed to provide the desired activated dipeptide. These results made us abandon this route, and we turned our attention to the more divergent A.1.2 route in which both units, namely, anthranilate and valine, would be sequentially coupled to the tryptophan derivative (Scheme 4). At the outset, *p*-nitrophenyl ester anthranilate **21** was envisioned as a coupling partner. However, since the protection of the free aniline on this substrate under classical *N*-Fmoc protection conditions (Fmoc-Cl and NaHCO_3_ in dioxane/H_2_O) was unfruitful (data not shown), this substrate was discarded.

We then shifted our attention to isatoic anhydride (**22**) as anthranilic acid source and followed the experimental procedure reported in the total synthesis of (+)-psychrophilin E (**5**, Figure 2) for the condensation of anthranilic acid and a tryptophan derivative.^5^ This protocol is based on the generation of a “naked” fluoride anion in the presence of KF and 18-crown ether-6. This strong base deprotonates the indole nitrogen generating an indolide ion^15^ that reacts with isatoic anhydride at 60 °C. These conditions have been also successfully used for the coupling at room temperature between tryptophan derivatives and *p*-nitrophenyl ester derivatives of *N*-Cbz-protected alanine and leucine residues.^12^

The application of this methodology to our starting substrate (*R*)-**16** delivered the product (*R*)-**23** in a very low yield. However, after optimization of all of the reaction parameters (see the SI for further details), particularly the reaction temperature, the desired product could be isolated in 83% yield. The subsequent condensation was performed with commercially available *N*-Boc-valine (**24**) and *N*-Fmoc-valine (**14**), which gave access to two acyclic intermediates sensitive to different reaction conditions (acid or basic). Upon treatment of aniline **23** and these two valine derivatives with DCC in CH_2_Cl_2,_^5^ acyclic intermediates (*R*)-**18** and (*R*)-**25**, respectively, were obtained in good yields. Hydrolysis of methyl esters on these two intermediates using Me_3_SnOH in DCE at 60 °C provided the corresponding carboxylic acids (*R*)-**26** and (*R*)-**27** also in satisfactory yields.^16^ The following deprotection of the valine primary amine on (*R*)-**26** and (*R*)-**27**, respectively, in basic (Et_2_NH) or acidic (TFA) media, gave rise to the fully deprotected precursor (*R*)-**12**, ready to be converted into macrolactam **8** without further purification. The subsequent macrolactam formation was attempted varying several reaction parameters such as solvent (CH_2_Cl_2_, DMF), coupling reagent (HATU, HOAt, 6-Cl-HOBt), base (Et_3_N, DIPEA), equivalents of the reagents, protocols such as addition rates of the reagents (syringe pump, dropping funnel, syringe), and/or reaction times. Nevertheless, no satisfactory result was obtained. Since a tertiary amine-promoted *N*-Fmoc removal has been reported in the literature,^17^ a one-pot *N*-Fmoc deprotection-cyclization reaction was attempted with *N*-Fmoc derivative **26**, although without success (data not shown). The progress of the macrolactam formation was followed by analysis of aliquots in HPLC-MS, which showed the formation of the product (**8**), as well as a cyclic dimer or diolide arising from the intermolecular condensation of two molecules of the linear precursor and a subsequent macrolactam formation of this intermediate, and several other decomposition products. Furthermore, an epimerization of the product (**8**) and the diolide occurred over the reaction course, probably due to the sensitivity of these two products to the basic reaction media. As reported in the literature, not all of the conformations and configurations of the acyclic precursor assure cyclization.^5,7^ This fact has been proven along the synthesis of (+)-psychrophilin E (**5**, Figure 2) since from a mixture of epimers of the acyclic precursor, differing from **12** in the proline residue replacing the valine, only the (*S*)-tryptophan epimer reacted to give the product, being the corresponding (*S*)-macrolactam the more stable of the two. On the other hand, proline (as in (+)-psychrophilin E, **5** in Figure 2) usually adopts *cis*-amide geometries to favor cyclization.^18^ Our results agree with these observations since the (*R*)-tryptophan-containing precursor **12** did not react to furnish the desired macrolactam. Regardless of these disappointing results, a fairly pure fraction of the product was submitted to the bromocyclization reaction. The standard protocol in the presence of NBS and CH_2_Cl_2_ as solvent led to the recovery of part of the starting material accompanied by decomposition products. To force the formation of the inner ring, the reaction temperature was increased to 60 °C and CH_3_CN was used as the solvent instead. However, only the formation of byproducts during the reaction course was observed by HPLC-MS. The failure of this unprecedented bromocyclization and the poor efficiency of the formation of the macrocycle **8** encouraged us to design alternative synthetic routes.

### Type B Strategies toward the *Exo* and *Endo* Diastereomers of d-Trp-regio-**4**

#### Route B.1

**Scheme 5:**
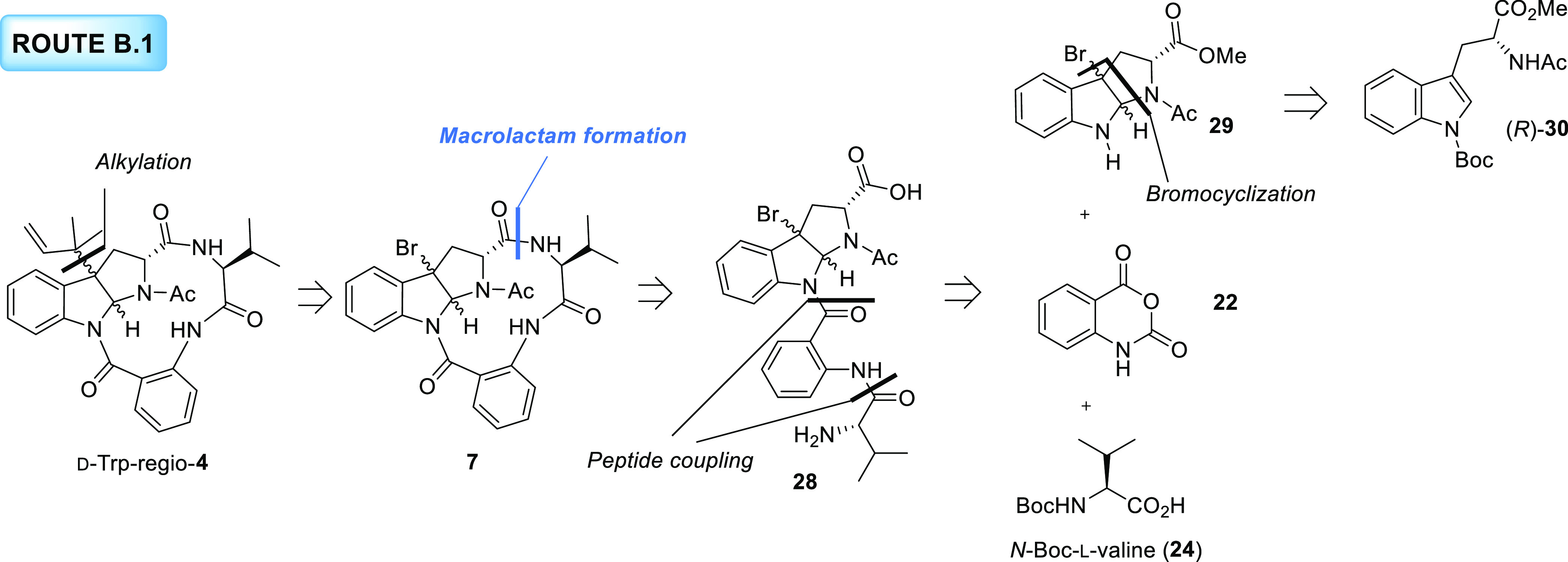
Retrosynthetic Analysis for the New Proposed Structure of Novofumigatamide (d-Trp-regio-**4**) following Route B.1

In a new retrosynthetic plan, attention was shifted toward the group of strategies B. The amide formation between the tryptophan and the valine units was selected as the key reaction leading to the synthetic product d-Trp-regio-**4** from precursor **28** (Scheme 5). Although in the original proposal the installation of the reverse prenyl group was postponed to the last step of the synthesis, addressed from cyclic precursor **7**, we envisioned that this group could be incorporated at other stages of the synthetic pathway (*vide infra*). Acyclic bromo precursor **28** would be accessed through the sequential coupling of the anthranilic acid and valine residues with bromopyrroloindoline **29**, as performed in Route A.1.2. Very likely the nucleophilicity of the indole nitrogen on this substituted pyrroloindoline is enhanced with respect to that of the tryptophan derivative (*R*)-**16** used in the previous route, which should facilitate the condensation with the remaining amino acid residues. Finally, in this retrosynthetic plan, the bromocyclization of tryptophan derivative (*R*)-**30** to furnish **29** was proposed as an early step of the synthetic route.

**Scheme 6:**
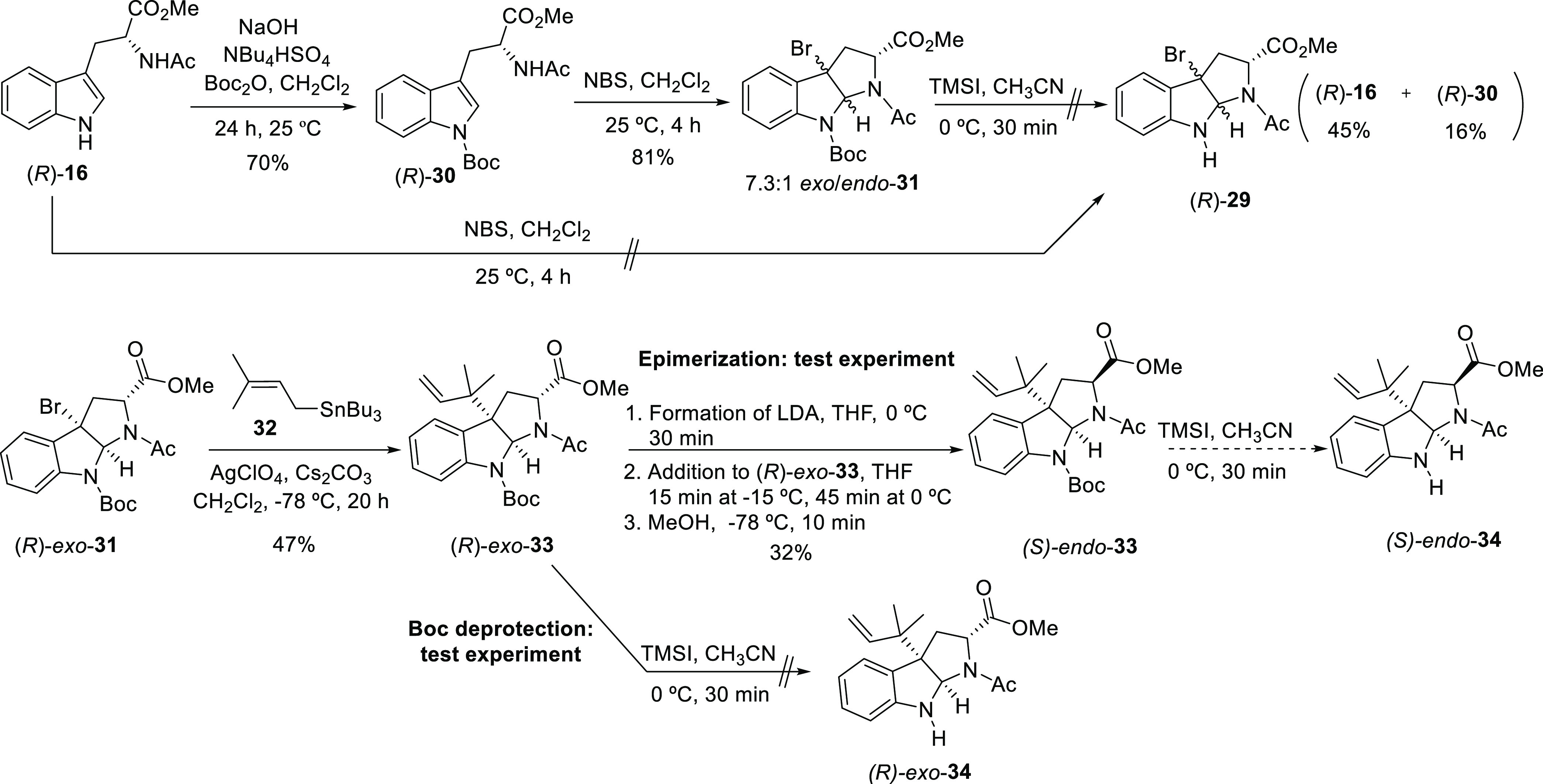
Synthetic Route toward Intermediates **29** and **34** following Route B.1

The new synthetic approach started with the protection of the indole nitrogen on (*R*)-**16** upon treatment with the standard reagents (Boc_2_O, NaOH, phase-transfer catalysis) (Scheme 6). *N*-Boc-tryptophan derivative (*R*)-**30** was then subjected to the classical protocol for the bromocyclization in the presence of NBS and without any other additive, which provided the desired bromopyrrolidinoindolines (*R*)-**31** in a ratio favorable to the *exo* isomer. Only two examples of bromocyclization of *N*-acetylated tryptophan derivatives have been described, although the protection of the indole nitrogen with a methoxy group or the absence of the α-enolizable proton were required to achieve efficient transformations.^19,20^ Subsequent *N*-Boc deprotection of the mixture of *exo* and *endo* brominated intermediates (*R*)-**31** in the presence of TMSI was unfortunately fruitless: tryptophan precursors (*R*)-**16** and (*R*)-**30** were isolated in 45% and 16% yields, respectively, after chromatographic purification. As reported in our previous work (DOI: 10.1021/acs.joc.2c01127), bromopyrroloindole moieties with unmasked pyrrole nitrogen are unstable compounds. With particular substrate (*R*)-**29**, which possesses the indole nitrogen unveiled, none of the diastereomers proved to be stable under these reaction conditions. As expected, an alternative direct bromocyclization of unprotected tryptophan derivative (*R*)-**16** in the presence of NBS delivered a mixture of undesired products containing bromine atoms at different positions of the aromatic system. With the aim of converting these intermediates into more stable molecules, *endo* and *exo* isomers of (*R*)-**31** were chromatographically separated and *R*-*exo*-**31** was reverse-prenylated using the conditions described in our previous manuscript (DOI: 10.1021/acs.joc.2c01127) with tributyl stannane (**32**) as nucleophile and AgClO_4_ as silver salt.^21^ As a test experiment to explore a different pathway to access the *endo* isomer, the *exo* prenylated compound (*R*-*exo*-**33**) was subjected to standard epimerization conditions using LDA as a base and a protonation with MeOH at low temperature (remark: the desired *R* -*endo*-**33** would be obtained from the *S*-*exo*-**33** isomer). Although this process, which resulted in a 32% yield of the desired (*S*)-*endo*-**33** diastereomer, was likely to be improved by increasing the reaction temperature, as demonstrated in Part I of this manuscript (DOI: 10.1021/acs.joc.2c01127),^2^ the route was followed by the removal of the *N*-Boc protecting group. As a test experiment, *N*-Boc deprotection on diastereomer (*R*)-*exo*-**33** was performed. Unfortunately, treatment of this substrate with TMSI in CH_3_CN at 0 °C led to a complex mixture. With the latter example, the instability of unprotected bromopyrroloindoles became once more notable, though surprisingly, (*R*)-*exo*-**33** possesses the C3a position blocked with an alkyl group.

The low yields obtained for the chemical transformations of this route, the lack of efficient access to *endo* isomers, and the instability of intermediates with unprotected indole nitrogen atoms, required for the subsequent anchoring of the anthranilic unit, forced us to discard this synthetic option.

#### Route B.2

**Scheme 7:**
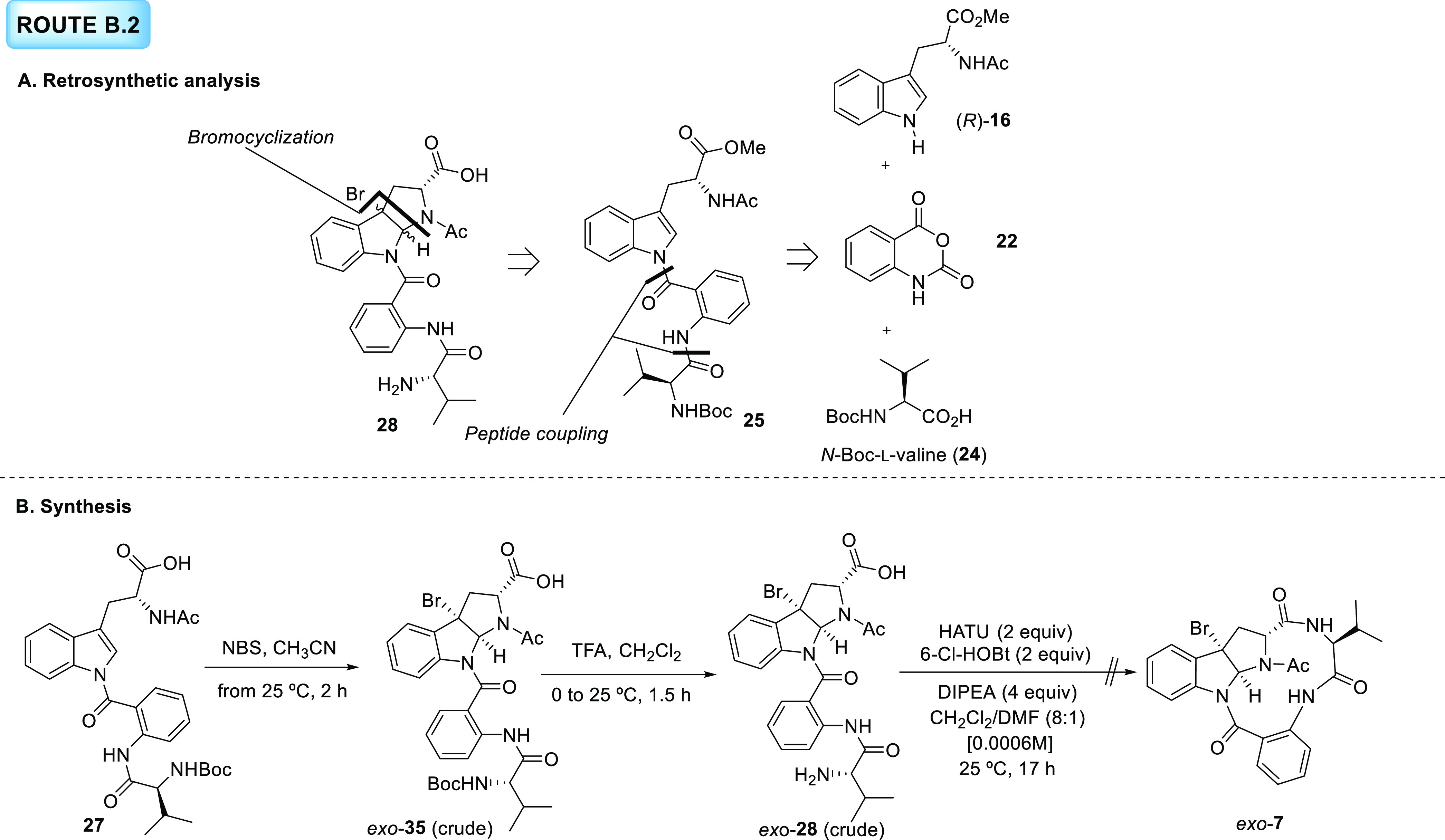
**A**. Retrosynthetic Analysis for Route B.2. **B**. Synthesis toward Macrolactam **7** Following Route B.2

Similar shortcomings were also found in our previous manuscript (DOI: 10.1021/acs.joc.2c01127), so following a similar optional strategy, we proposed the construction of linear intermediate **28***via* a diastereoselective bromocyclization of tripeptide **25**, which has been already synthesized in route A.1.2 from tryptophan derivative (*R*)-**16**, isatoic anhydride (**22**), and *N*-Boc-l-valine (**24**) building blocks (Scheme 7A).

**Scheme 8:**
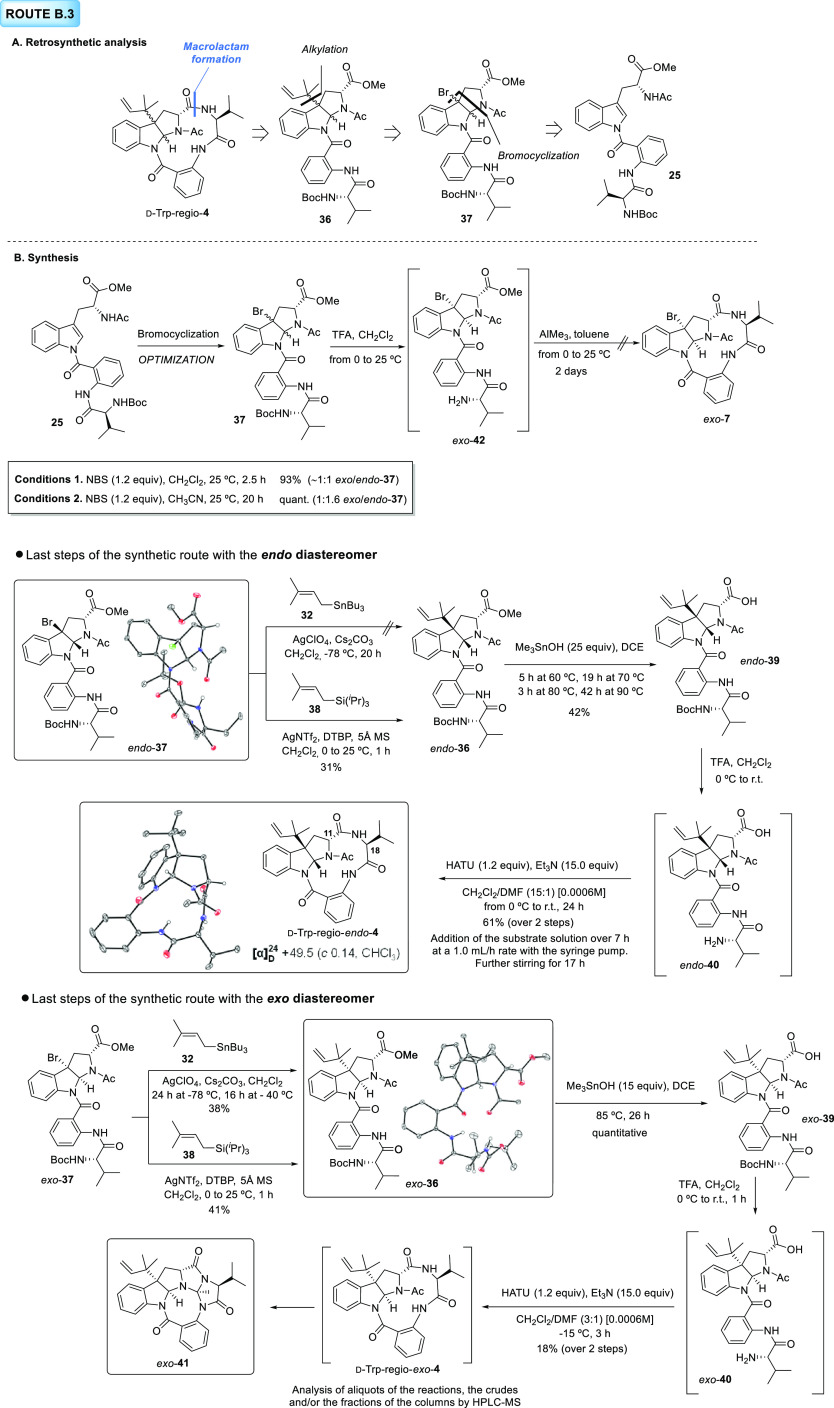
A. Retrosynthetic Analysis for Route B.3. B. Total Synthesis of *Exo* and *Endo* Isomers of d-Trp-regio-**4** Following Route B.3 The ORTEP diagrams of the X-ray structures of d-Trp-regio--*endo*-**4** and *endo*-**37** are represented with the ellipsoids drawn at 30% probability level, whereas that of *exo*-**36** is represented with the ellipsoids drawn at 50% probability level.

Taking advantage of the large amounts of intermediate **27** prepared in route A.1.2, bromocyclization was initially tested on this substrate bearing a free carboxylic acid (Scheme 7B). Notably, treatment of **27** with NBS in CH_3_CN for 2 h led to the formation of the *exo* product (*exo*-**35**) as a single diastereomer. While the crude for this *exo* isomer showed a moderately pure ^1^H NMR spectrum, the handling of this compound (purification by column chromatography and/or the use of CDCl_3_ as NMR solvent) caused its full decomposition. To avoid that, the reaction crude was used in the next step without further purification. Even though *N*-Boc removal by exposure of *exo*-**35** to TFA showed decomposition, which demonstrated the instability of the previous intermediate in acidic media, the crude of this reaction was immediately submitted to macrolactam formation conditions in the presence of HATU and 6-Cl-HOBt as coupling reagents.^5^ As expected, decomposition products were observed over the reaction course, a fact that was confirmed after an unsuccessful attempt to isolate and identify any product of the reaction. At this point of our research, the instability of bromopyrroloindole *exo*-**35** was ascribed to the simultaneous presence of the bromine and the carboxylic acid functional groups in close proximity within the molecule. Thus, to skip this intermediate, a new retrosynthetic analysis was envisaged (Scheme 8A).

#### Route B.3

In our new plan, the macrolactam formation would be postponed to the last step of the synthesis from acyclic intermediate **36** after ester hydrolysis and *N*-Boc deprotection. The stability of the CO_2_H-NH_2_-acyclic precursor of d-Trp-regio-**4** (not shown) will be secured by the early replacement of the bromine atom on **37** by a reverse prenyl group. Finally, we envisioned that fully protected bromopyrroloindole **37** would be a stable intermediate obtained from previously prepared linear tripeptide **25**. As observed in previous experiments of the current manuscript and reported also in Part I (DOI: 10.1021/acs.joc.2c01127),^2^ diastereoselective bromocyclization with fully protected precursors occurs readily and without decomposition problems related to the stability of the compounds.

**Figure 3:**
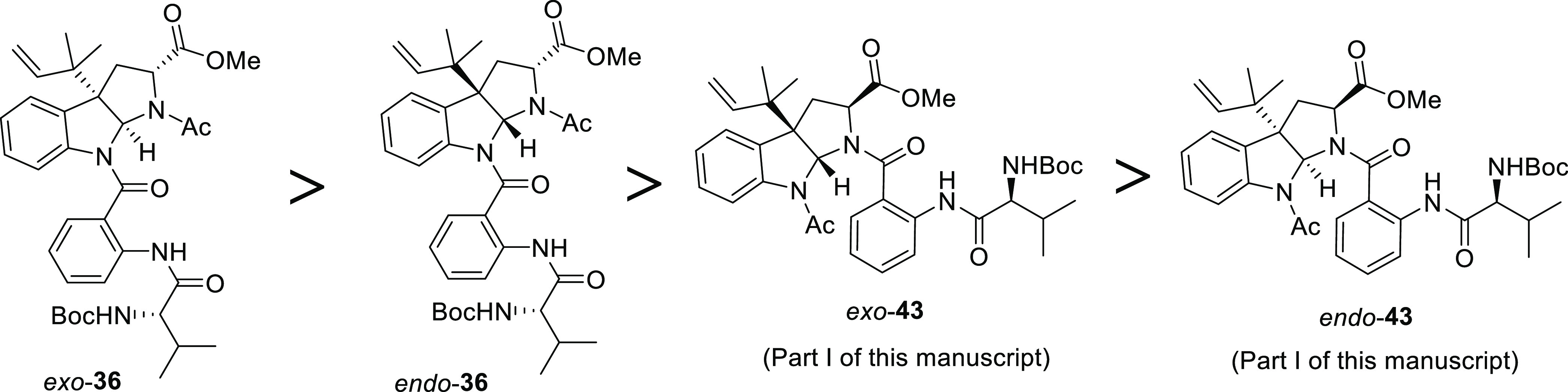
Reactivity toward the hydrolysis of methyl esters on pyrroloindoline-bearing intermediates.

The new synthetic plan began with optimization studies aimed at finding proper conditions to selectively direct the stereochemical course of bromocyclization of **25** toward *exo*- or *endo*-**37** diastereomeric products (Scheme 8B). The results reported in Part I of the current manuscript suggest that this is not a straightforward task.^2^ Examples of bromocyclization reactions in which a dioxopiperazine acts as the nucleophile can be found in the literature. In these examples, diastereoselectivity can be tuned toward the *exo* or *endo* isomers by varying the solvent, reaction temperature, or brominating reagent.^22−24^ Following these precedents, different conditions were screened, but the diastereoselectivity proved to be poor with our substrate **25**. The first set of reaction conditions depicted in Scheme 8B, conditions 1, allowed us to obtain an almost equimolar mixture of the *endo*/*exo*-**37** isomers using NBS in CH_2_Cl_2_ as solvent and room temperature. The replacement of the solvent by CH_3_CN (conditions 2) slightly shifted the *exo*/*endo* bias toward the *endo* product. After separation of both isomers by column chromatography, the remaining steps of the synthetic pathway B.3 were carried out with each diastereomer. Reverse prenylation of *endo*-**37**, whose structure was confirmed by X-ray diffraction analysis, was first attempted using prenyl stannane **32** as a nucleophile. However, most of the starting material was recovered from the reaction mixture, together with small amounts of the corresponding alcohol derivative resulting from trapping of the intermediate carbocation by water.^21^ Fortunately, the employment of the analogous prenyl silane reagent **38** delivered the corresponding product (*endo*-**36**) in a 31% yield, which falls within the range of yields obtained in this transformation for complex substrates, as explained in Part I (DOI: 10.1021/acs.joc.2c01127).^2,25^ Methyl ester saponification, following the standard Me_3_SnOH-based protocol used throughout this synthetic project,^16^ turned out to be more cumbersome than expected. Taking into consideration the conclusions drawn from our previous results with complex substrates having the methyl ester functionality connected to C2 of hexahydropyrrolo[2,3-*b*]indole skeletons, this sort of structures should undergo hydrolysis under milder reaction conditions.^2^ When *endo*-**36** was submitted to the standard conditions, the problems found before with regioisomeric counterparts were encountered. Given the lack of reactivity of this substrate at the reported temperature (60 °C), this was progressively increased to 90 °C. Furthermore, up to 25 equiv of the reagent were portionwise added to reach full conversion. This substrate required also long reaction times, which led to the concomitant formation of degradation byproducts and a drop in the yield. In comparison with the regioisomeric *endo* diastereomer prepared in Part I (DOI: 10.1021/acs.joc.2c01127) of this manuscript (Figure 3, *endo*-**43**),^2^ in which the valine-anthranilate fragment is tethered to the pyrrole nitrogen, the hydrolysis of *endo*-**36** did not require intermediate workups to remove excess of the tin hydroxide and other byproducts interfering in the reaction progress. Thus, these results confirm the influence of the steric bulk in the proximity of the ester functionality on the efficiency of the reaction. Next, *N*-Boc cleavage in *endo*-**39** in the presence of TFA provided fully deprotected *endo*-**40** intermediate, which was used as a crude product in the final macrolactam formation step. Although the efficiency of this reaction would be difficult to predict beforehand given its dependence on the configuration and the preferred conformation of the acyclic precursor, among other factors,^2,18,26,27^ we surmised that this cyclization process would be more challenging than the analogous reactions affording the regioisomeric structures originally proposed for novofumigatamide (d-Trp-*exo*-**1**, Figure 1). We rationalized that this would be mainly due to the restrictions imposed by the rigid pyrroloindoline skeleton and the ring strain of the newly formed polycyclic structure. Consequently, with the aim of avoiding competing intermolecular condensation reactions, we selected a protocol based on the use of very high dilution conditions ([6 × 10^–4^ M]) and a very slow addition of the HATU-activated carboxylic acid intermediate, generated from *endo*-**40**, to a solution of the base (Et_3_N). Gratifyingly, the desired final product (d-Trp-regio-*endo*-**4**) was obtained with a good yield over the two steps. Unfortunately, the spectroscopic data did not match those of the natural product. Meaningfully, the ^1^H NMR spectrum of d-Trp-regio-*endo*-**4** recorded at 298K showed defined peaks for all of the protons of the molecule, which is an indicator of the rigid skeleton of the structure and a feature that is likely shared with natural novofumigatamide, given the highly resolved NMR spectra shown by the natural product. The specific optical rotation for this new synthetic product showed a positive value, opposite in sign to that of the natural product, which is in line with our previous observations for final structures arising from d-tryptophan and l-valine (Figure 1 and Part I; DOI: 10.1021/acs.joc.2c01127).^2^ Fortunately, we were able to obtain suitable crystals for X-ray diffraction analysis, which confirmed the structure of this *endo* final product. Surprisingly, peaks for diagnostic H11 and H18 α-enolizable protons have exchanged their position in the ^1^H NMR spectrum with respect to the equivalent protons in the original constitutional isomeric structures proposed for novofumigatamide (see Part I:^2^ novofumigatamide d-Trp-*Exo*-**1**, *N*-Boc-*exo*-novofumigatamide d-Trp-Boc-*Exo*-**2**, l-Trp-*Endo*-**11**, and l-Trp-*Exo*-**1**; DOI: 10.1021/acs.joc.2c01127). Moreover, unusually deshielded chemical shift for *endo* H11 (δ_H11_ 5.32–5.13 ppm) and shielded chemical shift for H18 (δ_H18_ 4.21 ppm) were observed.

The same synthetic pathway was followed to prepare the *exo* diastereomer (d-Trp-regio-*exo*-**4**). Reverse prenylation of bromopyrroloindole *exo*-**37** employing any of the two conditions used for this purpose all over this project afforded the corresponding product *exo*-**36**, the structure of which was also corroborated by single-crystal X-ray diffraction analysis. The difference in reactivity shown by both isomers when prenyl tributyl stannane **32** was used as the (reverse) prenyl group source is highly remarkable: although *endo*-**37** did not react in the presence of this nucleophile, *exo*-**37** gave rise to the product in a yield that is in agreement with reported literature values and also with our previous results, although slightly lower than the yield obtained with prenyl silane **38**, which seems to be a more general nucleophile. The different behavior toward alkylation exhibited by the *exo* and *endo* diastereomeric intermediates prepared during this project (Part I of this series; DOI: 10.1021/acs.joc.2c01127)^2^ and by the different molecular skeletons reported in the literature suggest that the reverse prenylation is a “diastereospecific” and substrate-dependent reaction.^21,25^ As predicted beforehand, methyl ester on *exo*-**36** was hydrolyzed under milder reaction conditions than that of the corresponding *endo*-**36** diastereomer since a lower number of Me_3_SnOH equivalents and a shorter reaction time were required to complete the reaction. Furthermore, an excellent yield was obtained for the isolated product, namely, carboxylic acid *exo*-**39**. This observation is also in accordance with our previous experiments involving *exo* and *endo* diastereomeric regioisomers **43**, which possess the valine-anthranilate fragment anchored through the pyrroline nitrogen (Figure 3). The comparison of the four substrates (two pairs of diastereomeric regioisomers) reveals that both the proximity of the bulky group (valine-anthranilate chain) to the methyl ester and the configuration of the substrate determine the efficiency of the reaction, with the *endo* isomer with the more sterically crowded environment around the ester being the least prone to react under these reaction conditions.

The final two steps of the synthetic sequence were performed next. TFA-promoted *N*-Boc removal on *exo*-**39** provided a crude mixture containing the fully deprotected intermediate *exo*-**40**, which was used in the final macrolactamization step without further purification. Although several conditions for the macrocyclization were screened, the desired final product d-Trp-regio-*exo*-**4** could not be isolated (see the SI for further details). HPLC-MS analysis of aliquots of the reaction, the crude or the column fractions revealed the presence of the product (d-Trp-regio-*exo*-**4**) in trace amounts, together with the diolide and an unknown compound with a molecular mass of 498.286 g/mol, likely resulting from the loss of two molecules of water during the cyclization process. In addition, several decomposition products were also detected in the reaction mixture. Surprisingly, when the temperature of the macrolactam formation was decreased to −15 °C and the reaction time shortened to 3 h, the unknown compound (*exo*-**41**) was obtained as the major product. The availability of larger amounts of this undesired byproduct allowed establishing its identity by full spectroscopic characterization (Scheme 8). The chemical structure of *exo*-**41** suggested a double condensation. First, between the free carboxylic acid and the free primary amine giving rise to the desired final *exo* product (d-Trp-regio-*exo*-**4**), and second between the carbonyl group of the *N*-acetyl group and the *N*-amide of the anthranilic unit, in close proximity, to afford an intermedium acyliminium ion, further trapped by the second competing amide nitrogen nucleophile. The release of water during the formation of this putative intermediate would take place thanks to the concomitant proton abstraction of the remaining NH-amide. To confirm the identity of this new polycyclic structure, the carbon chemical shift of the newly formed quaternary center (**C**CH_3_NNN) played a key role since this carbon appeared at δ 91.2 ppm as a singlet and showed a significant HMBC correlation with the directly attached methyl group and with the proximal proton of the bridged carbon on the pyrroloindoline framework (CNNH). Structurally similar chemical functionalities found in the literature display similar chemical shift values.^28^ The stereochemistry of this new quaternary center was tentatively assigned to (*S*)- **C**CH_3_NNN, as depicted in Scheme 8, based on a weak NOESY correlation between the methyl group and the bridged carbon on the pyrroloindoline framework (CNNH), observed in a partially decomposed sample (see the SI).

Further attempts to obtain the final product d-Trp-regio-*exo*-**4** also met with failure. A direct amide formation from ester *exo*-**42** under AlMe_3_ catalysis was attempted (Scheme 8). Upon treatment with TFA, the *N*-Boc group on *exo*-**37** was removed and the crude of this reaction was subjected to the amide formation protocol previously reported for the synthesis of (+)-aszonalenin.^29^ Differently, a more diluted solution was employed to avoid the formation of the diolide and/or other oligomerization products. Monitoring this reaction by HPLC-MS revealed the lack of reactivity of the starting material after stirring for 48 h (Scheme 8B).

#### Route B.4. Alternative Routes toward d-Trp-regio-*exo*-**4**

**Scheme 9:**
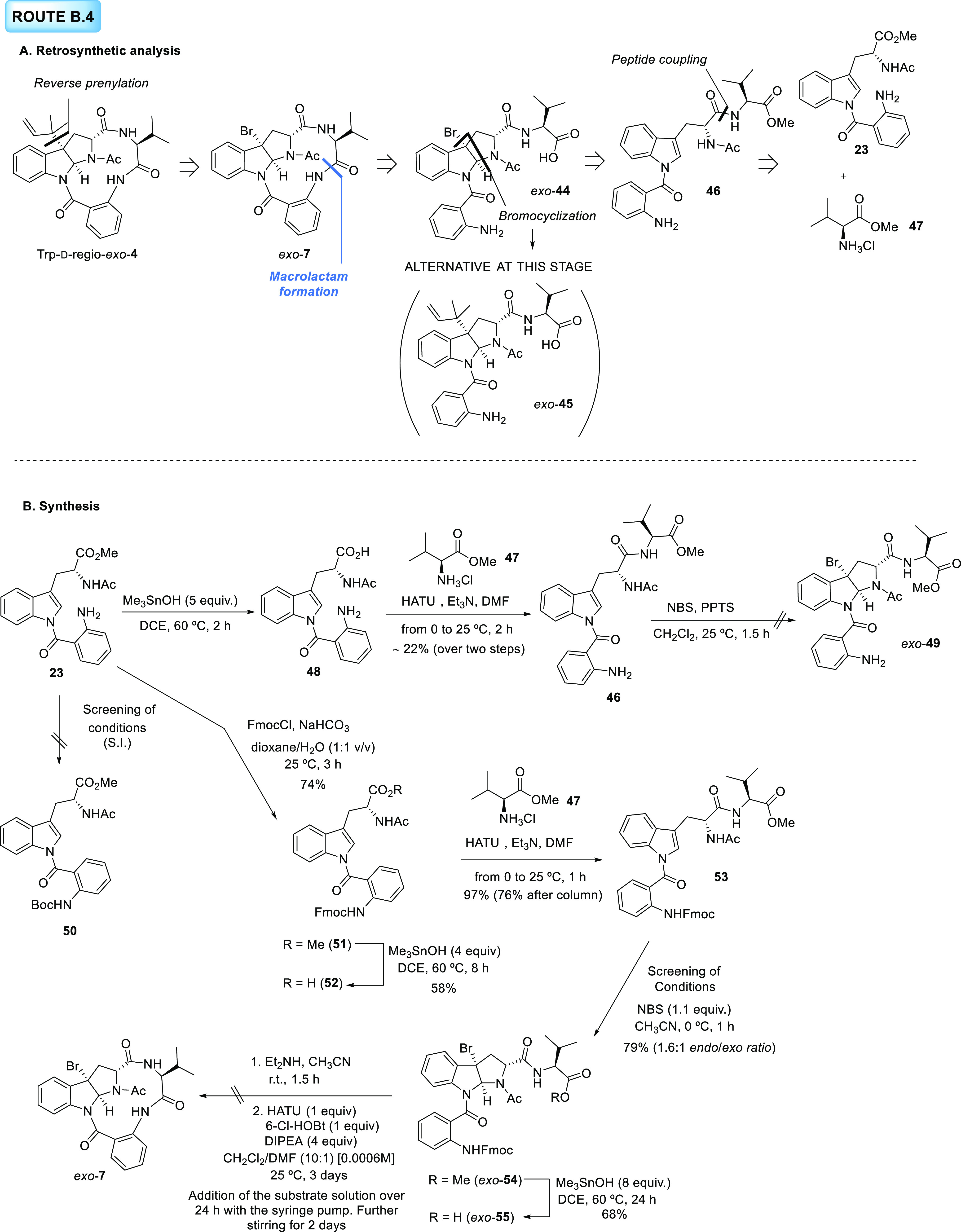
**A**. Retrosynthetic Analysis for Route B.4. **B**. Synthetic Approach toward d-Trp-regio-*exo*-**4** Following Route B.4

All of the drawbacks encountered in the previous synthetic route forced us to consider a new pathway to approach d-Trp-regio-*exo*-**4** differing from route B.3 in the amide formation reaction selected to close the macrolactam ring. In this new route, B.4, the retrosynthetic scission of the macrolactam through the amide bond between the anthranilic unit and the valine framework will produce open-chain precursor *exo*-**44** (Scheme 9A). The stability of bromopyrroloindole **44**, already possessing the unprotected amino and carboxylic acid functionalities, would determine the synthetic stage at which the prenyl group should be introduced. According to this assumption, reverse-prenylated compound *exo*-**45** would also be a possible intermediate of this route. Both pyrroloindole structures, namely, *exo*-**44** and *exo*-**45**, would be obtained from acyclic precursor **46** after a diastereoselective bromocyclization, and alkylation in the case of **45**. Precursor **46**, in turn, originates from the condensation of tryptophan derivative **23**, already used in the previous routes, and commercially available l-valine methyl ester hydrochloride **47**. Our new synthetic proposal started with the saponification of methyl ester **23** using the standard protocol, which delivered the corresponding product **48**. Given the high polarity exhibited by this product and the inherent difficulties of its purification, the crude mixture was immediately used in the following step. Condensation of **48** with valine methyl ester hydrochloride **47** in the presence of HATU and Et_3_N produced impure tryptophan derivative **46** in a scant 22% yield over the two steps. When this intermediate was treated with NBS and PPTS in CH_2_Cl_2_ to promote a selective bromocyclization biased toward the *exo*-**49** diastereomer, only decomposition byproducts were observed by ^1^H NMR. Most likely, the failure of this pathway could be ascribed to the free aniline group on the anthranilic unit, and therefore protection of this group on **23** was next addressed as a first step. However, since attempts to protect the aniline on **23** with an *N*-Boc group were fruitless (see the SI for further information), our attention turned to the incorporation of an *N*-Fmoc group. Classical conditions to achieve the protection of the molecule with this group (Fmoc-Cl, NaHCO_3_ in dioxane/H_2_O) afforded the *N*-Fmoc-protected derivative **51** in a 74% yield. The subsequent hydrolysis promoted by Me_3_SnOH proved to be problematic due to the lability of the *N*-Fmoc groups under the reaction conditions. The equivalents of the reagent were portionwise added to adjust the amount required to reach full conversion but only a moderate 58% yield was obtained. Next, coupling of carboxylic acid **52** with valine methyl ester hydrochloride **47** using the conditions described above resulted in the formation of adduct **53** as a highly pure crude in an excellent yield, which decreased after purification by column chromatography, probably due to the loss of the *N*-Fmoc-protecting group. Despite the synthetic efforts to optimize the following bromocyclization reaction, both isomers (in a 1.6:1 *endo*/*exo* ratio) could not be obtained in a highly pure form, which we anticipated would cause problems of reactivity in the next steps. After separation of both diastereomers, *ex*o-**54** was submitted to our saponification protocol, which required the addition of 8 equiv of the tin hydroxide reagent and led to a moderate yield of 68% due to the formation of decomposition products derived from the *N*-Fmoc loss. Remarkably, compound *exo*-**55** proved to be stable despite the presence of both the bromopyrroloindole moiety and the free carboxylic acid. Unlike structures *exo*-**35** and *exo*-**28** prepared in route B.2, which showed low stability, the two functionalities on *exo*-**55** are not in spatial proximity, and this fact could explain the difference in stability between both types of structures. The next two steps of the sequence were sequentially performed without isolation of the intermediate. *N*-Fmoc deprotection of *exo*-**55** by treatment with Et_2_NH in CH_3_CN proceeded smoothly, although decomposition products were observed over the reaction course. Final macrolactam formation with the crude mixture of the previous reaction was accomplished using a combination of coupling reagents (HATU, 6-Cl-HOBt) and a slow addition with a syringe pump. Nevertheless, degradation products were mostly detected by HPLC-MS. This failure and the low yields obtained throughout the synthetic sequence due to the *N*-Fmoc loss made us also abandon this route.

At this stage of the project, several reasons were devised to explain the failure of all of the synthetic approaches toward d-Trp-regio-*exo*-**4**; (i) the instability of the final product under the reaction conditions; (ii) its intrinsic high energy due to the stress imparted by the ring strain on its chemical structure; (iii) conformational and/or configurational factors that precluded the cyclization process; and (iv) a combination of the previous factors. A structure lacking stability cannot be regarded as a plausible candidate to be the natural product, which showed long-term stability in deuterated solvents and made possible the full characterization of the minute amounts isolated from the natural source.^1^

#### Route B.3 toward the Exo and Endo Diastereomers of l-Trp-regio-**4**

**Scheme 10:**
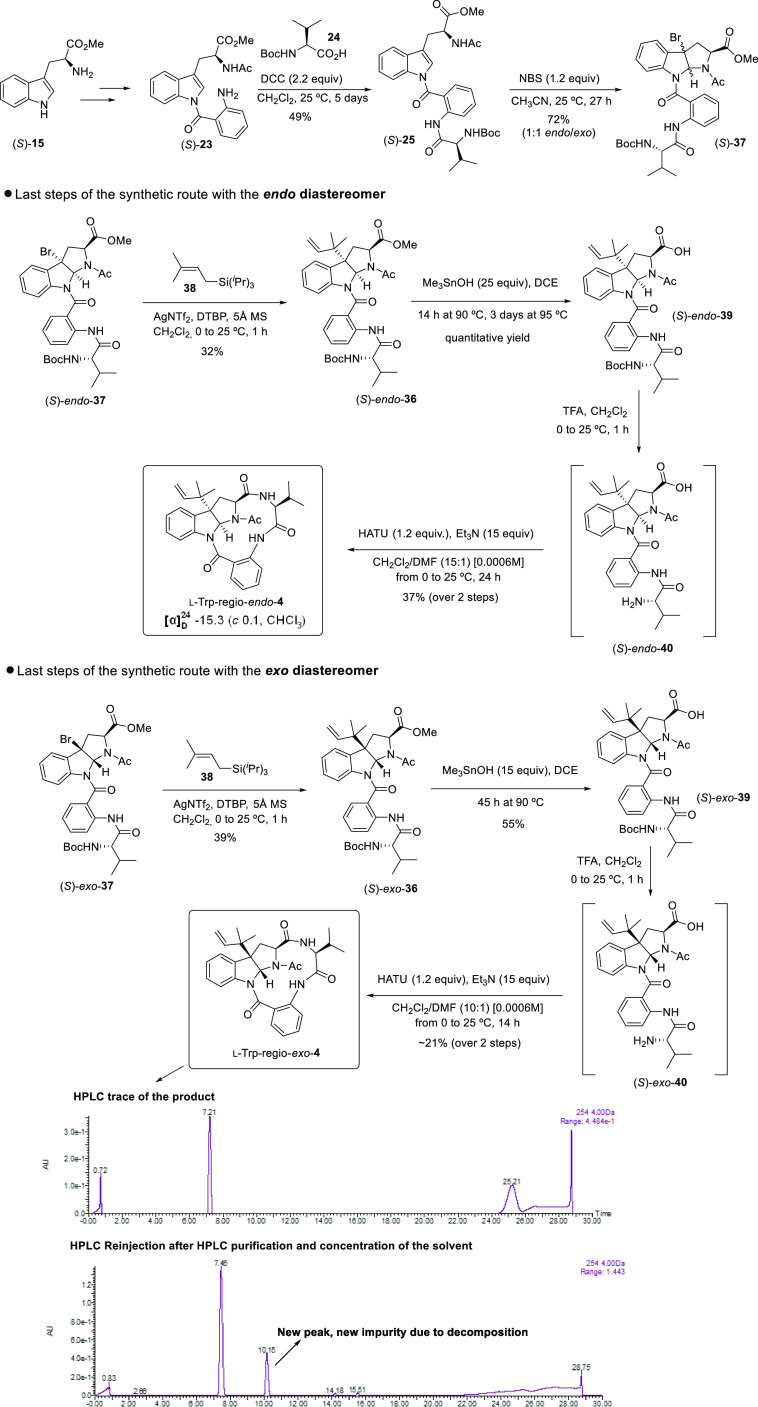
Total Synthesis of *Exo* and *Endo* Isomers of l-Trp-regio-**4** Following Route B.3

Our strong conviction that the natural product has its stereochemical origin on l-tryptophan^2^ encouraged us to address the total synthesis of the corresponding l-Trp-regio-*endo*-**4** and l-Trp-regio-*exo*-**4** diastereomers. Scheme 10 displays the total synthesis of both diastereomers following route B.3. Enantiomer (*S*)-**23** was prepared from l-tryptophan methyl ester (*S*)-**15** following the same chemical sequence used for the *R*-series. The subsequent condensation with *N*-Boc-valine **24** in the presence of DCC was not as efficient as the same condensation affording the diastereomeric product since the corresponding diastereomer (*S*)-**25** was obtained in a moderate 49% yield after 5 days of reaction. At this point of the research project, our interest was mostly focused on reaching the final products to confirm or discard the match of these structures with the natural product, so further optimizations were not addressed along this synthetic route. Diastereoselective bromocyclization employing conditions 1 depicted in Scheme 8 (NBS in CH_3_CN at 25 °C) furnished a 1:1 mixture of *endo*/*exo* (*S*)-**37** diastereomers, which were chromatographically separated to continue the route independently with each of them. Alkylation of (*S*)-*endo*-**37** and (*S*)-*exo*-**37** using prenyl triisopropyl silane **38** as nucleophile exhibited the same trend as d-tryptophan-derived intermediates since similar yields were obtained for the (*S*)-*endo*-**36** and the (*S*)-*exo*-**36** diastereomers. Methyl esters of both isomers (*S*)-**36** were slightly less reactive toward the Me_3_SnOH-promoted hydrolysis than their diastereomeric counterparts, which further emphasizes the importance of the configuration of these molecules on their reactivity. The subsequent *N*-Boc removal on **39** upon acidic treatment gave rise to (*S*)-*endo*-**40** and (*S*)-*exo*-**40** products, which without purification were subjected to the macrolactamization conditions to afford the final molecules. Using the same protocol described above for the cyclization of the acyclic precursor, (*S*)-*endo*-**40** furnished the desired final synthetic product l-Trp-regio-*endo*-**4** in a 37% yield over the two steps. Unfortunately, the NMR spectra of this new final product did not concur with the spectra of the natural product, although, as observed for d-Trp-regio-*endo*-**4** and the natural product, sharply defined signals in the ^1^H NMR spectra recorded at 25 °C were observed. In addition, as reported above for d-Trp-regio-*endo*-**4**, ^1^H NMR chemical shifts for diagnostic H11 and H18 showed surprisingly atypical values: H-*endo* fell within the range of δ_H11_ 5.32–5.13 ppm, whereas α-proton for the valine unit appeared at an unusual upshielded value of δ_H18_ 3.18 ppm, with a difference of more than 1 ppm with respect to the equivalent proton in d-Trp-regio-*endo*-**4**. As expected for an l-tryptophan-derived final product, optical rotation showed a negative value.

Given the problems encountered to accomplish the macrolactamization toward d-Trp-regio-*exo*-**4** from acyclic precursor (*R*)-**40**, likely attributed to the instability of the final product, the addition of a solution of the activated carboxylic acid (*S*)-*exo*-**40** to the solution of the base was performed, as previously described for the (*R*)-**40** diastereomer, over a period of 14 h, but the reaction was only further stirred for 1 h to avoid further decomposition of the final product. To our surprise, the mass of the product was detected in the reaction mixture by HPLC-MS. After purification by column chromatography, a moderately pure fraction of the final product l-Trp-regio-*exo*-**4** could be isolated and a ^1^H NMR acquired. Attempts to further purify this fraction by HPLC to obtain a suitable sample for full characterization were unfruitful since the product decomposed quickly upon handling. Reinjection of the fraction obtained after HPLC purification confirmed the instability of the product since another peak arising from product degradation was observed (Scheme 10). Fortunately, the low-quality ^1^H NMR recorded for this final synthetic product l-Trp-regio-*exo*-**4** was sufficient to allow discarding this skeleton as that of the natural product. A comparison between route B.3 for d- and l-tryptophan series revealed important differences in the reactivity and stability between the diastereomeric intermediates, which is reflected on the yields achieved on the different synthetic steps, generally lower for the l-series. Particular attention must be paid to the cyclization process: whereas both regio-*endo*-**4** diastereomers could be formed from their acyclic precursor using the standard protocol, the isolated yield for l-Trp-regio-*endo*-**4** is considerably lower than the corresponding yield for d-Trp-regio-*endo*-**4** diastereomer. Likewise, while d-Trp-regio-*exo*-**4** could only be detected by HPLC-MS, but never isolated, an impure sample of diastereomeric l-Trp-regio-*exo*-**4** sufficed to record a ^1^H NMR spectrum and discard this structure as the one matching the natural product. These observations seem to confirm that several factors such as the stability of the final product and/or the conformational and/or configurational features of the acyclic precursors determine the final outcome of the macrolactam formation.^27^

#### Route B.5 toward the Exo and Endo Diastereomers of l-Trp-regio-**56**

**Scheme 11:**
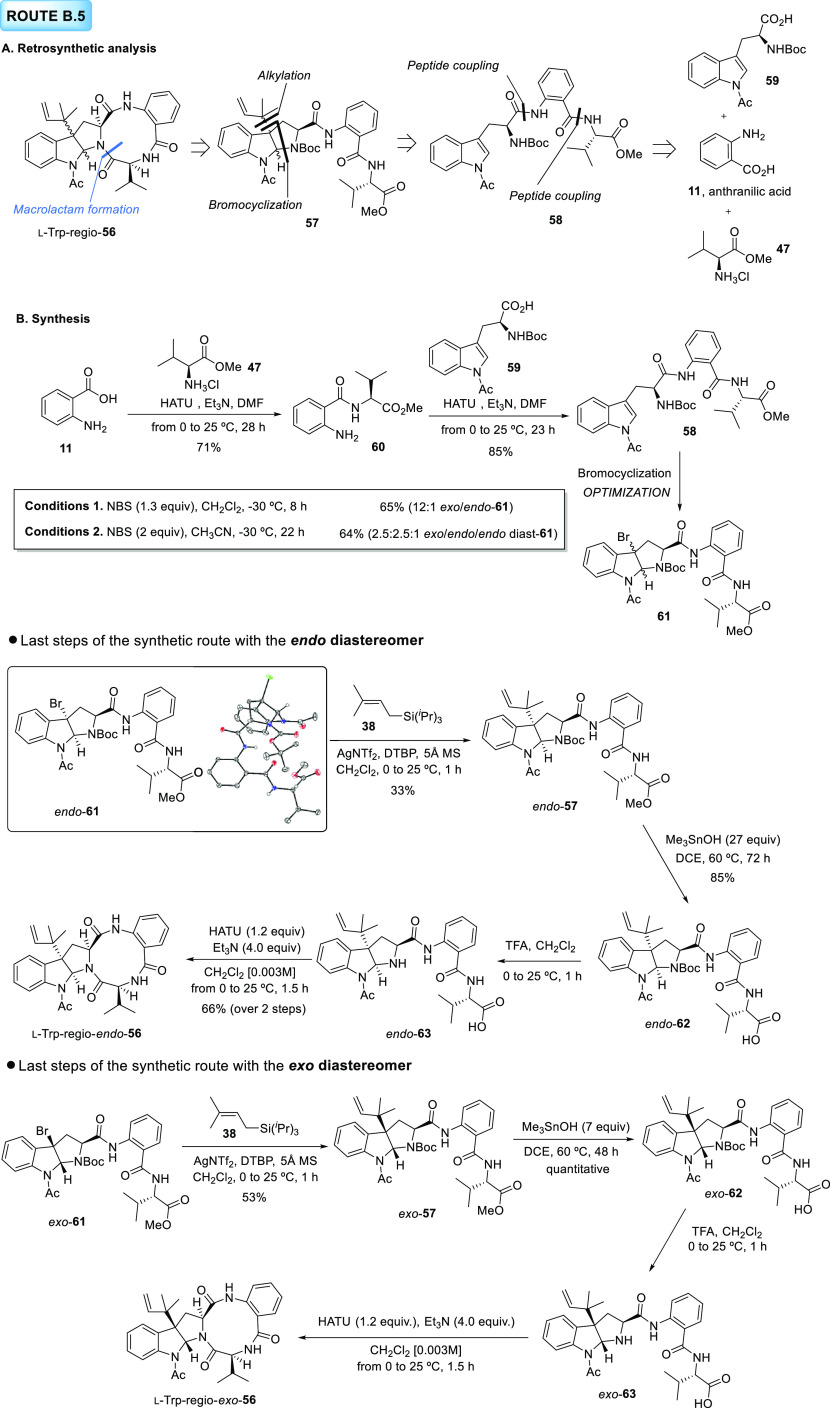
Total Synthesis of *Exo* and *Endo* Isomers of l-Trp-regio-**56** Following Route B.5. (A) Retrosynthetic Analysis. (B) Synthetic Route toward *Exo* and *Endo* Isomers of l-Trp-regio-**56** The ORTEP diagram of the X-ray structure of *endo*-**61** is represented with the ellipsoids drawn at 50% probability level.

Continuing with our campaign to identify the correct structure of natural novofumigatamide, the constitutional isomer obtained from the positional exchange of the valine and the anthranilate units was envisioned as an alternative structure (l-Trp-regio-**56**, Scheme 11). Since the position of the acetyl group and the connectivity between the macrolactam ring and the hexahydropyrrolo[2,3-*b*]indole is not altered in this new proposal with respect to the original structure, the route toward l-Trp-Exo-**1** developed in the first part of this contribution (DOI: 10.1021/acs.joc.2c01127) was selected as the most appropriate synthetic approach (Scheme 11A).^2^ In the retrosynthetic analysis for this route, named B.5, the final ring-closing macrolactam formation to complete the construction of the synthetic product would occur through the formation of the amide bond between the valine and the tryptophan unit from prenylated pyrroloindole precursor **57**. In turn, this precursor could be traced back, after sequential bromocyclization-alkylation reactions, to linear intermediate **58**. The latter would be synthesized by condensation of anthranilic acid (**11**) and the appropriate valine (**47**) and tryptophan (**59**) derivatives.

In Part I of this manuscript, we rationalized the stereochemical origin of novofumigatamide based on the relative and absolute configurations of additional metabolites isolated from the same fungus genre, *Aspergillus novofumigatus*.^2^ According to the available data,^30,31^ this fungus would produce secondary metabolites containing l-tryptophan amino acids. The coincidence in the sign of the optical rotation of novofumigatamide and of all of the final products hitherto synthesized from l-tryptophan confirmed this fact. Thus, only *exo*- and *endo*-**56** final products arising from l-tryptophan were approached.

Our last synthetic effort began with the preparation of dipeptide **60** by HATU-mediated condensation of anthranilic acid **11** and valine methyl ester hydrochloride **47**.^32^ A second condensation between this dipeptide and l-tryptophan derivative **59** (prepared in Part I; DOI: 10.1021/acs.joc.2c01127),^2^ following the same protocol, furnished acyclic precursor **58**. As in previous routes, the following diastereoselective bromocyclization was subjected to a screening of reaction conditions (see the SI for further information), but unfortunately none of the conditions tested furnished the *endo* bromopyrroloindole *endo*-**61** as the major isomer. Using NBS in CH_2_Cl_2_ at −30 °C, the *exo* diastereomer was isolated as the major product in a very good (12:1) *exo*/*endo* ratio. On the other hand, when the solvent was replaced by CH_3_CN, a mixture of *exo*/*endo*/*endo* epimeric products was obtained. Moreover, as reported previously,^2^ when this solvent was used, 2 equiv of NBS and longer reaction times were required to complete the conversion to the product. Remarkably, yields for the bromocyclization of **58** were lower than those obtained using previously described intermediates. To our delight, an X-ray crystal structure of *endo*-**61** confirmed the identity of this diastereomer. After separation of both bromohexahydropyrrolo[2,3-*b*]indole isomers **61**, the route continued independently with each of them. Reverse prenylation with our more general protocol led to the corresponding alkylated products **57** in yields unexpectedly higher than those obtained with other regioisomeric intermediates previously synthesized in this project. This fact was particularly noteworthy for *exo*-**57**, which was isolated in a satisfactory 53% yield. The behavior of *exo*- and *endo*-**57** toward the subsequent hydrolysis was unexpected since the longer distance between the ester functionality and the *endo* or *exo*-pyrroloindoline core would anticipate a straightforward saponification. Although *exo*-**57** reacted under standard conditions (7 equiv of Me_3_SnOH and 60 °C), *endo*-**57** required 4-fold equivalents of Me_3_SnOH employed with the *exo* isomer, longer reaction times, and intermediate workups to remove byproducts and excess of nonreacting reagent. Very likely, a puckering of this structure leading to a less accessible reaction site would be responsible for this unanticipated result. With the free carboxylic acids *exo*- and *endo*-**62** in hand, the TFA-based *N*-Boc removal was the next step addressed. Surprisingly, an epimerization occurred during the unveiling of the amine group on *exo*-**62** (see the HPLC chromatograms in the SI), a side reaction never observed before during this deprotection reaction. Final macrolactam formation was attained with this mixture of epimers using the conditions developed in Part I to cyclize similar regioisomers (DOI: 10.1021/acs.joc.2c01127).^2^ Nevertheless, an almost 1:1 mixture of epimers of the final product l-Trp-regio-*exo*-**56** was observed by HPLC-MS. Unfortunately, the two epimers decomposed during the workup or upon contact with the CDCl_3_ employed as a deuterated solvent for the acquisition of the ^1^H NMR data. While *N*-Boc unveiling in *endo*-**62** did not occur with a concomitant epimerization of the product, during the subsequent macrolactam formation, small amounts of a new epimer were observed. Although these epimers could not be separated by column chromatography, the final product l-Trp-regio-*endo*-**56** proved to be more stable than the *exo* counterpart, and consequently, ^1^H NMR spectra in CDCl_3_ could be recorded. Unluckily, the signals displayed in the spectrum of this new synthetic structure did not match those of the natural product. Test experiments demonstrated the fast decomposition rate of this new skeleton, which precluded its full characterization. This decomposition process occurred readily in a short period of time by handling the compound (dissolving and concentrating the sample several times), or simply upon storage of the sample at −30 °C for several days. Therefore, the instability of these new isomers and their tendency to epimerize are strong evidences to discard these structures as the natural product.

## Conclusions

In summary, we have reported the total synthesis of several constitutional isomers of the structure originally proposed for novofumigatamide. The new synthetic products arise either from the positional exchange of the valine and the anthranilate units or from a different connectivity of the macrolactam ring with the hexahydropyrrolo[2,3-*b*]indole core. In addition, all of them fulfill most of the 2D NMR and ROESY correlations reported for the natural alkaloid. Up to six different synthetic routes were explored to approach the *exo* and *endo* diastereomers of the final products. The first group of constitutional isomers prepared in this work display a 12-membered ring macrolactam connected with the pyrroloindoline framework through the indole nitrogen, whereas the acetyl group is placed at the pyrrole nitrogen. A route based on the formation of the macrolactam through the condensation between the tryptophan and valine edges, route B.3, could be successfully developed and gave rise to *endo* final products arising from l- or d-tryptophan and l-valine amino acids (d-Trp-regio-*endo*-**4** and l-Trp-regio-*endo*-**4**). The corresponding *exo* products are not stable structures, whereas a ^1^H NMR spectrum of a moderately pure sample of l-Trp-regio-*exo*-**4** could be recorded, and d-Trp-regio-*exo*-**4** was only detected in trace amounts in the reaction mixtures. In addition, a new compound arising from two consecutive condensation reactions within the molecule (*exo*-**41**) was also identified along the formation of the last-mentioned *exo* diastereomer. The second group of constitutional isomers described in this manuscript derive from the positional exchange between the valine and the anthranilate residues within the macrolactam ring. Using a synthetic route developed in Part I (DOI: 10.1021/acs.joc.2c01127),^2^l-Trp-regio-*exo*-**56** and l-Trp-regio-*endo*-**56** could be accessed. However, none of these final products were stable enough to accomplish a full characterization. A ^1^H NMR spectrum of a moderately pure sample of l-Trp-regio-*endo*-**56** led to discarding this structure for the natural product.

**Figure 4:**
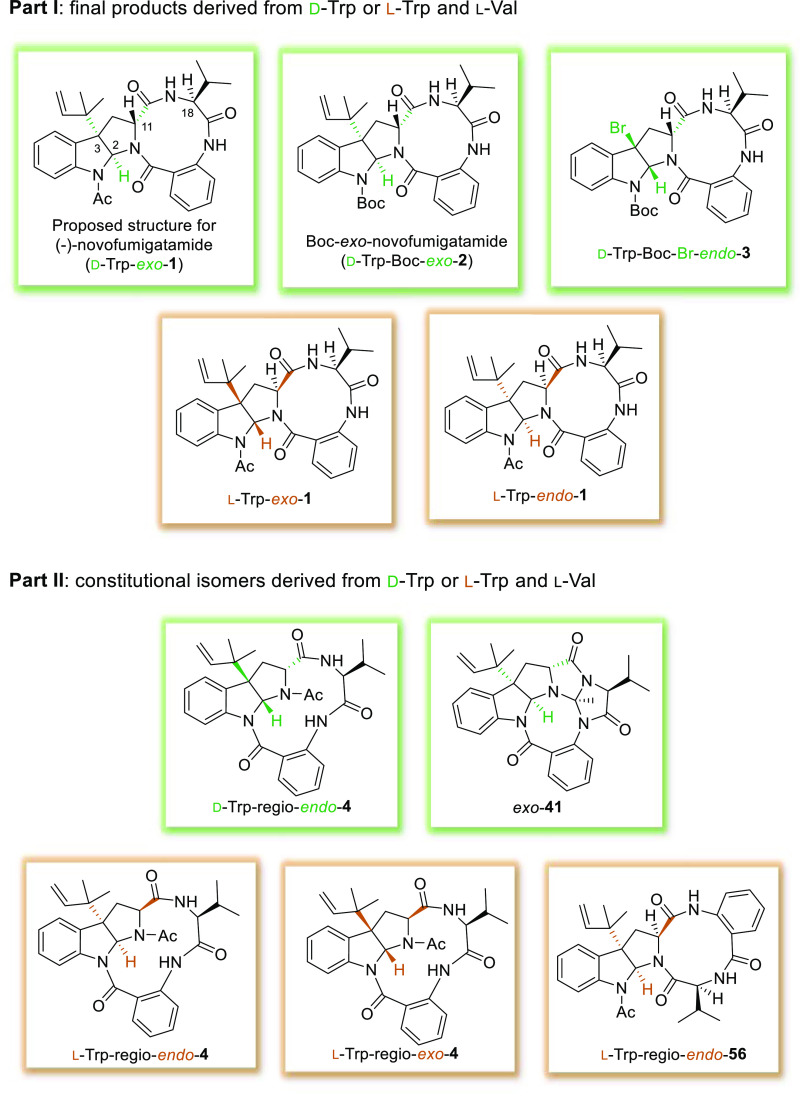
Final products synthesized throughout the synthetic project (Part I^2^ and Part II).

In all of the routes studied, the high dependence of several transformations (bromocyclization, hydrolysis of methyl esters, reverse prenylation, and macrolactam formation reactions) on the structure and the relative configuration of the intermediates and final products was demonstrated, which made the prediction of the outcome of these processes a challenging task. X-ray diffraction analysis of advanced intermediates and final products proved to be a very helpful tool to unambiguously confirm the identity of these compounds. Unfortunately, none of the spectroscopic data of the final products prepared herein were consistent with those reported for natural (−)-novofumigatamide. Furthermore, the instability shown by some of these final products makes them unsuitable as candidates for the naturally occurring compound. A comparison between all of the NMR data collected during the development of this synthetic project, the data reported in the literature for similar compounds, and the spectroscopic data of natural novofumigatamide led us to conclude that the correct structure of the natural product is a rigid *endo*-structure derived from l-tryptophan. Alternatively, a flexible skeleton with a preferred conformation fixed by means of strong intramolecular hydrogen bonding interactions could be proposed.^33^Figure 4 shows an overview of all of the final structures prepared in the course of this research project (Part I^2^ and Part II).

Synthetic organic chemists have often corrected some of the reported structures of natural products including their core skeletons, functional group location, and the relative and absolute configurations.^34−36^ However, the disagreement between the spectra of the natural and the synthetic compounds makes the effort rather frustrating, in particular when despite our efforts the structure remains undetermined, as recently recognized by Fürstner *et al*. on natural product chagonensine^37^ using the words of Winston Churchill referring to the Soviet Union in October 1939 as “A riddle wrapped in a mystery inside an enigma”, which could also be applied nowadays. Fortunately, efforts of natural product chemists toward achieving a more accurate structural elucidation^38^ and also the Raw Data Initiative^39^ are suggesting new avenues to extract all of the valuable information contained in the experimental data set and carry out rigorous structural determination, finally reducing uncertainties and discouraging additional work of synthetic/medicinal chemists.

## Supporting Information Available

Synthesis and characterization of substrates, optimization of reaction conditions, comparative tables of the spectroscopic data of the natural and the synthetic products, spectra collection and HPLC-MS traces of the compounds synthesized, X-ray crystallographic data, and references (PDF)
