# Supplementary material for: Puzzling Out the Structure of Novofumigatamide: Total Synthesis of Constitutional Isomers. Part II
Source: J Org Chem. 2022 Sep 21;87(19):12528–46. doi: 10.1021/acs.joc.2c01228 (PMC9552235; doi:10.1021/acs.joc.2c01228)
Supplement: Supplementary file 1 — jo2c01228_si_001.pdf [file jo2c01228_si_001.pdf]

Supporting Information

Puzzling Out the Structure of Novofumigatamide: Total Synthesis of Constitutional Isomers. Part II.

Patricia García-Domínguez,* and Angel R. de Lera*

CINBIO, Universidade de Vigo, 36310 Vigo, Spain

Table of contents

1. General information S1

2. Synthesis and characterization of substrates. Optimization of reaction conditions S3

3. Comparative tables of the spectroscopic data of the natural and the synthetic products S42

4. Spectra collection and HPLC-MS traces.. S43

5. X-Ray structures.. S109

6. References S117

1. General information

$CHCl_3$ and EtOH were used from commercial sources as analytical reagent-grade solvents ("wet" solvents) or dried according to published methods and distilled before use if required. THF, CH_2Cl_2, DMF, CH_3CN and MeOH were dried using a solvent purification system (Puresolv^{TM}, Innovative Technology). Alternatively, Aldrich MeOH, DMF and CH_3CN 99.8% packaged under argon in a resealable Sure/Seal^{TM} bottle were used. DCE (1,2-dichloroethane) 99.5%, extra dry, kept over Molecular Sieves and packaged under argon in a resealable AcroSeal® was used. 1,4-Dioxane 99.5%, extra dry, kept over Molecular Sieves and packaged under nitrogen in a resealable AcroSeal® or Sigma-Aldrich 1,4-Dioxane anhydrous 99.8% in Sure/Seal™, were used. Et_3N and DIPEA were dried by distillation over CaH_2. Alternatively, Et_3N ($\geq$ 99.5%) and DIPEA (99.5%) packaged under argon in a resealable Sure/Seal^{TM} bottle were purchased from Aldrich. Et_2NH was dried by distillation with CaH_2. All other reagents were commercial compounds of the highest purity available, which were used as received unless otherwise noted. All the reactions were carried out under argon, and those not involving aqueous reagents were carried out in oven-dried glassware. All solvents and anhydrous solutions were transferred through syringes and cannulae previously dried in an oven for at least 12 h and stored in a desiccator with KOH. For reactions at low temperatures, ice/water or CO_2/acetone systems were used. For different temperatures, a HaaKe EK90 Immersion Cooler apparatus (-78 to 0 °C) was used. Analytical TLC was performed on aluminium plates with Merck Kieselgel $60F_{254}$ and visualized by UV irradiation (254 nm) or by staining with an ethanolic solution of phosphomolybdic acid or an ethanolic solution of *p*-anisaldehyde. Flash-column chromatography was carried out on Merck Kieselgel 60 (230–400 mesh), Silicycle SiliaFlash® P60 (230-400 mesh) or C18-SiO_2 (Waters, 55–105 µm, 125Å) under pressure or using an automated Teledyne Isco CombiFlash® Rf+ system. HPLC was performed using a Waters 515 Pump, a Waters 600 Controller, and a Waters 2487 Dual λ Absorbance Detector. HPLC-MS system contains the following modules: Waters 2545 pump, Waters 2767 Sample Manager, Waters SFO System Fluidics Organizer, Waters 2898 Photodiode Array Detector, MICROMASS Quattro micro^{TM} API mass spectrometer and Edwards 28 Vacuum Pump.

^{1}H NMR and ^{13}C{^{1}H} NMR spectra were recorded at room temperature or at the indicated temperature (343 K, 353 K, 363 K) on a Bruker AVANCE 400 spectrometer operating at 400.16 and 100.62 MHz, respectively, with residual protic solvent as the internal reference ($CDCl_3$, δ_H = 7.26 and δ_C= 77.2 ppm; DMSO-d_6, δ_H = 2.50 and δ_C = 39.52 ppm; CD_3OD, δ_H = 3.31 ppm); chemical shifts (δ) are given in parts per million (ppm) and coupling constants (*J*) are given in Hertz (Hz). The proton spectra are reported as follows: δ (multiplicity, coupling constant *J*, number of protons, assignment). The DEPT135 pulse sequence and the standard 2D experiments (HSQC, HMBC, COSY) were used to aid in the assignment of signals in the ^{1}H

NMR spectra. HRMS (ESI-TOF) were measured with a Bruker SolariX XR mass spectrometer equipped with a 7T actively shielded magnet. Ions were generated using an Apollo API electrospray ionization (ESI) source, with a voltage between 1800 and 2200 V (to optimize ionization efficiency) applied to the needle, and a counter voltage of 450 V applied to the capillary. ESI spectra samples were prepared by adding a spray solution of 70:29.9:0.1 (*v/v/v*) MeOH/water/formic acid to a solution of the sample at a *v/v* ratio of 1 to 5% to give the best signal-to-noise ratio. IR spectra were recorded with a JASCO FT/IR-4200 spectrophotometer from a thin film deposited onto NaCl glass or with an ATR module. Specific optical rotations were measured on a JASCO P-1020 or a JASCO P-2000 polarimeters with a Na lamp (glass cell, 3.5 x 100 mm). Melting points were measured in a Stuart Scientific apparatus.

2. Synthesis and characterization of substrates. Optimization of reaction conditions

Methyl (*R*)-2-Amino-3-(indole-3'-yl)propanoate (*R*)-15

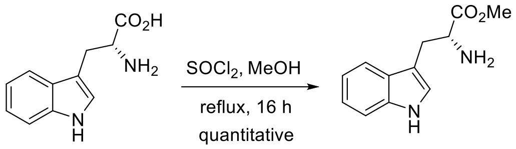

9 (*R*)-15

To a cooled (0 ºC) solution of thionyl chloride (3.4 mL, 5.52 g, 46.0 mmol) in MeOH (40 mL) D-tryptophan **9** was added (4.0 g, 20.0 mmol). A reflux condenser was then fitted to the round-bottomed flask and the reaction mixture was stirred in a metal heating block at 70 ºC for 16 h. The solvent was removed under reduced pressure to obtain the corresponding hydrochloride. The residue was neutralized with a saturated aqueous solution of $NaHCO_3$, and the aqueous layer was extracted with EtOAc (3x). The combined organic layers were dried (anhydrous Na_2SO_4), filtered and the solvent evaporated to give 4.36 g (quantitative yield) of the titled compound as a brown foam.[1]

^{1}H-NMR (400.16 MHz, CD_3OD) δ 7.52 (dt, J = 7.9, 1.1 Hz, 1H, ArH), 7.34 (dt, J = 8.2, 1.0 Hz, 1H, ArH), 7.12 – 7.06 (m, 2H, ArH), 7.01 (ddd, J = 8.0, 7.0, 1.1 Hz, 1H, ArH), 3.76 (dd, J = 6.7, 5.7 Hz, 1H, H_2), 3.64 (s, 3H, $CO_2C\underline{H_3}$), 3.19 (ddd, J = 14.3, 5.7, 0.9 Hz, 1H, H_{3A}), 3.10 (ddd, J = 14.3, 6.7, 0.8 Hz, 1H, H_{3B}) ppm.

Methyl (*R*)-2-Acetylamino-3-(indole-3'-yl)propanoate (*R*)-16

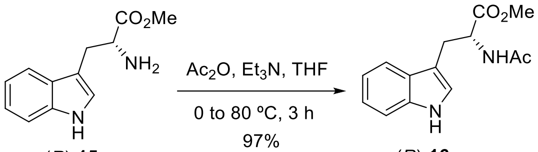

(*R*)-15 (*R*)-16

General procedure for the acetylation of amines. To a solution of the ester (*R*)-**15** (4.36 g, 20.0 mmol) and Et_3N (2.9 mL, 2.1 g, 21.0 mmol) in THF (222 mL) at 0 ºC, acetic anhydride (2.1 mL, 2.25 g, 22.0 mmol) was added and the mixture was stirred in a metal heating block at 80 ºC for 3 h. The reaction mixture was quenched with H_2O, the layers were separated, and the aqueous layer was extracted with EtOAc (3x). The combined organic layers were sequentially washed with a 1M HCl aqueous solution, a saturated aqueous solution of $NaHCO_3$ and a saturated aqueous solution of NaCl. The combined organic layers were dried over anhydrous Na_2SO_4 and concentrated under reduced pressure. The residue was triturated 4 times with Et_2O (1 x 60 mL and 3 x 30 mL) to give 5.03 g (97% yield) of the titled compound as a white solid.[2]

^{1}H NMR (400.16 MHz, $CDCl_3$, 298 K) δ 8.14 (s, 1H, NH), 7.57 – 7.49 (m, 1H, ArH), 7.36 (dt, J = 8.1, 0.9 Hz, 1H, ArH), 7.20 (ddd, J = 8.2, 7.1, 1.2 Hz, 1H, ArH), 7.12 (ddd, J = 8.0, 7.1, 1.1

Hz, 1H, ArH), 6.98 (d, J = 2.4 Hz, 1H, ArH), 5.98 (d, J = 7.5 Hz, 1H, NH), 4.96 (dt, J = 7.9, 5.3 Hz, 1H, H_2), 3.70 (s, 3H, $CO_2C\underline{H_3}$), 3.36 (ddd, J = 14.8, 5.4, 0.7 Hz, 1H, H_{3A}), 3.30 (ddd, J = 14.8, 5.1, 0.7 Hz, 1H, H_{3B}), 1.96 (s, 3H, $COC\underline{H_3}$) ppm.

(*S*)-2-(2'-{[(9*H*-Fluoren-9-yl)methoxycarbonyl]amino}-3'-methylbutanamido)benzoic Acid 17

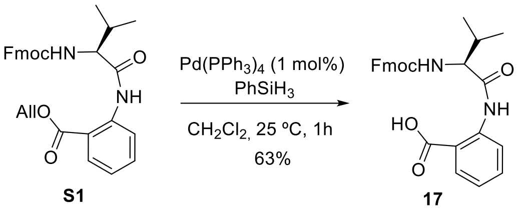

To a stirred solution of protected ester **S1** (0.5 g, 1.0 mmol), prepared in Part I of this manuscript (DOI: 10.1021/acs.joc.2c01127),[3] and $Pd(PPh_3)_4$ (12.0 mg g, 0.01 mmol) in CH_2Cl_2 (16 mL), phenylsilane (0.25 mL, 2.01 mmol) was added dropwise and the resulting mixture was stirred for 1 h at 25 ºC. The reaction mixture was diluted with CH_2Cl_2 (6 mL), the layers were separated, and the organic layer was washed with a 10% aqueous solution of HCl (2x) and a saturated aqueous solution of NaCl (2x), dried over anhydrous Na_2SO_4 and the solvent was evaporated. The residue was purified by flash column chromatography (CombiFlash® Rf+ system, 40 g silica gel, gradient from 70:30 to 20:80 *v/v* hexane/EtOAc, flow rate = 40 mL/min) to afford 0.29 g (63% yield) of the titled compound as a white foam. **^{1}H NMR** (400.16 MHz, DMSO-d_6, 343 K) δ 8.53 (d, J = 8.2 Hz, 1H, ArH), 8.04 (dd, J = 7.8, 1.7 Hz, 1H, ArH), 7.91 – 7.82 (m, 2H, ArH), 7.81 – 7.64 (m, 2H, ArH), 7.50 (br s, 1H, NH), 7.44 – 7.21 (m, 5H, ArH), 7.04 (td, J = 7.8, 1.2 Hz, 1H, ArH), 4.36 – 4.19 (m, 3H, $H_{2'}$ + $CO_2\underline{CH_2}Ph$), 3.92 (dd, J = 8.2, 6.6 Hz, 1H, Ar<u>CH</u>Ar), 2.24 – 2.13 (m, 1H, $C\underline{H}(CH_3)_2$), 0.97 (major rotamer, d, J = 6.8 Hz, 3H, $CH(C\underline{H_3})_2$), 0.96 (major rotamer, d, J = 6.8 Hz, 3H, $CH(C\underline{H_3})_2$) ppm. **^{13}C{^{1}H} NMR** (100.62 MHz, DMSO-d_6, 343 K) δ 170.2 (s), 169.8 (s), 156.2 (s), 143.7 (s), 143.6 (s), 140.5 (s), 140.2 (s), 131.2 (d), 130.9 (d), 127.3 (d), 126.8 (d), 125.1 (d), 121.6 (d), 119.7 (d), 118.7 (d), 65.8 (t), 62.2 (d), 46.6 (d), 29.8 (d), 19.0 (q), 18.0 (q) ppm. **HRMS** (ESI-TOF) *m/z*: $[M + H]^+$ calcd. for $C_{27}H_{27}N_2O_5$, 459.1914; found, 459.1916. **IR** (NaCl): ν 3400-3100 (br, O-H, N-H), 3066 (w, C-H), 3017 (w, C-H), 2965 (m, C-H), 1678 (s, C=O), 1589 (s), 1514 (s), 1449 (s), 1389 (s), 1336 (m), 1298 (m), 1243 (m), 1162 (w), 1106 (w), 1033 (w), 934 (w), 758 (s), 741 (s), 703 (w), 667 (w) cm^{-1}. $[\alpha]_D^{21}$ –163.3 (*c* 0.13, $CHCl_3$).

(*S*)-4-Nitrophenyl [2’-(2’’-{[(9*H*-Fluoren-9-yl)methoxycarbonyl]amino}-3’’-methylbutanamido)]benzoate 20

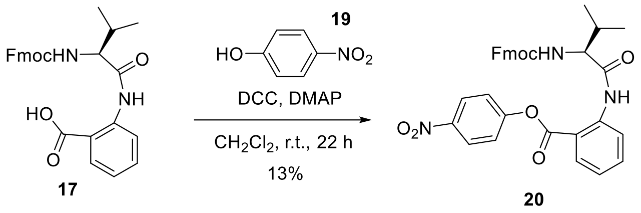

p-Nitrofenol **19** (0.02 g, 0.14 mmol), benzoic acid **17** (0.07 g, 0.16 mmol) and DCC (0.04 g, 0.18 mmol) were stirred in CH_2Cl_2 (1 mL) at 0 ºC for 1 h. DMAP (1.0 mg, 8.2 μmol) was then added and the solution was allowed to warm to room temperature and stirred for 22 h. The solution was then filtered and the solvent evaporated. The residue was purified by flash column chromatography (CombiFlash® Rf+ system, 12 g silica gel, gradient from 95:5 to 90:10 *v/v* hexane/EtOAc, flow rate = 30 mL/min) to afford 11.0 mg (13% yield) of the titled compound.

^{1}H NMR (400.16 MHz, DMSO-*d*$_6$, 343 K)

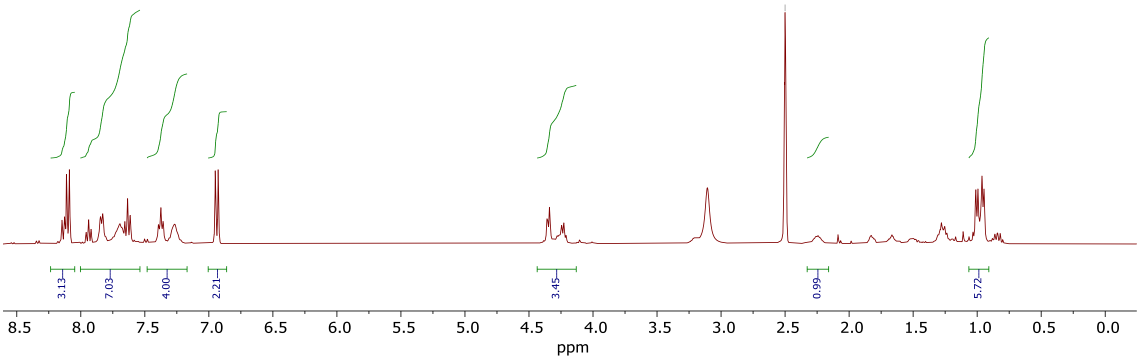

Methyl (*R*)-2-(Acetylamino)-3-[1’-(2”-aminobenzoyl)indole-3’-yl]propanoate (*R*)-23

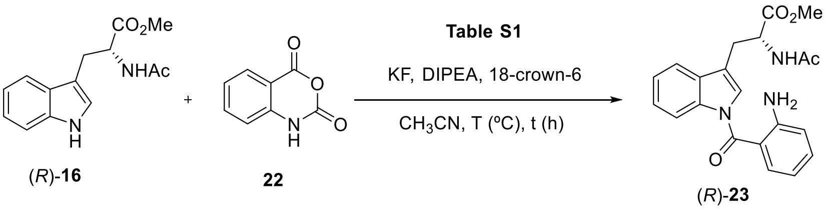

Entry	Equivalents reagents	T ºC	t (h)	Yield (%)
**1**	Isatoic anhydride (2 equiv.), KF (5 equiv.), DIPEA (2 equiv.), 18-crown-6 (5 equiv.)	**60**	24	11
**2**	Isatoic anhydride (2 equiv.), KF (5 equiv.), DIPEA (2 equiv.), 18-crown-6 (5 equiv.)	**90**	24	64
**3**	Isatoic anhydride (**4 equiv.**), KF (5 equiv.), DIPEA (2 equiv.), 18-crown-6 (5 equiv.)	90	24	78
**4**	Isatoic anhydride (4 equiv.), KF (5 equiv.), DIPEA (2 equiv.), 18-crown-6 (5 equiv.)	90	**48**	83
**5**	Isatoic anhydride (4 equiv.), KF (**2.5 equiv.**), DIPEA (2 equiv.), 18-crown-6 (**2.5 equiv.**)	90	24	83

In the glovebox a round-bottomed flask was charged with potassium fluoride (1.12 g, 19.21 mmol) and 18-crown-6 (5.08 g, 19.21 mmol). Returning to the bench, CH_3CN (256 mL), isatoic anhydride **22** (5.01 g, 30.74 mmol), the indole derivative (*R*)-**16** (2.0 g, 7.68 mmol) and DIPEA (2.63 mL, 1.99 g, 15.37 mmol) were loaded into the flask and the reaction mixture was stirred overnight in a metal heating block at 90 ºC for 24 h. The reaction was quenched with water and the mixture extracted with EtOAc (3x). The combined organic layers were washed with a saturated aqueous solution of NaCl, dried over anhydrous Na_2SO_4 and the solvent was evaporated. The residue was purified by flash column chromatography (silica gel, gradient from 60:40 to 50:50 *v/v* hexane/EtOAc) to afford 2.42 g (83% yield) of the titled compound as a beige solid. **M.p.**: 161-163 ºC (hexane/CH_2Cl_2/MeOH). **^{1}H NMR** (400.16 MHz, $CDCl_3$, 298 K) δ 8.26 (d, *J* = 8.1 Hz, 1H, ArH), 7.51 (d, *J* = 6.8 Hz, 1H, ArH), 7.39 – 7.27 (m, 4H, ArH), 7.11 (s, 1H, ArH), 6.78 (d, *J* = 8.6 Hz, 1H, ArH), 6.73 (t, *J* = 7.5 Hz, 1H, ArH), 6.13 (br s, 1H, NH), 4.98 – 4.89 (m, 1H, H_2), 3.68 (s, 3H, $CO_2C\underline{H_3}$), 3.30 (dd, *J* = 14.7, 5.7 Hz, 1H, H_{3A}), 3.18 (dd, *J* = 14.8, 5.0 Hz, 1H, H_{3B}), 1.95 (s, 3H, $COC\underline{H_3}$) ppm. **^{13}C{^{1}H} NMR** (100.62 MHz, $CDCl_3$, 298 K) δ 172.2 (s), 169.8 (s), 169.0 (s), 149.1 (s), 136.2 (s), 133.5 (d), 131.4 (d), 131.1 (s), 126.2 (d), 125.1 (d), 123.8 (d), 118.8 (d), 117.2 (d), 116.7 (d), 116.4 (d), 116.1 (s), 115.8 (s), 52.8 (d), 52.6 (q), 27.4 (t), 23.3 (q) ppm. **HRMS** (ESI-TOF) *m/z*: $[M + H]^+$ calcd. for $C_{21}H_{22}N_3O_4$, 380.1605; found, 380.1601. **IR** (NaCl): ν 3475 (s, NH_2), 3373 (s, NH_2), 3054 (w, C-H), 3011 (w, C-H), 2952 (w, C-H), 1742 (s, C=O), 1667 (s, C=O), 1619 (s, C=O), 1590 (s), 1533 (s), 1491 (s), 1452 (s), 1367 (s), 1329 (s), 1250 (s), 1218 (s), 1043 (m), 885 (m), 752 (s), 665 (m) cm^{-1}. $[\alpha]_D^{18}$ –44.9 (*c* 0.34, $CHCl_3$).

Methyl (*S*)-2-(Acetylamino)-3-[1'-(2"-aminobenzoyl)indole-3'-yl]propanoate (*S*)-23: $[\alpha]_D^{24}$ +45.1 (*c* 0.19, $CHCl_3$).

Methyl (2*R*,2'''*S*)-2-Acetylamino-3-(1'-{2"-[2'''-(*tert*-butoxycarbonyl)amino-3'''-methylbutanamido]benzoyl}indole-3'-yl)propanoate (*R*)-25

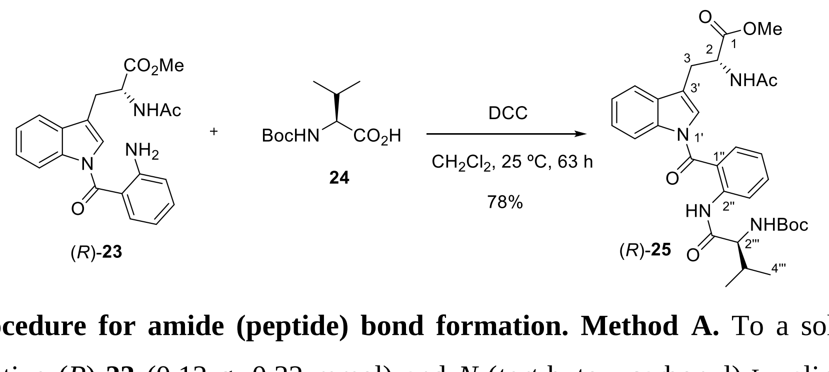

General procedure for amide (peptide) bond formation. Method A. To a solution of the indole derivative (*R*)-**23** (0.12 g, 0.32 mmol) and *N*-(*tert*-butoxycarbonyl)-L-valine **24** (0.1 g, 0.47 mmol) in CH_2Cl_2 (10.5 mL) stirred at room temperature, DCC (0.23 g, 1.11 mmol) was added in three portions over a period of 48 h. After the addition of the last portion of DCC, the

mixture was further stirred for 15 h, filtered through a pad of Celite®, and the solvent was concentrated. The residue was purified by flash column chromatography (CombiFlash® Rf+ system, 24 g silica gel, gradient from 50:50 to 30:70 *v/v* hexane/EtOAc, flow rate = 35 mL/min) to afford 0.14 g (78% yield) of the titled compound as a white solid. **M.p.**: 154-156 ºC (hexane/CH_2Cl_2/MeOH). **^{1}H NMR** (400.16 MHz, DMSO-d_6, 343 K) δ 9.87 (br s, 1H, NH), 8.20 – 8.14 (m, 1H, ArH), 8.08 (d, *J* = 7.8 Hz, 1H, NH), 7.72 – 7.66 (m, 1H, ArH), 7.64 – 7.57 (m, 2H, ArH), 7.45 (dd, *J* = 7.7, 1.5 Hz, 1H, ArH), 7.36 – 7.28 (m, 3H, ArH), 7.16 (s, 1H, ArH), 6.40 (br s, 1H, NH), 4.56 (td, *J* = 8.1, 5.6 Hz, 1H, H_2), 3.91 (t, *J* = 7.4 Hz, 1H, $H_{2'''}$), 3.61 (s, 3H, $CO_2C\underline{H_3}$), 3.17 – 3.05 (m, 1H, H_{3A}), 2.99 (dd, *J* = 14.7, 8.5 Hz, 1H, H_{3B}), 1.99 – 1.88 (m, 1H, $C\underline{H}(CH_3)_2$) 1.79 (s, 3H, $COC\underline{H_3}$), 1.33 (s, 9H, CO_2*t*Bu), 0.82 (d, *J* = 6.8 Hz, 3H, $CH(C\underline{H_3})_2$), 0.77 (d, *J* = 6.8 Hz, 3H, $CH(C\underline{H_3})_2$) ppm. **^{13}C{^{1}H} NMR** (100.16 MHz, DMSO-d_6, 343 K) δ 171.6 (s), 170.3 (s), 168.9 (s), 166.0 (s), 155.0 (s), 135.5 (s), 135.4 (s), 131.2 (d), 130.3 (s), 128.8 (d), 127.2 (s), 125.7 (d), 124.1 (d), 123.9 (d), 123.6 (d), 123.0 (d), 118.4 (d), 116.2 (s), 115.6 (d), 77.9 (s, $\underline{C}(CH_3)_3$), 60.0 (d), 51.6 (d), 51.4 (q, $CO_2\underline{C}H_3$), 29.9 (d, $\underline{C}H(CH_3)_2$), 27.8 (q, 3x, $C(\underline{C}H_3)_3$), 26.2 (t), 21.8 (q, $CO\underline{C}H_3$), 18.6 (q, $CH(\underline{C}H_3)_2$), 17.4 (q, $CH(\underline{C}H_3)_2$) ppm. **HRMS** (ESI-TOF) *m/z*: $[M + H]^+$ calcd. for $C_{31}H_{39}N_4O_7$, 579.2813; found, 579.2805. **IR** (NaCl): ν 3400-3100 (br, N-H), 3052 (w, C-H), 3004 (w, C-H), 2970 (m, C-H), 2932 (w, C-H), 2875 (w, C-H), 1743 (m, C=O), 1687 (s, C=O), 1674 (s, C=O), 1602 (m), 1582 (m), 1520 (s), 1452 (s), 1368 (s), 1331 (m), 1297 (m), 1253 (m), 1220 (m), 1169 (m), 1041 (m), 1017 (m), 986 (m), 879 (m), 753 (s), 665 (m) cm^{-1}. $[\alpha]_D^{19}$ –34.2 (*c* 0.15, $CHCl_3$).

A proof of concept for the existence of different stable conformers (both for intermediates and final products) fixed by means of strong intramolecular hydrogen bonding interactions was obtained after handling intermediate (*R*)-**25** under different conditions. Upon exposure of this intermediate to different solvent systems (organic and aqueous), temperatures and vacuum conditions, two different rotamers were serendipitously observed in the ^{1}H NMR spectrum.

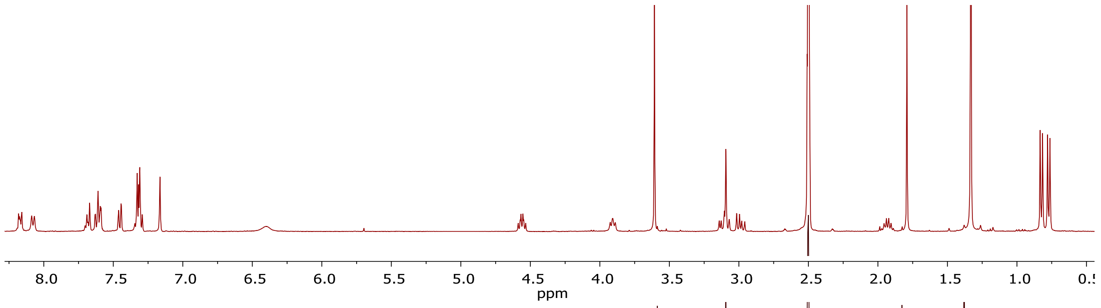

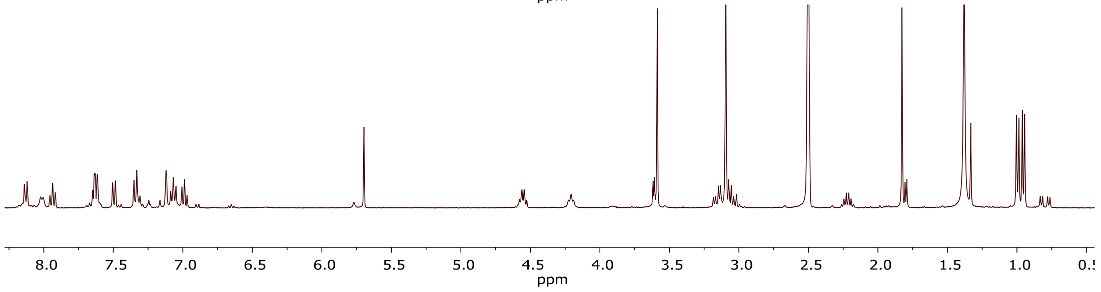

Figure S1. Rotamers observed in the ^{1}H NMR spectrum of intermediate (*R*)-**25** upon exposure to different conditions.

(2*R*,2'''*S*)-2-Acetylamino-3-(1'-{2"-[2'''-(*tert*-butoxycarbonyl)amino-3'''-methylbutanamido]benzoyl}indole-3'-yl)propanoic Acid (*R*)-27

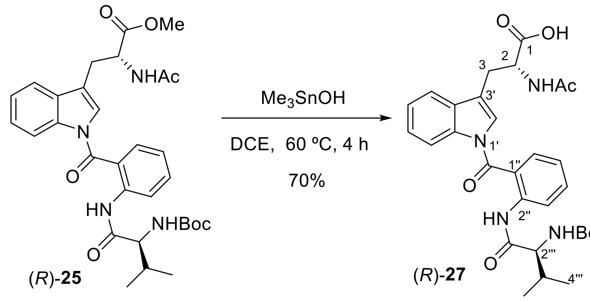

General procedure for the hydrolysis of methyl esters. Methyl ester (*R*)-**25** (0.12 g, 0.21 mmol) was dissolved in DCE (2.1 mL) and, after addition of trimethyltin hydroxide (0.19 g, 1.03 mmol), the mixture was stirred in a metal heating block at 60 ºC for 4 h. After completion of the reaction, the mixture was concentrated *in vacuo*, and the residue was taken up in ethyl acetate. The organic layer was washed with a 5% aqueous solution of HCl (3x), and a saturated aqueous solution of NaCl, dried over anhydrous Na_2SO_4 and the solvent was removed *in vacuo*. The residue was purified by flash column chromatography (silica gel, 90:10 *v/v* CH_2Cl_2/MeOH) to afford 82.0 mg (70% yield) of the titled compound as a very hygroscopic white solid. **^{1}H NMR** (400.16 MHz, DMSO-d_6, 343 K) δ 9.87 (br s, 1H, NH), 8.20 – 8.13 (m, 1H, ArH), 7.89 (d, J = 7.8 Hz, 1H, NH), 7.79 – 7.67 (m, 1H, ArH), 7.66 – 7.58 (m, 2H, ArH), 7.46 (dd, J = 7.7, 1.6 Hz, 1H, ArH), 7.36 – 7.24 (m, 3H, ArH), 7.17 (s, 1H, ArH), 6.42 (br s, 1H, NH), 4.56 – 4.43 (m, 1H, H_2), 3.96 – 3.88 (m, 1H, $H_{2'''}$), 3.14 (dd, J = 14.8, 4.9 Hz, 1H, H_{3A}), 2.96 (dd, J = 14.8, 8.5 Hz, 1H, H_{3B}), 2.00 – 1.88 (m, 1H, C$\underline{H}$(CH_3)$_2$), 1.78 (s, 3H, COC$\underline{H_3}$), 1.33 (s, 9H, CO_2*t*Bu), 0.83 (d, J = 6.8 Hz, 3H, CH(C$\underline{H_3}$)$_2$), 0.77 (d, J = 6.8 Hz, 3H, CH(C$\underline{H_3}$)$_2$) ppm. **^{13}C{^{1}H} NMR** (100.62 MHz, DMSO-d_6, 343 K) δ 172.5 (s), 170.2 (s), 168.7 (s), 166.0 (s), 155.0 (s), 135.6 (s), 135.4 (s), 131.2 (d), 130.5 (s), 128.8 (d), 127.1 (s), 125.5 (d), 124.0 (d), 123.9 (d), 123.6 (d), 123.0 (d), 118.5 (d), 116.8 (s), 115.5 (d), 77.9 (s, $\underline{C}$(CH_3)$_3$), 60.0 (d), 51.7 (d), 29.9 (d, $\underline{C}$H(CH_3)$_2$), 27.8 (q, 3x, C($\underline{C}H_3$)$_3$), 26.4 (t), 22.0 (q, CO$\underline{C}H_3$), 18.6 (q, CH($\underline{C}H_3$)$_2$), 17.4 (q, CH($\underline{C}H_3$)$_2$) ppm. **HRMS** (ESI-TOF) *m/z*: [M + H]$^+$ calcd. for $C_{30}H_{36}N_4O_7$, 565.2657; found, 565.2649. **IR** (NaCl): ν 3400-3200 (br, O-H, N-H), 3009 (m, C-H), 2967 (m, C-H), 2930 (m, C-H), 1687 (s, C=O), 1665 (s, C=O), 1604 (m), 1583 (m), 1523 (s), 1453 (s), 1368 (s), 1332 (m), 1255 (m), 1218 (m), 1165 (m), 1042 (w), 1019 (w), 880 (w), 755 (s) cm^{-1}. $[\alpha]_D^{19}$ –84.2 (*c* 0.17, $CHCl_3$).

Methyl (2*R*,2’’’*S*)-2-Acetylamino-3-(1’-{2”-[2’’’-(9*H*-Fluoren-9-yl)methoxycarbonylamino -3’’’-methylbutanamido]benzoyl}indole-3’-yl)propanoate (*R*)-18

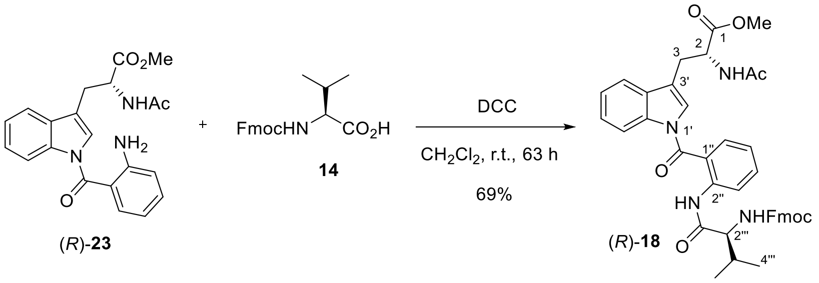

Following the general procedure described above for the amide (peptide) bond formation (**Method A**), the reaction of indole derivative (*R*)**-23** (0.15 g, 0.4 mmol), *N*-Fmoc-L-valine **14** (0.2 g, 0.59 mmol) and DCC (0.12 g, 0.59 mmol) in CH_2Cl_2 (13 mL) at room temperature for 63 h afforded, after purification by flash column chromatography (CombiFlash® Rf+ system, 40 g silica gel, gradient from 50:50 to 40:60 *v/v* hexane/EtOAc, flow rate = 40 mL/min)), 0.19 g (69% yield) of the titled compound as a white solid.

^{1}H NMR (400.16 MHz, DMSO-*d*$_6$, 343 K)

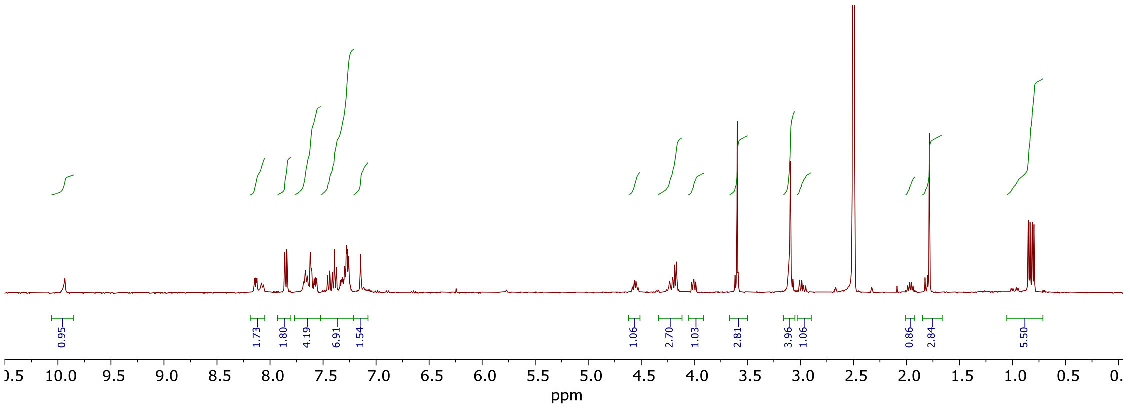

(2*R*,2’’’*S*)-2-Acetylamino-3-(1’-{2”-[2’’’-(9*H*-Fluoren-9-yl)methoxycarbonylamino -3’’’-methylbutanamido]benzoyl}indole-3’-yl)propanoic Acid (*R*)-26

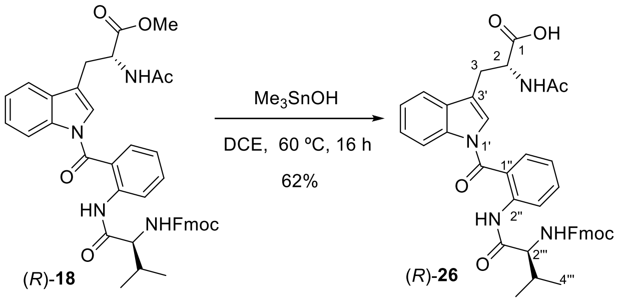

Following the general procedure described above for the hydrolysis of methyl esters, the reaction of ester (*R*)**-18** (0.02 g, 0.03 mmol) with trimethyltin hydroxide (24.0 g, 0.13 mmol) in DCE (0.3 mL) at 60 ºC (metal heating block) for 16 h afforded, after purification by flash column chromatography (silica gel, 90:10 *v/v* CH_2Cl_2/MeOH), 11.0 mg (62% yield) of the titled

compound as a white solid. **^{1}H NMR** (400.16 MHz, DMSO-d_6, 343 K) δ 9.92 (br s, 1H, NH), 8.14 – 8.09 (m, 1H, ArH), 7.85 (d, J = 7.3 Hz, 2H, ArH), 7.71 – 7.55 (m, 5H, ArH), 7.49 – 7.43 (m, 1H, ArH), 7.43 – 7.35 (m, 2H, ArH), 7.34 – 7.21 (m, 5H, ArH), 7.13 (s, 1H, ArH), 4.52 – 4.42 (m, 1H, H_2), 4.28 – 4.12 (m, 3H, OC<u>H</u>$_2$C<u>H</u>Ar$_2$), 4.01 (dd, J = 8.7, 6.5 Hz, 1H, $H_{2'''}$), 3.20-3.05 (m, 1H, H_{3A}), 2.94 (dd, J = 14.8, 8.1 Hz, 1H, H_{3B}), 2.02 – 1.90 (m, 1H, C<u>H</u>(CH$_3$)$_2$), 1.77 (s, 3H, (s, 3H, COC<u>H$_3$</u>), 0.84 (d, J = 6.8 Hz, 3H, CH(C<u>H$_3$</u>)$_2$), 0.80 (d, J = 6.8 Hz, 3H, CH(C<u>H$_3$</u>)$_2$) ppm. **^{13}C{^{1}H} NMR** (100.62 MHz, DMSO-d_6, 343 K) δ 173.7 (s), 170.1 (s), 168.5 (s), 166.0 (s), 155.9 (s), 143.7 (s), 143.5 (s), 140.5 (s), 135.4 (s), 131.2 (d), 130.9 (s), 128.9 (d), 128.6 (d, rotamer), 127.9 (s), 127.3 (d, 2x), 127.0 (d), 126.8 (d), 126.7 (d, rotamer), 125.4 (d), 125.1 (d), 125.0 (d), 124.3 (d), 124.1 (d), 123.9 (d, rotamer), 122.9 (d), 121.1 (d), 119.7 (d), 118.8 (d), 117.5 (s), 115.5 (d), 65.6 (t), 60.3 (d), 52.7 (d), 46.5 (d), 30.1 (d), 26.9 (t), 22.3 (q), 18.8 (q), 17.7 (q) ppm. **HRMS** (ESI-TOF) *m/z*: $[M + H]^+$ calcd. for $C_{40}H_{39}N_4O_7$, 687.2813; found, 687.2815. **IR** (NaCl): ν 3500-3100 (br, O-H, N-H), 3066 (m, C-H), 3015 (m, C-H), 2964 (m, C-H), 2932 (m, C-H), 1693 (s, C=O), 1682 (s, C=O), 1667 (s, C=O), 1660 (s, C=O), 1519 (s), 1452 (s), 1369 (s), 1332 (s), 1255 (s), 1219 (s), 756 (s) cm^{-1}. $[\alpha]_D^{22}$ –35.2 (c 0.18, $CHCl_3$).

Macrolactam 8

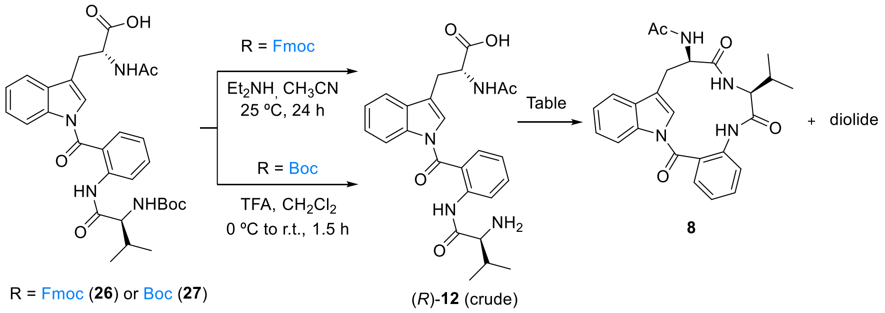

Entry	Reaction conditions	Yield (over two steps)*
	**With R = Boc, (*R*)-27**	
**1**	HATU/HOAt (1:1, 3 equiv. each)<br>DIPEA (147 equiv.), CH_2Cl_2/DMF (3:1), 25 ºC, 6 d<br>Addition of the activated substrate solution to a solution of the base at a flow rate of 0.4 mL/min with the syringe pump. 6 days of additional stirring	Mixture of compounds, decomposition.
**2**	HATU (3 equiv.), 6-Cl-HOBt (2.5 equiv.), DIPEA (321 equiv.), CH_2Cl_2/DMF (3:1) [0.0005M], 25 ºC, 6 days.<br>Addition of the activated substrate solution to a solution of the base over 4 days at a flow rate of 0.3 mL/h with the syringe pump. 2 days of additional stirring	~29% (product) + 6% (diolide)<br>Yields (over 2 steps) are not accurate since the fractions of the column are impure and difficult to analyze by ^{1}H-NMR. An epimerization occurred over the reaction course (two minor peaks with the mass of the product and the diolide observed by HPLC-MS)
**3**	HATU (3 equiv.), 6-Cl-HOBt (2.5 equiv.), DIPEA **(50 equiv.)**, CH_2Cl_2/DMF **(8:1)** [0.0006M], 25 ºC, 1 day<br>Addition of the activated substrate solution to a solution	~38% (over two steps). Not accurate yield. The diolide was also formed (observed by HPLC-MS), but it was not isolated. An

	of the base over 4 h at a flow rate of 0.6 mL/h with the syringe pump and 20 h of additional stirring	epimerization also occurred.
**4**	HATU **(2 equiv.)**, 6-Cl-HOBt **(2 equiv.),** DIPEA **(4 equiv.)**, CH_2Cl_2/DMF **(8:1)** [0.0006M], 25 ºC, 17 h<br>1 h of addition of the activated substrate solution to a solution of the base with a pressure-equilizing dropping funnel and 16 h of additional stirring	~48% (over two steps). Not accurate yield. The diolide was also formed (observed by HPLC-MS), but it was not isolated. An epimerization also occurred.
**5**	HATU (1.2 equiv.), Et_3N (1.2 equiv.), CH_2Cl_2/DMF (3:1), 0 ºC, 45 min **(standard method)**<br>No slow addition	Formation of the diolide as the major product (observed by HPLC-MS). An epimerization also occurred.
**6**	HATU (1.2 equiv.), Et_3N (1.2 equiv.), CH_2Cl_2/DMF (40:1) [0.0006M], from 0 ºC to rt, 3.5 days<br>Addition of the activated substrate solution to a solution of the base over 12 h at a flow rate of 0.83 mL/h with the syringe pump and 3 days of additional stirring	14% (product + epimer) + 18% (diolide + epimer) (over two steps)
**With R = Fmoc, (*R*)-26**		
**7**	HATU, Et_3N (1.2 equiv.), CH_2Cl_2/DMF (3:1)<br>25 ºC, 67 h	Mixture of compounds, decomposition.
**8**	HATU, Et_3N, CH_2Cl_2<br>25 ºC, 16 h<br>The deprotection of the Fmoc group was skipped. The cyclization was directly attempted with substrate (*R*)-**26.** The experiment was performed with 3 or 10 equiv. of Et_3N	Decomposition

* Injection of aliquots of the reactions, the crudes and/or the fractions of the columns in HPLC-MS: SunFire™ analytic C18 column, 5 µm, 250 x 4.6 mm, gradient from 5% to 100% CH_3CN/H_2O in 20 min or from 30% to 100% CH_3CN/H_2O in 20 min, flow rate = 1 mL/min.

Methyl (*R*)-3-[1'-(*tert*-Butoxycarbonyl)indole-3'-yl]-2-(acetylamino)propanoate (*R*)-30

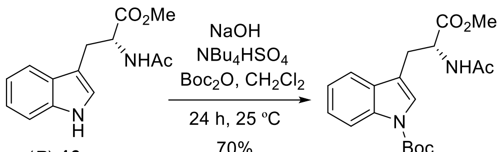

(*R*)-**16** (*R*)-**30**

Powdered sodium hydroxide (1.04 g, 25.93 mmol) was added to a solution of D-tryptophan derivative (*R*)-**16** (1.5 g, 5.76 mmol) and tetrabutylammonium hydrogen sulfate (0.2 g, 0.58 mmol) in CH_2Cl_2 (58 mL). The mixture was stirred for 30 min at 25 ºC. Di-*tert*-butyldicarbonate (2.52 g, 11.53 mmol) was then added and the mixture was stirred for 24 h. It was filtered through a pad of Celite® and the filtrate was evaporated *in vacuo*. The residue was purified by flash column chromatography (CombiFlash® Rf+ system, 80 g silica gel, 95:5 *v/v* hexane/EtOAc/MeOH, flow rate = 60 mL/min) to afford 1.45 g (70% yield) of the titled compound as a colourless foam. The spectroscopic data matched those previously reported.[4]

(2*R*,3a*S*,8a*S*)-1-Acetyl-3a-bromo-(1,2,3,3a,8,8a)-hexahydropyrrolo[2,3-*b*]indole-2,8-dicarboxylic Acid 8-*tert*-Butyl Ester 2-Methyl Ester ((*R*)-*exo*-31)

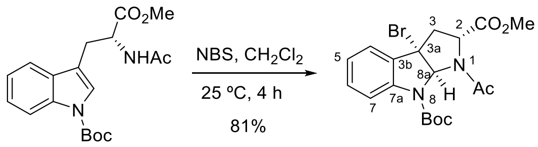

(*R*)-**30** (*R*)-*exo*-**31**

General procedure for the bromocyclization with *N*-bromosuccinimide (Method A). To a stirred solution of the of the tryptophan derivative (*R*)-**30** (0.08 g, 0.22 mmol) in CH_2Cl_2 (2 mL) at room temperature, NBS (0.04 g, 0.22 mmol) was added, and the reaction mixture was stirred at this temperature for 5 h. The mixture was diluted with CH_2Cl_2, and the organic layer was washed with H_2O (2x). The organic layer was dried over anhydrous Na_2SO_4, and the solvent was concentrated. The residue was purified by flash column chromatography (CombiFlash® Rf+ system, 24 g silica gel, gradient from 50:50 to 30:70 *v/v* hexane/EtOAc, flow rate = 35 mL/min) to afford 0.07 g (72% yield) of (*R*)-*exo*-**31** and 9.3 mg (9% yield) of the *endo*-**31** isomer, both as light-yellow foams. Data for (*R*)-*exo*-**31**: **^{1}H NMR** (400.16 MHz, $CDCl_3$, 298 K) δ 7.45 (br s, 1H, ArH), 7.40 – 7.31 (m, 2H, ArH), 7.18 (td, *J* = 7.5, 1.1 Hz, 1H, ArH), 6.46 (s, 1H, H_{8a}), 3.93 (dd, *J* = 10.7, 6.2 Hz, 1H, H_2), 3.74 (s, 3H, $CO_2C\underline{H_3}$), 3.23 (dd, *J* = 12.7, 6.3 Hz, 1H, H_{3A}), 2.82 (dd, *J* = 12.7, 10.7 Hz, 1H, H_{3B}), 2.25 (s, 3H, $COC\underline{H_3}$), 1.58 (s, 9H, $CO_2t\underline{Bu}$) ppm. **^{13}C{^{1}H} NMR** (100.62 MHz, $CDCl_3$, 298 K) δ 171.0 (s), 170.6 (s), 152.6 (s), 141.0 (s), 133.3 (s), 130.9 (d), 125.7 (d), 123.2 (d), 119.8 (d), 84.6 (s), 83.6 (d), 60.4 (s), 59.8 (d), 52.7 (q, $CO_2\underline{C}H_3$), 40.8 (t), 28.3 (q, 3x, $CO_2C(\underline{C}H_3)_3$), 22.2 ($CO\underline{C}H_3$) ppm. **HRMS** (ESI-TOF) *m/z*: $[M + H]^+$ calcd. for $C_{19}H_{23}{}^{79}BrN_2O_5$, 439.0863; found, 439.0873. **IR** (NaCl): ν 3005 (w, C-H), 2979 (m, C-H), 2953 (w, C-H), 2935 (w, C-H), 1751 (s, C=O), 1714 (s, C=O), 1667 (s, C=O), 1604 (w), 1477 (s), 1438 (m), 1397 (s), 1368 (s), 1332 (s), 1278 (s), 1260 (s), 1204 (s), 1157 (s), 1085 (w), 1064 (w), 1012 (m), 958 (w), 854 (m), 755 (s) cm^{-1}. $[\alpha]_D^{22}$ +90.4 (*c* 0.22, $CHCl_3$).

(2*R*,3a*S*,8a*S*)-1-Acetyl-3a-(3'-methylbut-1-en-3'-yl)-(1,2,3,3a,8,8a)-hexahydropyrrolo[2,3-*b*]indole-2,8- dicarboxylic Acid 8-*tert*-Butyl Ester 2-Methyl Ester ((*R*)-*exo*-33)

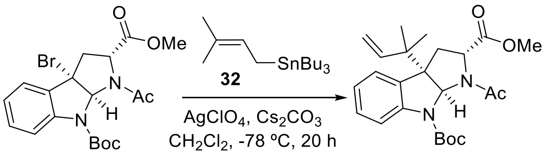

(*R*)-*exo*-**31** (*R*)-*exo*-**33**

General procedure for reverse prenylation with tributyl(3-methyl-2-butenyl)stannane. Method A. To a cooled (−78 °C) solution of the of *exo*-bromopyrroloindoline (*R*)-*exo*-**31** (0.05 g, 0.11 mmol), Cs_2CO_3 (0.06 g, 0.17 mmol) and tributyl(3-methyl-2-butenyl)stannane[5-6] **32** (0.06 g, 0.17 mmol) in CH_2Cl_2 (1.7 mL) was added $AgClO_4$ (0.05 g, 0.23 mmol). After stirring

at −78 °C for 20 h, the reaction was quenched with a saturated aqueous solution of NH_4Cl, and the mixture warmed up to room temperature. The layers were separated, and the aqueous layer was extracted with CH_2Cl_2 (3x). The combined organic layers were dried over anhydrous Na_2SO_4, filtered and the solvent was evaporated. The residue was purified by flash column chromatography (CombiFlash® Rf+ system, 12 g silica gel, gradient from 90:10 *v/v* hexane/EtOAc to EtOAc 100%, flow rate = 30 mL/min) to afford 23.0 mg (47% yield) of the titled compound as a colourless foam. **^{1}H NMR** (400.16 MHz, $CDCl_3$, 298 K) δ 7.38 – 7.22 (br m, 2H, ArH), 7.20 – 7.15 (m, 1H, ArH), 7.14 – 7.04 (m, 1H, ArH), 6.29 – 6.02 (br s, 1H, H_{8a}), 5.82 (dd, *J* = 17.4, 10.8 Hz, 1H, $C(CH_3)_2C\underline{H}=CH_2$), 5.09 (d, *J* = 10.6 Hz, 1H, $C(CH_3)_2CH=C\underline{H}_{cis}H$), 5.03 (dd, *J* = 17.4, 1.1 Hz, 1H, $C(CH_3)_2CH=C\underline{H}_{trans}H$), 3.83 (dd, *J* = 10.6, 6.5 Hz, 1H, H_2), 3.70 (s, 3H, $CO_2C\underline{H_3}$) 2.46 – 2.36 (m, 1H, H_{3A}), 2.34 – 2.17 (m, 4H, H_{3B} + $COC\underline{H_3}$), 1.53 (s, 9H, $CO_2C(C\underline{H_3})_3$), 1.06 (s, 3H, $C(C\underline{H_3})_2CH=CH_2$), 0.94 (s, 3H, $C(C\underline{H_3})_2CH=CH_2$) ppm. **^{13}C{^{1}H} NMR** (100.62 MHz, $CDCl_3$, 298 K) δ 172.5 (s), 171.5 (s), 153.0 (s), 143.0 (d), 142.2 (s), 133.8 (s), 128.8 (d), 124.9 (d), 124.3 (d), 119.4 (d), 114.8 (t), 82.6 (s), 79.5 (d), 62.9 (s), 59.7 (d), 52.4 (q), 40.4 (s), 34.0 (t), 28.3 (q, 3x, $CO_2C(\underline{C}H_3)_3$), 23.0 (q), 22.4 (q, 2x) ppm. **HRMS** (ESI-TOF) *m/z*: $[M + H]^+$ calcd. for $C_{24}H_{33}N_2O_5$, 429.2384; found, 429.2388. **IR** (NaCl): ν 2976 (w, C-H), 1750 (m, C=O), 1706 (s, C=O), 1664 (s, C=O), 1479 (m), 1398 (s), 1368 (m), 1332 (s), 1157 (s) cm^{-1}. **$[\alpha]_D^{22}$** +15.6 (*c* 0.22, $CHCl_3$).

(2*S*,3a*S*,8a*S*)-1-Acetyl-3a-(3’-methylbut-1-en-3’-yl)-(1,2,3,3a,8,8a)-hexahydropyrrolo[2,3-*b*]indole-2,8- dicarboxylic Acid 8-*tert*-Butyl Ester 2-Methyl Ester ((*S*)-*endo*-33)

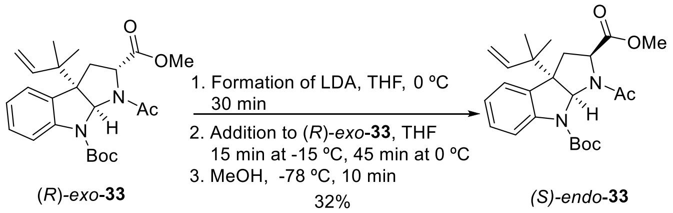

To a solution of diisopropylamine (33.0 µL, 24 mg, 0.24 mmol) in THF (0.8 mL) at 0 ºC, *n*-BuLi (0.09 mL, 2.12 M in hexane, 0.2 mmol) was added and the resulting mixture was stirred for 30 min at this temperature. The hexahydropyrroloindole (*R*)-*exo*-**33** was dissolved in THF (0.4 mL) and cooled down to –15 ºC. The solution of lithium diisopropylamide was added at this temperature and the reaction mixture was stirred at this temperature for 45 min. The enolate was quenched by addition of MeOH at -78 ºC. After stirring for 10 minutes, the reaction mixture was warmed to room temperature and diluted with EtOAc. A saturated aqueous solution of NH_4Cl was added and the aqueous layer was extracted with EtOAc (3x). The combined organic layers were washed with a saturated aqueous solution of NaCl, dried over anhydrous Na_2SO_4 and the solvent evaporated. The residue was purified by flash column chromatography (silica gel, 60:40 *v/v* hexane/EtOAc) to afford 13.5 mg (32% yield) of the titled

compound as a yellow foam. **^{1}H NMR** (400.16 MHz, $CDCl_3$, 298 K) δ 7.40 – 7.30 (m, 1H, ArH), 7.27 – 7.19 (m, 1H, ArH), 7.17 – 7.10 (m, 1H, ArH), 7.05 – 6.99 (m, 1H, ArH), 6.13 (br s, 1H, H_{8a}), 5.83 (dd, *J* = 17.4, 10.8 Hz, 1H, $C(CH_3)_2C\underline{H}=CH_2$), 5.16 – 5.00 (m, 2H, $C(CH_3)_2CH=C\underline{H_2}$), 4.88 (d, *J* = 9.3 Hz, 1H), 3.07 (s, 3H, $CO_2C\underline{H_3}$), 2.63 – 2.41 (m, 2H, $2H_3$), 2.37 (s, 3H, $COC\underline{H_3}$), 1.55 (s, 9H, $CO_2C(C\underline{H_3})_3$), 1.07 (s, 3H, $C(C\underline{H_3})_2CH=CH_2$), 0.91 (s, 3H, $C(C\underline{H_3})_2CH=CH_2$) ppm. **^{13}C{^{1}H} NMR** (100.62 MHz, $CDCl_3$, 298 K) δ 171.4 (s), 171.2 (s), 153.2 (s), 143.2 (d), 133.03 (s), 128.9 (d), 125.8 (d), 123.5 (d), 118.8 (d), 114.9 (t), 82.5 (s), 80.1 (d), 63.2 (s), 58.9 (d), 52.0 (q), 40.5 (s), 34.2 (t), 28.4 (q, 3x, $CO_2C(\underline{C}H_3)_3$), 22.8 (q, 2x), 22.4 (q) ppm. **HRMS** (ESI-TOF) *m/z*: $[M + H]^+$ calcd. for $C_{24}H_{33}N_2O_5$, 429.2384; found, 429.2385. **IR** (NaCl): ν 2975 (m, C-H), 2931 (m, C-H), 1750 (m, C=O), 1705 (s, C=O), 1666 (s, C=O), 1479 (m), 1398 (s), 1368 (s), 1334 (s), 1272 (m), 1256 (m), 1158 (s) cm^{-1}. $[\alpha]_D^{21}$ –25.5 (*c* 0.26, $CHCl_3$).

REMARK: one ^{13}C{^{1}H} NMR signal singlet is missing.

(2*R*,3a*S*,8a*S*,2’’*S*)-1-Acetyl-3a-bromo-8-{2’-[2”-(*tert*-butoxycarbonyl)amino-3’’-methylbutanamido]benzoyl}-(1,2,3,3a,8,8a)-hexahydropyrrolo[2,3-*b*]indole-2-carboxylic Acid Methyl Ester (*exo*-37) and (2*R*,3a*R*,8a*R*,2’’*S*)-1-Acetyl-3a-bromo-8-{2’-[2”-(*tert*-butoxycarbonyl)amino-3’’-methylbutanamido]benzoyl}-(1,2,3,3a,8,8a)-hexahydropyrrolo[2,3-*b*]indole-2-carboxylic Acid Methyl Ester (*endo*-37)

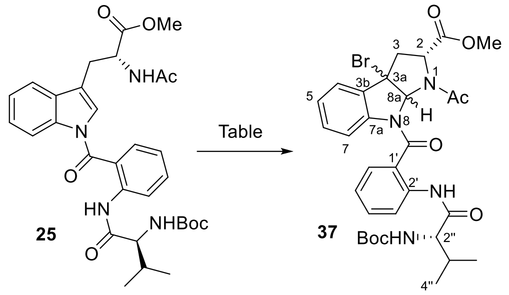

Entry	Reaction conditions	Yield
**1**	NBS (1.2 equiv.), CH_3CN, 25 ºC, 20 h	Quantitative (1.6:1 *endo*/*exo*)
**2**	Br_2 (4.8 equiv.), CH_2Cl_2, 0 ºC, 20 min	39% (1.2:1 *endo*/*exo*)
**3**	Br_2 **(1.8 equiv.)**, CH_2Cl_2, 0 ºC, **50 min**	68% (1:1.4 *endo*/*exo*)
**4**	NBS (1.2 equiv.), CH_2Cl_2, 25 ºC, 2.5 h	93% (1:1.2 *endo*/*exo*)
**5**	NBS (1.2 equiv.), CH_3CN, -30 ºC, 22 h	Quantitative* (2:1 *endo*/*exo*)
**6**	NBS (1.2 equiv.), CH_2Cl_2, -30 ºC- (-15 ºC), 22 h	84% (1:1.1 *endo*/*exo*)

* Different result depending on the scale

Following the general procedure described above for the bromocyclization with *N*-bromosuccinimide (Method A), the reaction of ester **25** (0.04 g, 0.07 mmol) and NBS (15 mg,

0.083 mmol) in CH_3CN (7 mL) at room temperature for 20 h afforded, after purification by flash column chromatography (silica gel, gradient from 70:30 to 10:90 *v/v* hexane/EtOAc), 18.0 mg of *exo*-**37** (40% yield) and 28.0 mg of *endo*-**37** (60% yield), both as beige solids.

Exo-**37**. **^{1}H NMR** (400.16 MHz, DMSO-d_6, 343 K) δ 9.73 (br s, NH), 7.75 – 7.69 (m, 1H), 7.55 – 7.43 (m, 1H), 7.31 – 6.77 (m, 6H), 6.63-6.30 (m, 2H, H_{8a} + NH), 4.13 – 3.92 (m, 2H, H_2 + $H_{2''}$), 3.76 – 3.57 (m, 4H, $CO_2C\underline{H_3}$ + H_{3A}), 2.85 – 2.68 (m, 1H, H_{3B}), 2.34 – 1.93 (m, 4H, $COC\underline{H_3}$ + $C\underline{H}(CH_3)_2$), 1.39 (s, 9H, CO_2*t*Bu), 0.95 (d, J = 6.8 Hz, 3H, $CH(C\underline{H_3})_2$), 0.91 (d, J = 6.8 Hz, 3H, $CH(C\underline{H_3})_2$) ppm. **^{13}C{^{1}H} NMR** (100.62 MHz, DMSO-d_6, 343 K, D1 = 4) δ 170.5 (s), 170.2 (s), 169.9 (s), 167.7 (s), 155.0 (s), 140.2 (s), 135.7 (s), 133.2 (s), 130.6 (d), 129.8 (d), 128.4 (d), 127.2 (d), 125.5 (d, 2x), 123.8 (d, 2x), 119.0 (s), 84.2 (d), 78.0 (s, $\underline{C}(CH_3)_3$), 61.0 (s), 59.9 (d), 59.1 (d), 52.0 (q, $CO_2\underline{C}H_3$), 39.4 (t, HSQC), 30.2 (d, $\underline{C}H(CH_3)_2$), 27.8 (q, 3x, $C(\underline{C}H_3)_3$), 21.7 (q, $CO\underline{C}H_3$), 18.8 (q, 3x, $CH(\underline{C}H_3)_2$), 17.4 (q, $CH(\underline{C}H_3)_2$) ppm. **HRMS** (ESI-TOF) *m/z*: $[M + H]^+$ calcd. for $C_{31}H_{38}{}^{79}BrN_4O_7$, 657.1918; found, 657.1906. **IR** (NaCl): ν 35400-3100 (br, N-H), 3006 (w, C-H), 2965 (m, C-H), 2930 (m, C-H), 2873 (w, C-H), 1751 (m, C=O), 1714 (m, C=O), 1673 (s, C=O), 1602 (m, C=O), 1585 (m), 1520 (m), 1477 (m), 1447 (m), 1398 (m), 1368 (m), 1320 (m), 1288 (m), 1206 (m), 1165 (s), 1096 (w), 1086 (w), 1036 (w), 1019 (w), 886 (w), 874 (w), 756 (s) cm^{-1}. $[\alpha]_D^{21}$ +70.4 (*c* 0.16, $CHCl_3$).

REMARK: due to the low resolution of the spectra, the integration of signals in the ^{1}H NMR spectrum is not accurate and some ^{13}C{^{1}H} NMR signals are missing.

Endo-**37**. **^{1}H NMR** (400.16 MHz, DMSO-d_6, **363 K**) δ 9.48 (br s, 1H, NH), 8.02 (br s, 1H, NH), 7.66 – 7.30 (m, 4H, ArH), 7.29 – 7.00 (m, 4H, ArH), 6.57 – 6.38 (m, 2H, H_{8a} + NH), 4.80 (d, J = 8.9 Hz, 1H, H_2), 3.96 (t, J = 7.0 Hz, 1H, $H_{2''}$), 3.42 – 3.35 (m, 1H, H_{3A}), 3.26 – 3.21 (m, 1H, H_{3B}), 3.19 (s, 3H, $CO_2C\underline{H_3}$), 2.22 – 2.09 (m, 1H, $C\underline{H}(CH_3)_2$), 2.09 – 1.91 (br s, 3H, $COC\underline{H_3}$), 1.43 (s, 9H, CO_2*t*Bu), 0.96 (d, J = 6.8 Hz, 3H, $CH(C\underline{H_3})_2$), 0.93 (d, J = 6.8 Hz, 3H, $CH(C\underline{H_3})_2$) ppm. **^{13}C{^{1}H} NMR** (100.62 MHz, DMSO-d_6, **343 K and 363 K**) δ 178.(s), 170.1 (s, rotamer: 170.0), 169.4 (s), 168.1 (s), 155.2 (s), 141.6 (s), 136.4 (s), 132.0 (s), 130.6 (d), 130.1 (d, 2x), 127.5 (d, 2x), 124.4 (d), 124.2 (d), 123.0 (d), 116.4 (s), 84.7 (d), 78.1 (s, $\underline{C}(CH_3)_3$), 60.8 (s, HMBC), 60.8 (d), 59.1 (d), 51.5 (q), 41.5 (t), 29.7 (d), 27.8 (q, 3x), 21.4 (q), 18.7 (q), 17.6 (q) ppm. **HRMS** (ESI-TOF) *m/z*: $[M + H]^+$ calcd. for $C_{31}H_{38}{}^{79}BrN_4O_7$, 657.1918; found, 657.1907. **IR** (NaCl): ν 3500-3100 (br, N-H), 3007 (w, C-H), 2965 (w, C-H), 2932 (w, C-H), 1716 (s, C=O), 1669 (s, C=O), 1601 (m), 1585 (m), 1524 (m), 1475 (m), 1447 (m), 1405 (m), 1370 (m), 1341 (m), 1289 (m), 1237 (m), 1163 (s), 1093 (w), 1018 (w), 870 (w), 848 (w), 757 (s) cm^{-1}. $[\alpha]_D^{21}$ –36.3 (*c* 0.13, $CHCl_3$).

REMARK: due to the low resolution of the spectra, the integration of signals in the ^{1}H NMR spectrum is not accurate and some ^{13}C{^{1}H} NMR signals are missing.

(2*R*,3a*S*,8a*S*,2''*S*)-1-Acetyl-8-{2'-[2''-(*tert*-butoxycarbonyl)amino-3''-methylbutanamido]benzoyl}-3a-(3'''-methylbut-1-en-3'''-yl)-(1,2,3,3a,8,8a)-hexahydropyrrolo[2,3-*b*]indole-2-carboxylic Acid Methyl Ester (*exo*-36)

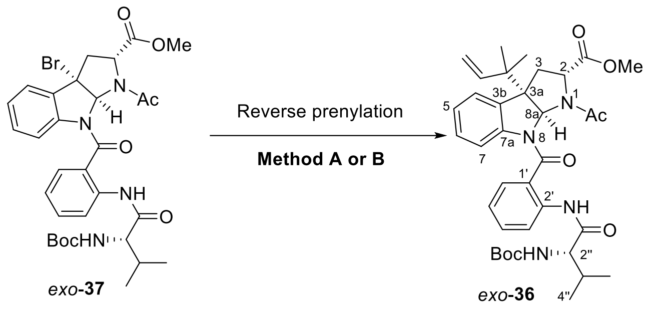

Method A. Following the general procedure described above for the reverse prenylation with tributyl(3-methyl-2-butenyl)stannane, the reaction of *exo*-bromopyrroloindoline *exo*-**37** (0.1 g, 0.16 mmol), Cs_2CO_3 (0.08 g, 235.0 μmol), prenyl tributylstannane (84.0 mg, 235.0 μmol) and $AgClO_4$ (0.1 g, 0.47 mmol) in CH_2Cl_2 (2.3 mL) at −78 °C for 20 h afforded, after purification by flash column chromatography (CombiFlash® Rf+ system, 24 g silica gel, gradient from 80:20 to 30:70 *v/v* hexane/EtOAc, flow rate = 35 mL/min), the desired compound *exo*-**36** (38.2 mg, 38% yield) as a white solid.

General procedure for the reverse prenylation with triisopropyl(3-methyl-2-butenyl)silane 38. **Method B.**[3] To a cooled (0 ºC) stirred solution of brominated precursor *exo*-**37** (0.1 g, 0.15 mmol), freshly activated 5Å MS (0.85 g), DTBP (0.05 mL,0.04 g, 0.22 mmol) and triisopropyl(3-methyl-2-butenyl)silane **38** (0.05 g, 0.22 mmol) in CH_2Cl_2 (3 mL) was added $AgNTf_2$ (0.13 g, 0.03 mmol). After stirring for one minute, the resulting mixture was allowed to warm up to room temperature, stirred for 1 h and then quenched with a saturated aqueous solution of $NaHCO_3$. The mixture was extracted with CH_2Cl_2 (3x) and the combined layers were washed with H_2O, filtered through a pad of Celite® and concentrated under reduced pressure. The residue was purified by flash column chromatography (silica gel, 70:30 *v/v* hexane/EtOAc), the desired compound *exo*-**36** (0.04 g, 41% yield) as a white solid. **^{1}H NMR** (400.16 MHz, DMSO-d_6, **353 K**) δ 9.23 (br s, 1H, NH), 8.01 (br s, 1H, NH), 7.54 – 7.46 (m, 1H, ArH), 7.42 (dd, J = 7.7, 1.3 Hz, 1H, ArH), 7.14 (td, J = 7.5, 1.1 Hz, 1H, ArH), 7.09 (td, J = 7.5, 1.1 Hz, 1H, ArH), 7.04 – 6.94 (m, 3H, ArH), 6.53 (br s, 1H, H_{8a}), 6.35 (br s, 1H, NH), 6.20 (br s, 1H, ArH, HSQC), 5.97 (dd, J = 17.4, 10.8 Hz, 1H, $C(CH_3)_2C\underline{H}=CH_2$), 5.17 (dd, J = 10.8, 1.1 Hz, 1H, $C(CH_3)_2CH=C\underline{H}_{cis}H$), 5.11 (d, J = 17.4 Hz, 1H, $C(CH_3)_2CH=C\underline{H}_{trans}H$), 4.16 – 3.83 (m, 2H, H_2 + $H_{2''}$), 3.69 (s, 3H, $CO_2C\underline{H_3}$), 2.73 – 2.59 (m, 1H, H_{3A}), 2.44 – 2.29 (m, 1H, H_{3B}), 2.20 – 1.94 (m, 4H, $COC\underline{H_3}$ + $C\underline{H}(CH_3)_2$), 1.35 (s, 9H, CO_2tBu), 1.13 (s, 3H, $C(C\underline{H_3})_2CH=CH_2$), 1.04 (s, 3H, $C(C\underline{H_3})_2CH=CH_2$), 0.92 (d, J = 6.8 Hz, 3H, $CH(C\underline{H_3})_2$), 0.89 (d, J = 6.8 Hz, 3H, $CH(C\underline{H_3})_2$) ppm. **^{13}C{^{1}H} NMR** (100.62 MHz, DMSO-d_6, **353 K**) δ 171.3 (s), 170.6 (s), 170.1 (s), 166.0 (s),

154.9 (s), 142.9 (d), 140.6 (s), 135.3 (s), 134.6 (s), 130.0 (d), 127.6 (d, 2x), 126.1 (d), 125.3 (d), 124.2 (d), 123.7 (d), 116.8 (d), 114.1 (t), 77.9 (s), 77.5 (d), 59.8 (d, 2x), 59.0 (s), 51.7 (q), 40.1 (s, HMBC), 34.5 (t), 30.2 (d, C̲H(CH$_3$)$_2$), 27.7 (q, 3x, C(C̲H$_3$)$_3$), 22.8 (q, C(C̲H$_3$)$_2$CH=CH$_2$), 22.1 (q, 2x, C(C̲H$_3$)$_2$CH=CH$_2$ + COC̲H$_3$), 18.6 (q, CH(C̲H$_3$)$_2$), 17.4 (q, CH(C̲H$_3$)$_2$) ppm. **HRMS** (ESI-TOF) *m/z*: $[M + H]^+$ calcd. for $C_{36}H_{47}N_4O_7$, 647.3439; found, 647.3442. **IR** (NaCl): ν 3500-3100 (br, N-H), 3008 (w, C-H), 2969 (m, C-H), 2930 (m, C-H), 2874 (w, C-H), 1748 (m, C=O), 1713 (m, C=O), 1695 (m, C=O), 1666 (s, C=O), 1612 (m), 1588 (m), 1540 (m), 1498 (m), 1477 (m), 1449 (m), 1401 (m), 1367 (m), 1336 (m), 1286 (m), 1241 (m), 1202 (m), 1174 (m), 1090 (w), 1040 (w), 1018 (w), 923 (w), 874 (w), 755 (m) cm^{-1}. $[\alpha]_D^{24}$ +33.0 (*c* 0.23, $CHCl_3$). REMARK: due to the low resolution of the spectra, the integration of signals in the ^{1}H NMR spectrum is not accurate and two $^{13}C\{^1H\}$ NMR signals are missing.

(2*R*,3a*S*,8a*S*,2''*S*)-1-Acetyl-8-{2'-[2''-(*tert*-butoxycarbonyl)amino-3''-methylbutanamido]benzoyl}-3a-(3'''-methylbut-1-en-3'''-yl)-(1,2,3,3a,8,8a)-hexahydropyrrolo[2,3-*b*]indole-2-carboxylic Acid (*exo*-39)

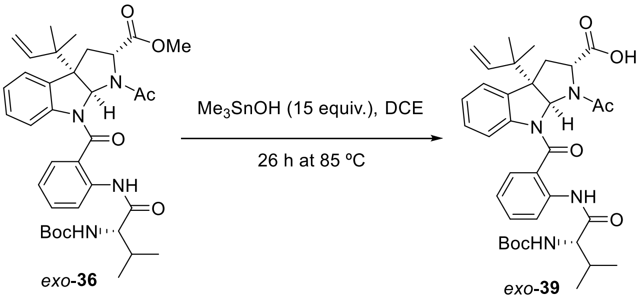

Following the general procedure described above for the hydrolysis of methyl esters, the reaction of *exo*-**36** (0.02 g, 0.03 mmol) with trimethyltin hydroxide (25.0 mg, 0.14 mmol) in DCE (0.6 mL) at 85 ºC (metal heating block) for 26 h afforded, after purification by flash column chromatography (silica gel, gradient from 95:5 to 90:10 CH_2Cl_2/MeOH), 16.0 mg (87%) of the titled compound as a highly hygroscopic white solid.

(2*R*,3a*S*,8a*S*,2'*S*)-1-Acetyl-8-[2'-(2''-amino-3''-methylbutanamido)benzoyl]-3a-(3'''-methylbut-1-en-3'''-yl)-(1,2,3,3a,8,8a)-hexahydropyrrolo[2,3-*b*]indole-2-carboxylic Acid (*exo*-40)

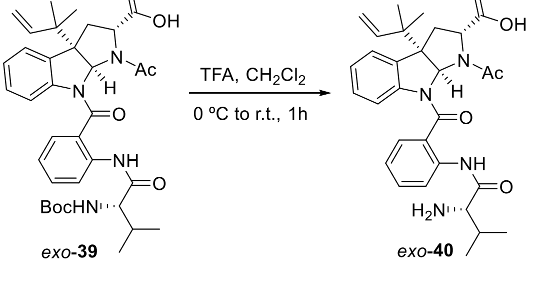

General procedure for the deprotection of the *N*-Boc group. TFA (0.29 mL, 0.43 g, 3.77 mmol) was added to a cooled (0 ºC) stirred solution of Boc-amide *exo*-**39** (11.5 mg, 0.02 mmol) in CH_2Cl_2 (2 mL), and the resulting mixture was stirred at room temperature for 1 h. The reaction mixture was concentrated *in vacuo.* The residue was redissolved in CH_2Cl_2 and concentrated again, and this process was repeated several times until it became a white solid, which was then subjected to high vacuum for 30 min. The crude trifluoroacetate salt was used directly in the next step without further purification.

New synthetic novofumigatamide from D-Trp and L-Val (D-Trp-regio-*exo*-4)

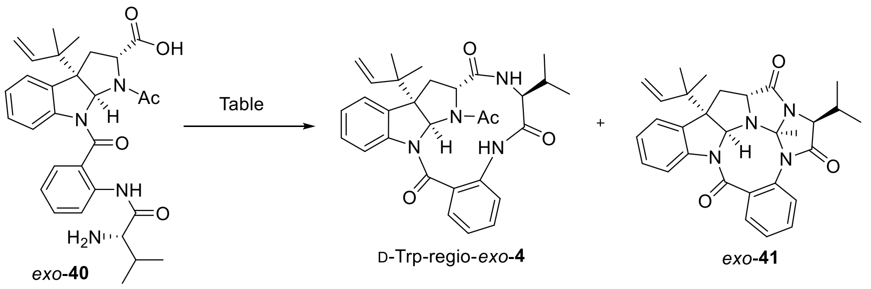

Entry	Reaction conditions	Yield*
**1**	HATU (1.2 equiv.), Et_3N (15 equiv.), CH_2Cl_2/DMF (15:1) [0.0006M], from 0 ºC to room temperature, 37 h. Addition of the activated substrate solution to a solution of the base over 18 h at a 0.4 mL/h flow rate with the syringe pump. Further stirring for 19 h.	The mass of the product was detected by HPLC-MS (~min 14). An unknown compound with molecular mass 498.286 g/mol was the major product in one of the fractions of the column. The other fraction was de diolide.
**2**	HATU (1.2 equiv.), Et_3N (15 equiv.), CH_2Cl_2/DMF (15:1) [0.0006M], from 0 ºC to room temperature, 17 h. Addition of the activated substrate solution to a solution of the base over 9 h at a 1.1 mL/h rate with the syringe pump. Further stirring for 8 h.	The mass of the product was detected by HPLC-MS (~min 14). By ^{1}H NMR it is difficult to draw a conclusion. The diolide was not detected.
**3**	HATU (3 equiv.), 6-Cl-HOBt (2.5 equiv.) DIPEA (20 equiv.), CH_2Cl_2/DMF (3:1) [0.0005M], 25 ºC, 6 days. Addition of the activated substrate solution to a solution of the base over 4 days at a 0.1 mL/h flow rate with the syringe pump. 2 days of additional stirring.	The mass of the product was never detected by HPLC-MS. The ^{1}H NMR of the crude showed mixture of compounds.
**4**	HATU (3 equiv.), Et_3N (15 equiv.), CH_2Cl_2/DMF (3:1) [0.0005M], from 0 ºC to **50 ºC**, 14 h. Addition of the activated substrate solution to a solution of the base **at 50 ºC** over 8 h with the syringe pump and 6 h of additional stirring.	The mass of the product was never detected by HPLC-MS. The ^{1}H NMR of the crude showed mixture of compounds.
**5**	HATU (3 equiv.), Et_3N (15 equiv.), CH_2Cl_2/DMF (3:1) [0.0005M], from **-30 ºC to rt**, **2 h**. No slow addition	The mass of the product was detected by HPLC-MS. An unknown compound with molecular mass 498.286 g/mol and 539.369 g/mol was the major compound in the crude

* Injection of aliquots of the reactions, the crudes and/or the fractions of the columns in HPLC-MS: Scharlau Kromaphase 100, C18, 5 μm, 250 x 4.6 mm, gradient from 50% CH_3CN/H_2O to 100% CH_3CN in 20 min, 1.0 mL/min.

Polycyclic by-product *exo*-41

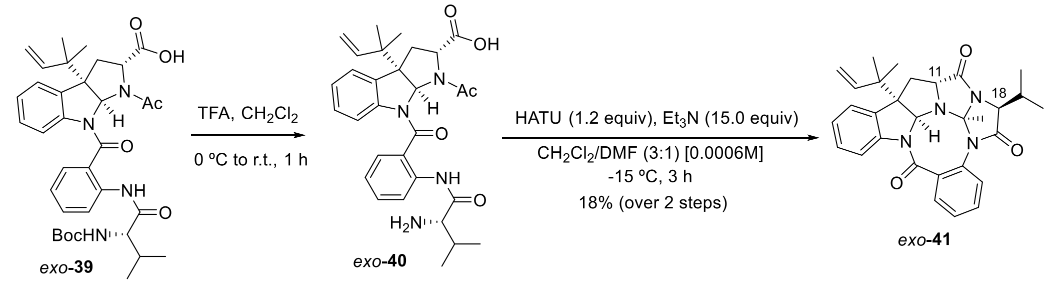

Following the general procedure described above for the deprotection of the *N*-Boc group, the reaction of *exo*-**39** (15.8 mg, 25.0 μmol), TFA (0.40 mL, 0.59 g, 5.19 mmol) in CH_2Cl_2 (2.8 mL) at room temperature for 2 h afforded a residue of *exo*-**40** that was used in the next step without further purification.

General procedure for the macrolactamization. Method A. To a solution of tryptophan derivative *exo*-**40** (13.0 mg, 25.0 μmol) in CH_2Cl_2 (31 mL) and DMF (10.5 mL), HATU (11.0 mg, 30.0 μmol) was added, and the resulting mixture was stirred at 0 ºC for 20 minutes. The solution of the activated acid was cooled down to –15 ºC and Et_3N (0.05 mL, 0.04 g, 0.38 mmol) was added. The mixture was further stirred for 3 h at this temperature. To the reaction mixture was added H_2O, the layers were separated, and the aqueous layer was extracted with CH_2Cl_2 (3x). The combined organic layers were washed with H_2O (6x), dried (anhydrous Na_2SO_4) and the solvent was evaporated. If DMF remained in the residue, it was dissolved again in EtOAc, washed several times with H_2O, dried (anhydrous Na_2SO_4) and the solvent was concentrated. The residue was purified by flash column chromatography (silica gel, 70:30 *v/v* hexane/EtOAc) to afford 2.2 mg (18% yield, over two steps) of the titled compound as a white solid. **^{1}H NMR** (400.16 MHz, $CDCl_3$, 298 K) δ 8.10 – 8.04 (m, 1H, ArH), 7.61 – 7.59 (m, 1H, ArH), 7.59 – 7.57 (m, 1H, ArH), 7.52 (td, *J* = 7.6, 1.7 Hz, 1H, ArH), 7.44 (td, *J* = 7.5, 1.3 Hz, 1H, ArH), 7.37 (ddd, *J* = 8.1, 7.4, 1.3 Hz, 1H, ArH), 7.35 – 7.28 (m, 1H, ArH), 5.92 (dd, *J* = 17.4, 10.8 Hz, 1H, $C(CH_3)_2C\underline{H}=CH_2$), 5.20 (s, 1H, H_2), 5.12 (dd, *J* = 10.8, 1.0 Hz, 1H, $C(CH_3)_2CH=C\underline{H}_{cis}H$), 5.05 (dd, *J* = 17.4, 1.0 Hz, 1H, $C(CH_3)_2CH=C\underline{H}_{trans}H$), 4.12 (d, *J* = 2.5 Hz, 1H, H_{18}), 4.01 (dd, *J* = 9.7, 6.6 Hz, 1H, H_{11}), 3.19 – 3.07 (m, 1H, $C\underline{H}(CH_3)_2$), 2.34 – 2.28 (m, 2H, $2H_3$), 1.40 (s, 3H, $N_3CC\underline{H}_3$), 1.23 (d, *J* = 7.2 Hz, 3H, $CH(C\underline{H}_3)_2$), 1.11 (s, 3H, $C(C\underline{H}_3)_2CH=CH_2$), 0.90 (s, 3H, $C(C\underline{H}_3)_2CH=CH_2$), 0.82 (d, *J* = 6.7 Hz, 3H, $CH(C\underline{H}_3)_2$) ppm. **^{13}C{^{1}H} NMR** (100.62 MHz, $CDCl_3$, 298 K) δ 171.3 (s), 170.7 (s), 168.1 (s), 144.0 (s), 143.6 (d), 135.7 (s), 131.6 (d), 130.6 (d), 129.5 (s), 129.3 (d), 128.1 (d), 127.8 (d), 125.1 (d), 124.5 (d), 118.2 (d), 115.4 (t), 91.2 (s), 76.4 (d), 69.6 (s), 66.3 (d), 66.2 (d), 41.7 (s), 36.3 (t), 26.0 (q), 24.4 (d), 23.6 (q), 23.2 (q), 18.8 (q), 16.4 (q) ppm. **HRMS** (ESI-TOF) *m/z*: $[M + H]^+$ calcd. for $C_{30}H_{33}N_4O_3$, 497.2547; found, 497.2551. **IR** (NaCl): ν 2961 (w, N-H), 2919 (m, N-H), 2849 (w,

C-H), 1719 (s, C=O), 1663 (m), 1475 (m), 1460 (m), 1381 (m) cm^{-1}. $[\alpha]_D^{20}$ + 182.8 (*c* 0.11, $CHCl_3$).

REMARK: one $^{13}C\{^1H\}$ NMR signal is missing.

(2*R*,3a*R*,8a*R*,2''*S*)-1-Acetyl-8-{2'-[2"-(*tert*-butoxycarbonyl)amino-3''-methylbutanamido]benzoyl}-3a-(3'''-methylbut-1-en-3'''-yl)-(1,2,3,3a,8,8a)-hexahydropyrrolo[2,3-*b*]indole-2-carboxylic Acid Methyl Ester (*endo*-36)

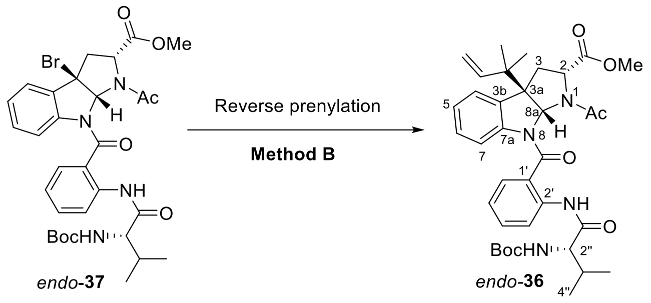

Following the general procedure described above for the reverse prenylation with triisopropyl(3-methyl-2-butenyl)silane (**Method B**), the reaction of *endo*-bromopyrroloindoline *endo*-**37** (0.05 g, 0.08 mmol), prenyl triisopropyl silane **38** (0.03 g, 0.11 mmol), freshly activated 5Å MS (0.42 g), DTBP (0.03 mL, 0.02 g, 0.11 mmol) and $AgNTf_2$ (0.06 g, 0.15 mmol) in CH_2Cl_2 (1.5 mL) at room temperature for 1 h afforded, after purification by flash column chromatography (CombiFlash® Rf+ system, 12 g silica gel, gradient from 80:20 to 60:40 *v/v* hexane/EtOAc, flow rate = 30 mL/min), the desired compound *endo*-**36** (15.0 mg, 31% yield) as a white solid. **M.p.**: 203-205 ºC (hexane/toluene). **^{1}H NMR** (400.16 MHz, DMSO-d_6, 343 K) δ 7.55 – 7.46 (m, 1H, ArH), 7.32 – 7.07 (m, 4H, ArH), 7.03 – 6.90 (m, 2H, ArH), 6.49 (br, 1H, H_{8a}), 6.39 (br, 1H, ArH, HSQC), 6.02 (dd, *J* = 17.3, 10.7 Hz, 1H, $C(CH_3)_2C\underline{H}=CH_2$), 5.16 (dd, *J* = 10.9, 1.2 Hz, 1H, $C(CH_3)_2CH=C\underline{H}_{cis}H$), 5.12 (d, *J* = 17.7 Hz, 1H, $C(CH_3)_2CH=C\underline{H}_{trans}H$), 4.97 – 4.82 (m, 1H, H_2), 3.96 (app. t, *J* = 7.3 Hz, 1H, $H_{2''}$), 3.08 (s, 3H, CO_2CH_3), 2.71 – 2.59 (m, 1H, H_{3A}), 2.57 – 2.42 (m, 1H, H_{3B}), 2.22 – 1.94 (m, 4H, $COC\underline{H_3}$ + $C\underline{H}(CH_3)_2$), 1.39 (s, 9H, CO_2tBu), 1.11 (s, 3H, $C(C\underline{H_3})_2CH=CH_2$), 1.02 (s, 3H, $C(C\underline{H_3})_2CH=CH_2$), 0.88 (d, *J* = 6.9 Hz, 3H, $CH(C\underline{H}_3)_2$), 0.86 (d, *J* = 6.8 Hz, 3H, $CH(C\underline{H}_3)_2$) ppm. **^{13}C{^{1}H} NMR** (100.62 MHz, DMSO-d_6, 343 K) δ 170.4 (s, 2x), 169.6 (s), 167.0 (s), 155.0 (s), 143.4 (d), 142.4 (s), 135.4 (s), 132.9 (s), 130.3 (d), 127.7 (d), 127.0 (d), 125.5 (d), 123.9 (d), 123.0 (d), 115.6 (d), 114.0 (t), 78.8 (s, $\underline{C}(CH_3)_3$), 78.0 (d, C_{8a}), 60.3 (d), 59.1 (d), 59.1 (s, HMBC), 51.2 (q, $CO_2\underline{C}H_3$), 40.1 (s, HMBC), 35.2 (t), 30.0 (d, $\underline{C}H(CH_3)_2$), 27.8 (q, 3x, $C(\underline{C}H_3)_3$), 22.8 (q, $C(\underline{C}H_3)_2CH=CH_2$), 22.3 (q, $C(\underline{C}H_3)_2CH=CH_2$), 21.7 (q, $CO\underline{C}H_3$), 18.7 (q, $CH(\underline{C}H_3)_2$), 17.5 (q, $CH(\underline{C}H_3)_2$) ppm. **HRMS** (ESI-TOF) *m/z*: $[M + H]^+$ calcd. for $C_{36}H_{47}N_4O_7$, 647.3439; found, 647.3439. **IR** (NaCl): ν 3500-3100 (br, N-H), 3004 (w, C-H), 2969 (m, C-H), 2934 (w, C-H), 2875 (w, C-H), 1715 (s, C=O), 1691 (s, C=O), 1667 (s, C=O), 1602 (m), 1586

(m), 1518 (m), 1479 (m), 1448 (m), 1392 (m), 1367 (m), 1336 (m), 1235 (m), 1202 (m), 1165 (m), 1089 (w), 1042 (w), 1013 (w), 924 (w), 874 (w), 754 (m) cm^{-1}. **$[\alpha]_D^{22}$** +11.4 (*c* 0.26, $CHCl_3$). REMARK: due to the low resolution of the spectra, the integration of the ^{1}H NMR spectrum is not accurate and two $^{13}C\{^1H\}$ NMR signals are missing.

(2*R*,3a*R*,8a*R*,2''*S*)-1-Acetyl-8-{2'-[2"-(*tert*-butoxycarbonyl)amino-3''-methylbutanamido]benzoyl}-3a-(3'''-methylbut-1-en-3'''-yl)-(1,2,3,3a,8,8a)-hexahydropyrrolo[2,3-*b*]indole-2-carboxylic Acid (*endo*-39)

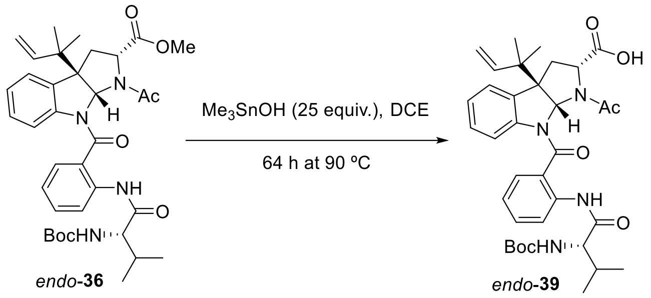

Following the general procedure described above for the hydrolysis of methyl esters, the reaction of *endo*-**36** (15.0 mg, 23.0 μmol), trimethyltin hydroxide (0.1 g, 0.58 mmol) in DCE (0.5 mL) at 90 ºC (metal heating block) for 64 h afforded, after purification by flash column chromatography (silica gel, 90:10 *v/v* CH_2Cl_2/MeOH), 6 mg (42% yield) of the titled compound as a highly hygroscopic white solid.

(2*R*,3a*R*,8a*R*,2''*S*)-1-Acetyl-8-[2'-(2"-amino-3''-methylbutanamido)benzoyl]-3a-(3'''-methylbut-1-en-3'''-yl)-(1,2,3,3a,8,8a)-hexahydropyrrolo[2,3-*b*]indole-2-carboxylic Acid (*endo*-40)

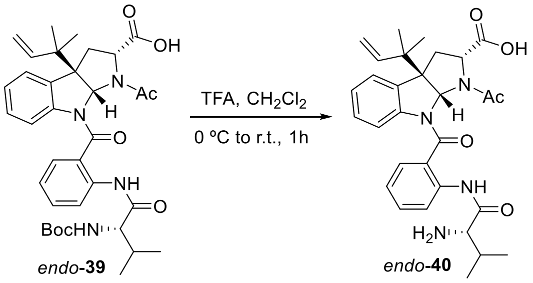

Following the general procedure described above for the deprotection of the *N*-Boc group, the reaction of *endo*-**39** (6.0 mg, 9.5 μmol), TFA (0.15 mL, 0.22 g, 1.97 mmol) in CH_2Cl_2 (1 mL) at room temperature for 1 h afforded a residue that was used in the next step without further purification.

New synthetic novofumigatamide from D-Trp and L-Val (D-Trp-regio-*endo*-4)

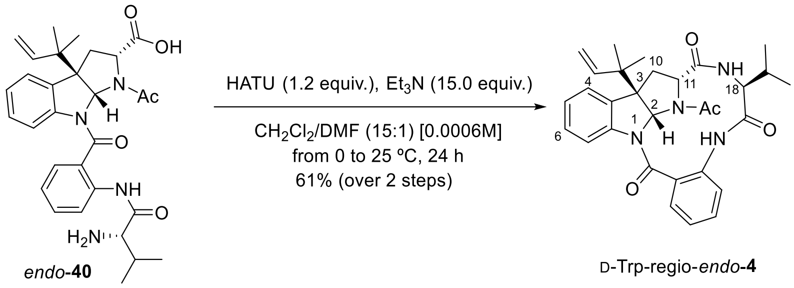

General procedure for the macrolactamization. Method B. To a solution of the tryptophan derivative *endo*-**40** (5.0 mg, 9.5 μmol) in CH_2Cl_2 (7 mL) and DMF (1 mL), HATU (4.0 mg, 11.4 μmol) was added, and the resulting mixture was stirred at 0 ºC for 20 minutes. The solution of the activated acid was added to a solution of Et_3N (20 μL, 14.4 mg, 0.14 mmol) in CH_2Cl_2 (8 mL) via syringe pump over a period of 7 h and at flow rate of 1.07 mL/h. The mixture was further stirred for 17 h while the temperature was raised from 0 ºC to room temperature. To the reaction mixture was added H_2O, the layers were separated, and the aqueous layer was extracted with CH_2Cl_2 (3x). The combined organic layers were washed with H_2O (6x), dried (anhydrous Na_2SO_4) and the solvent was evaporated. If DMF remained in the residue, it was dissolved again in EtOAc, washed several times with H_2O, dried (anhydrous Na_2SO_4) and the solvent was concentrated. The residue was purified by flash column chromatography (silica gel, 60:40 *v/v* hexane/EtOAc) to afford 3.0 mg (61% yield, over two steps) of the titled compound as a white solid. **^{1}H NMR** (400.16 MHz, $CDCl_3$, 298 K) δ 8.56 (dd, *J* = 8.4, 0.8 Hz, 1H, ArH), 8.36 (br s, 1H, NH), 7.63 – 7.50 (m, 1H, ArH), 7.49 – 7.34 (m, 2H, ArH), 7.30 – 7.26 (m, 1H, ArH), 7.06 (td, *J* = 7.6, 1.0 Hz, 1H, ArH), 6.89 (ddd, *J* = 8.6, 7.4, 1.3 Hz, 1H, ArH), 6.54 (s, 1H, H_2), 6.05 (dd, *J* = 17.3, 10.8 Hz, 1H, $C(CH_3)_2C\underline{H}=CH_2$), 5.73 (d, *J* = 8.3 Hz, 1H, ArH), 5.32 – 5.13 (m, 3H, $C(CH_3)_2CH=C\underline{H_2}$ + H_{11}), 4.21 (dd, *J* = 8.5, 3.6 Hz, 1H, H_{18}), 2.82 (d, *J* = 12.9 Hz, 1H, H_{10A}), 2.66 (s, 3H, $COC\underline{H_3}$), 2.58 – 2.45 (m, 1H, $C\underline{H}(CH_3)_2$), 2.41 (dd, *J* = 12.9, 9.0 Hz, 1H, H_{10B}), 1.23 (s, 3H, $C(C\underline{H_3})_2CH=CH_2$), 1.04 (s, 3H, $C(C\underline{H_3})_2CH=CH_2$), 0.95 (d, *J* = 6.9 Hz, 3H, $CH(C\underline{H_3})_2$), 0.84 (d, 3H, *J* = 7.0 Hz, $CH(C\underline{H_3})_2$) ppm. **^{13}C{^{1}H} NMR** (100.62 MHz, $CDCl_3$, 298 K) δ 173.8 (s), 170.8 (s), 169.1 (s), 167.8 (s), 143.1 (d), 139.5 (s), 134.8 (s), 134.5 (s), 132.6 (d), 129.2 (d), 127.7 (d), 126.2 (s), 126.0 (d), 125.0 (d), 124.9 (d), 119.8 (d), 115.6 (t), 111.8 (d), 79.7 (d), 62.0 (s), 61.3 (d), 59.4 (d), 42.4 (s), 34.8 (t), 28.6 (d, $\underline{C}H(CH_3)_2$), 23.4 (q, $C(\underline{C}H_3)_2CH=CH_2$), 23.0 (q, $C(\underline{C}H_3)_2CH=CH_2$), 21.8 (q, $CO\underline{C}H_3$), 19.8 (q, $CH(\underline{C}H_3)_2$), 16.7 (q, $CH(\underline{C}H_3)_2$) ppm. **HRMS** (ESI-TOF) *m/z*: $[M + H]^+$ calcd. for $C_{30}H_{35}N_4O_4$, 515.2653; found, 515.2646. **IR** (NaCl): ν 3388 (w, N-H), 3300 (w, N-H), 2961 (m, C-H), 2924 (m, C-H), 2871 (w, C-H), 2852 (w, C-H), 1703 (s, C=O), 1649 (s, C=O), 1583 (m), 1532 (m), 1509 (m), 1483 (m), 1451 (m), 1394 (m), 1367 (m), 1321 (m), 1304 (m), 1362 (m), 1203 (w), 1163 (w), 1447 (w), 1087 (w), 1039 (w), 1008 (w), 919 (w), 899 (w), 753 (m) cm^{-1}. **$[\alpha]_D^{24}$** +49.5 (*c* 0.14, $CHCl_3$).

REMARK: some solubility issues were found when MeOH was used as solvent to measure the specific optical rotations for some intermediates and final products. Remarkably, the first final product synthesized, the proposed structure of novofumigatamide (D-Trp-*exo*-**1**),[3] was not fully soluble in this solvent, which prompted us to use $CHCl_3$ as solvent to measure this physical property for this and the subsequent final products.

Other routes explored towards D-Trp-regio-*exo*-4

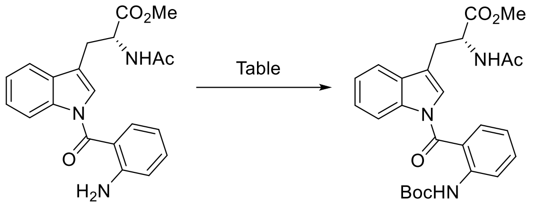

(*R*)-**23** **50**

Entry	Conditions	Comments/Yield (%)
**1**	Boc_2O (3.2 equiv.), Et_3N (1.6 equiv.)<br>DMAP (0.2 equiv.), THF<br>from rt to 50 ºC, 3 days<br>(*Tetrahedron* **2014**, *70*, 5541)[7]	The reaction did not work. Recovery of the starting material (13% yield) and a 32% yield of a secondary product which corresponds to the D-tryptophan derivative without the anthranilic unit and the unprotected indole.
**2**	Boc_2O (1.1 equiv.), EtOH (not dried),<br>60 ºC, 16 h<br>(*Nat. Chem. Biol.* **2012**, *8*, 823)[8]	Poor conversion to the product. The SM and the product have a very similar Rf and are difficult to separate by flash column chromatography.
**3**	Boc_2O (1.1 equiv.), Amberlyst®-15 hydrogen form, EtOH (not dried), rt, 22 h<br>(*Angew. Chem. Int. Ed.* **2016**, *55*, 12643)[9]	Same result of **Entry 2**
**4**	Boc_2O (1.1 equiv.), CH_2Cl_2, rt, 40 h	Same result of **Entry 2**
**5**	1. Boc_2O (2.1 equiv.), EtOH (dried), 60 ºC, 19 h.<br>2. Addition of Amberlyst®-15 hydrogen form, 60 ºC, 3 days. Addition of 1 equiv. of Boc_2O.	Conditions of **entry 2** were used with anhydrous EtOH. The reaction was monitored by TLC and HPLC-MS, which showed that most of the SM was left. Then, three portions of Amberlyst resin (**entry 3**) were added over a period of 3 days (3 x 0.038 g resin/mmol substrate), together with another equivalent of Boc_2O. The reaction did not evolve and the ^1H-NMR spectrum of the crude showed unreacted SM, reaction product and descomposition products.

Methyl (*R*)-2-Acetylamino-3-{1'-[2"-({[(9*H*-Fluoren-9^{iv}-yl)methoxy]carbonyl}amino) benzoyl]indole-3'-yl}propanoate 51

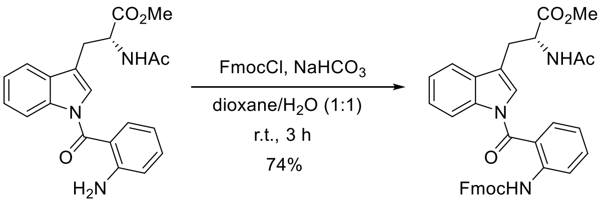

(*R*)-**23** **51**

A solution of 9-fluorenylmethoxycarbonyl chloride (34.0 mg, 0.13 mmol) in dioxane (0.53 mL) was added to a stirred solution of the tryptophan derivative (*R*)-**23** (0.05 g, 0.13 mmol) in a 10% aqueous solution of $NaHCO_3$ (0.53 mL). After 3 h of stirring at 25 ºC, water was added, and the resulting mixture was extracted with EtOAc (4x). The combined organic layers were dried over

anhydrous Na_2SO_4, filtered and the solvent was evaporated. The residue was purified by flash column chromatography (CombiFlash® Rf+ system, 12 g silica gel, gradient from 70:30 to 30:70 *v/v* hexane/EtOAc, flow rate = 30 mL/min) to afford 0.14 g (74% yield) of the titled compound as a white solid. **^{1}H NMR** (400.16 MHz, DMSO-d_6, 343 K) δ 9.57 (s, 1H, NH), 8.20 (d, *J* = 7.5 Hz, 1H, ArH), 8.12 (d, *J* = 7.7 Hz, 1H, NH), 7.84 (d, *J* = 7.5 Hz, 2H, ArH), 7.65 – 7.44 (m, 6H, ArH), 7.39 (t, *J* = 7.5 Hz, 2H, ArH), 7.37 – 7.22 (m, 5H, ArH), 7.12 (s, 1H, ArH), 4.54 (app. td, *J* = 8.2, 5.7 Hz, 1H, H_2), 4.24 (d, *J* = 7.0 Hz, 2H, $OC\underline{H_2}CHAr_2$), 4.08 (t, *J* = 7.0 Hz, 1H, $OCH_2C\underline{H}Ar_2$), 3.58 (s, 3H, $CO_2C\underline{H_3}$), 3.08 (dd, *J* = 14.7, 5.1 Hz, 1H, H_{3A}), 2.95 (dd, *J* = 15.0, 8.4 Hz, 1H, H_{3B}), 1.77 (s, 3H, $COC\underline{H_3}$) ppm. **^{13}C{^{1}H} NMR** (100.62 MHz, $CDCl_3$, 298 K) δ 171.7 (s), 169.0 (s), 166.2 (s), 153.8 (s), 143.3 (s), 140.4 (s), 136.1 (s), 135.4 (s), 131.3 (d), 130.3 (s), 129.2 (d), 127.5 (s), 127.3 (d), 126.7 (d), 125.7 (d), 124.8 (d), 124.2 (d), 124.0 (d), 123.9 (d), 123.1 (d), 119.7 (d), 118.5 (d), 116.3 (s), 115.6 (d), 65.9 (t), 51.7 (d), 51.5 (q), 46.3 (d), 26.2 (t), 21.9 (q) ppm. **HRMS** (ESI-TOF) *m/z*: $[M + H]^+$ calcd. for $C_{36}H_{32}N_3O_6$, 602.2285; found, 602.2282. **IR** (NaCl): ν 3400-3100 (br, N-H), 3065 (w, C-H), 3016 (w, C-H), 2952 (w, C-H), 1738 (s, C=O), 1664 (s, C=O), 1602 (m), 1585 (m), 1524 (s), 1451 (s), 1370 (s), 1330 (s), 1302 (m), 1217 (s), 1042 (m), 755 (s) cm^{-1}. $[\alpha]_D^{22}$ –17.5 (*c* 0.23, $CHCl_3$).

(*R*)-2-Acetylamino-3-{1’-[2”-({[(9*H*-Fluoren-9^{iv}-yl)methoxy]carbonyl}amino) benzoyl]indole-3’-yl}propanoic Acid 52

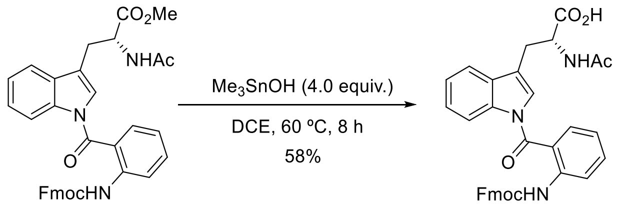

51 **52**

Following the general procedure described above for the hydrolysis of methyl esters, the reaction of tryptophan derivative **51** (0.9 g, 1.5 mmol) with trimethyltin hydroxide (1.08 g, 6.0 mmol) in DCE (15 mL) at 60 ºC (metal heating block) for 8 h afforded, after purification by flash column chromatography (silica gel, gradient from 95:5 to 90:10 *v/v* CH_2Cl_2/MeOH), 0.51 g (58% yield) of the titled compound as a white solid. **^{1}H NMR** (400.16 MHz, DMSO-d_6, 343 K) δ 9.55 (s, 1H, NH), 8.22 – 8.12 (m, 1H), 7.96 (d, *J* = 6.9 Hz, 1H, NH, ArH), 7.84 (dt, *J* = 7.6, 1.0 Hz, 2H, ArH), 7.63 – 7.45 (m, 6H, ArH), 7.39 (tt, *J* = 7.5, 1.0 Hz, 2H, ArH), 7.34 – 7.21 (m, 5H, ArH), 7.12 (s, 1H, ArH), 4.53 – 4.39 (m, 1H, H_2), 4.23 (d, *J* = 7.1 Hz, 2H, $OC\underline{H_2}CHAr_2$), 4.07 (t, *J* = 7.0 Hz, 1H, $OCH_2C\underline{H}Ar_2$), 3.09 (dd, *J* = 14.8, 5.0 Hz, 1H, H_{3A}), 2.92 (dd, *J* = 14.8, 8.8 Hz, 1H, H_{3B}), 1.75 (s, 3H, $COC\underline{H_3}$) ppm. **^{13}C{^{1}H} NMR** (100.62 MHz, DMSO-d_6, 343 K) δ 172.6 (s), 168.8 (s), 166.2 (s), 153.7 (s), 143.3 (s), 140.4 (s), 136.1 (s), 135.3 (s), 131.3 (d), 130.4 (s), 129.2 (d), 127.4 (s), 127.3 (d), 126.7 (d), 125.6 (d), 124.8 (d), 124.1 (d), 124.0 (d),

123.9 (d), 123.1 (d), 119.7 (d), 118.6 (d), 116.7 (s), 115.6 (d), 65.9 (t), 51.6 (d), 46.2 (d), 26.3 (t), 22.0 (q) ppm. **HRMS** (ESI-TOF) *m/z*: $[M + H]^+$ calcd. for $C_{35}H_{30}N_3O_6$, 588.2129; found, 588.2127. **IR** (NaCl): ν 3400-3100 (br, O-H, N-H), 3065 (m, C-H), 3017 (m, C-H), 2952 (m, C-H), 1733 (s, C=O), 1664 (s, C=O), 1602 (m), 1521 (s), 1451 (s), 1367 (s), 1330 (s), 1215 (s), 1042 (m), 754 (s) cm^{-1}. $[\alpha]_D^{23}$ –60.9 (*c* 0.24, $CHCl_3$).

Methyl (2*S*,2'*R*)-2-[2'-(Acetylamino)-3'-{1''-[2'''-({[(9*H*-Fluoren-9^{iv}-yl)methoxy]carbonyl}amino)benzoyl]indole-3''-yl}propanamido]-3-methylbutanoate 53

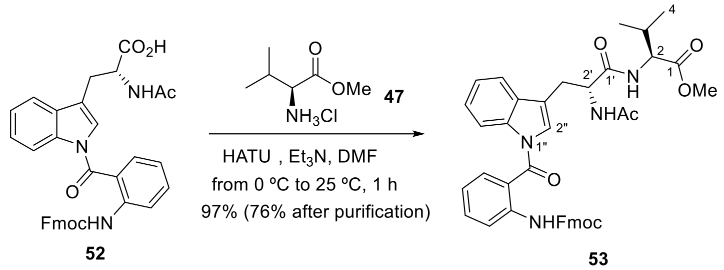

General procedure for amide (peptide) bond formation. Method B. To a cooled (0 ºC) solution of the tryptophan derivative **52** (0.45 g, 0.77 mmol) and L-valine methyl ester hydrochloride **47** (0.15 g, 0.92 mmol) in DMF (10 mL) were added Et_3N (0.26 mL, 0.19 g, 1.84 mmol) and HATU (0.35 g, 0.92 mmol) and the mixture was stirred for 2 h at 25 ºC. Water (10 mL) was added to the reaction mixture, the layers were separated, and the aqueous layer was extracted with EtOAc (3x). The combined organic layers were washed with a saturated aqueous solution of NaCl (3x), dried over anhydrous Na_2SO_4 and the solvent was evaporated. The residue (0.52 g, 97% yield) was purified by flash column chromatography (CombiFlash® Rf+ system, 80 g silica gel, gradient from 50:50 to 20:80 *v/v* hexane/EtOAc, flow rate = 60 mL/min) to obtain 0.41 g (76% yield) of the titled compound as a white solid. ^{1}**H NMR** (400.16 MHz, DMSO-*d*$_6$, 343 K) δ 9.53 (s, 1H, NH), 8.20 – 8.10 (m, 2H, ArH + NH), 7.88 (d, *J* = 8.5 Hz, 1H, NH), 7.84 (dt, *J* = 7.6, 1.0 Hz, 2H, ArH), 7.68 – 7.63 (m, 1H, ArH), 7.62 – 7.57 (m, 1H, ArH), 7.56 – 7.50 (m, 3H, ArH), 7.47 (dd, *J* = 7.7, 1.7 Hz, 1H, ArH), 7.41 – 7.35 (m, 2H, ArH), 7.34 – 7.23 (m, 5H, ArH), 7.14 (s, 1H, ArH), 4.70 (td, *J* = 8.6, 5.5 Hz, 1H, $H_{2'}$), 4.22 (d, *J* = 7.0 Hz, 2H, $OC\underline{H_2}CHAr_2$), 4.11 (dd, *J* = 8.4, 6.4 Hz, 1H, H_2), 4.08 – 4.03 (m, 1H, $OCH_2C\underline{H}Ar_2$), 3.61 (s, 3H, $CO_2C\underline{H_3}$), 2.99 (dd, *J* = 14.7, 5.1 Hz, 1H, H_{3A}), 2.82 (dd, *J* = 14.4, 8.8 Hz, 1H, H_{3B}), 2.00 – 1.87 (m, 1H, $C\underline{H}(CH_3)_2$), 1.74 (s, 3H, $COC\underline{H_3}$), 0.75 (d, *J* = 6.8 Hz, 6H, $CH(\underline{CH_3})_2$) ppm. ^{13}**C{^{1}H} NMR** (100.62 MHz, DMSO-*d*$_6$, 343 K) δ 171.5 (s), 171.2 (s), 168.8 (s), 166.2 (s), 153.7 (s), 143.4 (s), 143.3 (s), 140.4 (s), 136.1 (s), 135.3 (s), 131.3 (d), 130.6 (s), 129.3 (d), 127.4 (s), 127.3 (d), 126.7 (d), 125.7 (d), 124.8 (d), 124.1 (d), 124.0 (d), 123.9 (d), 122.9 (d), 119.7 (d), 119.0 (d), 116.8 (s), 115.5 (d), 65.9 (t), 57.1 (d), 52.0 (d), 51.3 (q), 46.3 (d), 29.7 (d), 27.5 (t), 22.1 (q), 18.5 (q), 17.8 (q) ppm. **HRMS** (ESI-TOF) *m/z*: $[M + H]^+$ calcd. for $C_{41}H_{41}N_4O_7$, 701.2970; found, 701.2969. **IR** (NaCl): ν 3400-

3100 (br, N-H), 3066 (w, C-H), 3018 (w, C-H), 2962 (w, C-H), 1739 (s, C=O), 1649 (s, C=O), 1584 (m), 1523 (s), 1451 (s), 1364 (s), 1330 (m), 1303 (m), 1213 (s), 1041 (m), 755 (s) cm^{-1}. $[\alpha]_D^{23}$ +35.0 (*c* 0.26, $CHCl_3$).

Methyl (2*S*,2'''*S*)-2-Acetylamino-3-(1'-{2"-[2'''-(*tert*-butoxycarbonyl)amino-3'''-methylbutanamido]benzoyl}indole-3'-yl)propanoate (*S*)-25

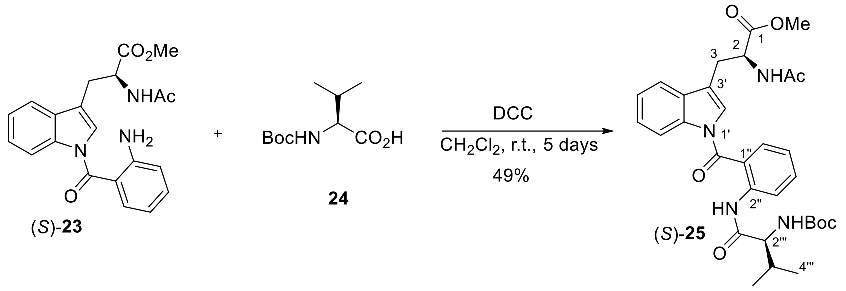

Following the general procedure described above for the amide (peptide) bond formation (Method A), the reaction of the indole derivative (*S*)-**23** (0.5 g, 1.32 mmol), *N*-(*tert*-butoxycarbonyl)-L-valine **24** (0.32 g, 1.45 mmol) and DCC (0.6 g, 2.9 mmol) in CH_2Cl_2 (44 mL) at room temperature for 5 days afforded, after purification by flash column chromatography (silica gel, gradient from 50:20:30 *v/v* hexane/ CH_2Cl_2/EtOAc), 0.37 g (49% yield) of the titled compound as a white solid. **^{1}H NMR** (400.16 MHz, DMSO-d_6, 338 K) δ 9.94 (br s, 1H, NH), 8.21 – 8.10 (m, 2H, ArH + NH), 7.69 – 7.56 (m, 3H, ArH), 7.44 (dd, *J* = 7.5, 1.3 Hz, 1H, ArH), 7.36 – 7.28 (m, 3H, ArH), 7.17 (s, 1H, ArH), 6.50 (br s, 1H, NH), 4.54 (td, *J* = 8.3, 5.5 Hz, 1H, H_2), 3.91 (t, *J* = 7.2 Hz, 1H, $H_{2'''}$), 3.60 (s, 3H, $CO_2C\underline{H}_3$), 3.15 – 3.08 (m, 1H, H_{3A}), 2.98 (ddd, *J* = 14.7, 8.6, 0.9 Hz, 1H, H_{3B}), 1.97 – 1.86 (m, 1H, $C\underline{H}(CH_3)_2$), 1.79 (s, 3H, $COC\underline{H}_3$), 1.33 (s, 9H, CO_2tBu), 0.82 (d, *J* = 6.8 Hz, 3H, $CH(C\underline{H}_3)_2$), 0.76 (d, *J* = 6.8 Hz, 3H, $CH(C\underline{H}_3)_2$) ppm. **^{13}C{^{1}H} NMR** (100.62 MHz, DMSO-d_6, 338 K) δ 171.6 (s), 170.3 (s), 168.9 (s), 166.0 (s), 155.0 (s), 135.5 (s), 135.4 (s), 131.2 (d), 130.3 (s), 128.8 (d), 127.3 (s), 125.7 (d), 124.1 (d), 124.0 (d), 123.6 (d), 123.1 (d), 118.4 (d), 116.2 (s), 115.6 (d), 77.9 (s, $\underline{C}(CH_3)_3$), 59.9 (d), 51.6 (d), 51.4 (q, $CO_2\underline{C}H_3$), 29.9 (d, $\underline{C}H(CH_3)_2$), 27.8 (q, 3x, $C(\underline{C}H_3)_3$), 26.2 (t), 21.9 (q, $CO\underline{C}H_3$), 18.7 (q, $CH(\underline{C}H_3)_2$), 17.4 (q, $CH(\underline{C}H_3)_2$) ppm. **HRMS** (ESI-TOF) *m/z*: $[M + H]^+$ calcd. for $C_{31}H_{39}N_4O_7$, 579.2813; found, 579.2821. **IR** (NaCl): ν 3500-3100 (br, N-H), 3006 (w, C-H), 2969 (m, C-H), 2933 (w, C-H), 1743 (m, C=O), 1687 (s, C=O), 1671 (s, C=O), 1604 (m), 1582 (m), 1523 (s), 1453 (s), 1368 (s), 1332 (m), 1299 (m), 1255 (m), 1219 (m), 1172 (m), 1042 (w), 1018 (w), 881 (w), 754 (s), 666 (w) cm^{-1}. $[\alpha]_D^{22}$ –6.0 (*c* 0.12, $CHCl_3$).

(2*S*,3a*R*,8a*R*,2'*S*)-1-Acetyl-3a-bromo-8-{2'-[2"-(*tert*-butoxycarbonyl)amino-3''-methylbutanamido]benzoyl}-(1,2,3,3a,8,8a)-hexahydropyrrolo[2,3-*b*]indole-2-carboxylic Acid Methyl Ester ((*S*)-*exo*-37) and (2*S*,3a*S*,8a*S*,2'*S*)-1-Acetyl-3a-bromo-8-{2'-[2"-(*tert*-butoxycarbonyl)amino-3''-methylbutanamido]benzoyl}-(1,2,3,3a,8,8a)-hexahydropyrrolo[2,3-*b*]indole-2-carboxylic Acid Methyl Ester ((*S*)-*endo*-37)

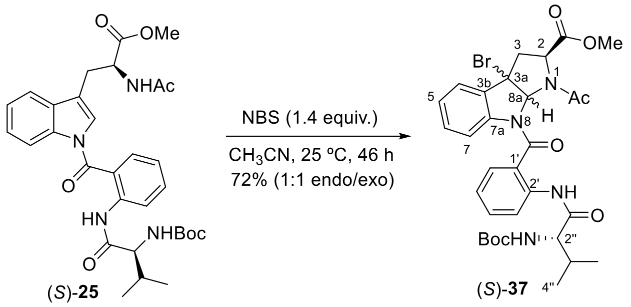

Following the general procedure described above for the bromocyclization with *N*-bromosuccinimide (**Method A**), the reaction of (*S*)-**25** (0.2 g, 0.35 mmol) with NBS (0.09 g, 0.48 mmol) in CH_3CN (34.6 mL) at room temperature for 46 h afforded, after purification by flash column chromatography (CombiFlash® Rf+ system, 80 g silica gel, gradient from 60:40 to 30:70 *v/v* hexane/EtOAc, flow rate = 60 mL/min), 73 mg of (*S*)-*exo*-**37** (32% yield) and 90 mg of the (*S*)-*endo*-**37** isomer (40% yield) as beige solids.

(*S*)-*Exo*-**37**. **^{1}H NMR** (400.16 MHz, DMSO-d_6, **353 K**) δ 9.82 (br s, 1H, NH), 7.85 – 6.76 (m, 8H, ArH), 6.71 – 6.27 (m, 2H, H_{8a} + NH), 4.07 – 3.87 (m, 2H, H_2 + $H_{2''}$), 3.76 – 3.56 (m, 4H, $CO_2\underline{CH_3}$ + H_{3A}), 2.87 – 2.64 (m, 1H, H_{3B}), 2.36 – 1.90 (m, 4H, $COCH_3$ + $C\underline{H}(CH_3)_2$), 1.41 (s, 9H, CO_2tBu), 0.96 (d, *J* = 6.8 Hz, 3H, $CH(C\underline{H_3})_2$), 0.91 (d, *J* = 6.8 Hz, 3H, $CH(C\underline{H_3})_2$) ppm. **$^{13}C\{^1H\}$ NMR** (100.62 MHz, DMSO-d_6, **353 K**) δ 170.6 (s), 170.2 (s), 170.0 (s), 167.9 (s), 155.1 (s), 140.4 (s), 135.7 (s), 133.1 (s), 130.6 (d, 2x), 129.8 (d), 127.4 (d, 2x), 125.5 (d), 123.8 (d, 2x), 119.9 (s), 84.4 (d), 78.0 (s), 61.3 (s), 60.5 (d), 59.1 (d), 52.0 (q), 39.5 (t, HSQC), 30.0 (d), 27.9 (q, 3x), 21.9 (q), 18.8 (q), 17.9 (q) ppm. **HRMS** (ESI-TOF) *m/z*: $[M + H]^+$ calcd. for $C_{31}H_{38}{}^{79}BrN_4O_7$, 657.1918; found, 657.1912. **IR** (NaCl): ν 3500-3100 (br, N-H), 3009 (w, C-H), 2966 (m, C-H), 2932 (w, C-H), 1751 (m, C=O), 1714 (m, C=O), 1669 (s, C=O), 1604 (m), 1586 (m), 1516 (m), 1477 (m), 1448 (m), 1401 (m), 1369 (m), 1344 (m), 1320 (m), 1289 (m), 1207 (m), 1165 (s), 1096 (w), 1087 (w), 885 (w), 874 (w), 754 (s) cm^{-1}. $[\alpha]_D^{26}$ –90.5 (*c* 0.16, $CHCl_3$).

REMARK: due to the low resolution of the spectra, the integration of the ^{1}H NMR spectrum is not accurate and some $^{13}C\{^1H\}$ NMR signals are missing.

(*S*)-*Endo*-**37**. **^{1}H NMR** (400.16 MHz, DMSO-d_6, 343 K) δ 7.67 – 7.38 (m, 4H, ArH), 7.36 – 6.84 (m, 4H, ArH), 6.70 – 6.27 (m, 2H, H_{8a} + NH), 4.79 (app. d, *J* = 9.1 Hz, 1H, H_2), 4.03 – 3.89 (m, 1H, $H_{2''}$), 3.45-3.31 (m, 1H, H_{3A}), 3.30-2.99 (m, 4H, H_{3B} + $CO_2C\underline{H_3}$), 2.21 – 2.06 (m, 1H, $C\underline{H}(CH_3)_2$), 2.08 – 1.86 (br s, 3H, $COC\underline{H_3}$), 1.40 (s, 9H, CO_2tBu), 0.94 (d, *J* = 6.8 Hz, 3H, $CH(C\underline{H_3})_2$), 0.91 (d, *J* = 6.8 Hz, 3H, $CH(C\underline{H_3})_2$) ppm. **$^{13}C\{^1H\}$ NMR** (100.16 MHz, DMSO-d_6,

343 K) δ 178.6 (s), 170.1 (s, rotamer: 170.2), 169.4 (s), 168.0 (s), 155.1 (s), 141.5 (s), 136.4 (s), 132.1 (s), 130.6 (d, 2x), 130.1 (d), 127.6 (d), 124.4 (d), 124.2 (d, 2x), 123.2 (d), 116.6 (s), 84.5 (d), 78.0 (s), 60.5 (d), 59.1 (d), 51.5 (q), 41.6 (t), 29.9 (d), 27.8 (q, 3x), 21.4 (q), 18.8 (q), 17.5 (q) ppm. **HRMS** (ESI-TOF) *m/z*: $[M + H]^+$ calcd. for $C_{31}H_{38}{}^{79}BrN_4O_7$, 657.1918; found, 657.1914. **IR** (NaCl): ν 3500-3100 (br, N-H), 3072 (w, C-H), 3008 (w, C-H), 2969 (w, C-H), 1713 (s, C=O), 1670 (s, C=O), 1600 (w), 1585 (w), 1521 (m), 1475 (m), 1405 (m), 1370 (m), 1342 (m), 1288 (m), 1237 (m), 1172 (s), 1092 (w), 1035 (w), 849 (w), 757 (s) cm^{-1}. $[\alpha]_D^{26}$ +29.2 (*c* 0.2, $CHCl_3$).

REMARK: due to the low resolution of the spectra, the integration of signals in the ^1H NMR spectrum is not accurate and some $^{13}C\{^1H\}$ NMR signals are missing.

The two pairs of diastereomers (*R*)-*exo*-**37**/(*R*)-*endo*-**37** (from D-Trp and L-Val) and (*S*)-*exo*-**37**/(*S*)-*endo*-**37** (from L-Trp and L-Val) displayed almost identical ^1H NMR and $^{13}C\{^1H\}$ NMR spectra. The structure of (*R*)-*endo*-**37** and the prenylated derivative (*R*)-*exo*-**36** (from D-Trp and L-Val) were inequivocally determined by X-ray diffraction analysis. To check that an epimerization did not occur at any of the stereocenters of the molecules, the four diastereomers were subjected to separation by HPLC-MS. The chromatograms obtained are shown in section "**4. Spectra collection and HPLC-MS traces**".

(2*S*,3a*R*,8a*R*,2'*S*)-1-Acetyl-8-{2'-[2"-(*tert*-butoxycarbonyl)amino-3''-methylbutanamido]benzoyl}-3a-(3'''-methylbut-1-en-3'''-yl)-(1,2,3,3a,8,8a)-hexahydropyrrolo[2,3-*b*]indole-2-carboxylic Acid Methyl Ester ((*S*)-*exo*-36)

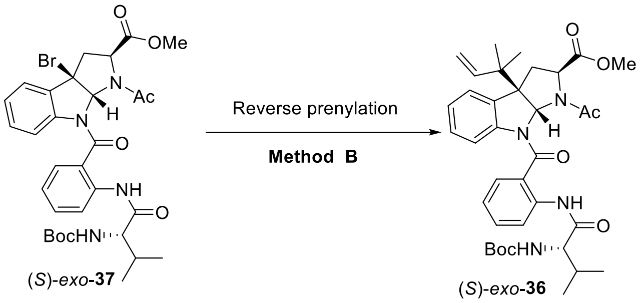

Following the general procedure described above for the reverse prenylation with triisopropyl(3-methyl-2-butenyl)silane (**Method B**), the reaction of *exo*-bromopyrroloindoline (*S*)-*exo*-**37** (0.04 g, 0.05 mmol), prenyl triisopropyl silane **38** (0.02 g, 0.08 mmol), freshly activated 5Å MS (0.28 g), DTBP (0.02 mL,0.02 g, 0.08 mmol) and $AgNTf_2$ (0.05 g, 0.11 mmol) in CH_2Cl_2 (1.1 mL) at room temperature for 1 h afforded, after purification by flash column chromatography (silica gel, 60:40 *v/v* hexane/EtOAc), the desired compound (*S*)-*exo*-**36** (12.3 mg, 39% yield) as a white solid. ^{1}**H NMR** (400.16 MHz, DMSO-d_6, 343 K) δ 9.35 (br s, 1H, NH), 7.50 (td, *J* = 7.8, 1.6 Hz, 1H, ArH), 7.42 (dd, *J* = 7.7, 1.3 Hz, 1H, ArH), 7.16 (td, *J* = 7.5, 1.2 Hz, 1H, ArH), 7.10 (td, *J* = 7.5, 1.1 Hz, 1H, ArH), 7.05 – 6.96 (m, 3H, ArH), 6.51 (br s, 1H,

H_{8a}), 6.42 (br s, 1H, NH), 6.28 (br s, 1H, ArH, HSQC), 5.98 (dd, *J* = 17.4, 11.1 Hz, 1H, $C(CH_3)_2C\underline{H}=CH_2$), 5.18 (dd, *J* = 10.8, 1.1 Hz, 1H, $C(CH_3)_2CH=C\underline{H}_{cis}H$), 5.12 (d, *J* = 17.3 Hz, 1H, $C(CH_3)_2CH=C\underline{H}_{trans}H$), 4.07 – 3.90 (m, 2H, H_2 + $H_{2''}$), 3.66 (s, 3H, $CO_2C\underline{H_3}$), 2.69 – 2.54 (m, 1H, H_{3A}), 2.41 – 2.28 (m, 1H, H_{3B}), 2.22 – 1.94 (m, 4H, $COC\underline{H_3}$ + $C\underline{H}(CH_3)_2$), 1.40 (s, 9H, CO_2*t*Bu), 1.13 (s, 3H, $C(C\underline{H_3})_2CH=CH_2$), 1.04 (s, 3H, $C(C\underline{H_3})_2CH=CH_2$), 0.86 (d, *J* = 6.7 Hz, 3H, $CH(C\underline{H}_3)_2$), 0.84 (d, *J* = 6.8 Hz, 3H, $CH(C\underline{H}_3)_2$) ppm. **^{13}C{^{1}H} NMR** (100.62 MHz, DMSO-d_6, 343 K) δ 171.2 (s), 170.6 (s), 170.0 (s), 166.4 (s), 154.9 (s), 143.0 (d), 140.7 (s), 135.3 (s), 134.6 (s), 130.1 (d), 127.6 (d), 126.2 (d), 125.4 (d), 124.4 (d), 124.0 (d), 117.1 (d), 114.2 (t), 78.0 (d), 77.7 (s), 60.0 (d, 2x), 59.1 (s), 51.6 (q), 40.3 (s, HMBC), 34.5 (t), 30.3 (d), 27.8 (q, 3x), 22.9 (q), 22.2 (q, 2x, $C(\underline{C}H_3)_2CH=CH_2$ + $CO\underline{C}H_3$), 18.7 (q), 17.4 (q) ppm. **HRMS** (ESI-TOF) *m/z*: $[M + H]^+$ calcd. for $C_{36}H_{47}N_4O_7$, 647.3439; found, 647.3434. **IR** (NaCl): ν 3500-3100 (br, N-H), 2968 (m, C-H), 2930 (m, C-H), 1748 (m, C=O), 1717 (m, C=O), 1665 (s, C=O), 1587 (m), 1477 (m), 1448 (m), 1392 (m), 1367 (m), 1335 (m), 1286 (m), 1202 (m), 1173 (m), 1038 (w), 1010 (w), 923 (w), 876 (w), 755 (m) cm^{-1}. $[\alpha]_D^{24}$ –24.1 (*c* 0.25, $CHCl_3$).

(2*S*,3a*R*,8a*R*,2'*S*)-1-Acetyl-8-{2'-[2"-(*tert*-butoxycarbonyl)amino-3''-methylbutanamido]benzoyl}-3a-(3'''-methylbut-1-en-3'''-yl)-(1,2,3,3a,8,8a)-hexahydropyrrolo[2,3-*b*]indole-2-carboxylic Acid ((*S*)-*exo*-39)

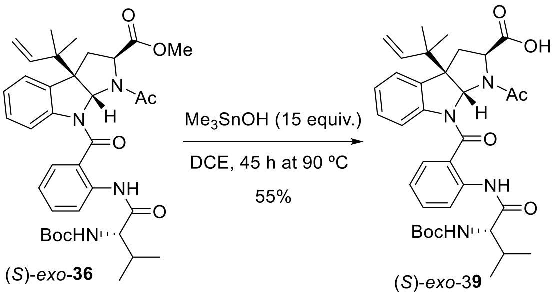

Following the general procedure described above for the hydrolysis of methyl esters, the reaction of (*S*)-*exo*-**36** (13.2 mg, 20.0 μmol) with trimethyltin hydroxide (0.06 g, 0.31 mmol) in DCE (0.3 mL) at 90 ºC (metal heating block) for 14 h afforded, after purification by flash column chromatography (silica gel, gradient from 95:5 to 90:10 *v/v* CH_2Cl_2/MeOH), 7 mg (55% yield) of the titled compound as a highly hygroscopic white solid.

(2*S*,3a*R*,8a*R*,2'*S*)-1-Acetyl-8-[2'-(2"-amino-3''-methylbutanamido)benzoyl]-3a-(3'''-methylbut-1-en-3'''-yl)-(1,2,3,3a,8,8a)-hexahydropyrrolo[2,3-*b*]indole-2-carboxylic Acid ((*S*)-*exo*-40)

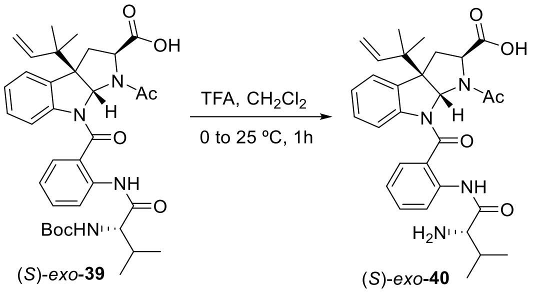

Following the general procedure described above for the deprotection of the *N*-Boc protected amines, the reaction of (*S*)-*exo*-**39** (7.1 mg, 11.2 μmol) with TFA (0.18 mL, 0.27 g, 2.33 mmol) in CH_2Cl_2 (1.3 mL) at room temperature for 1 h afforded a residue that was used in the next step without further purification.

New synthetic novofumigatamide from L-Trp and L-Val (L-Trp-regio-*exo*-4)

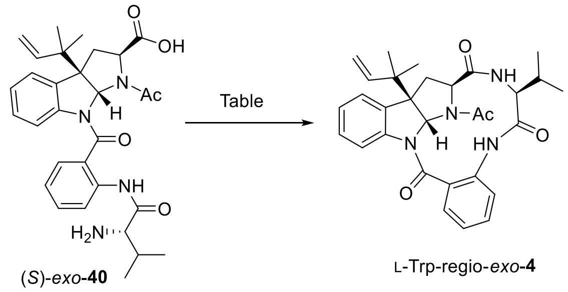

Entry	Reaction conditions	Yield*
**1**	HATU (1.2 equiv.), Et_3N (15 equiv.), CH_2Cl_2/DMF (10:1 *v/v*) [0.0006M], from 0 ºC to r.t., 20 h. Addition of the activated substrate solution to a solution of the base over 14 h (0.5 mL/h flow rate) with the syringe pump. Further stirring for 6 h.	The HPLC-MS chromatogram of an aliquot of the reaction and the crude showed the peak of the product. Nevertheless, it could not be isolated after flash column chromatography.
**2**	HATU (1.2 equiv.), Et_3N (15 equiv.), CH_2Cl_2/DMF (10:1) [0.0006M], from 0 ºC to r.t., 20 h. Addition of the activated substrate solution to a solution of the base over 14 h (0.72 mL/h flow rate) with the syringe pump. **Further stirring for 1 h. Repetition of entry 1 in a larger scale.**	In the HPLC-MS chromatogram of the crude we clearly observed the peak of the product. After flash column chromatography, the product was purified again by HPLC-MS, without success. The product seems to be unstable.

* Injection of aliquots of the reactions, the crudes and/or the fractions of the columns in HPLC-MS: Scharlau Kromaphase 100, C18, 5 μm, 250 x 4.6 mm, gradient from 50% CH_3CN/H_2O to 100% CH_3CN in 20 min, 1.0 mL/min.

The general procedure for the macrolactamization (**Method B**) was used. To a solution of tryptophan derivative (*R*)-*exo*-**40** (6 mg, 11.2 μmol) in CH_2Cl_2 (9 mL) and DMF (1 mL), HATU (5.1 mg, 13.4 μmol) was added, and the resulting mixture was stirred at 0 ºC for 20 min. The solution of the activated acid was added to a solution of Et_3N (22 μL, 16 mg, 0.16 mmol) in CH_2Cl_2 (9 mL) via syringe pump over a period of **14 h** and at flow rate of **0.72 mL/h** while the

temperature raised from 0 ºC to room temperature. To the reaction mixture was added H_2O, the layers were separated, and the aqueous layer was extracted with CH_2Cl_2 (3x). The combined organic layers were washed with H_2O (6x), dried (anhydrous Na_2SO_4) and the solvent was evaporated. If DMF remained in the crude, it was dissolved again in EtOAc, washed several times with H_2O, dried (anhydrous Na_2SO_4) and the solvent was concentrated. The residue was purified by flash column chromatography (silica gel, 90:10 *v/v* hexane/EtOAc) to afford 1.2 mg (21% yield, over two steps) of the titled compound as a white solid. Although the fraction of the column was further purified by RP-HPLC (Scharlau, C18 Kromaphase, 5 mm, 250 x 4.6 mm, gradient from 50% CH_3CN/H_2O to 100% CH_3CN in 20 min, 1.0 mL/min), the product decomposed before a full characterization could be performed.

(2*S*,3a*S*,8a*S*,2'*S*)-1-Acetyl-8-{2'-[2"-(*tert*-butoxycarbonyl)amino-3''-methylbutanamido]benzoyl}-3a-(3'''-methylbut-1-en-3'''-yl)-(1,2,3,3a,8,8a)-hexahydropyrrolo[2,3-*b*]indole-2-carboxylic Acid Methyl Ester ((*S*)-*endo-36*)

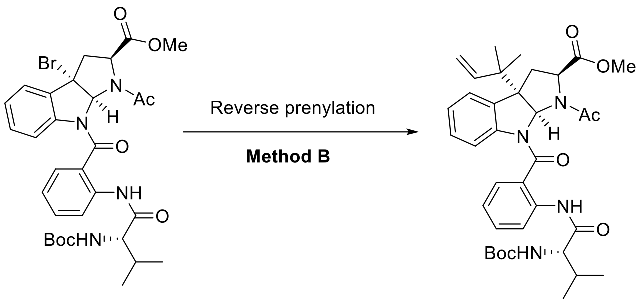

(*S*)-*endo*-**37** (*S*)-*endo*-**36**

Following the general procedure described above for the reverse prenylation with triisopropyl(3-methyl-2-butenyl)silane (**Method B**), the reaction of *endo*-bromopyrroloindoline (*S*)-*endo*-**37** (0.05 g, 0.08 mmol), prenyl triisopropyl silane **38** (0.03 g, 0.11 mmol), freshly activated 5Å MS (0.42 g), DTBP (0.03 mL, 0.02 g, 0.11 mmol) and $AgNTf_2$ (0.06 g, 0.15 mmol) in CH_2Cl_2 (1.5 mL) at room temperature for 1 h afforded, after purification by flash column chromatography (silica gel, 60:40 *v/v* hexane/EtOAc) the desired compound (15 mg, 32% yield) as a white solid. **^{1}H NMR** (400.16 MHz, DMSO-d_6, 343 K) δ 7.59 – 7.47 (m, 1H, ArH), 7.37 – 7.02 (m, 4H, ArH), 7.01 – 6.88 (m, 2H, ArH), 6.49 (br s, 1H, H_{8a}), 6.41 (br s, 1H, NH), 6.24 (d, *J* = 7.3 Hz, 1H, ArH, HSQC), 6.02 (dd, *J* = 17.4, 10.8 Hz, 1H, $C(CH_3)_2C\underline{H}=CH_2$), 5.16 (dd, *J* = 10.8, 1.2 Hz, 1H, $C(CH_3)_2CH=C\underline{H}_{cis}H$), 5.11 (d, *J* = 17.4 Hz, 1H, $C(CH_3)_2CH=C\underline{H}_{trans}H$), 4.97 – 4.80 (m, 1H, H_2), 4.08 – 3.93 (m, 1H, $H_{2''}$), 3.08 (s, 3H, $CO_2C\underline{H}_3$), 2.74 – 2.59 (m, 1H, H_{3A}), 2.57 – 2.46 (m, 1H, H_{3B}), 2.22 – 1.91 (m, 4H, $COC\underline{H}_3$ + $C\underline{H}(CH_3)_2$), 1.38 (s, 9H, CO_2tBu), 1.11 (s, 3H, $C(C\underline{H}_3)_2CH=CH_2$), 1.01 (s, 3H, $C(C\underline{H}_3)_2CH=CH_2$), 0.90 (d, *J* = 6.8 Hz, 3H, $CH(C\underline{H}_3)_2$), 0.87 (d, *J* = 6.9 Hz, 3H, $CH(C\underline{H}_3)_2$) ppm. **^{13}C{^{1}H} NMR** (100.62 MHz, DMSO-d_6, 343 K) δ 170.4 (s), 170.2 (s), 169.9 (s), 166.9 (s), 155.0 (s), 143.3 (d), 142.3 (s), 135.4 (s), 132.9 (s), 130.4 (d), 127.7 (d), 127.0 (d), 125.6 (d),

123.7 (d), 122.9 (d), 115.2 (d), 114.0 (t), 78.0 (d, C_{8a}), 77.9 (s, $\underline{C}(CH_3)_3$, HMBC), 60.1 (d), 59.0 (d), 51.2 (q, $CO_2\underline{C}H_3$), 40.1 (s, HMBC), 35.0 (t), 30.2 (d, $\underline{C}H(CH_3)_2$), 27.8 (q, 3x, $C(\underline{C}H_3)_3$), 22.7 (q, $C(\underline{C}H_3)_2CH{=}CH_2$), 22.3 (q, $C(\underline{C}H_3)_2CH{=}CH_2$), 21.7 (q, $CO\underline{C}H_3$), 18.7 (q, $CH(\underline{C}H_3)_2$), 17.4 (q, $CH(\underline{C}H_3)_2$) ppm. **HRMS** (ESI-TOF) *m/z*: $[M + H]^+$ calcd. for $C_{36}H_{47}N_4O_7$, 647.3439; found, 647.3430. **IR** (NaCl): ν 3500-3100 (br, N-H), 2964 (m, C-H), 2929 (m, C-H), 2873 (w, C-H), 1718 (s, C=O), 1694 (s, C=O), 1670 (s, C=O), 1586 (m), 1477 (m), 1449 (m), 1392 (m), 1367 (m), 1330 (m), 1286 (m), 1235 (m), 1171 (m), 1090 (w), 1041 (w), 922 (w), 874 (w), 754 (m) cm^{-1}. $[\alpha]_D^{23}$ –19.0 (*c* 0.3, $CHCl_3$).

REMARK: due to the low resolution of the spectra, the integration of signals in the ^1H NMR spectrum is not accurate and two $^{13}C\{^1H\}$ NMR signals are missing.

(2*S*,3a*S*,8a*S*,2’*S*)-1-Acetyl-8-{2’-[2”-(*tert*-butoxycarbonyl)amino-3’’-methylbutanamido]benzoyl}-3a-(3’’’-methylbut-1-en-3’’’-yl)-(1,2,3,3a,8,8a)-hexahydropyrrolo[2,3-*b*]indole-2-carboxylic Acid ((*S*)-*endo*-39)

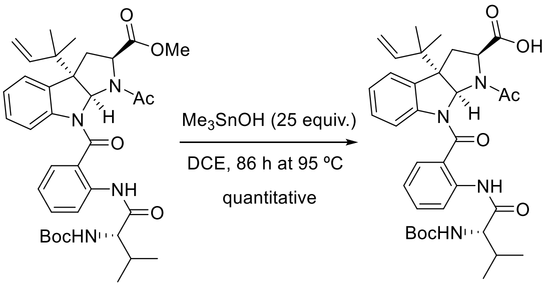

(*S*)-*endo*-**36** (*S*)-*endo*-**39**

Following the general procedure described above for the hydrolysis of methyl esters, the reaction of (*S*)-*endo*-**36** (0.02 g, 0.03 mmol) with trimethyltin hydroxide (0.14 g, 0.78 mmol) in DCE (0.5 mL) at 95 ºC (metal heating block) for 86 h afforded, after purification by flash column chromatography (silica gel 95:5 *v/v* CH_2Cl_2/MeOH), 0.02 g (quantitative yield) of the titled compound as a highly hygroscopic white solid.

(2*S*,3a*S*,8a*S*,2’*S*)-1-Acetyl-8-[2’-(2”-amino-3’’-methylbutanamido)benzoyl]-3a-(3’’’-methylbut-1-en-3’’’-yl)-(1,2,3,3a,8,8a)-hexahydropyrrolo[2,3-*b*]indole-2-carboxylic Acid ((*S*)-*endo*-40)

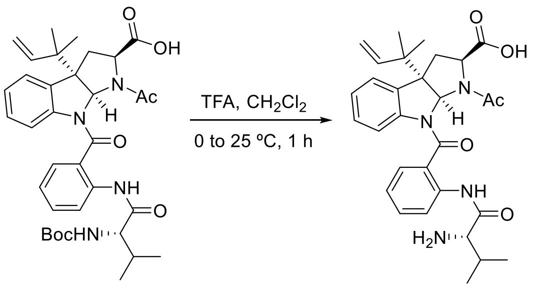

(*S*)-*endo*-**39** (*S*)-*endo*-**40**

Following the general procedure described above for the deprotection of the *N*-Boc protected amines, the reaction of (*S*)-*endo*-**39** (0.01 g, 15.8 μmol), TFA (0.25 mL, 0.37 g, 3.28 mmol) in CH_2Cl_2 (1.8 mL) at room temperature for 1 h afforded a residue that was used in the next step without further purification.

New synthetic novofumigatamide from L-Trp and L-Val (L-Trp-regio-*endo*-4)

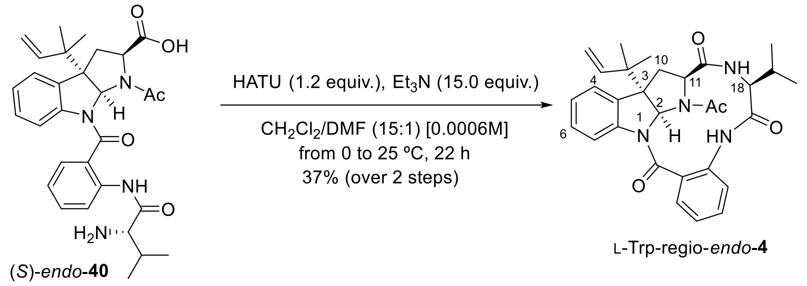

The general procedure for the macrolactamization (**Method B**) was used. To a solution of tryptophan derivative (*S*)-*endo*-**40** (8.0 mg, 15.8 μmol) in CH_2Cl_2 (9 mL) and DMF (1 mL), HATU (7.0 mg, 19.0 μmol) was added, and the resulting mixture was stirred at 0 ºC for 20 min. The solution of the activated acid was added to a solution of Et_3N (33.0 μL, 24.0 mg, 0.24 mmol) in CH_2Cl_2 (16 mL) via syringe pump over a period of 7 h and at a flow rate of 1.4 mL/h. The mixture was further stirred for 15 h while the temperature was raised from 0 ºC to room temperature, H_2O was added to the reaction mixture, the layers were separated, and the aqueous layer was extracted with CH_2Cl_2 (3x). The combined organic layers were washed with H_2O (6x), dried (anhydrous Na_2SO_4) and the solvent was evaporated. If DMF remained in the residue, it was dissolved again in EtOAc, washed several times with H_2O, dried (anhydrous Na_2SO_4) and the solvent was concentrated. The residue was purified by flash column chromatography (silica gel, 60:40 *v/v* hexane/EtOAc) to afford 3.0 mg (37% yield, over two steps) of the titled compound as a white solid. **^{1}H NMR** (400.16 MHz, $CDCl_3$, 298 K) δ 8.08 (br s, 1H, NH), 7.71 (d, *J* = 8.1 Hz, 1H, ArH), 7.62 – 7.48 (m, 2H, ArH + NH), 7.49 – 7.34 (m, 3H, ArH), 7.02 (t, *J* = 7.6 Hz, 1H, ArH), 6.86 (ddd, *J* = 8.5, 7.4, 1.2 Hz, 1H, ArH), 6.26 (s, 1H, H_2), 6.12-5.92 (m, 1H, $C(CH_3)_2C\underline{H}=CH_2$), 5.58 (d, *J* = 8.4 Hz, 1H, ArH), 5.32 – 5.13 (m, 3H, $C(CH_3)_2CH=C\underline{H_2}$ + H_{11}), 3.18 (dd, *J* = 10.5, 6.7 Hz, 1H, H_{18}), 2.86 – 2.72 (m, 1H, $C\underline{H}(CH_3)_2$), 2.68 – 2.53 (m, 4H, $COC\underline{H_3}$ + H_{10A}), 2.48 – 2.38 (m, 1H, H_{10B}), 1.21 (s, 3H, $C(C\underline{H_3})_2CH=CH_2$), 1.07 (s, 3H, $C(C\underline{H_3})_2CH=CH_2$), 1.03 (d, *J* = 6.8 Hz, 3H, $CH(C\underline{H_3})_2$), 0.84 (d, *J* = 6.6 Hz, 3H, $CH(C\underline{H_3})_2$) ppm. **^{13}C{^{1}H} NMR** (100.62 MHz, $CDCl_3$, 298 K) δ 172.9 (s), 172.0 (s), 170.5 (s), 167.6 (s), 143.2 (d), 140.2 (s), 135.4 (s), 133.1 (s), 132.7 (s), 131.4 (d), 128.6 (d), 127.4 (d), 127.3 (d, 2x), 125.2 (d), 124.6 (d), 115.3 (t), 111.5 (d), 80.7 (d, C_2), 66.3 (d), 62.0 (s), 60.5 (d), 42.5 (s), 35.3 (t), 27.5 (d, $\underline{C}H(CH_3)_2$), 23.6 (q, $C(\underline{C}H_3)_2CH=CH_2$), 23.2 (q, $C(\underline{C}H_3)_2CH=CH_2$), 21.8 (q, $CO\underline{C}H_3$), 20.8 (q, $CH(C\underline{H}_3)_2$), 20.5 (q, $CH(C\underline{H}_3)_2$) ppm. **HRMS** (ESI-TOF) *m/z*: $[M + H]^+$ calcd.

for $C_{30}H_{35}N_4O_4$, 515.2653; found, 515.2650. **IR** (NaCl): ν 3500-3100 (br, N-H), 2959 (m, C-H), 2924 (s, C-H), 2852 (m, C-H), 1713 (m, C=O), 1693 (m, C=O), 1648 (s, C=O), 1591 (w), 1582 (w), 1519 (m), 1484 (m), 1452 (m), 1401 (m), 1368 (m), 1336 (w), 1323 (w), 1297 (w), 1243 (w), 1204 (w), 1362 (w), 1166 (w), 1150 (w), 1088 (w), 1039 (w), 1008 (w), 923 (w), 907 (w), 751(m) cm^{-1}. $[\alpha]_D^{24}$ –15.3 (*c* 0.1, $CHCl_3$).

Methyl (2-Aminobenzoyl)-L-valinate 60

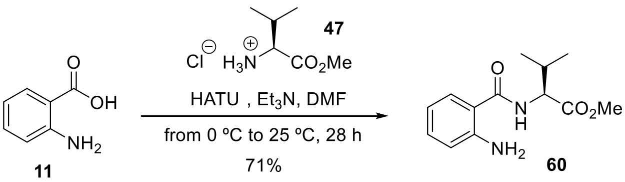

Following the general procedure described above for amide (peptide) bond formation (**Method B**), the reaction of L-valine methyl ester hydrochloride **47** (1.0 g, 6.0 mmol), anthranilic acid **11** (0.82 g, 6.0 mmol), HATU (3.4 g, 8.9 mmol) and Et_3N (2.1 mL, 1.51 g, 14.9 mmol) in DMF (60 mL) at 25 ºC for 28 h afforded, after purification by flash column chromatography (CombiFlash® Rf+ system, 80 g silica gel, gradient from 90:10 to 50:50 *v/v* hexane/EtOAc, flow rate = 60 mL/min), 1.06 g (71% yield) of the titled compound **60** as a white solid. The spectroscopic data matched those previously reported.[10]

^{1}H NMR (400.16 MHz, $CDCl_3$) δ 7.52 – 7.43 (m, 1H, ArH), 7.35 – 7.27 (m, 1H, ArH), 6.99 – 6.78 (m, 2H, ArH), 6.71 – 6.57 (m, 1H, CONH), 4.72 (dd, *J* = 8.5, 4.9 Hz, 1H, $H_{2'}$), 3.78 (s, 3H, $CO_2C\underline{H_3}$), 2.32 – 2.21 (m, 1H, $C\underline{H}(CH_3)_2$), 1.01 (d, *J* = 6.9 Hz, 3H, $CH(C\underline{H_3})_2$), 0.99 (d, *J* = 6.9 Hz, 3H, $CH(C\underline{H_3})_2$) ppm.

Methyl (2*S*,2’’*S*)-2-(2’-{[3’’-(1’’’-Acetylindole-3’’’-yl)-2’’-(*tert*-butoxycarbonyl amino)propanamido]benzoyl}amino)-3-methylbutanoate 58

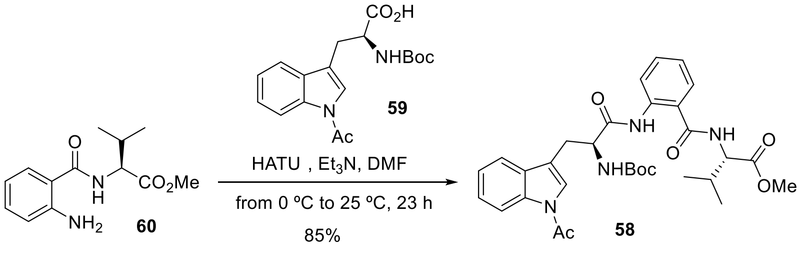

Following the general procedure described above for amide (peptide) bond formation (**Method B**), the reaction of anthranilic derivative **60** (0.92 g, 3.67 mmol), tryptophan derivative **59** (1.27 g, 3.67 mmol), HATU (2.09 g, 5.51 mmol) and Et_3N (0.77 mL, 0.56 g, 5.51 mmol) in DMF (50 mL) at 25 ºC for 23 h afforded, after purification by flash column chromatography (CombiFlash® Rf+ system, 120 g silica gel, gradient from 90:10 to 50:50 *v/v* hexane/EtOAc, flow rate = 85 mL/min), 1.8 g (85% yield) of the titled compound **58** as a white solid. **^{1}H NMR** (400.16 MHz, DMSO-d_6, 343 K) δ 8.68 (d, *J* = 7.2 Hz, 1H, NH), 8.50 (d, *J* = 8.3 Hz, 1H, ArH), 8.29 (d, *J* = 7.9 Hz, 1H, ArH), 7.87 (d, *J* = 6.9 Hz, 1H, ArH), 7.69 – 7.61 (m, 2H, 2 x ArH), 7.57

– 7.48 (m, 1H, ArH), 7.37 – 7.22 (m, 3H, 2 x ArH + NH), 7.23 – 7.14 (m, 1H, ArH), 4.45 – 4.26 (m, 2H, H_2 + $H_{2''}$), 3.68 (s, 2H, CO_2CH_3), 3.64 (s, 1H, CO_2CH_3, rotamer), 3.35 – 3.26 (m, 1H, $H_{3A''}$), 3.03 (dd, J = 14.9, 10.0 Hz, 1H, $H_{3B''}$), 2.59 (s, 3H, $COCH_3$), 2.28 – 2.14 (m, 1H, C<u>H</u>$(CH_3)_2$), 1.33 (s, 9H, $C(CH_3)_3$), 1.01 – 0.93 (m, 6H, CH(C<u>H$_3$</u>)$_2$) ppm. **$^{13}C\{^1H\}$ NMR** (100.62 MHz, DMSO-d_6, **343 K**) δ 171.3 (s, two peaks, rotamers), 170.4 (s), 168.4 (s), 168.2 (s), 155.2 (s), 138.4 (s), 134.9 (s), 131.8 (d), 130.1 (s), 128.5 (d), 124.4 (d, 2x), 122.9 (d), 122.4 (d), 120.3 (s), 120.0 (d, two peaks, rotamers), 118.6 (d), 117.6 (s), 115.6 (d), 78.2 (s), 58.2 (d, rotamer: 58.0), 55.6 (d), 51.4 (q, rotamer: 51.3), 29.4 (d, rotamer: 29.3), 27.8 (q, 3x), 26.4 (t), 23.3 (q), 18.8 (q, two peaks, rotamers), 18.6 (q, two peaks, rotamers) ppm. **HRMS** (ESI-TOF) *m/z*: $[M + H]^+$ calcd. for $C_{31}H_{38}N_4NaO_7$, 601.2633; found, 601.2635. **IR** (NaCl): ν 3500-3100 (br, N-H), 3012 (w, C-H), 2970 (w, C-H), 2932 (w, C-H), 1698 (s, C=O), 1650 (m, C=O), 1601 (m, C=O), 1587 (m), 1520 (s), 1451 (s), 1389 (m), 1370 (m), 1331 (m), 1250 (m), 1222 (m), 1168 (m), 753 (s) cm^{-1}. **$[\alpha]_D^{23}$** + 8.3 (*c* 0.2, $CHCl_3$).

Bromine-containing precursor of regioisomer L-Trp-regio-56 (*exo*-61 and *endo*-61)

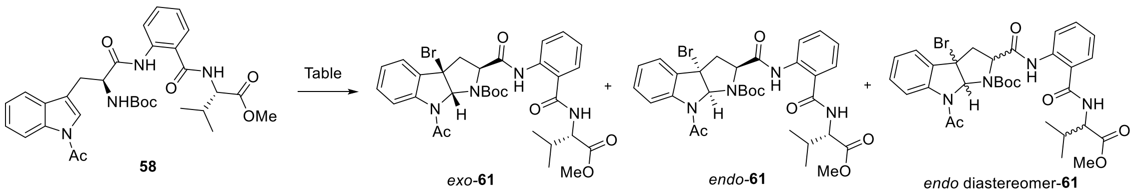

Entry	Reaction conditions	Yield
**1**	NBS (1.1 equiv.), CH_2Cl_2, 25 ºC, 3 h	59% (1:3 *endo*/*exo*)
**2**	NBS (2 equiv. added portionwise), CH_3CN, 25 ºC, 48 h	68% (2.2:1 *endo*/*exo*)
**3**	NBS (1.3 equiv.), CH_2Cl_2, -30 ºC, 8 h	65% (1:12 *endo*/*exo*)
**4**	NBS (2 equiv. added portionwise), CH_3CN, -30 ºC, 22 h	64% (2.5:1:2.5 *endo*/*endo* diast./*exo*)

Experimental procedure for entry 4. Following the general procedure described above for the bromocyclization with *N*-bromosuccinimide (**Method A**), the reaction of ester **58** (0.1 g, 0.17 mmol) and NBS (0.06 g, 0.35 mmol) in CH_3CN (17.3 mL) at - 30 ºC for 22 h afforded, after purification by flash column chromatography (CombiFlash® Rf+ system, 24 g silica gel, gradient from 90:10 to 70:30 *v/v* hexane/EtOAc, flow rate = 35 mL/min), 29.1 mg of *exo*-**61** (26% yield), 30.7 mg of the *endo* isomer *endo*-**61** (27% yield), and 13.1 mg of another *endo* diastereomer **61** (11% yield), the three of them as beige solids.

Exo-**61**. **^{1}H NMR** (400.16 MHz, DMSO-d_6, 343 K) δ 8.76 (d, J = 7.6 Hz, 1H, NH), 8.35 – 8.27 (m, 1H, ArH), 7.89 – 7.81 (m, 2H, ArH), 7.73 – 7.65 (m, 1H, ArH), 7.59 – 7.50 (m, 1H, ArH), 7.46 – 7.33 (m, 1H, ArH), 7.30 – 7.17 (m, 2H, 2 x ArH), 6.28 (s, 0.3H, $H_{8a''}$), 6.27 (s, 0.7H, $H_{8a''}$), 4.39 – 4.28 (m, 1H, H_2), 3.84 (dd, J = 9.5, 7.0 Hz, 1H, $H_{2''}$), 3.71 – 3.60 (m, 4H, CO_2CH_3

+ $H_{3A''}$), 2.82 – 2.70 (m, 1H, $H_{3B''}$), 2.57 (s, 3H, $COCH_3$), 2.31 – 2.15 (m, 1H, C<u>H</u>($CH_3)_2$), 1.20 (s, 9H, $C(CH_3)_3$), 1.05 – 0.93 (m, 6H, CH(C<u>H</u>$_3)_2$) ppm. **^{13}C{^{1}H} NMR** (100.62 MHz, DMSO-d_6, 343K) δ 171.4 (s, rotamer: 171.3), 169.5 (s), 168.7 (s, rotamer: 168.6), 168.4 (s, rotamer: 168.3), 151.4 (s), 140.4 (s), 138.0 (s, rotamer: 137.8), 132.7 (s), 131.9 (d, rotamer: 131.8), 130.4 (d), 128.6 (d, rotamer: 128.5), 124.9 (d), 123.8 (d, two peaks, rotamers), 122.9 (d), 121.4 (s, rotamer: 121.2), 120.3 (d), 118.3 (d, two peaks, rotamers), 84.4 (d), 81.0 (s), 62.1 (d), 61.0 (s), 58.3 (d), 51.4 (q), 40.4 (t), 29.3 (d, rotamer: 29.2), 27.3 (q), 23.2 (q), 18.8 (q, rotamers: 18.7, 18.6) ppm. **HRMS** (ESI-TOF) *m/z*: $[M + Na]^+$ calcd. for $C_{31}H_{37}{}^{79}BrN_4NaO_7$, 679.1738; found, 679.1742. **IR** (NaCl): ν 3500-3100 (br, N-H), 3013 (w, C-H), 2971 (w, C-H), 2933 (w, C-H), 1741 (m, C=O), 1716 (s, C=O), 1686 (s, C=O), 1646 (m, C=O), 1600 (m, C=O), 1589 (m), 1523 (s), 1449 (s), 1368 (s), 1320 (s), 1284 (s), 1210 (m), 1167 (s), 1137 (s), 754 (s) cm^{-1}. $[\alpha]_D^{24}$ –70.5 (*c* 0.22, $CHCl_3$).

Endo-**61**. **^{1}H NMR** (400.16 MHz, DMSO-d_6, 343 K) δ 8.42 (d, *J* = 8.0 Hz, 1H, NH), 8.08 (dd, *J* = 8.4, 1.2 Hz, 1H, ArH), 8.02 – 7.80 (m, 1H, ArH), 7.47 – 7.36 (m, 2H, ArH), 7.31 (ddd, *J* = 8.6, 7.3, 1.5 Hz, 1H, ArH), 7.04 (ddd, *J* = 7.9, 7.3, 1.3 Hz, 1H, ArH), 6.89 – 6.82 (m, 2H, 2 x ArH), 6.41 (s, 1H, $H_{8a''}$), 4.54 (d, *J* = 9.0 Hz, 1H, $H_{2''}$), 4.47 (dd, *J* = 8.0, 6.4 Hz, 1H, H_2), 3.79 (s, 3H, CO_2CH_3), 3.41 (d, *J* = 12.7 Hz, 1H, $H_{3A''}$), 3.20 (dd, *J* = 12.8, 9.5 Hz, 1H, $H_{3B''}$), 2.54 (s, 3H, $COCH_3$), 2.28 – 2.16 (m, 1H, C<u>H</u>($CH_3)_2$), 1.43 (s, 9H, $C(CH_3)_3$), 0.95 (d, *J* = 6.8 Hz, 3H, CH(C<u>H</u>$_3)_2$), 0.92 (d, *J* = 6.8 Hz, 3H, CH(C<u>H</u>$_3)_2$) ppm. **^{13}C{^{1}H} NMR** (100.62 MHz, DMSO-d_6, 343K) δ 171.4 (s), 168.9 (s), 168.0 (s), 167.5 (s), 153.3 (s), 141.2 (s), 138.5 (s), 132.5 (s), 131.5 (d), 129.4 (d), 128.0 (d), 124.2 (d), 123.7 (d), 121.9 (d), 118.7 (d), 118.4 (d), 118.4 (s), 85.3 (d), 81.3 (s), 62.6 (d), 61.9 (s), 57.4 (d), 51.5 (q), 42.1 (t), 29.7 (d), 27.6 (q), 23.0 (q), 18.7 (q), 18.4 (q) ppm. **HRMS** (ESI-TOF) *m/z*: $[M + Na]^+$ calcd. for $C_{31}H_{37}{}^{79}BrN_4NaO_7$, 679.1738; found, 679.1740. **IR** (NaCl): ν 3500-3000 (br, N-H), 3007 (w, C-H), 2966 (m, C-H), 2933 (m, C-H), 2874 (w, C-H), 1720 (s, C=O), 1681 (s, C=O), 1600 (m, C=O), 1587 (m), 1520 (s), 1394 (s), 1368 (s), 1340 (s), 1312 (s), 1286 (m), 1254 (m), 1214 (m), 1159 (s), 754 (s) cm^{-1}. $[\alpha]_D^{24}$ +137.8 (*c* 0.21, $CHCl_3$).

Endo diastereomer-**61**. **^{1}H NMR** (400.16 MHz, DMSO-d_6, 343 K) δ 8.45 (d, *J* = 7.2 Hz, 1H, NH), 8.07 (dd, *J* = 8.5, 1.2 Hz, 1H, ArH), 7.83 (dd, *J* = 8.0, 1.6 Hz, 1H, ArH), 7.53 – 7.44 (m, 1H, ArH), 7.43 – 7.36 (m, 1H, ArH), 7.30 (ddd, *J* = 8.6, 7.3, 1.5 Hz, 1H, ArH), 7.07 – 7.00 (m, 1H, ArH), 6.87 – 6.81 (m, 2H, 2 x ArH), 6.41 (s, 1H, $H_{8a''}$), 4.56 (d, *J* = 8.9 Hz, 1H, $H_{2''}$), 4.40 (app. t, *J* = 6.9 Hz, 1H, H_2), 3.63 (s, 3H, CO_2CH_3), 3.42 (d, *J* = 12.8 Hz, 1H, $H_{3A''}$), 3.19 (dd, *J* = 12.7, 9.4 Hz, 1H, $H_{3B''}$), 2.59 (s, 3H, $COCH_3$), 2.32 – 2.16 (m, 1H, C<u>H</u>($CH_3)_2$), 1.41 (s, 9H, $C(CH_3)_3$), 1.09 (d, *J* = 6.8 Hz, 3H, CH(C<u>H</u>$_3)_2$), 1.03 (d, *J* = 6.8 Hz, 3H, CH(C<u>H</u>$_3)_2$) ppm. **^{113}C{^{1}H} NMR** (100.62 MHz, DMSO-d_6, 343K) δ 171.5 (s), 169.1 (s), 167.9 (s), 167.8 (s), 153.2 (s), 141.2 (s), 138.5 (s), 132.3 (s), 131.5 (d), 129.4 (d), 128.1 (d), 124.2 (d), 123.8 (d),

121.8 (d), 118.6 (d), 118.3 (s), 118.1 (d), 85.4 (d), 81.4 (s), 62.7 (d), 62.0 (s), 58.0 (d), 51.1(q), 42.2 (t), 29.3 (d), 27.5 (q), 23.1 (q), 18.7 (q, 2x) ppm. **HRMS** (ESI-TOF) *m/z*: $[M + Na]^+$ calcd. for $C_{31}H_{37}{}^{79}BrN_4NaO_7$, 679.1738; found, 679.1740. **IR** (NaCl): ν 3400-3100 (br, N-H), 3008 (w, C-H), 2968 (m, C-H), 2932 (w, C-H), 1718 (s, C=O), 1680 (s, C=O), 1600 (m, C=O), 1587 (m), 1522 (s), 1449 (s), 1394 (s), 1369 (s), 1340 (s), 1312 (s), 1286 (m), 1254 (m), 1208 (m), 1160 (s), 754 (s) cm^{-1}. $[\alpha]_D^{22}$ –49.2 (*c* 0.2, $CHCl_3$).

Exo **reverse-prenylated precursor of regioisomer L-Trp-regio-56 (*exo*-57)**

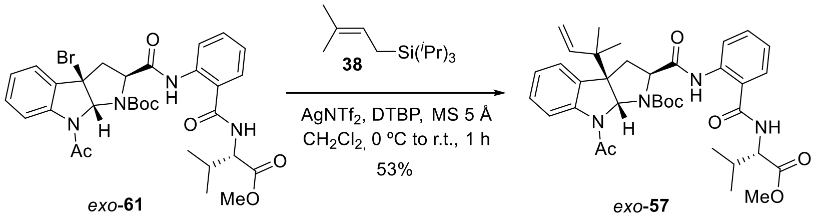

Following the general procedure described above for the reverse prenylation with triisopropyl(3-methyl-2-butenyl)silane (**Method B**), the reaction of *exo*-bromopyrroloindoline *exo*-**61** (0.2 g, 0.3 mmol), prenyl triisopropyl silane (0.1 g, 0.46 mmol), freshly activated 5Å MS (1.78 g), DTBP (0.1 mL, 0.09 g, 0.46 mmol) and $AgNTf_2$ (0.2 g, 0.46 mmol) in CH_2Cl_2 (6 mL) at room temperature for 1 h afforded, after purification by flash column chromatography (CombiFlash® Rf+ system, 24 g silica gel, gradient from 90:10 to 70:30 *v/v* hexane/EtOAc, flow rate = 35 mL/min), the desired compound *exo*-**57** (104.0 mg, 53% yield) as a white solid. **^{1}H NMR** (400.16 MHz, DMSO-d_6, 343 K) δ 8.76 (d, *J* = 7.7 Hz, 1H, NH), 8.44 – 8.35 (m, 1H, ArH), 7.90 – 7.75 (m, 2H, 2 x ArH), 7.58 – 7.49 (m, 1H, ArH), 7.45 – 7.35 (m, 1H, ArH), 7.32 (td, *J* = 7.8, 1.2 Hz, 1H, ArH), 7.18 (td, *J* = 7.6, 1.0 Hz, 2H, 2 x ArH), 5.98 (s, 1H, $H_{8a''}$), 5.88 – 5.75 (m, 1H, $C(CH_3)_2C\underline{H}=CH_2$), 5.09 – 4.98 (m, 2H, $C(CH_3)_2CH=C\underline{H_2}$), 4.38 (t, *J* = 7.4 Hz, 0.75H, 0.75H_2), 4.33 (t, *J* = 7.4 Hz, 0.25H, 0.25H_2), 3.69 (s, 0.7H, CO_2CH_3), 3.70 – 3.60 (m, 3.3H, CO_2CH_3 + $H_{2''}$), 2.67 (dd, *J* = 13.0, 7.2 Hz, 1H, $H_{3A''}$), 2.54 – 2.45 (m, 3H, $COCH_3$), 2.40 – 2.29 (m, 1H, $H_{3B''}$), 2.29 – 2.17 (m, 1H, $C\underline{H}(CH_3)_2$), 1.17 (s, 9H, $C(CH_3)_3$), 1.06 (s, 3H, $C(C\underline{H_3})_2CH=CH_2$, rotamer: 1.05), 1.02 – 0.94 (m, 6H, $CH(C\underline{H_3})_2$), 0.9 (s, 3H, $C(C\underline{H_3})_2CH=CH_2$, rotamer: 0.89) ppm. **^{13}C{^{1}H} NMR** (100.62 MHz, DMSO-d_6, 343K) δ 171.4 (s, rotamer: 171.3), 170.1 (s, two peaks, rotamers), 168.9 (s), 168.4 (s, two peaks, rotamers), 151.9 (s), 143.2 (d), 141.9 (s, two peaks, rotamers), 138.5 (s, rotamer: 138.4), 133.4 (s), 131.9 (d, two peaks, rotamers), 128.6 (d, rotamer: 128.5), 128.2 (d), 124.7 (d), 123.6 (d), 122.4 (d), 120.3 (s, rotamer: 120.2), 119.7 (d), 118.0 (d), 113.5 (t), 80.2 (s), 79.6 (d), 62.1 (d), 60.9 (s), 58.4 (d, rotamer: 58.1), 51.4 (q, two peaks, rotamers), 39.9 (s, HMBC), 34.0 (t), 29.3 (d, rotamer: 29.2), 27.4 (q, 3x), 23.3 (q), 22.6 (q), 21.7 (q, two peaks, rotamers), 18.8 (q, rotamer: 18.73), 18.68 (q, rotamer: 18.64) ppm. **HRMS** (ESI-TOF) *m/z*: $[M + H]^+$ calcd. for $C_{36}H_{47}N_4O_7$, 647.3439; found, 647.3442. **IR** (NaCl): ν 3500-3100 (br, N-H), 3009 (m, C-H), 2974 (m, C-H), 2933 (m, C-H), 1743 (s, C=O), 1714 (s, C=O), 1676 (s, C=O), 1651 (s, C=O), 1598 (s), 1589 (s), 1522 (s), 1478

(s), 1449 (s), 1405 (s), 1367 (s), 1328 (s), 1285 (s), 1212 (s), 1170 (s), 1151 (s), 928 (m), 755 (s) cm^{-1}. $[\alpha]_D^{24}$ –51.2 (*c* 0.17, $CHCl_3$).

***Exo* reverse-prenylated precursor of regioisomer L-Trp-regio-56 (*exo*-62)**

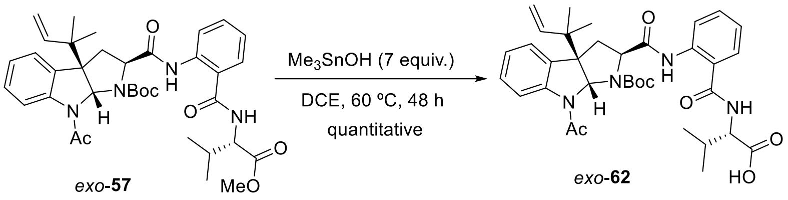

Following the general procedure described above for the hydrolysis of esters, the reaction of *exo*-**57** (0.06 g, 92.8 μmol) with trimethyltin hydroxide (0.12 g, 0.65 mmol) in DCE (1 mL) at 60 ºC (metal heating block) for 48 h afforded, after purification by flash column chromatography (silica gel, gradient from 95:5 to 90:10 *v/v* CH_2Cl_2/MeOH), 58.4 mg (quantitative yield) of the titled compound as a white solid. **^{1}H NMR** (400.16 MHz, DMSO-d_6, 343 K) δ 8.56 (d, *J* = 7.5 Hz, 1H NH), 8.43 – 8.36 (m, 1H, ArH), 7.90 – 7.74 (m, 2H, 2 x ArH), 7.57 – 7.48 (m, 1H, ArH), 7.46 – 7.38 (m, 1H, ArH), 7.32 (td, *J* = 7.9, 1.2 Hz, 1H, ArH), 7.22 – 7.13 (m, 2H, 2 x ArH), 5.98 (s, 1H, $H_{8a''}$), 5.81 (dd, *J* = 17.2, 10.9 Hz, 1H, $C(CH_3)_2C\underline{H}=CH_2$), 5.09 – 4.99 (m, 2H, $C(CH_3)_2CH=C\underline{H_2}$), 4.34 (dd, *J* = 7.8, 6.6 Hz, 1H, H_2), 3.65 (dd, *J* = 9.2, 7.4 Hz, 1H, $H_{2''}$), 2.68 (dd, *J* = 13.0, 7.3 Hz, 1H, $H_{3A''}$), 2.53 – 2.44 (br s, 3H, $COCH_3$), 2.33 (dd, *J* = 13.0, 9.7 Hz, 1H, $H_{3B''}$), 2.28 – 2.16 (m, 1H, $C\underline{H}(CH_3)_2$), 1.18 (s, 9H, $C(CH_3)_3$), 1.05 (s, 3H, $C(C\underline{H_3})_2CH=CH_2$), 1.00 (d, *J* = 6.8 Hz, 3H, $CH(C\underline{H_3})_2$), 0.99 (d, *J* = 6.8 Hz, 3H, $CH(C\underline{H_3})_2$), 0.89 (s, 3H, $C(C\underline{H_3})_2CH=CH_2$) ppm. **^{13}C{^{1}H} NMR** (100.62 MHz, DMSO-d_6, 343K) δ 172.1 (s), 170.1 (s), 168.9 (s), 168.3 (s), 151.9 (s), 143.2 (d), 141.9 (s), 138.5 (s, rotamer: 138.4), 133.4 (s), 131.8 (d, rotamer: 131.7), 128.5 (d, rotamer: 128.4), 128.2 (d), 124.7 (d), 123.7 (d), 122.4 (d, two peaks, rotamers), 120.4 (s), 119.6 (d), 118.0 (d), 113.5 (t), 80.3 (s), 79.6 (d), 62.1 (d, rotamer: 62.0), 61.0 (s), 58.0 (d), 39.8 (s, HMBC), 34.0 (t), 29.3 (d), 27.4 (q, 3x), 23.4 (q), 22.7 (q), 21.7 (q), 18.9 (q, two peaks, rotamers), 18.5 (q, rotamer: 18.4) ppm. **HRMS** (ESI-TOF) *m/z*: $[M + H]^+$ calcd. for $C_{35}H_{45}N_4O_7$, 633.3283; found, 633.3282. **IR** (NaCl): ν 3500-3000 (br, O-H, N-H), 3010 (m, C-H), 2971 (m, C-H), 2928 (m, C-H), 2875 (m, C-H), 2852 (m, C-H), 1715 (s, C=O), 1646 (m, C=O), 1589 (m), 1522 (s), 1479 (m), 1449 (m), 1412 (m), 1367 (m), 1329 (m), 1287 (m), 1151 (m), 928 (w), 755 (s) cm^{-1}. $[\alpha]_D^{24}$ –42.4 (*c* 0.29, $CHCl_3$).

Synthetic novofumigatamide from L-Trp (L-Trp-regio-*exo*-56)

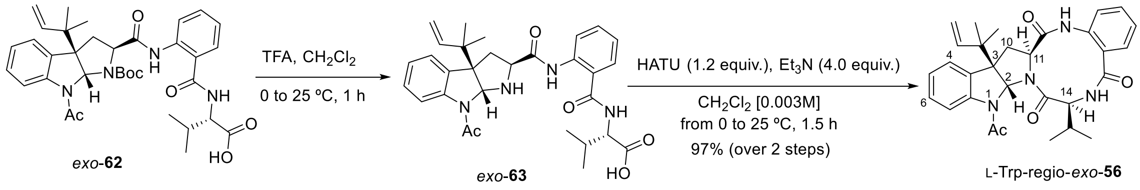

Following the general procedure described above for the deprotection of the *N*-Boc protected amines, the reaction of *exo*-**62** (0.02 g, 31.6 μmol) with TFA (0.06 mL, 0.09 g, 0.78 mmol) in

CH_2Cl_2 (0.3 mL) at room temperature for 1 h afforded a residue that was used in the next step without further purification. The reaction progress was monitored by HPLC-MS (Scharlau Kromaphase 100, C18, 5 μm, 250 x 4.6 mm, gradient from 50% CH_3CN/H_2O to 100% CH_3CN in 20 min, 1.0 mL/min).

Following the general procedure described above for the macrolactamization (Method A), the reaction of *exo*-**63** (17 mg, 32 μmol), Et_3N (17.8 μL, 13.0 mg, 0.13 mmol) and HATU (15.0 mg, 38.0 μmol) in CH_2Cl_2 (10.7 mL) at 25 ºC for 1.5 h afforded, after purification of the residue by flash column chromatography (silica gel, 97:3 *v/v* CH_2Cl_2/MeOH), a complex mixture of products. The reaction progress was monitored by HPLC-MS (Scharlau Kromaphase 100, C18, 5 μm, 250 x 4.6 mm, gradient from 50% CH_3CN/H_2O to 100% CH_3CN in 20 min, 1.0 mL/min).

***Endo* reverse-prenylated precursor of regioisomer L-Trp-regio-56 (*endo*-57)**

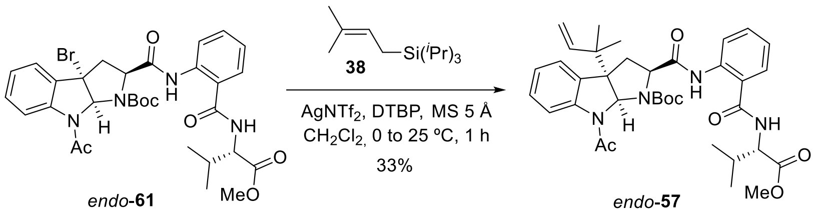

Following the general procedure described above for the reverse prenylation with triisopropyl(3-methyl-2-butenyl)silane (**Method B**), the reaction of *exo*-bromopyrroloindoline *endo*-**61** (0.19 g, 0.29 mmol), prenyl triisopropyl silane (0.1 g, 0.43 mmol), freshly activated 5Å MS (1.66 g), DTBP (0.1 mL, 0.08 g, 0.43 mmol) and $AgNTf_2$ (0.18 g, 0.43 mmol) in CH_2Cl_2 (5.7 mL) at room temperature for 1 h afforded, after purification by flash column chromatography (CombiFlash® Rf+ system, 24 g silica gel, gradient from 90:10 to 70:30 *v/v* hexane/EtOAc, flow rate = 35 mL/min), the desired compound *endo*-**57** (61.6 mg, 33% yield) as a white solid. **^{1}H NMR** (400.16 MHz, DMSO-d_6, 343 K) δ 8.40 (d, J = 8.0 Hz, 1H, NH), 7.99 (d, J = 8.0 Hz, 1H, ArH), 7.82 (dd, J = 7.9, 1.4 Hz, 1H, ArH), 7.43 – 7.33 (m, 1H, ArH), 7.32 – 7.24 (m, 1H, ArH), 7.18 (dd, J = 7.5, 1.1 Hz, 1H, ArH), 7.07 – 6.99 (m, 1H, ArH), 6.80 (td, J = 7.4, 1.3 Hz, 1H, ArH), 6.74 (td, J = 7.7, 1.4 Hz, 1H, ArH), 6.10 (s, 1H, $H_{8a''}$), 5.88 (dd, J = 17.6, 10.6 Hz, 1H, $C(CH_3)_2C\underline{H}=CH_2$), 5.11 – 5.04 (m, 2H, $C(CH_3)_2CH=C\underline{H_2}$), 4.60 – 4.53 (m, 1H, $H_{2''}$), 4.48 (dd, J = 8.0, 6.4 Hz, 1H, H_2), 3.77 (s, 3H, CO_2CH_3), 2.63 – 2.56 (m, 2H, $2H_3$), 2.46 (s, 3H, $COCH_3$), 2.28 – 2.15 (m, 1H, $C\underline{H}(CH_3)_2$), 1.44 (s, 9H, $C(CH_3)_3$), 1.06 (s, 3H, $C(C\underline{H_3})_2CH=CH_2$), 0.94 (d, J = 6.8 Hz, 3H, $CH(C\underline{H_3})_2$), 0.92 (d, J = 6.8 Hz, 3H, $CH(C\underline{H_3})_2$), 0.90 (s, 3H, $C(C\underline{H_3})_2CH=CH_2$) ppm. **^{13}C{^{1}H} NMR** (100.62 MHz, DMSO-d_6, 343K) δ 171.4 (s), 168.8 (s), 168.5 (s), 167.6 (s), 153.6 (s), 143.5 (d), 142.8 (s), 138.6 (s), 132.5 (s), 131.4 (d), 128.0 (d), 127.3 (d), 125.0 (d), 122.9 (d), 121.6 (d), 118.9 (d), 118.7 (s), 117.5 (d), 113.6 (t), 80.8 (s), 80.0 (d), 62.8 (d), 61.8 (s), 57.4 (d), 51.4 (q), 40.1 (s), 34.8 (t), 29.8 (d), 27.7 (q, 3x), 23.4 (q), 22.5 (q), 21.9 (q), 18.8 (q), 18.3 (q) ppm. **HRMS** (ESI-TOF) *m/z*: $[M + H]^+$ calcd. for

$C_{36}H_{47}N_4O_7$, 647.3455; found, 647.3449. **IR** (NaCl): ν 3500-3100 (br, N-H), 3006 (m, C-H), 2968 (m, C-H), 2933 (m, C-H), 2875 (m, C-H), 1738 (m, C=O), 1714 (s, C=O), 1673 (s, C=O), 1586 (m), 1520 (s), 1449 (s), 1395 (m), 1368 (m), 1341 (m), 1312 (m), 1258 (m), 1211 (m), 1169 (m), 1157 (m), 754 (m) cm^{-1}. $[\alpha]_D^{24}$ +80.5 (*c* 0.16, $CHCl_3$).

***Endo* reverse-prenylated precursor of regioisomer L-Trp-regio-56 (*endo*-62)**

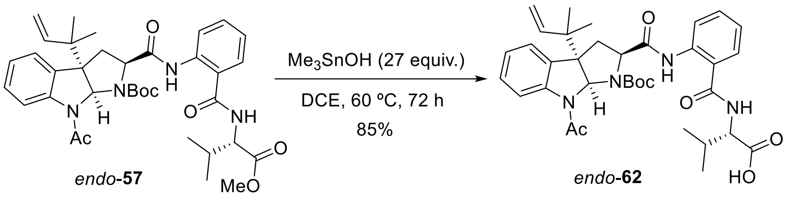

Following the general procedure described above for the hydrolysis of esters, the reaction of *endo*-**57** (0.04 g, 62.0 μmol) with trimethyltin hydroxide (0.3 g, 1.67 mmol) in DCE (1 mL) at 60 ºC (metal heating block) for 72 h afforded, after purification by flash column chromatography (silica gel, gradient from 97.5:2.5 to 95:5 *v/v* CH_2Cl_2/MeOH), 33.4 mg (85% yield) of the titled compound as a white solid.

REMARK: The reaction was followed by HPLC-MS. The starting material is very unreactive under these reaction conditions. It seems the reagent Me_3SnOH poisons the reaction, so after addition of 10 equiv. of it a workup was performed, and the reaction was set up again with the crude mixture. This process was repeated several times. **^{1}H NMR** (400.16 MHz, DMSO-d_6, 343 K) δ 8.06 – 7.84 (m, 2H, ArH + NH), 7.73 (d, *J* = 7.9 Hz, 1H, ArH), 7.39 (br s, 1H, ArH), 7.32 – 7.23 (m, 1H, ArH), 7.20 – 7.13 (m, 1H, ArH), 7.06 – 6.98 (m, 1H, ArH), 6.84 – 6.71 (m, 2H, 2 x ArH), 6.12 (s, 1H, $H_{8a''}$), 5.94 – 5.82 (m, 1H, $C(CH_3)_2C\underline{H}=CH_2$), 5.13 – 5.01 (m, 2H, $C(CH_3)_2CH=C\underline{H_2}$), 4.58 (d, *J* = 5.8 Hz, 1H, $H_{2''}$), 4.44 – 4.35 (m, 1H, H_2), 2.66 – 2.53 (m, 2H, $2H_3$), 2.46 (s, 3H, $COCH_3$), 2.28 – 2.13 (m, 1H, $C\underline{H}(CH_3)_2$), 1.43 (s, 9H, $C(CH_3)_3$), 1.05 (s, 3H, $C(C\underline{H_3})_2CH=CH_2$), 0.97 – 0.80 (m, 9H, $C(C\underline{H_3})_2CH=CH_2$ + $CH(C\underline{H_3})_2$) ppm. **^{13}C{^{1}H} NMR** (100.62 MHz, DMSO-d_6, 343K) δ 173.0 (s), 168.7 (s), 168.6 (s), 167.2 (s), 153.6 (s), 143.5 (d), 142.8 (s), 138.5 (s), 132.6 (s), 131.1 (d), 127.5 (d), 127.4 (d), 125.0 (d), 122.9 (d), 121.8 (d), 119.5 (s), 119.0 (d), 117.4 (d), 113.6 (t), 80.8 (s), 79.9 (d), 62.6 (d), 61.7 (s), 57.6 (d), 40.1 (s), 34.8 (t), 30.1 (d), 27.7 (q), 23.5 (q), 22.6 (q), 21.9 (q), 18.9 (q), 18.1 (q) ppm. **HRMS** (ESI-TOF) *m/z*: $[M + H]^+$ calcd. for $C_{35}H_{45}N_4O_7$, 633.3283; found, 633.3289. **IR** (NaCl): ν 3500-3000 (br, O-H, N-H), 2968 (m, C-H), 2931 (m, C-H), 1715 (m, C=O), 1676 (m, C=O), 1649 (m, C=O), 1588 (m), 1522 (s), 1478 (m), 1449 (m), 1416 (m), 1367 (m), 754 (m) cm^{-1}. $[\alpha]_D^{24}$ +26.8 (*c* 0.33, $CHCl_3$).

Synthetic novofumigatamide from L-Trp (L-Trp-regio-*endo*-56)

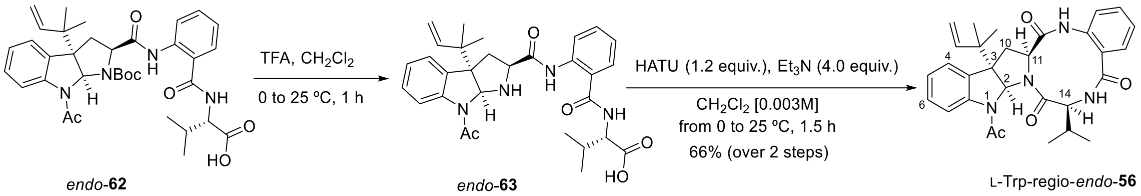

endo-**62** *endo*-**63** L-Trp-regio-*endo*-**56**

Following the general procedure described above for the deprotection of the *N*-Boc protected amines, the reaction of *endo*-**62** (15.0 mg, 24.0 μmol) with TFA (0.05 mL, 69.0 mg, 0.60 mmol) in CH_2Cl_2 (0.3 mL) at room temperature for 1 h afforded a residue that was used in the next step without further purification. The reaction progress was monitored by HPLC-MS (Scharlau Kromaphase 100, C18, 5 μm, 250 x 4.6 mm, gradient from 50% CH_3CN/H_2O to 100% CH_3CN in 20 min, 1.0 mL/min).

Following the general procedure described above for the macrolactamization (**Method A**), the reaction of *endo*-**63** (13.0 mg, 24.0 μmol), Et_3N (13.4 μL, 9.7 mg, 96.0 μmol) and HATU (11.0 mg, 29.0 μmol) in CH_2Cl_2 (8 mL) at 25 ºC for 1.5 h afforded, after purification of the residue by flash column chromatography (silica gel, 50:50 *v/v* hexane/EtOAc), 8.2 mg of an impure sample of the desired product L-Trp-regio-*endo*-**56**. The product gradually decomposed over time, so full characterization was not possible. The reaction progress was monitored by HPLC-MS (Scharlau Kromaphase 100, C18, 5 μm, 250 x 4.6 mm, gradient from 50% CH_3CN/H_2O to 100% CH_3CN in 20 min, 1.0 mL/min).

3. Comparative tables of the spectroscopic data of the natural and the synthetic products

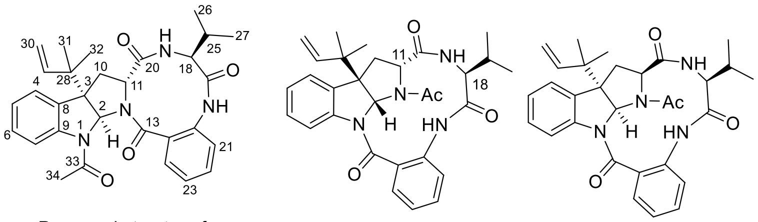

Proposed structure for (-)-novofumigatamide (D-Trp-*exo*-**1**)   D-Trp-regio-*endo*-**4**   L-Trp-regio-*endo*-**4**

The atom numbering from the original publication is used.[11]

Table 1. ^{1}H and $^{13}C\{^{1}H\}$ NMR data for the most representative signals of (-)-novofumigatamide (D-Trp-*exo*-**1**), D-Trp-regio-*endo*-**4** and L-Trp-regio-*endo*-**4** in $CDCl_3$ at 298 K.

	**Natural source**		**Synthetic compounds**			
	**(-)-novofumigatamide (D-Trp-*exo*-1)**		**D-Trp-regio-*endo*-4**		**L-Trp-regio-*endo*-4**	
**Atom no.**	δ_H (mult., *J*/Hz)	δ_C	δ_H (mult., *J*/Hz)	δ_C	δ_H (mult., *J*/Hz)	δ_C
2	6.04 (s)	79.2 CH	6.54 (s)	79.7 CH	6.26 (s)	80.7 CH
10	2.99 (dd, 12.4, 5.5, H_{10A})<br>2.62 (dd, 12.4, 10.7, H_{10B})	37.5 CH_2	2.82 (d, 12.9, H_{10A})<br>2.41 (dd, 12.9, 9.0, H_{10B})	34.8 CH_2	2.68-2.53 (m, H_{10A})<br>2.48-2.38 (m, H_{10B})	35.3 CH_2
11	4.43 (dd, 10.7, 5.5)	59.0 CH	5.32-5.13 (m)	61.3 CH	5.32-5.13 (m)	60.5 CH
18	5.22 (d, 7.5)	62.3 CH	4.21 (dd, 8.5, 3.6)	59.4 CH	3.18 (dd, 10.5, 6.7)	66.3 CH
25	1.98 (m)	31.9 CH	2.58-2.45 (m)	28.6 CH	2.86-2.72 (m)	27.5 CH
29	5.84 (dd, 17.4, 10.8)	143.1 CH	6.05 (dd, 17.3, 10.8)	143.1 CH	6.12-5.92 (m)	143.2 CH
30	5.13 (brs, 17.4)	114.6 CH_2	5.32-5.13 (m)	115.6 CH_2	5.32-5.13 (m)	111.5 CH_2

4. Spectra collection and HPLC-MS traces

^{1}H NMR (400.16 MHz, CD_3OD)

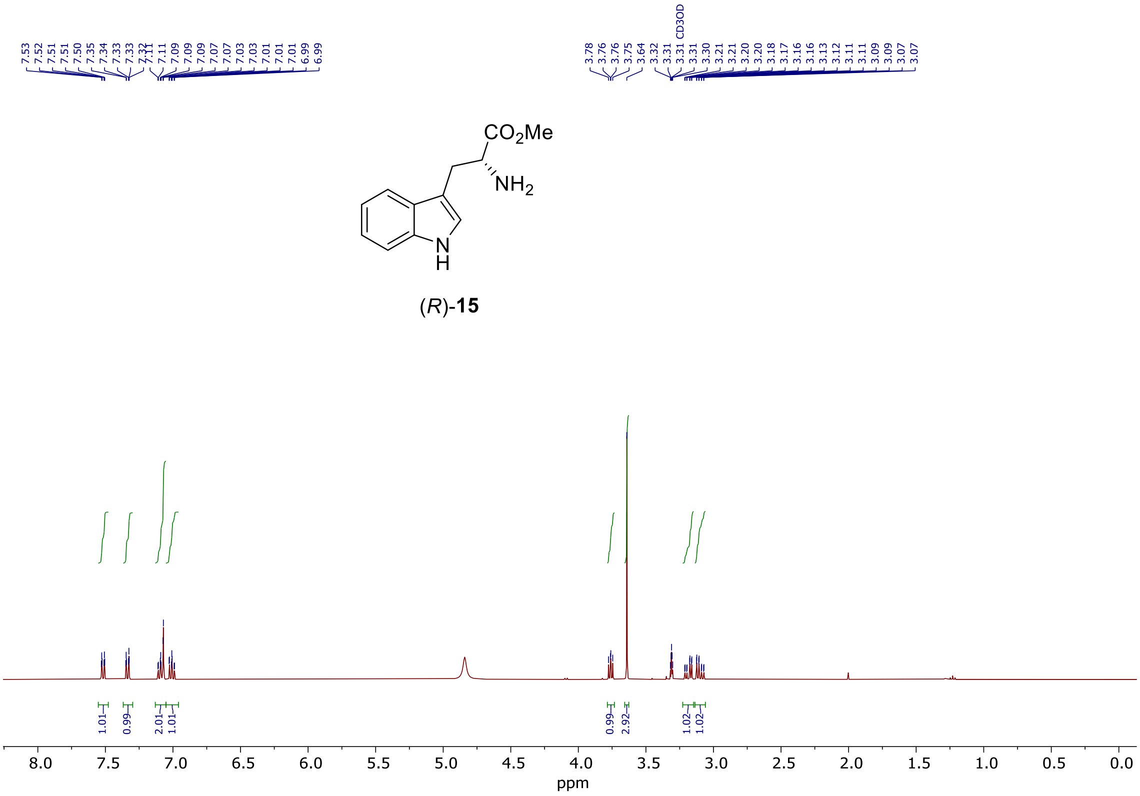

^{1}H NMR (400.16 MHz, $CDCl_3$)

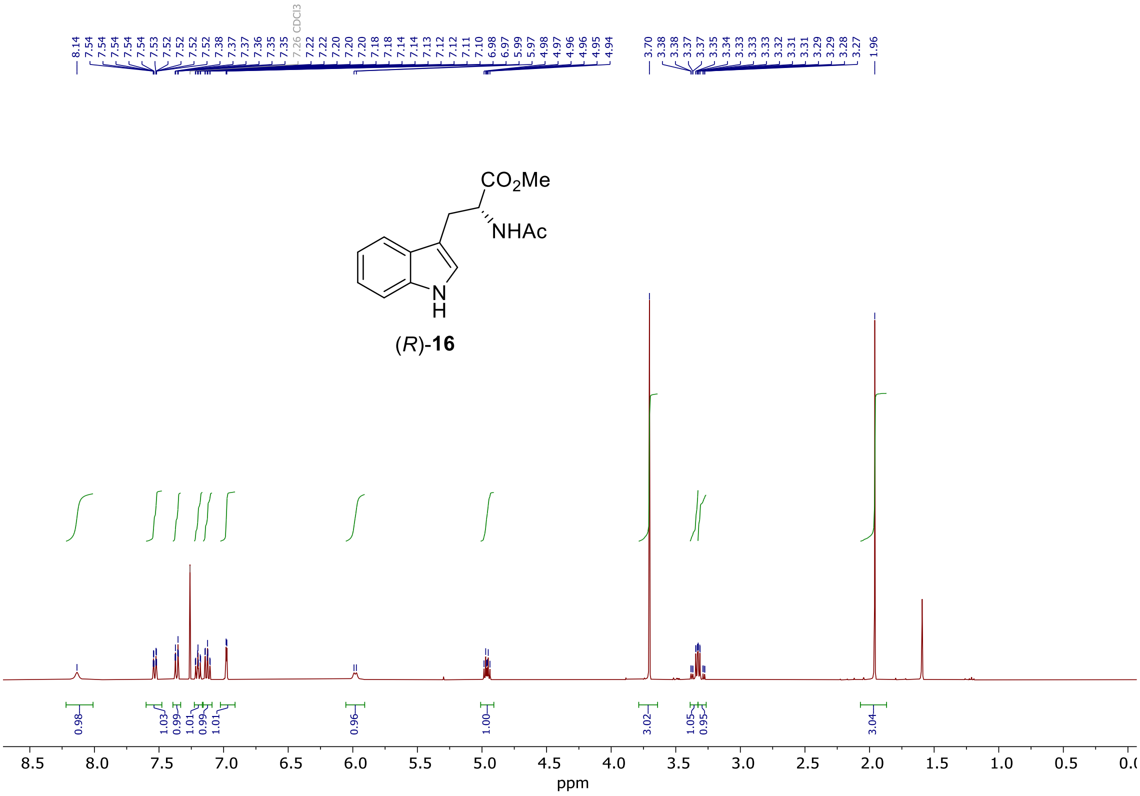

^{1}H NMR (400.16 MHz, DMSO-d_6, 343 K)

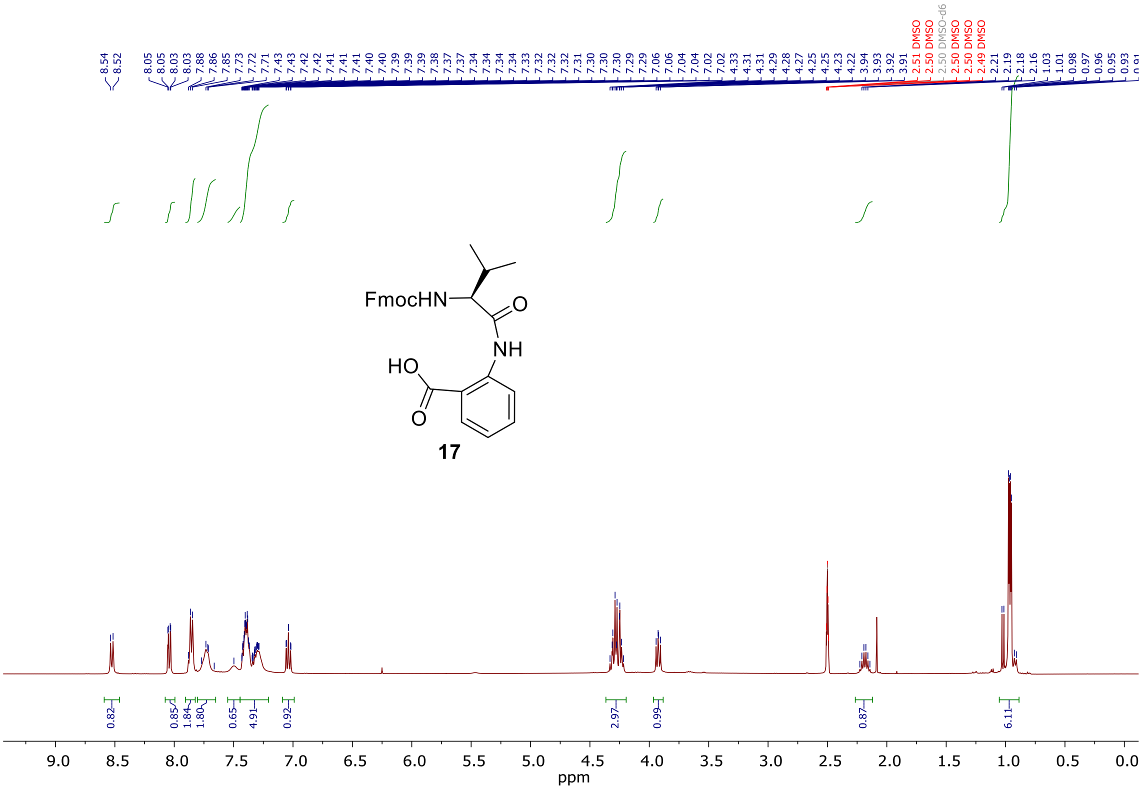

^{13}C{^{1}H} NMR (100.62 MHz, DMSO-d_6, 343 K)

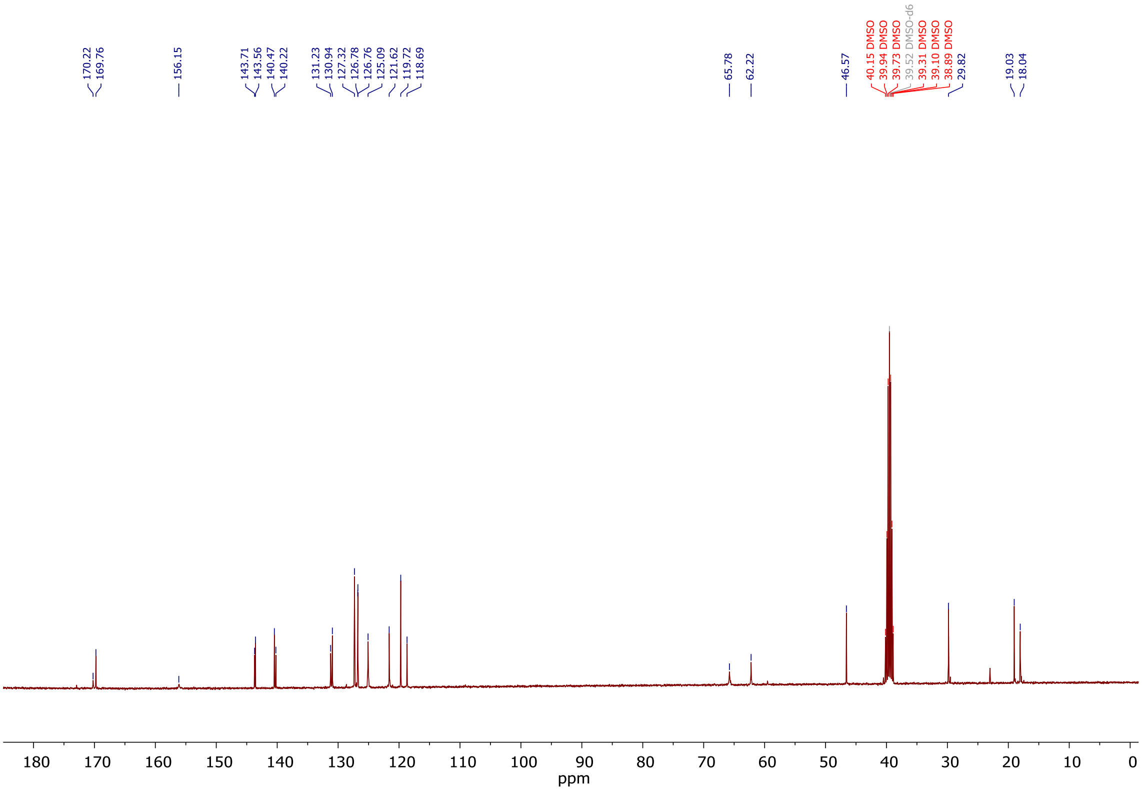

^{1}H NMR (400.16 MHz, CDCl$_3$, 298K)

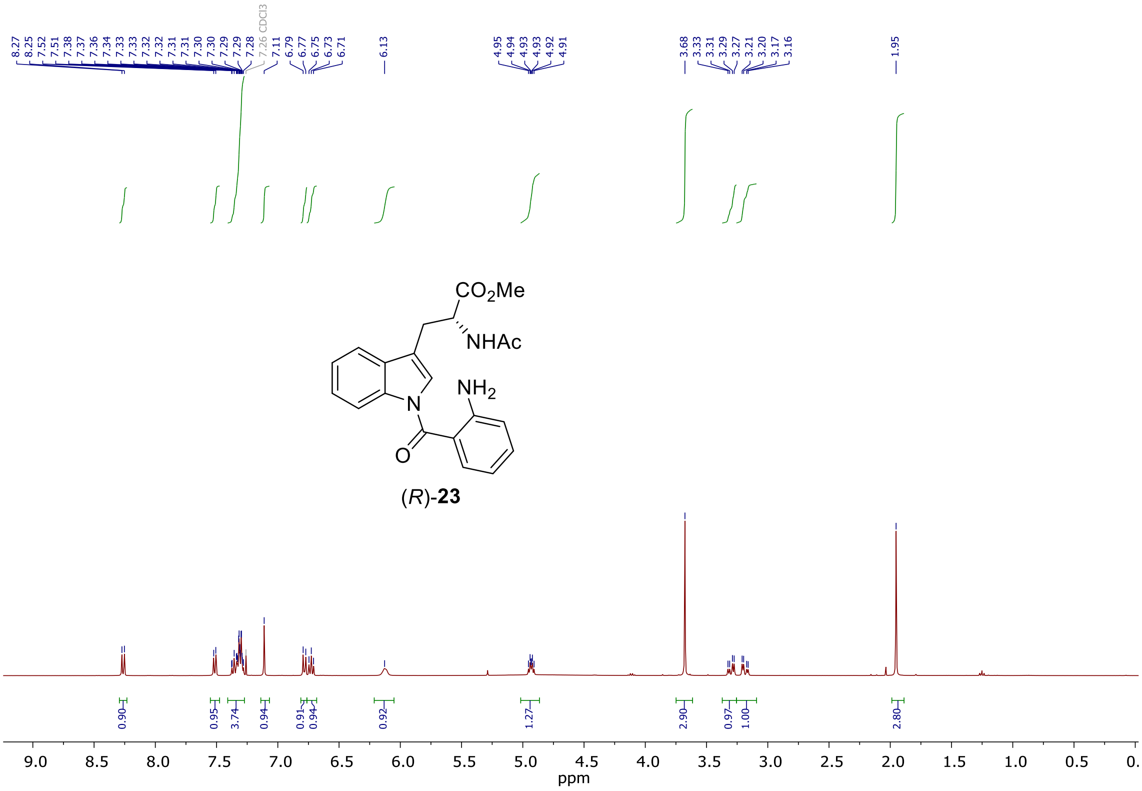

^{13}C{^{1}H} NMR (100.62 MHz, CDCl$_3$, 298 K)

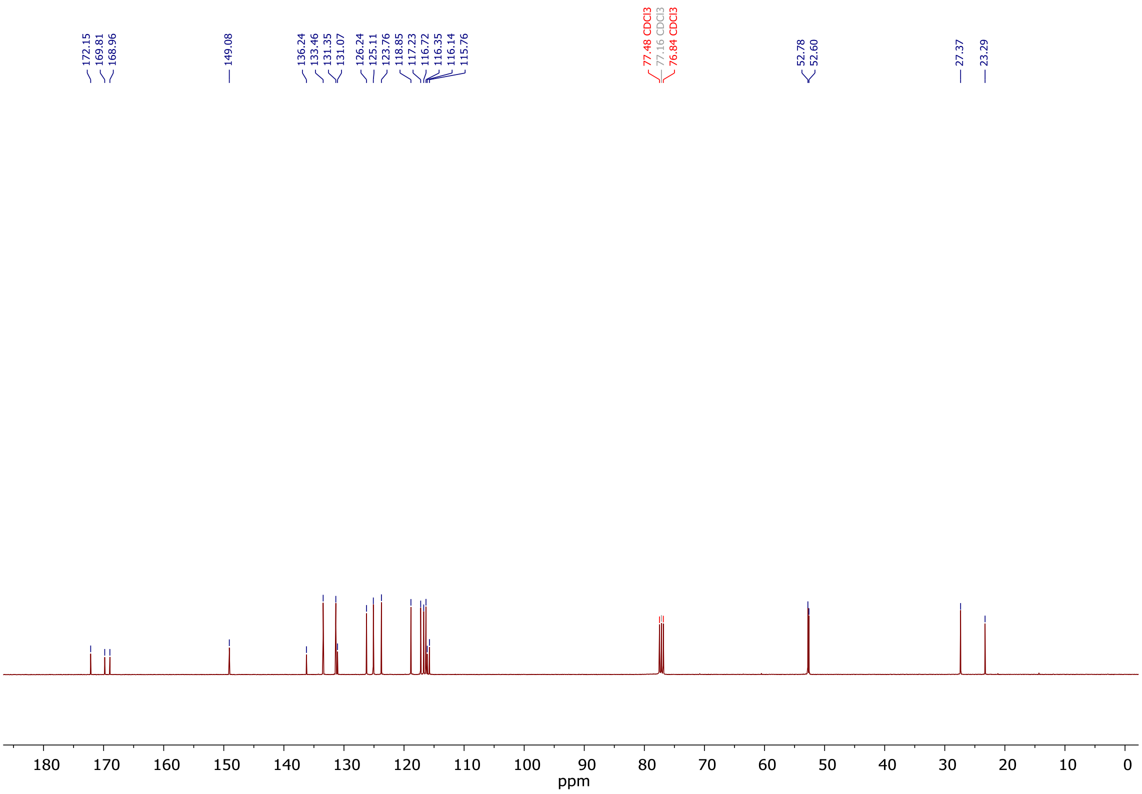

^{1}H NMR (400.16 MHz, DMSO-d_6, 343 K)

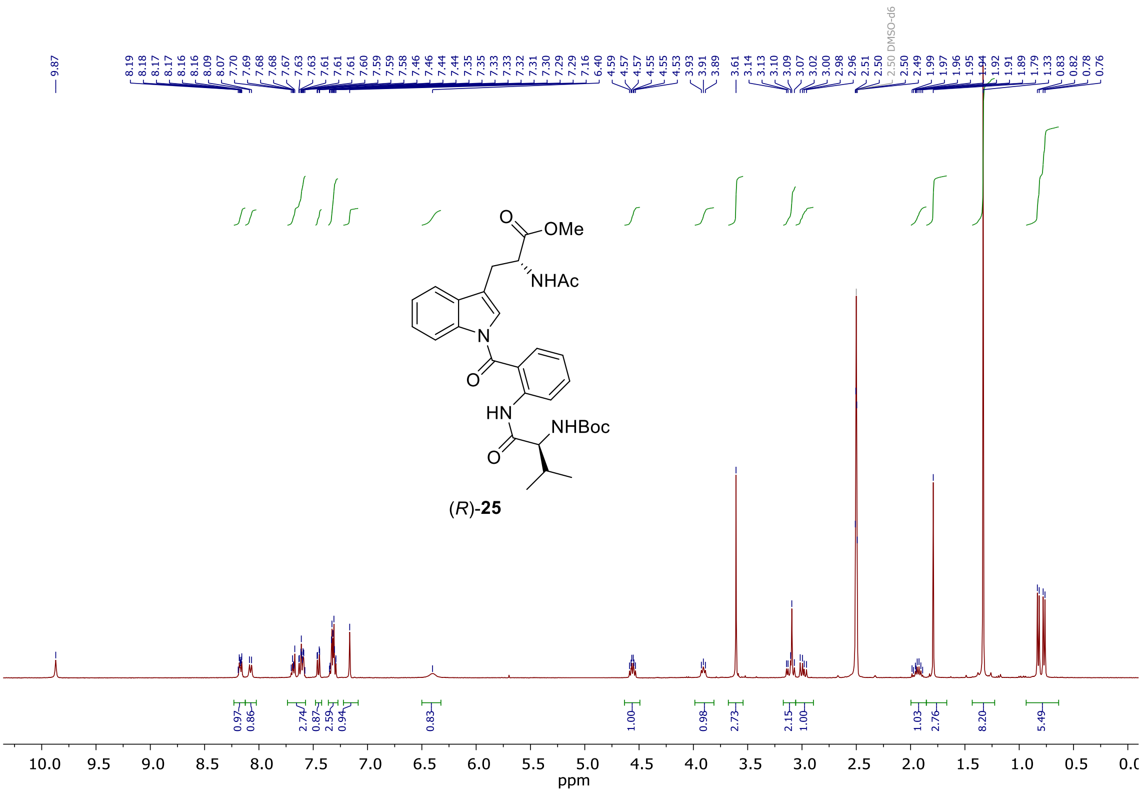

^{13}C{^{1}H} NMR (100.62 MHz, DMSO-d_6, 343 K)

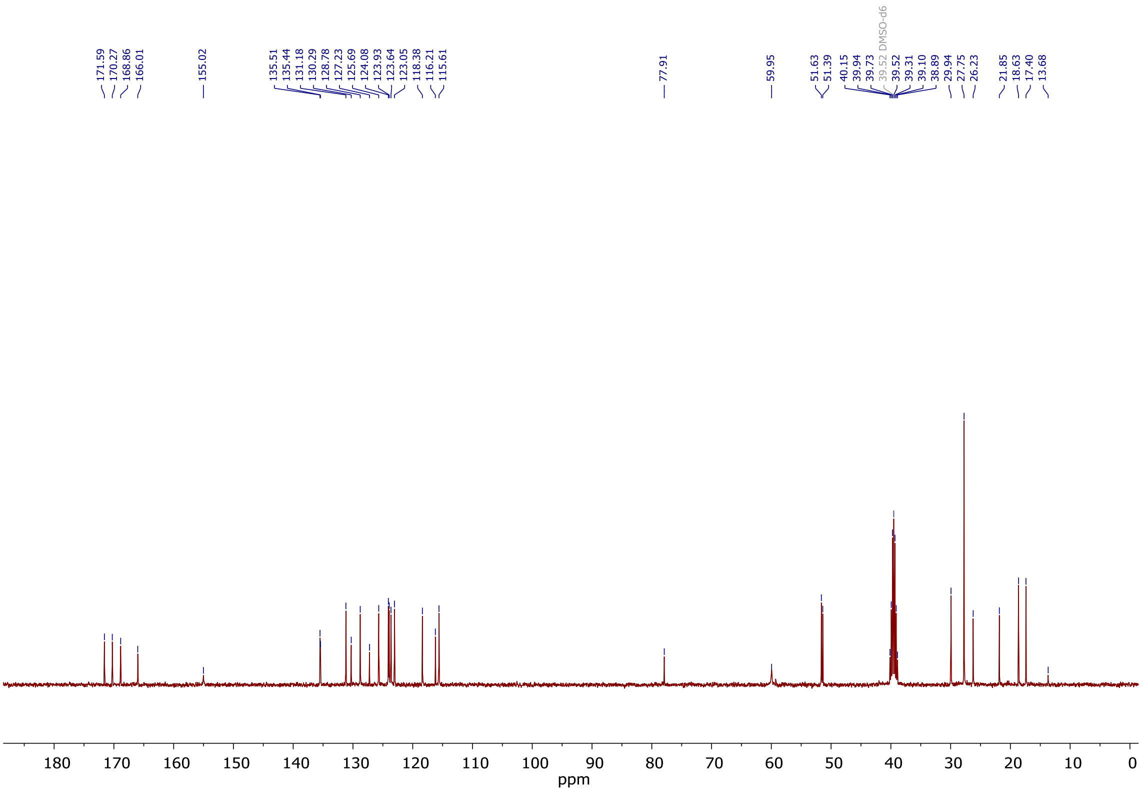

^{1}H NMR (400.16 MHz, DMSO-d_6, 343 K)

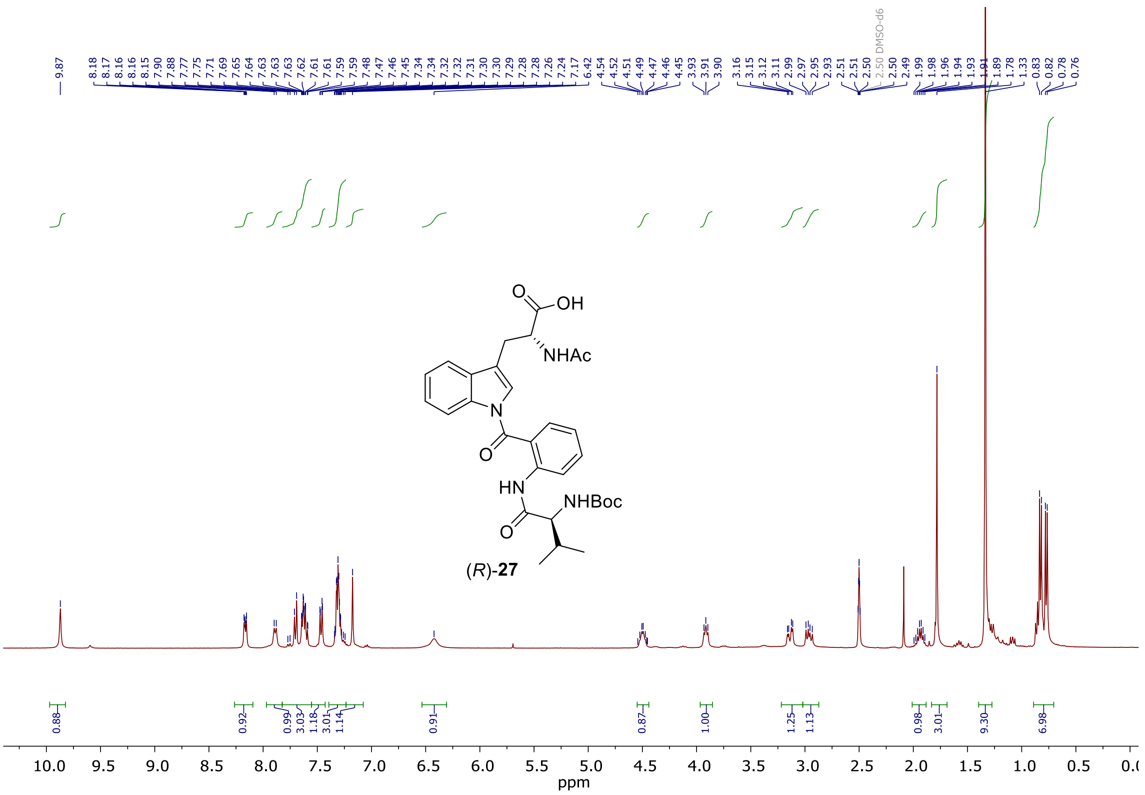

(*R*)-**27**

^{13}C{^{1}H} NMR (100.62 MHz, DMSO-d_6, 343 K)

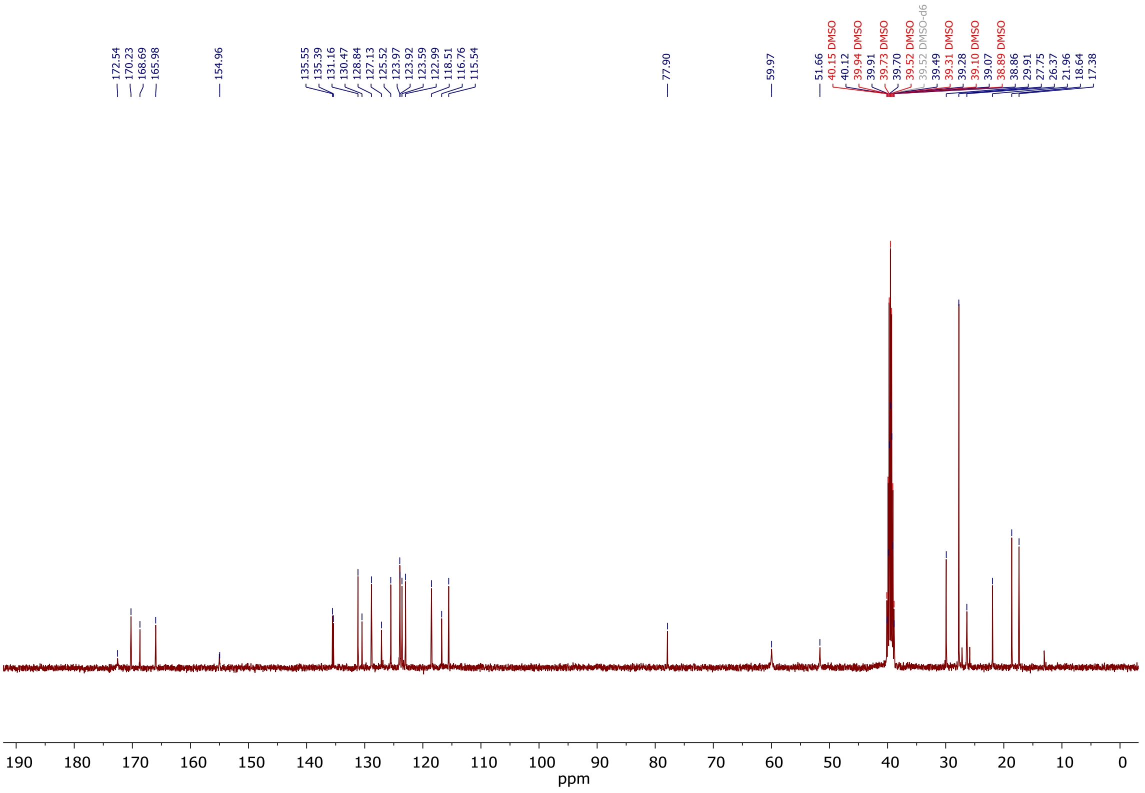

^{1}H NMR (400.16 MHz, DMSO-d_6, 343 K)

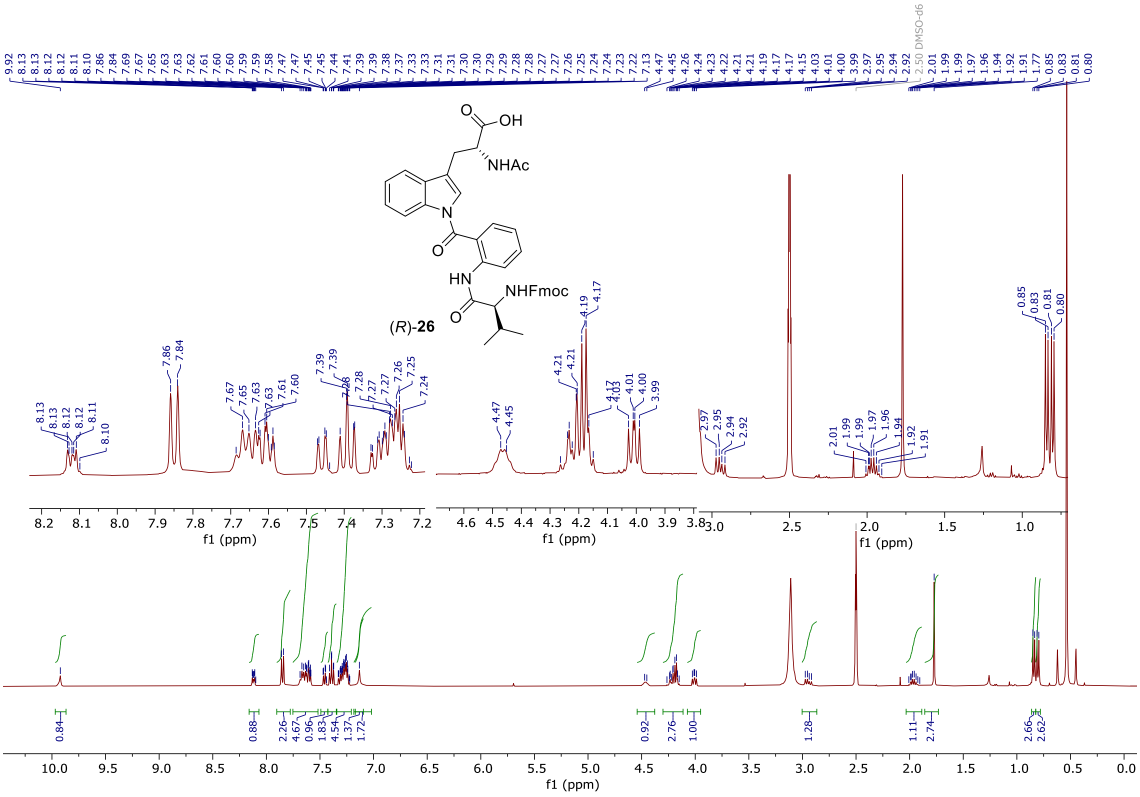

^{13}C{^{1}H} NMR (100.62 MHz, DMSO-d_6, 343 K)

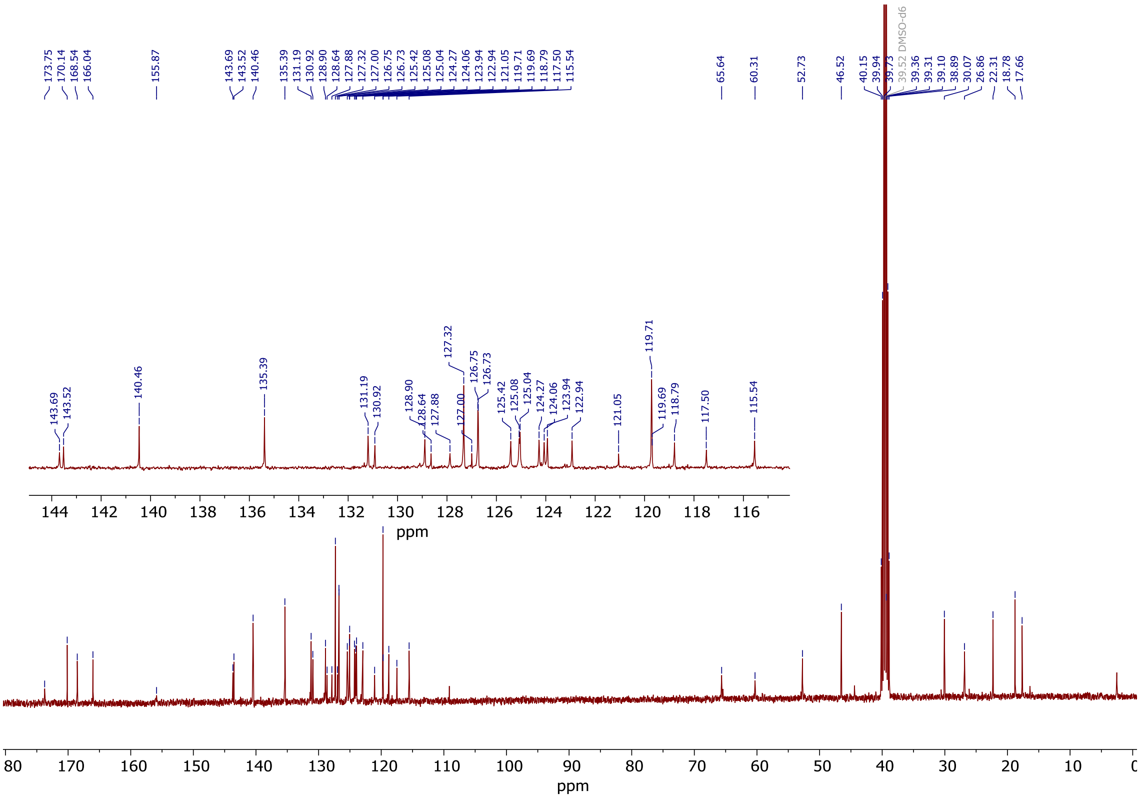

^{1}H NMR (400.16 MHz, DMSO-d_6, 343 K)

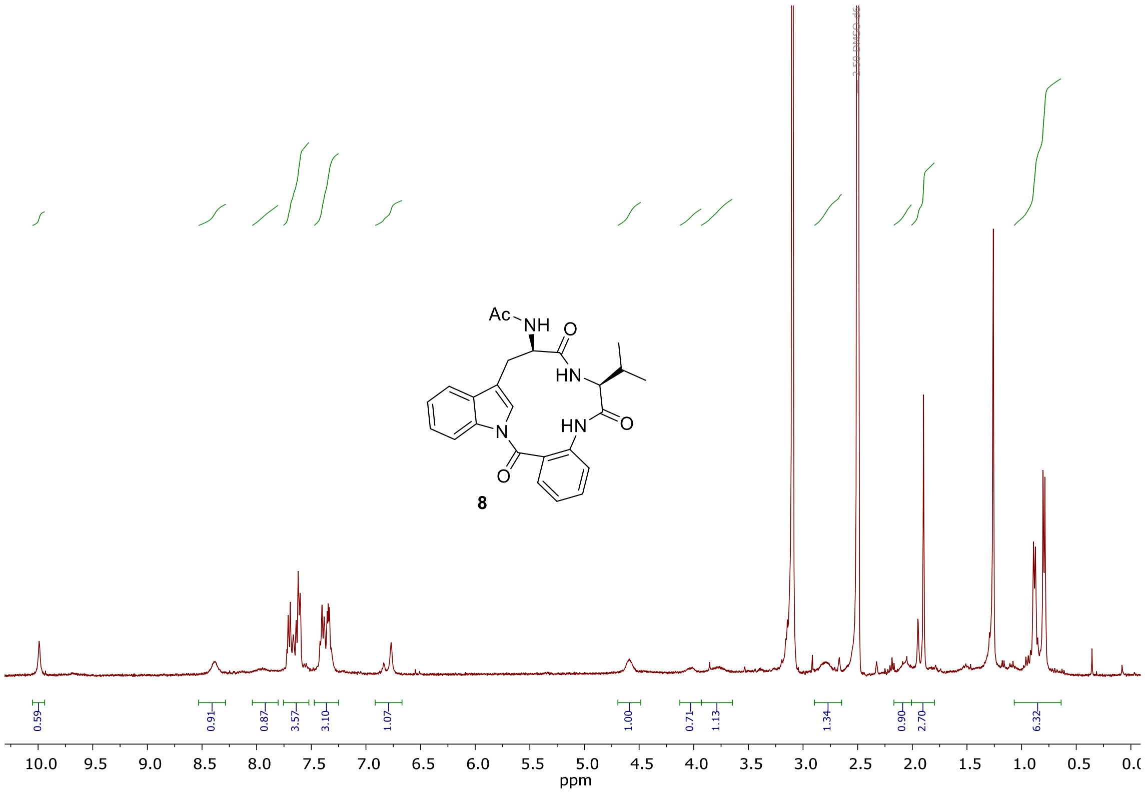

HPLC trace of **8** (SunFire™ analytic C18, 5 µm, 250 x 4.6 mm, gradient from CH_3CN/H_2O **5% to 100%** in 20 min, flow rate = 1.0 mL/min)

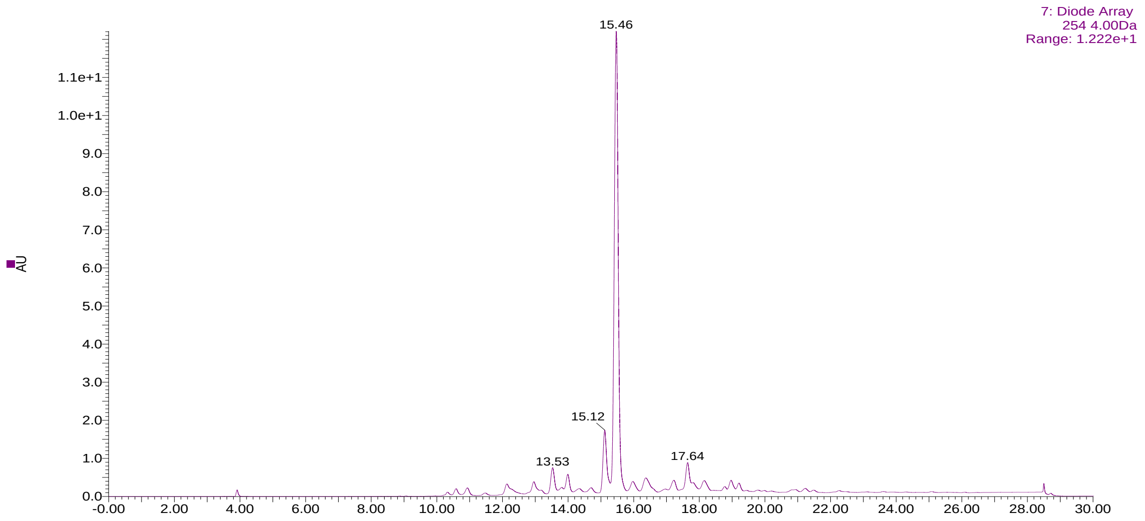

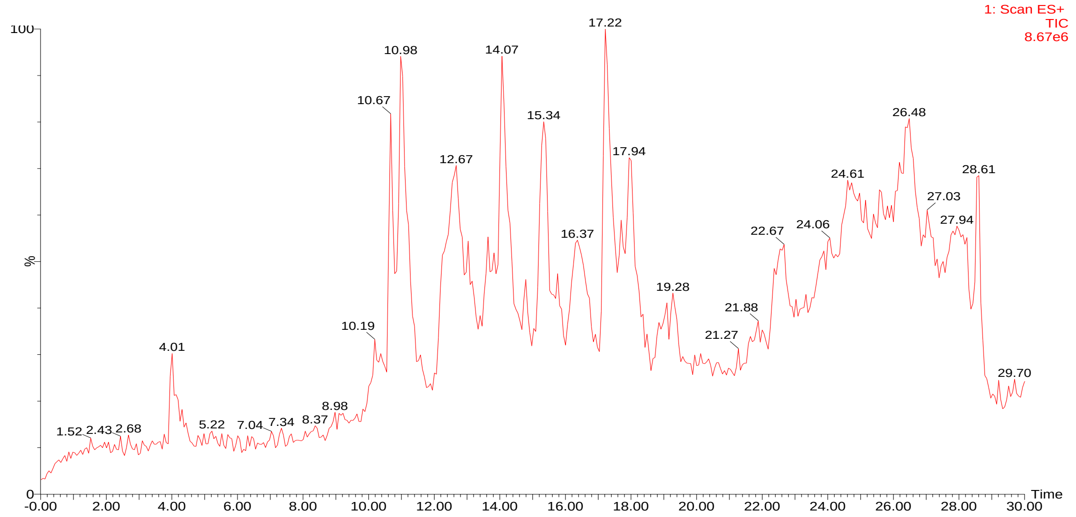

SunFire5 to 100AcN 20min 11072018 1 261 (15.762) Cm (260:263)

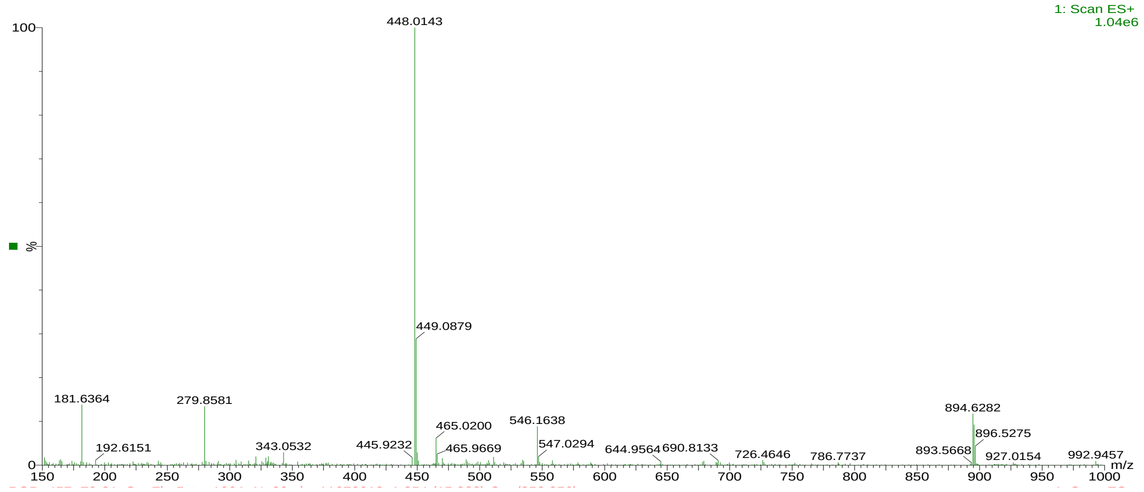

SunFire5 to 100AcN 20min 11072018 1 254 (15.338) Cm (252:256)

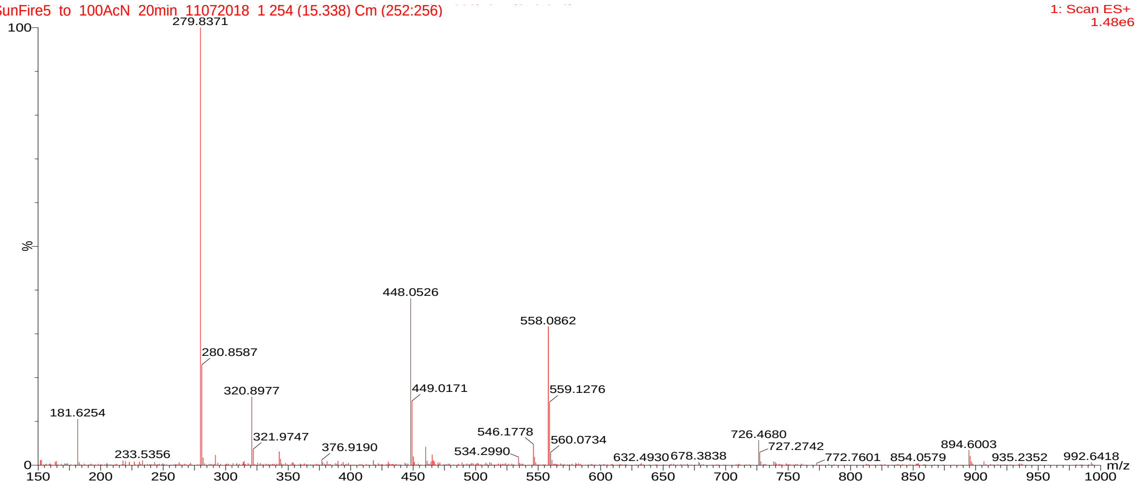

HPLC trace of **8** (SunFire™ analytic C18, 5 µm, flow = 1 mL/min, gradient from CH_3CN/H_2O **50% to 100%** in 20 min, 1.0 mL/min).

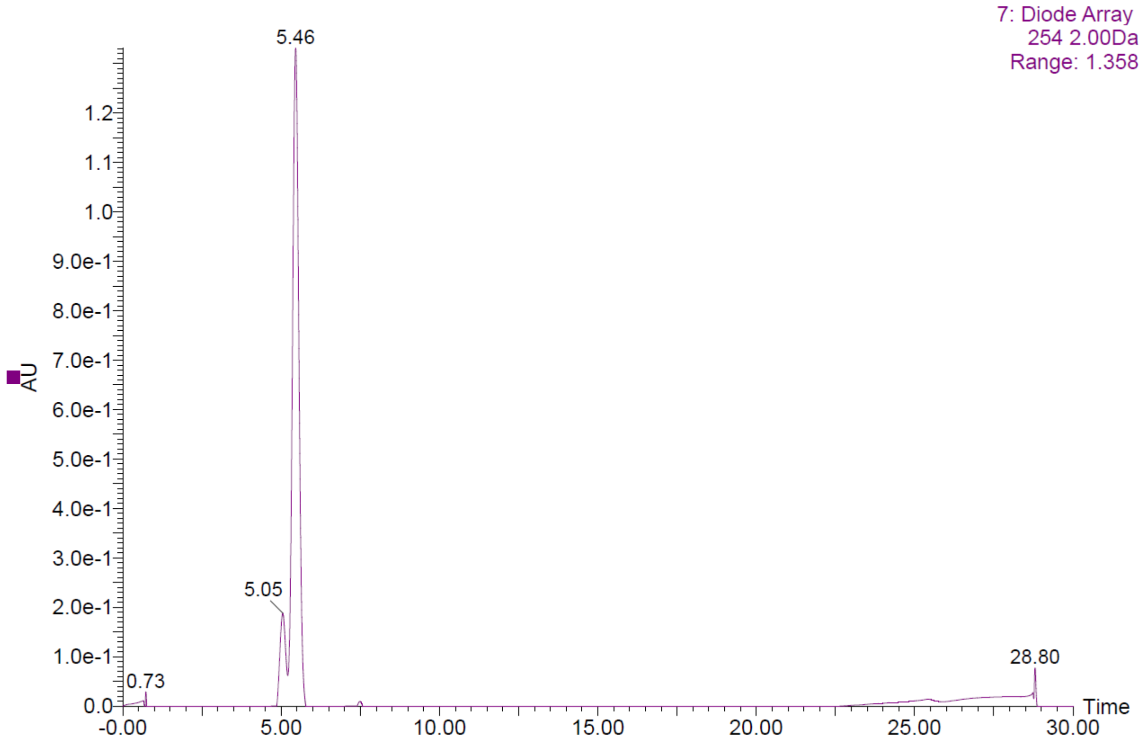

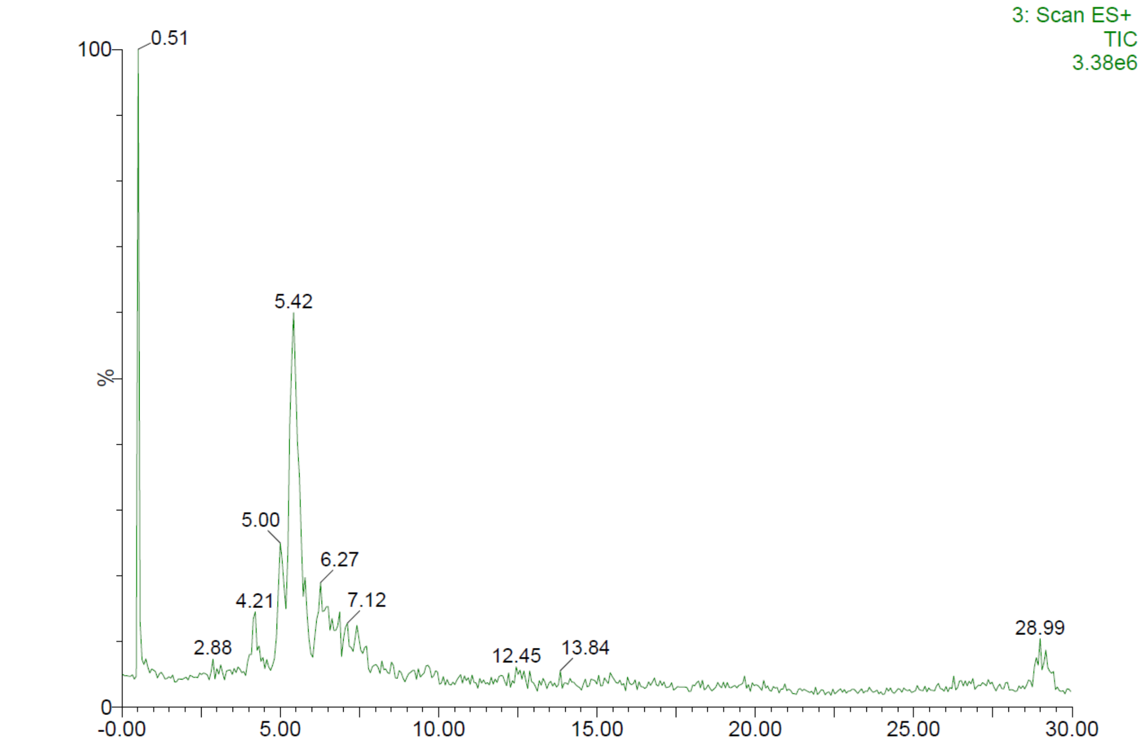

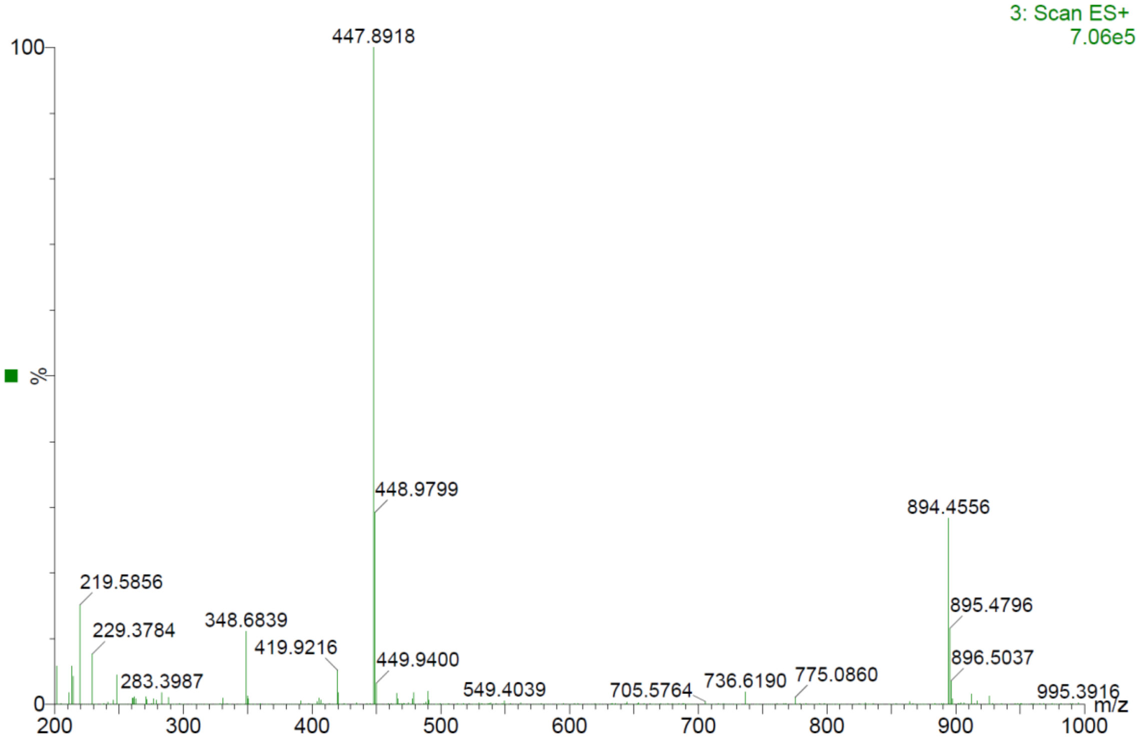
3: Scan ES+
7.06e5
447.8918
448.9799
894.4556
219.5856
895.4796
229.3784
348.6839
419.9216
449.9400
896.5037
283.3987
736.6190
775.0860
549.4039
705.5764
995.3916
m/z
%

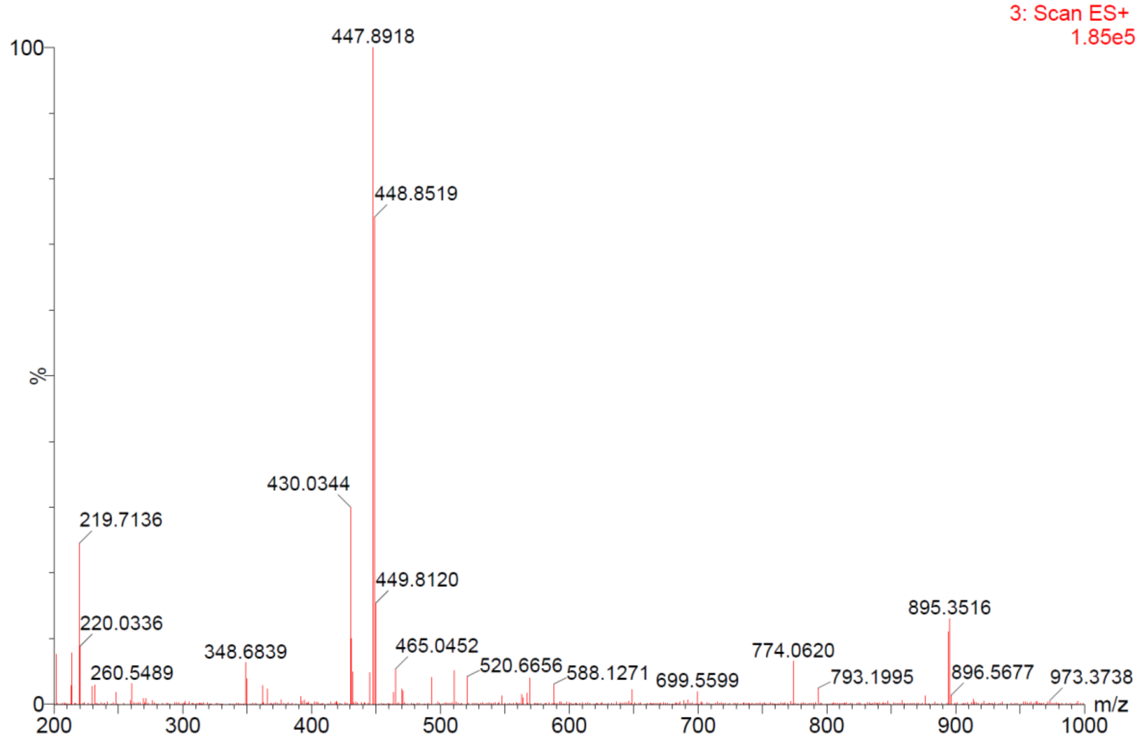
3: Scan ES+
1.85e5
447.8918
448.8519
430.0344
219.7136
449.8120
895.3516
220.0336
465.0452
774.0620
348.6839
520.6656
260.5489
588.1271
699.5599
793.1995
896.5677
973.3738
m/z
%

HPLC trace of diolide (SunFire™ analytic C18, 5 µm, 250 x 4.6 mm, gradient from CH_3CN/H_2O **5% to 100%** in 20 min, flow rate = 1.0 mL/min)

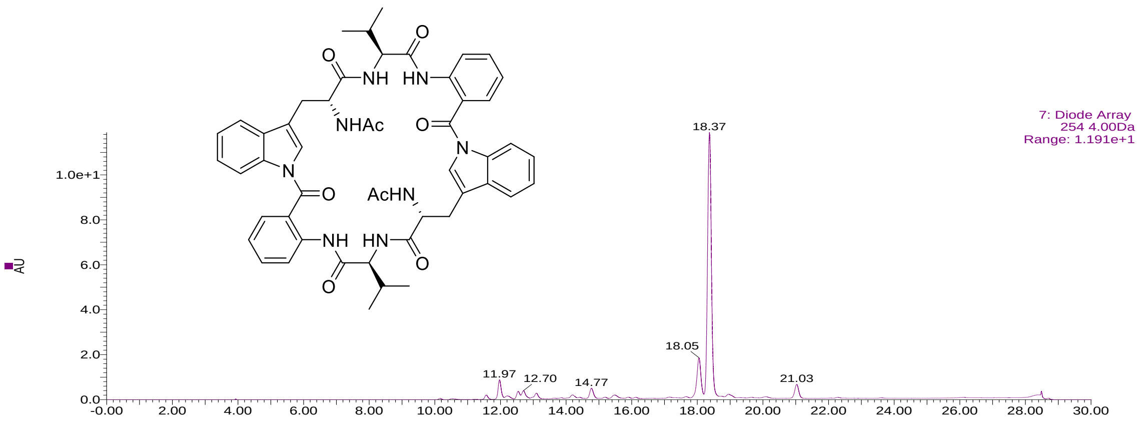

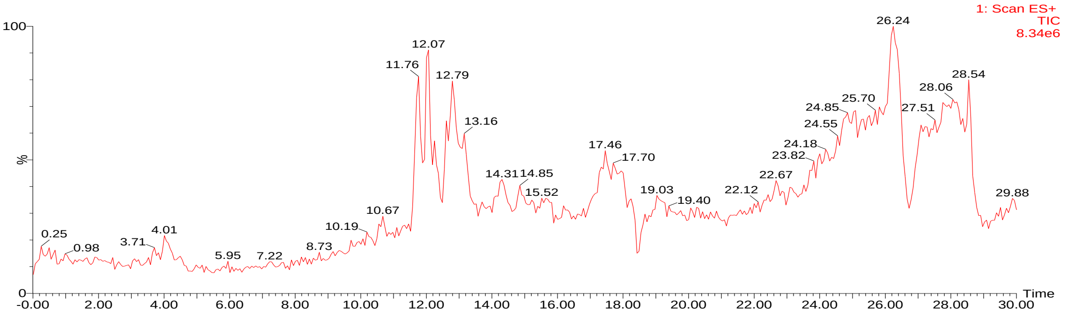

SunFire5_to_100AcN_20min_11072018 308 (18.609) Cm (305:308)

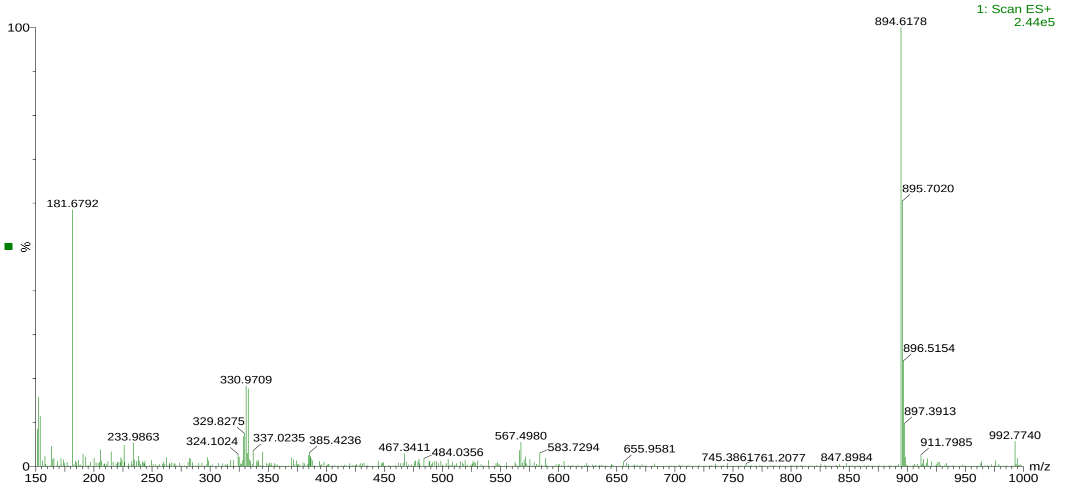

HPLC trace of diolide (SunFire™ analytic C18, 5 µm, 250 x 4.6 mm, gradient from CH_3CN/H_2O **50% to 100%** in 20 min, flow rate = 1.0 mL/min)

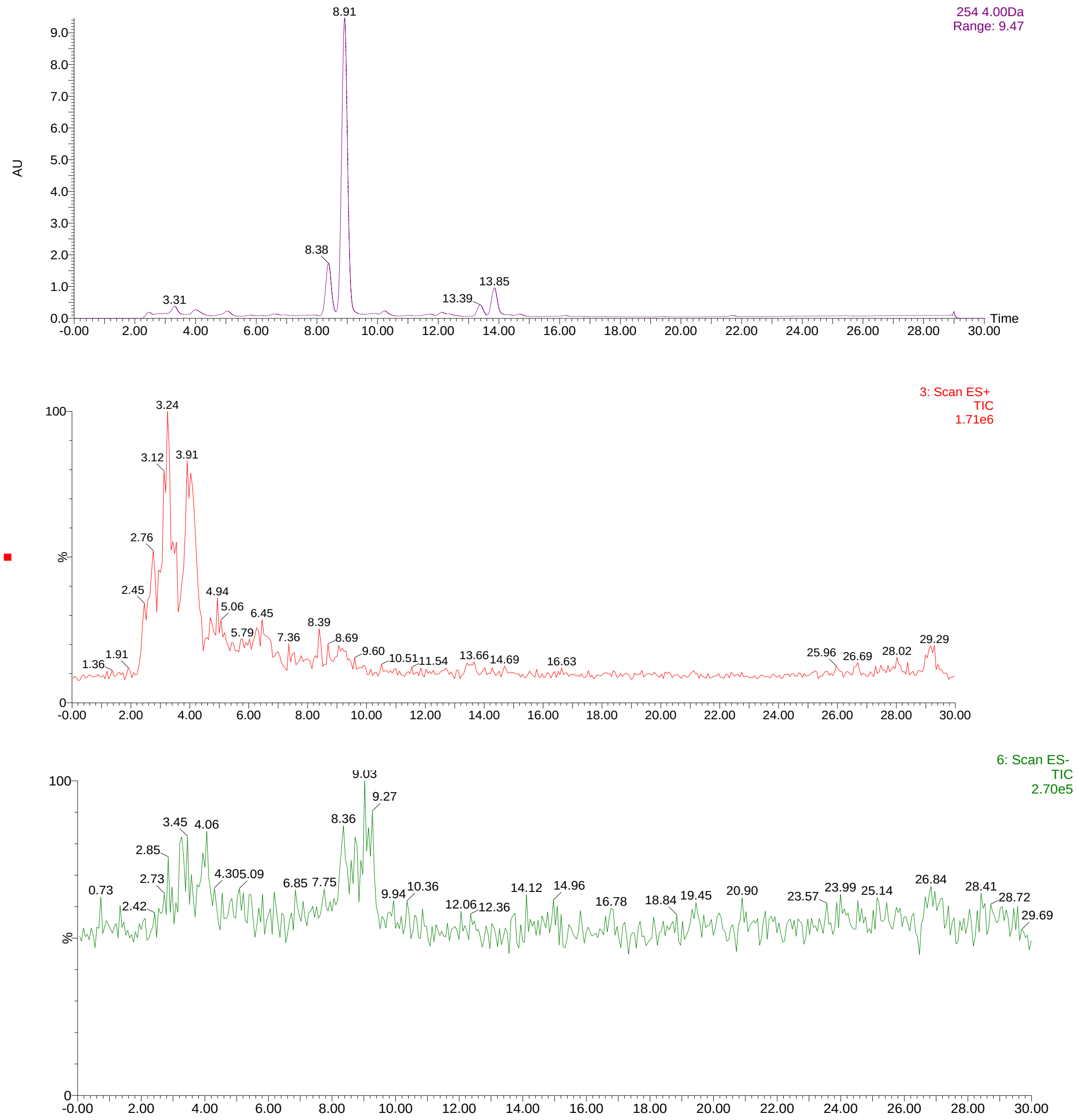

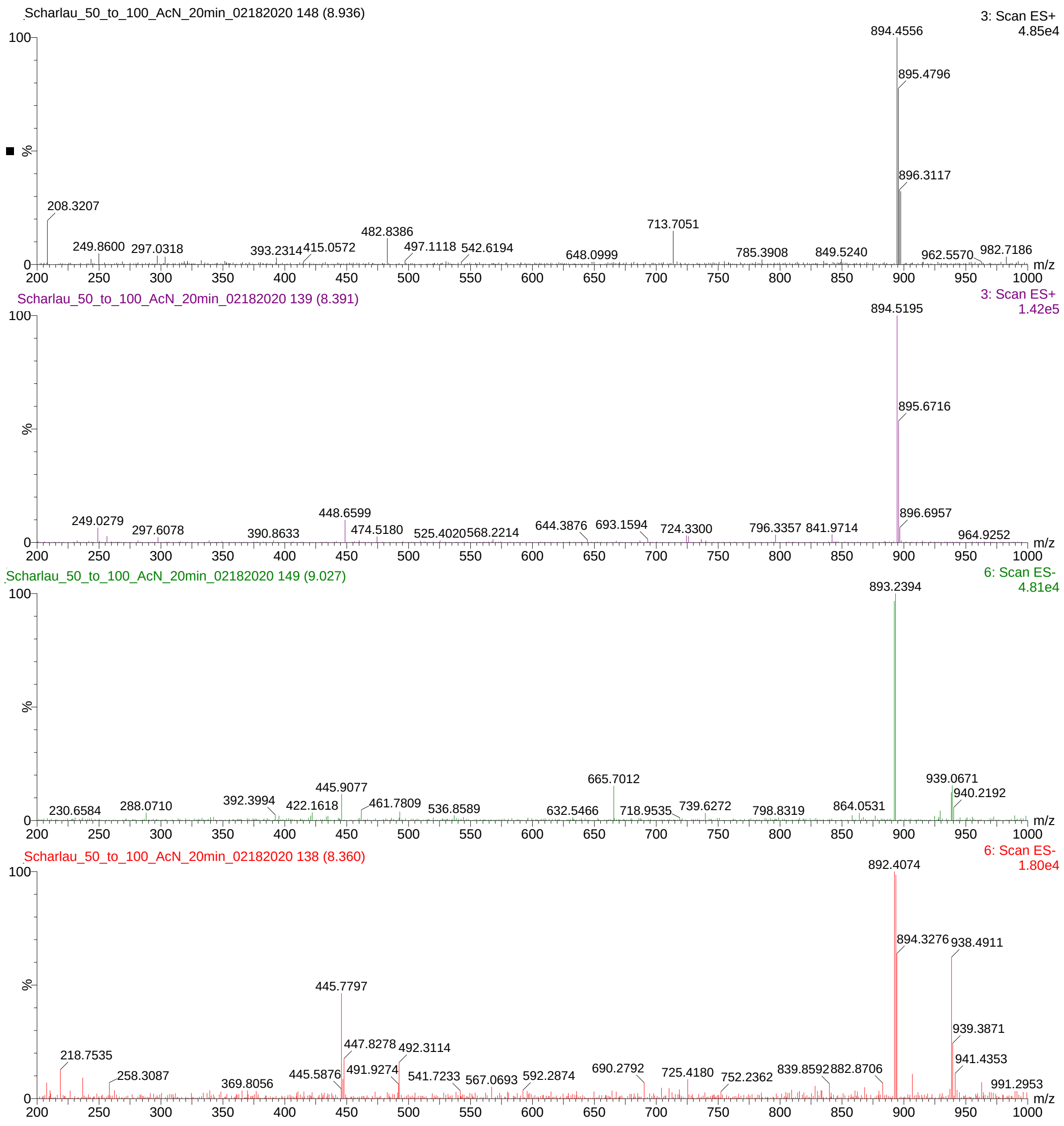

Scharlau_50_to_100_AcN_20min_02182020 148 (8.936)
3: Scan ES+
4.85e4
894.4556
895.4796
896.3117
208.3207
249.8600
297.0318
393.2314
415.0572
482.8386
497.1118
542.6194
648.0999
713.7051
785.3908
849.5240
962.5570
982.7186
Scharlau_50_to_100_AcN_20min_02182020 139 (8.391)
3: Scan ES+
1.42e5
894.5195
895.6716
896.6957
249.0279
297.6078
390.8633
448.6599
474.5180
525.4020
568.2214
644.3876
693.1594
724.3300
796.3357
841.9714
964.9252
Scharlau_50_to_100_AcN_20min_02182020 149 (9.027)
6: Scan ES-
4.81e4
893.2394
939.0671
940.2192
230.6584
288.0710
392.3994
422.1618
445.9077
461.7809
536.8589
632.5466
665.7012
718.9535
739.6272
798.8319
864.0531
Scharlau_50_to_100_AcN_20min_02182020 138 (8.360)
6: Scan ES-
1.80e4
892.4074
894.3276
938.4911
939.3871
941.4353
991.2953
218.7535
258.3087
369.8056
445.5876
445.7797
447.8278
491.9274
492.3114
541.7233
567.0693
592.2874
690.2792
725.4180
752.2362
839.8592
882.8706
%
m/z

^{1}H NMR (400.16 MHz, CDCl$_3$, 298K)

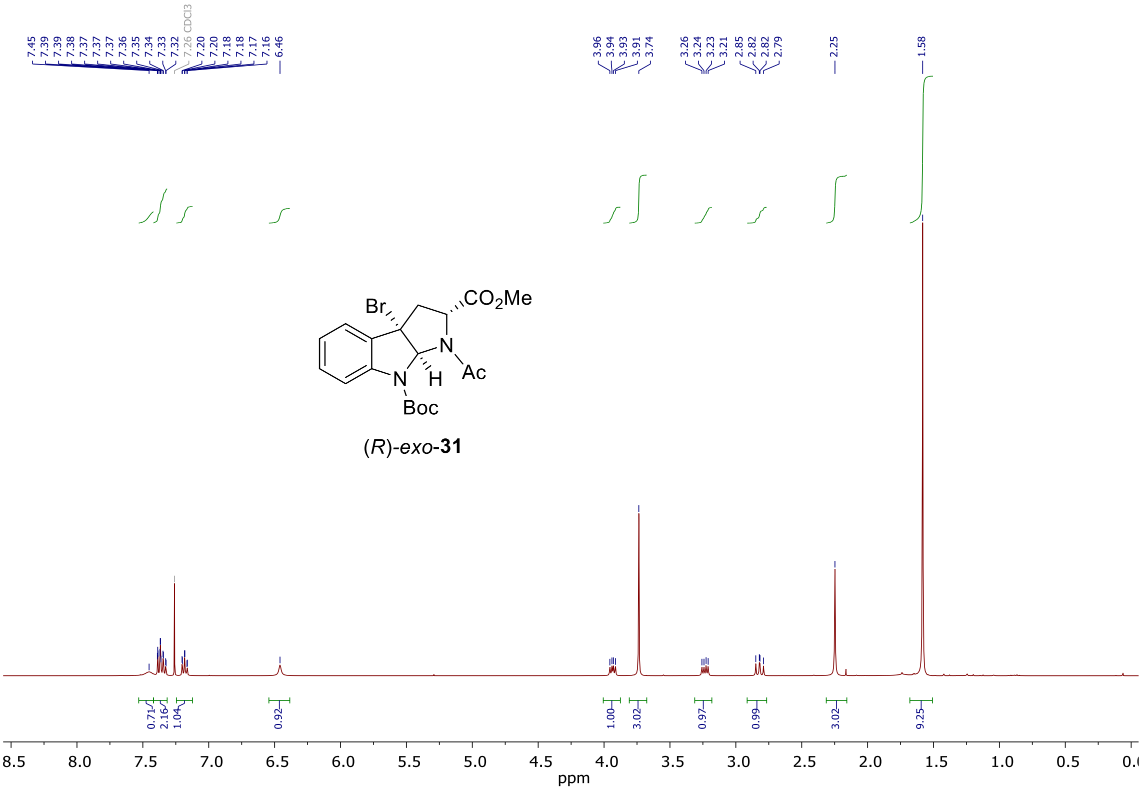

^{13}C{^{1}H} NMR (100.62 MHz, CDCl$_3$, 298 K)

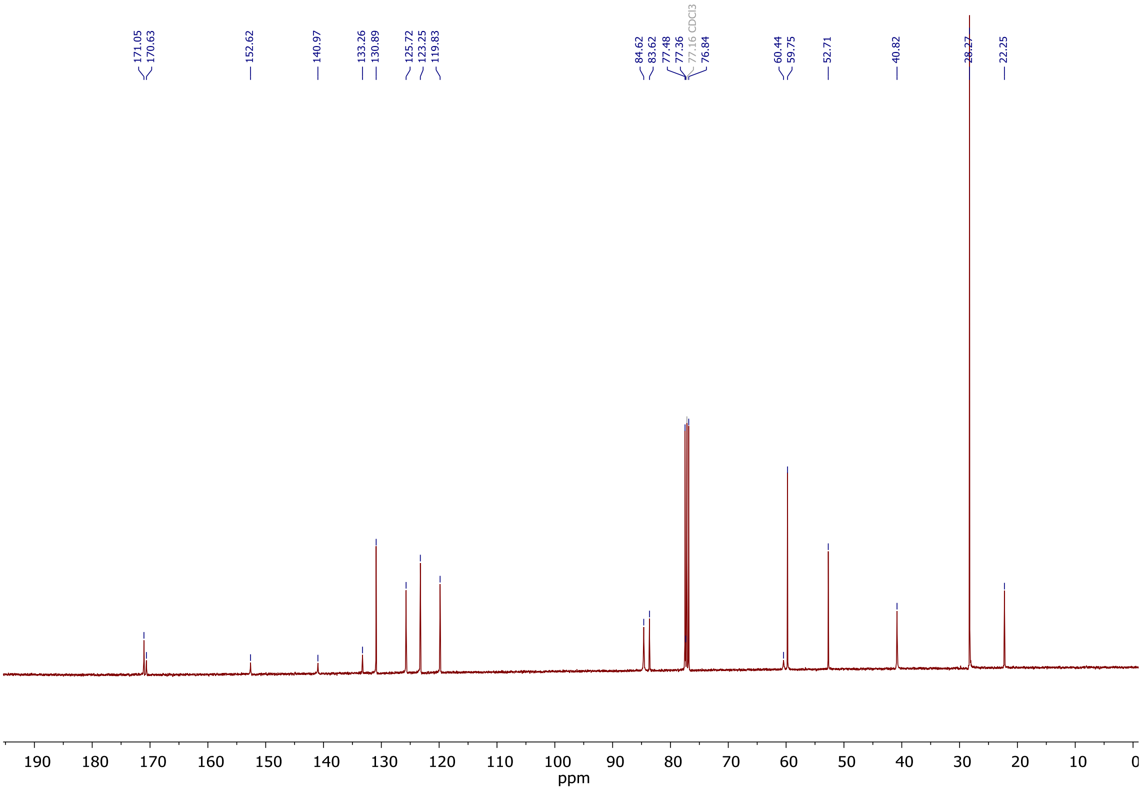

^{1}H NMR (400.16 MHz, $CDCl_3$, 298 K)

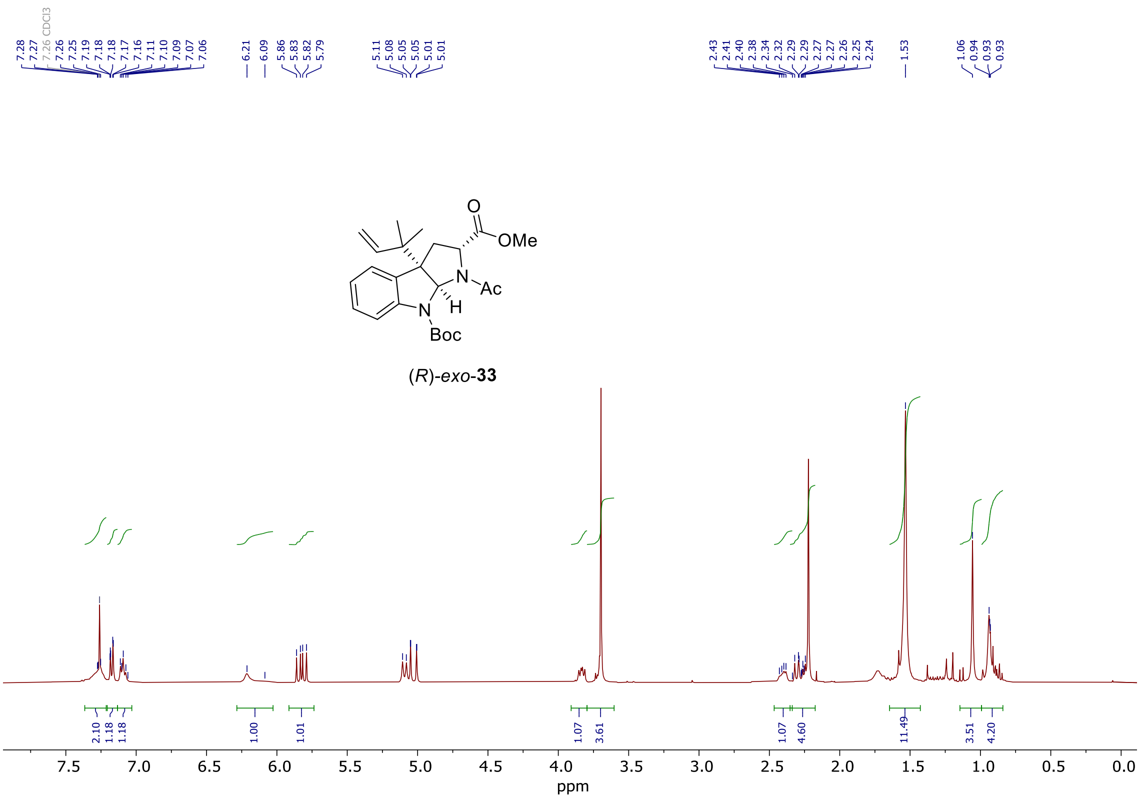

^{13}C{^{1}H} NMR (100.62 MHz, $CDCl_3$, 298 K)

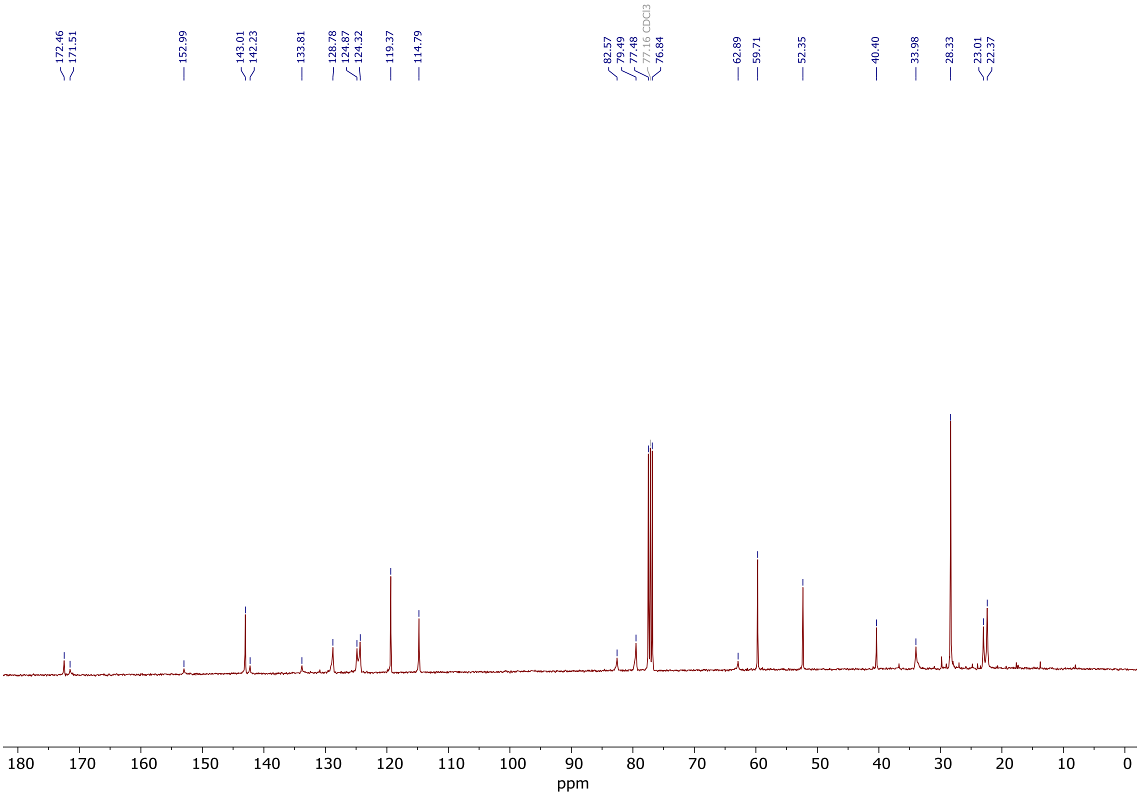

^{1}H NMR (400.16 MHz, $CDCl_3$, 298 K)

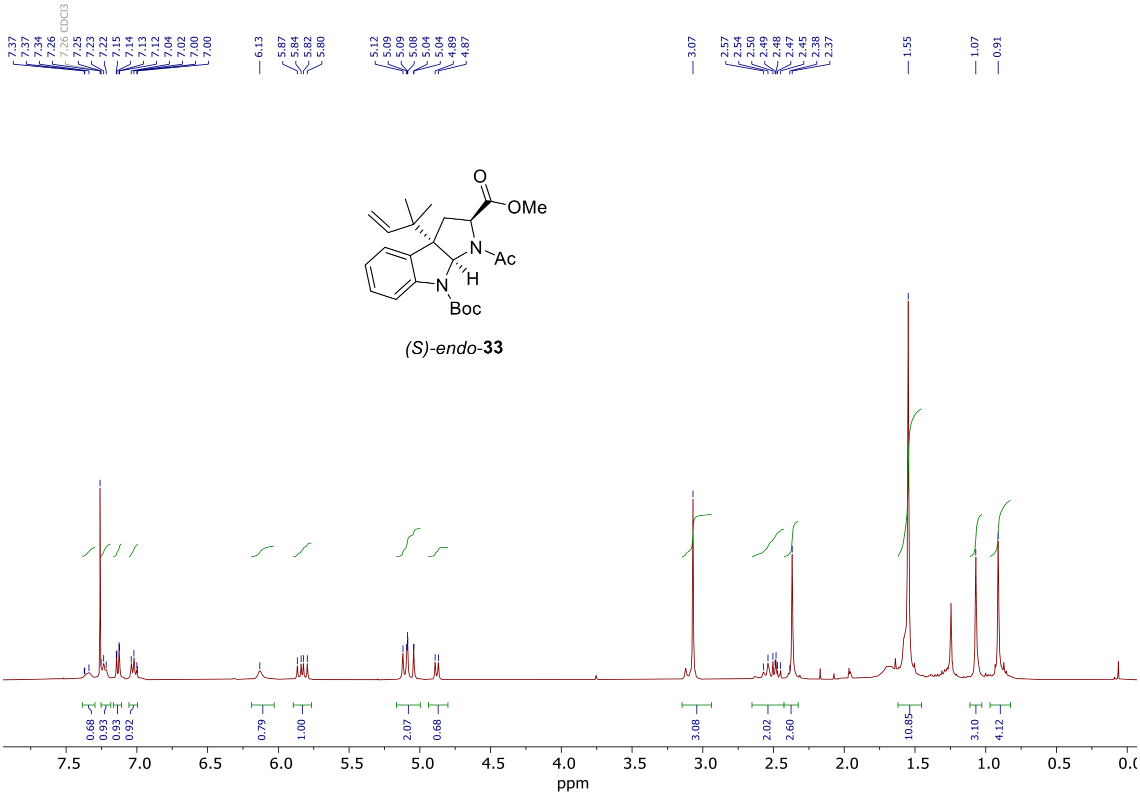

$^{13}C\{^{1}H\}$ NMR (100.62 MHz, $CDCl_3$, 298 K)

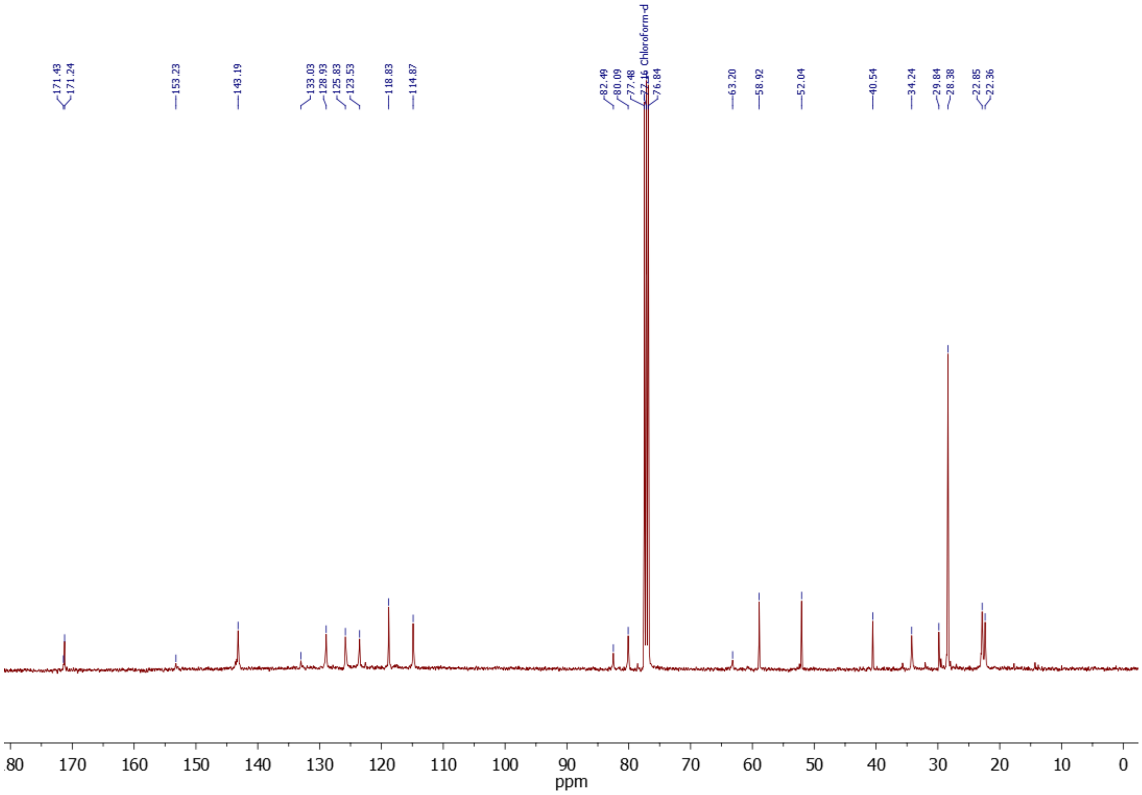

^{1}H NMR (400.16 MHz, DMSO-d_6, 343 K)

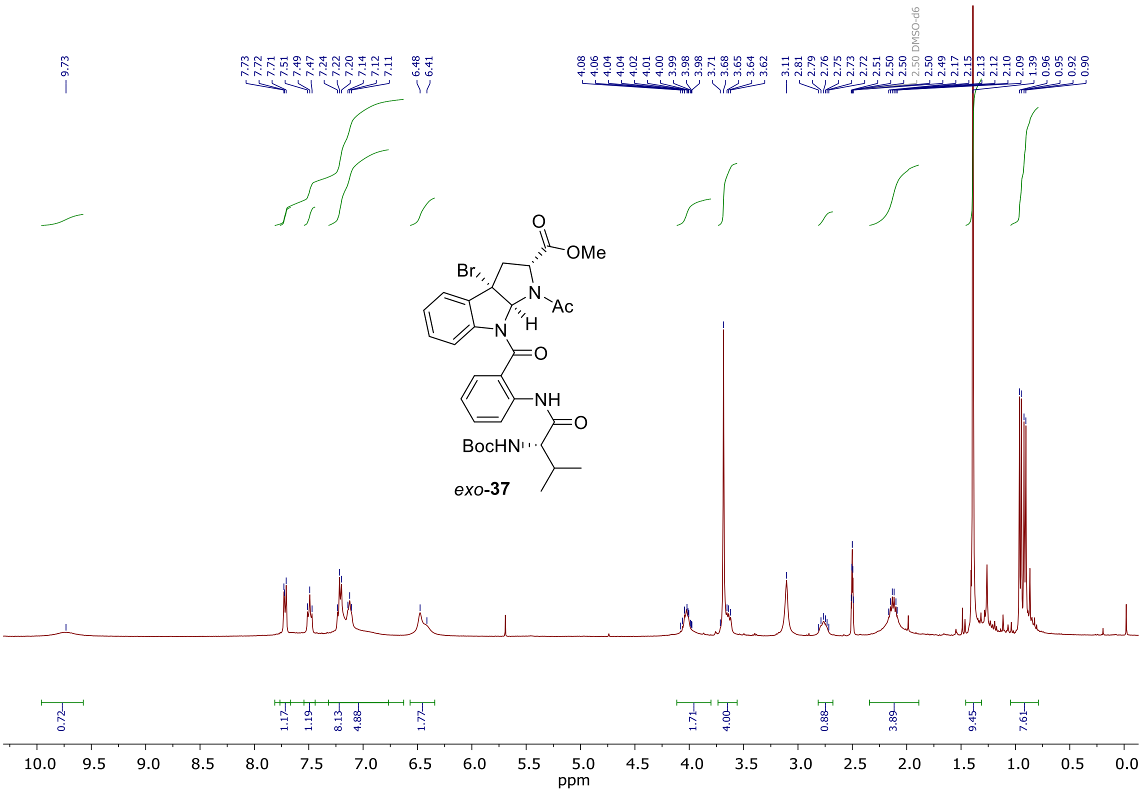

^{13}C{^{1}H} NMR (100.62 MHz, DMSO-d_6, 343 K)

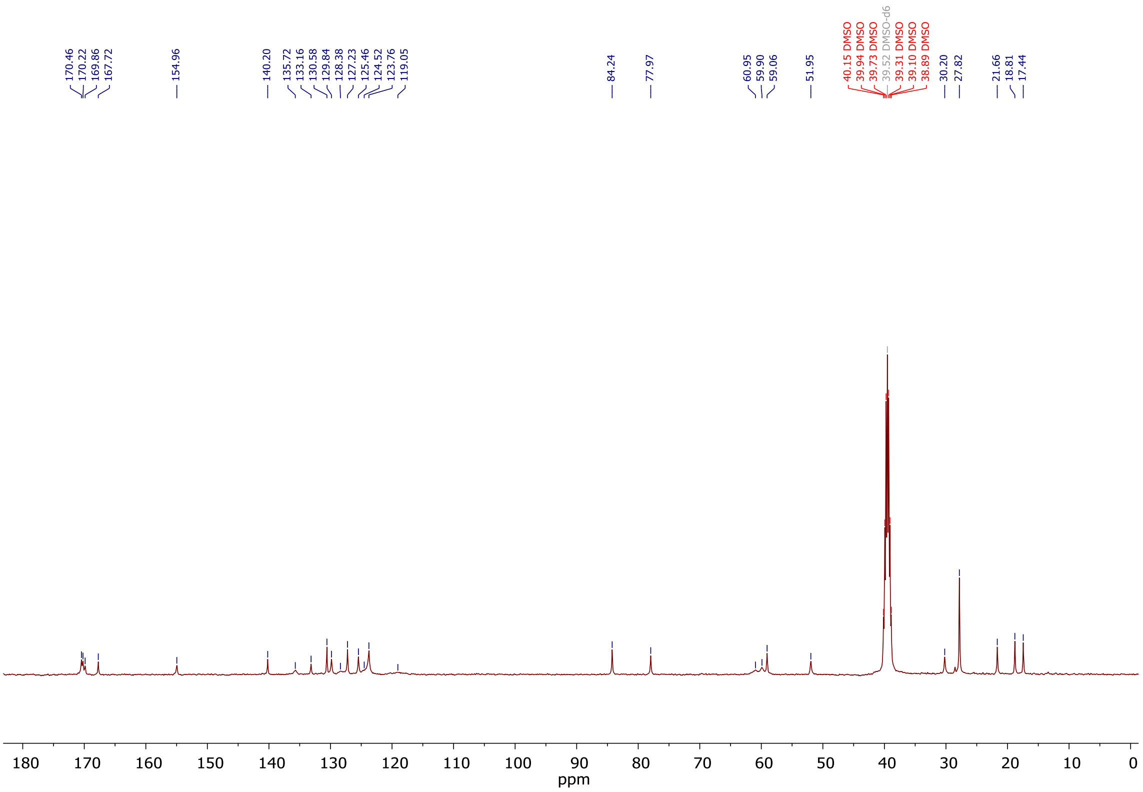

^{1}H NMR (400.16 MHz, DMSO-d_6, 363 K)

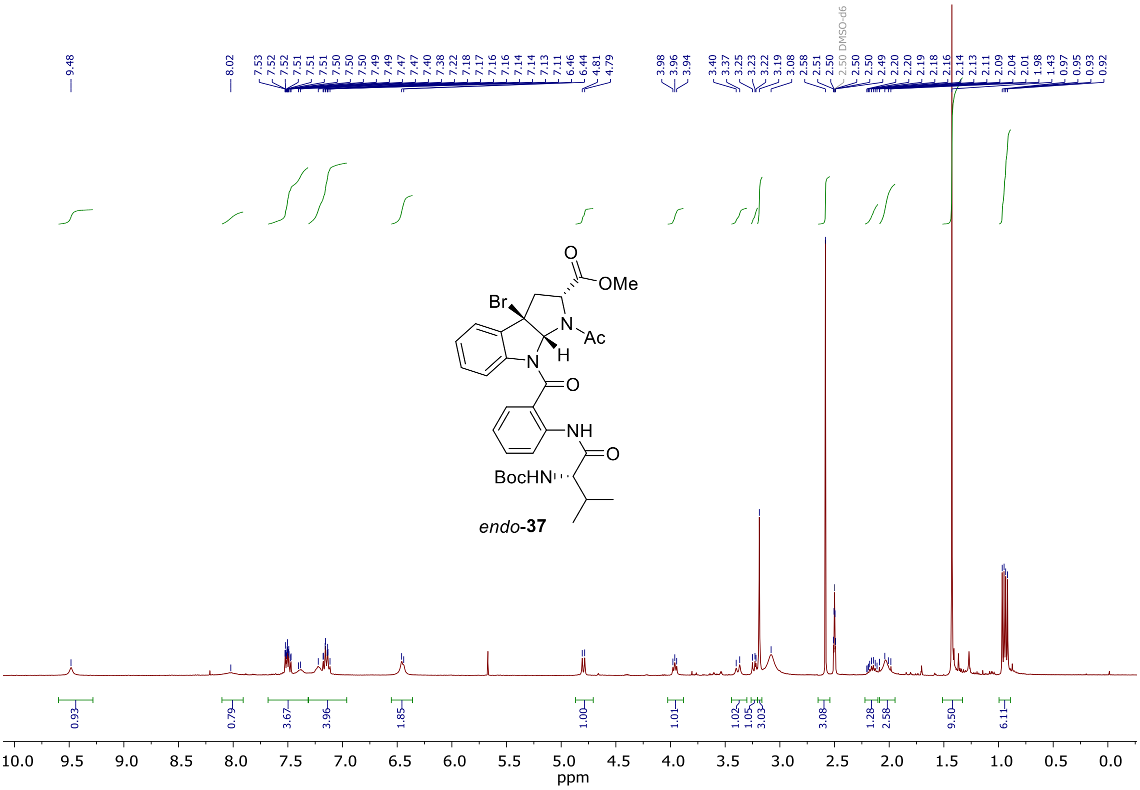

^{1}H NMR (400.16 MHz, DMSO-d_6, 343 K)

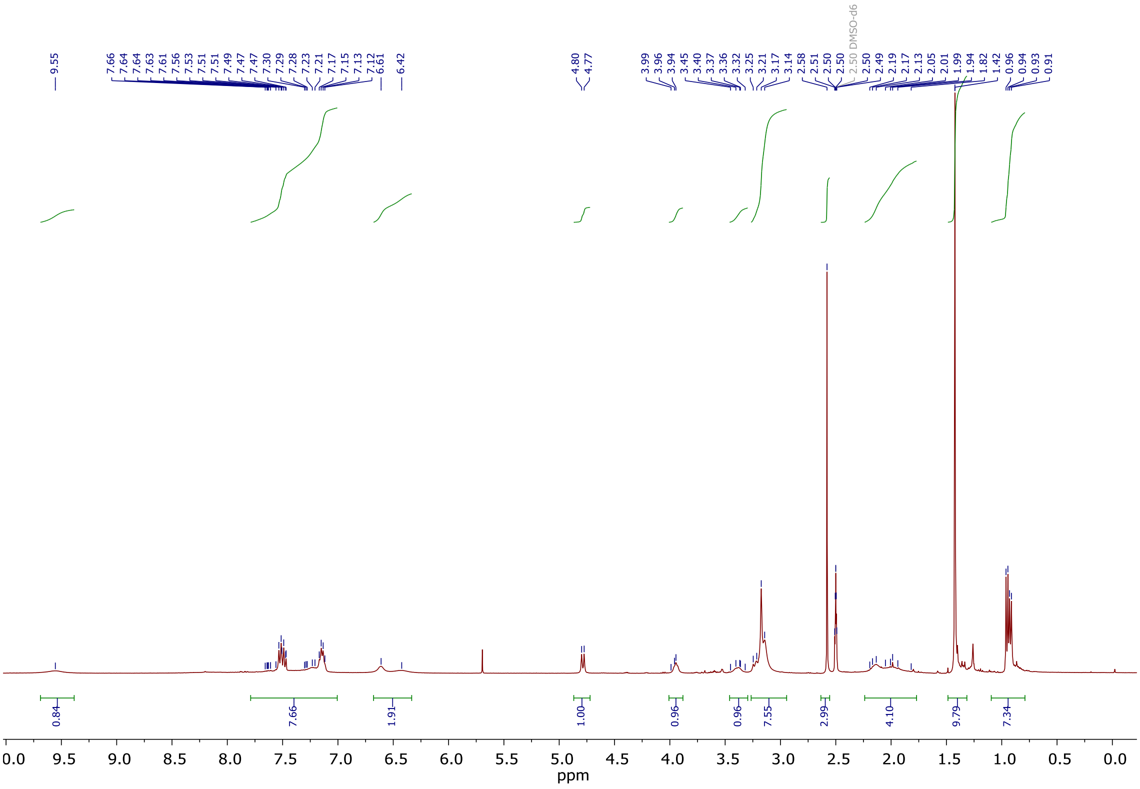

$^{13}C\{^{1}H\}$ NMR (100.62 MHz, DMSO-d_6, 363 K)

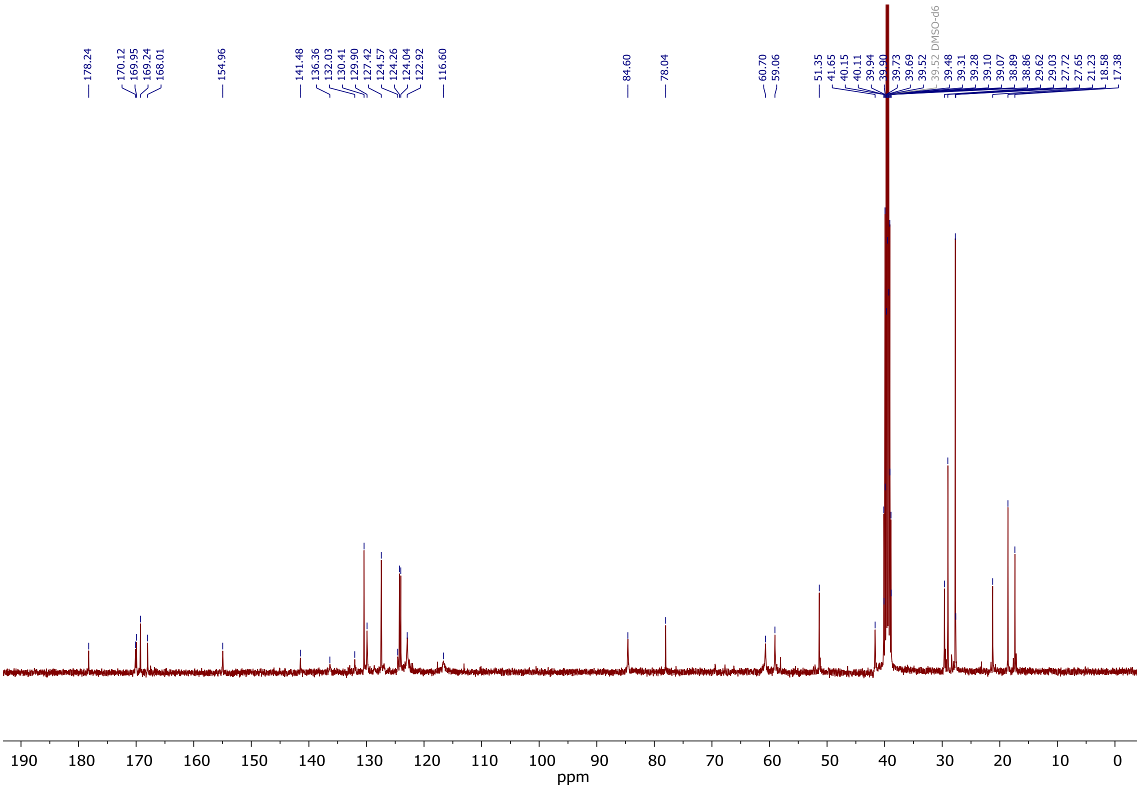

$^{13}C\{^{1}H\}$ NMR (100.62 MHz, DMSO-d_6, 343 K)

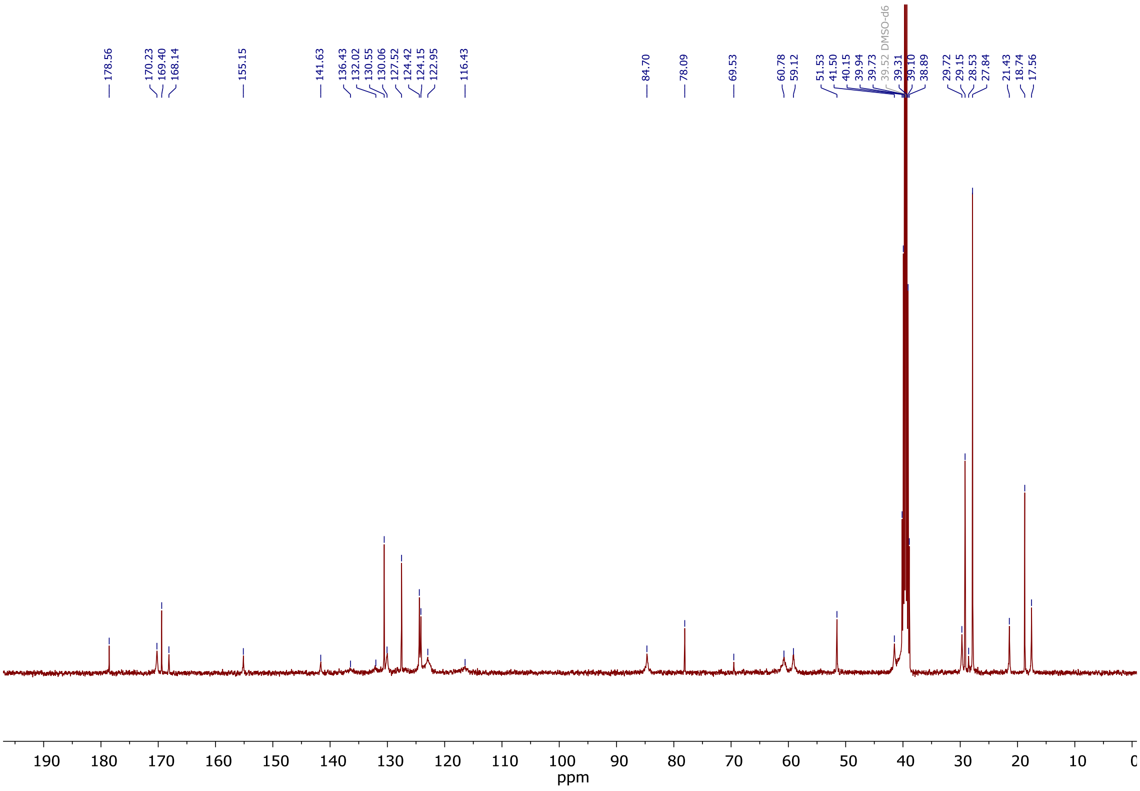

^{1}H NMR (400.16 MHz, DMSO-d_6, 353 K)

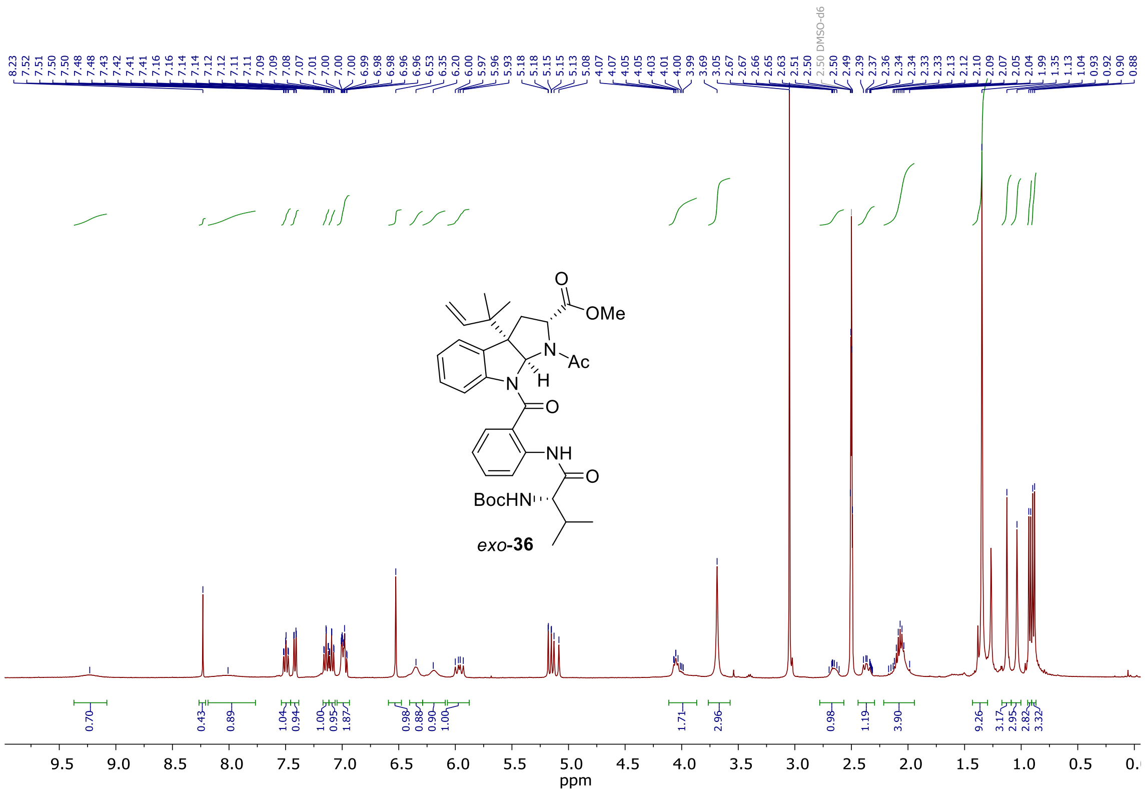

^{13}C{^{1}H} NMR (100.62 MHz, DMSO-d_6, 353 K)

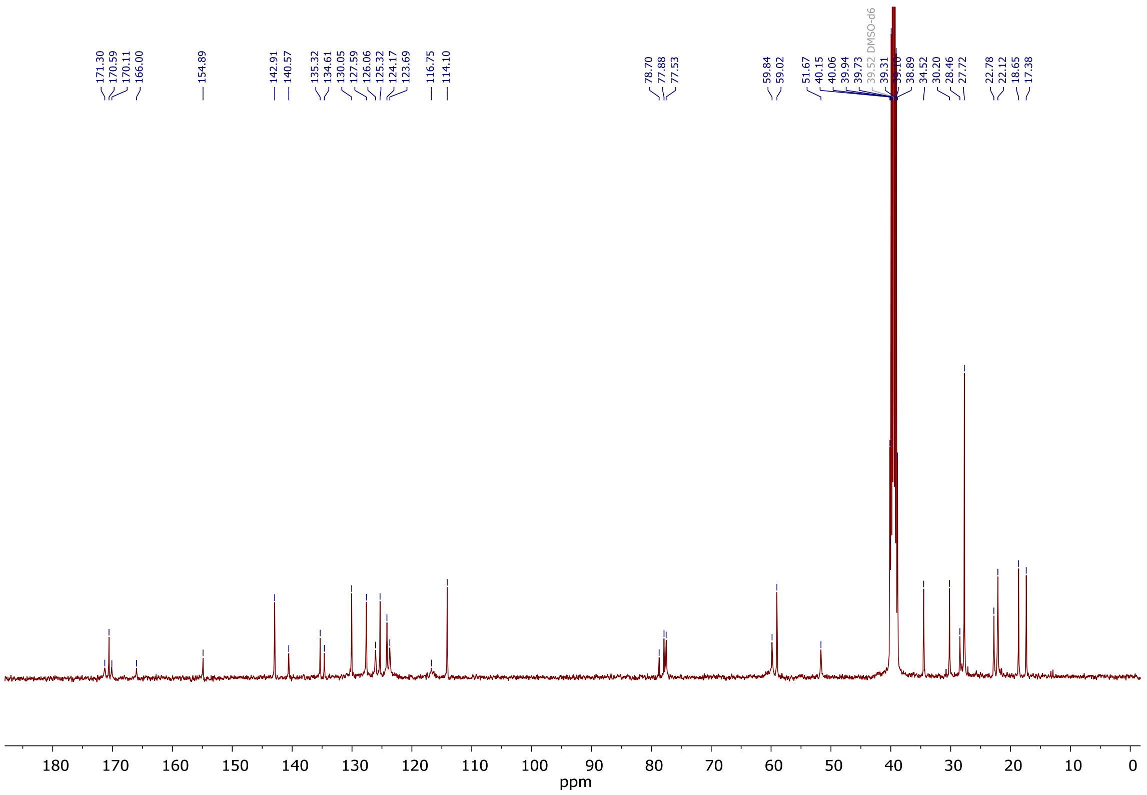

HPLC trace of the fraction of the column of *exo* isomer *exo*-**39** (Scharlau, C18 Kromaphase 100 Kromaphase 100, 5 µm, 250 x 4.6 mm, gradient from CH_3CN/H_2O 50% to CH_3CN 100% in 20 min, 1.0 mL/min).

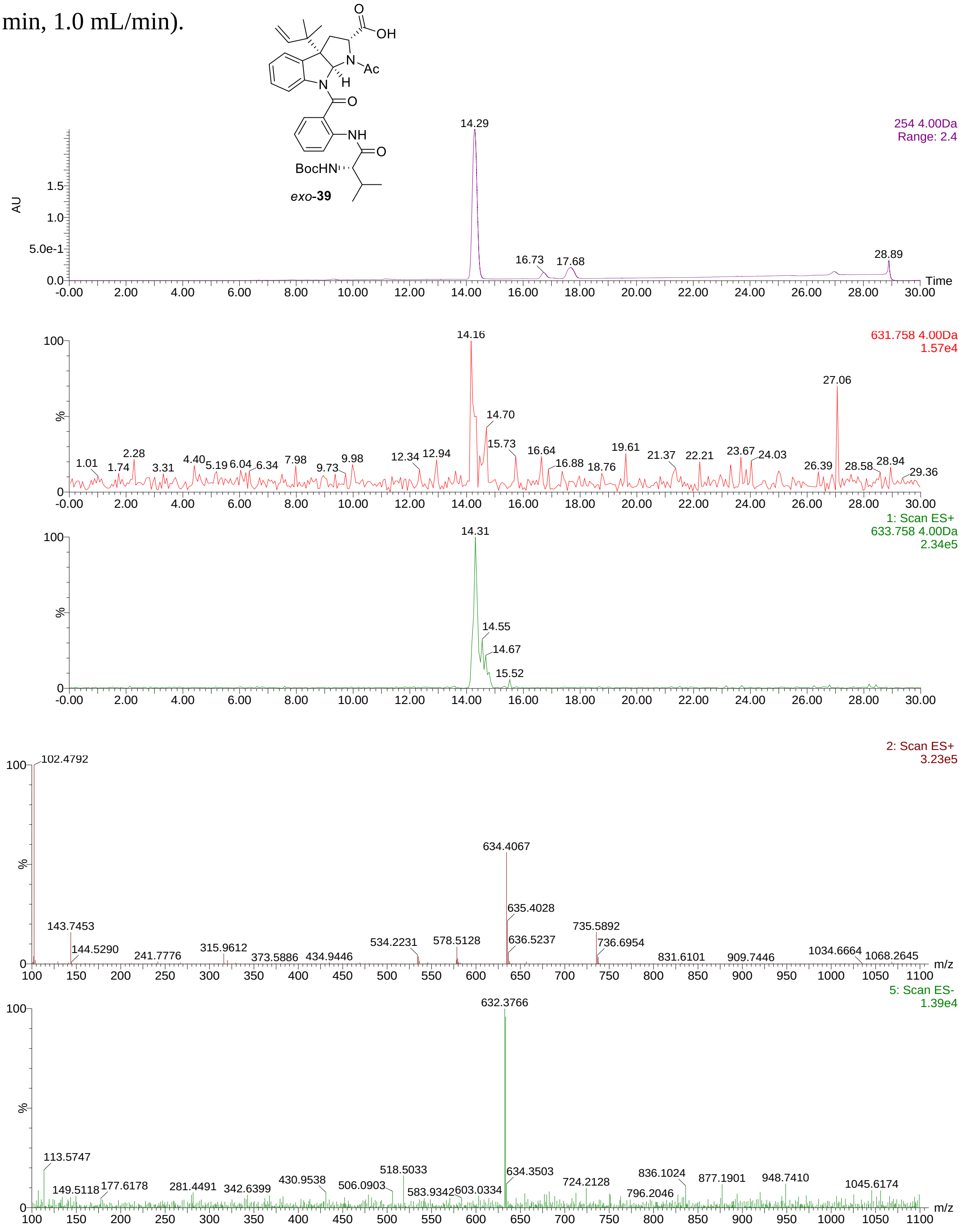

HPLC trace of the fraction of the column of *exo* isomer *exo*-**40** (Scharlau, C18 Kromaphase 100 Kromaphase 100, 5 µm, 250 x 4.6 mm, gradient from CH_3CN/H_2O 50% to CH_3CN 100% in 20 min, 1.0 mL/min).

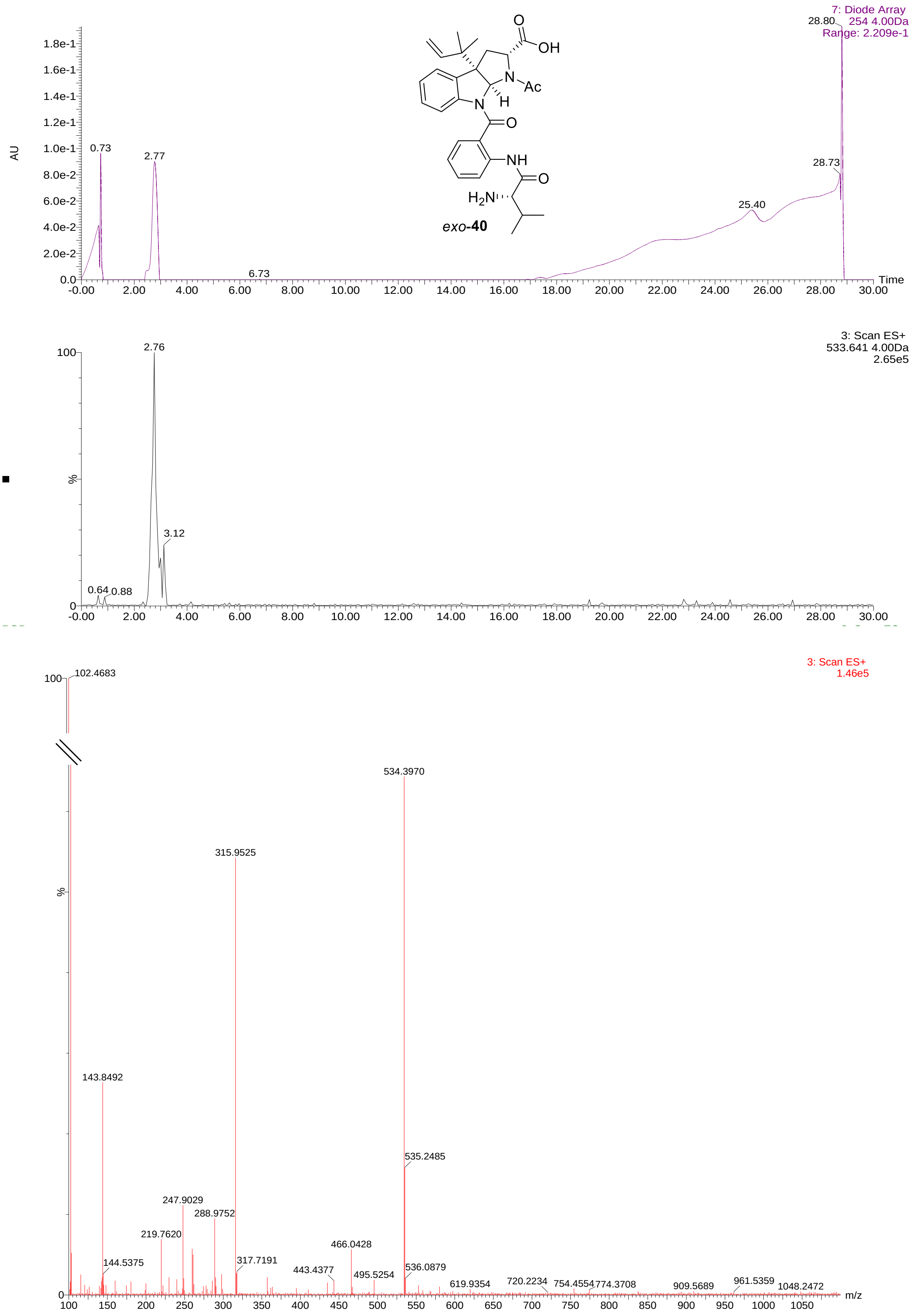

^{1}H NMR (400.16 MHz, $CDCl_3$)

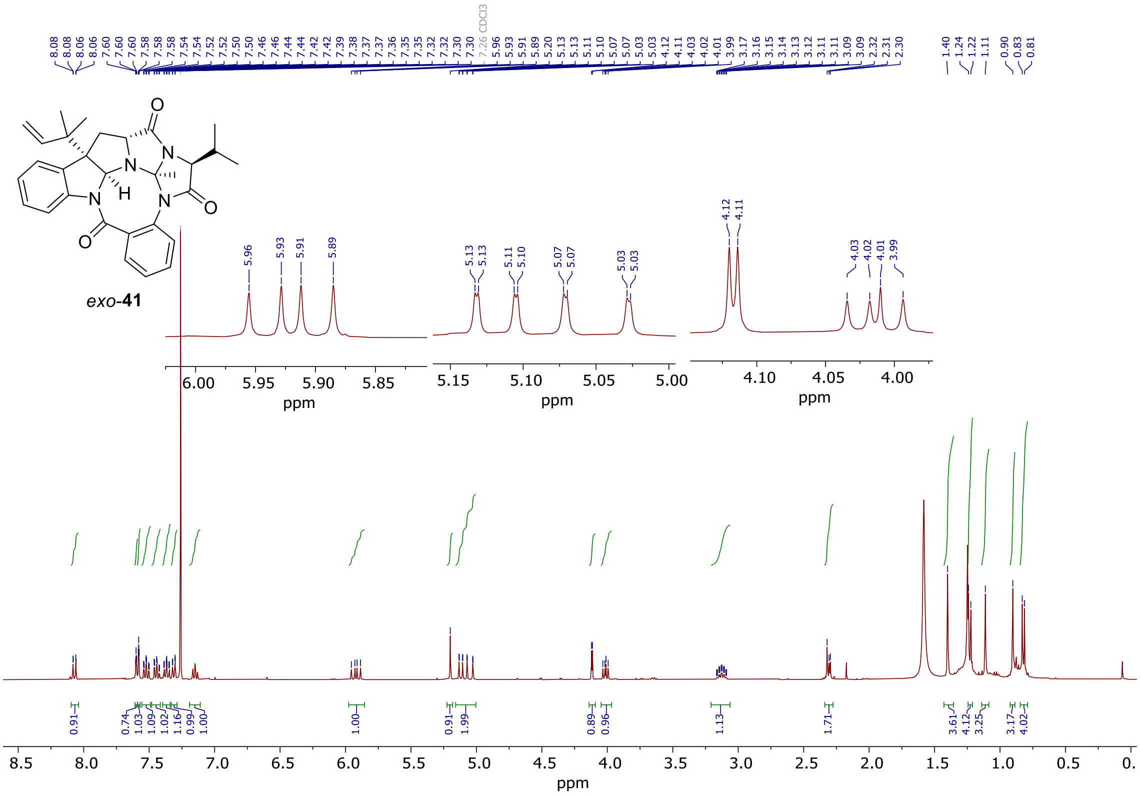

^{13}C{^{1}H} NMR (100.62 MHz, $CDCl_3$)

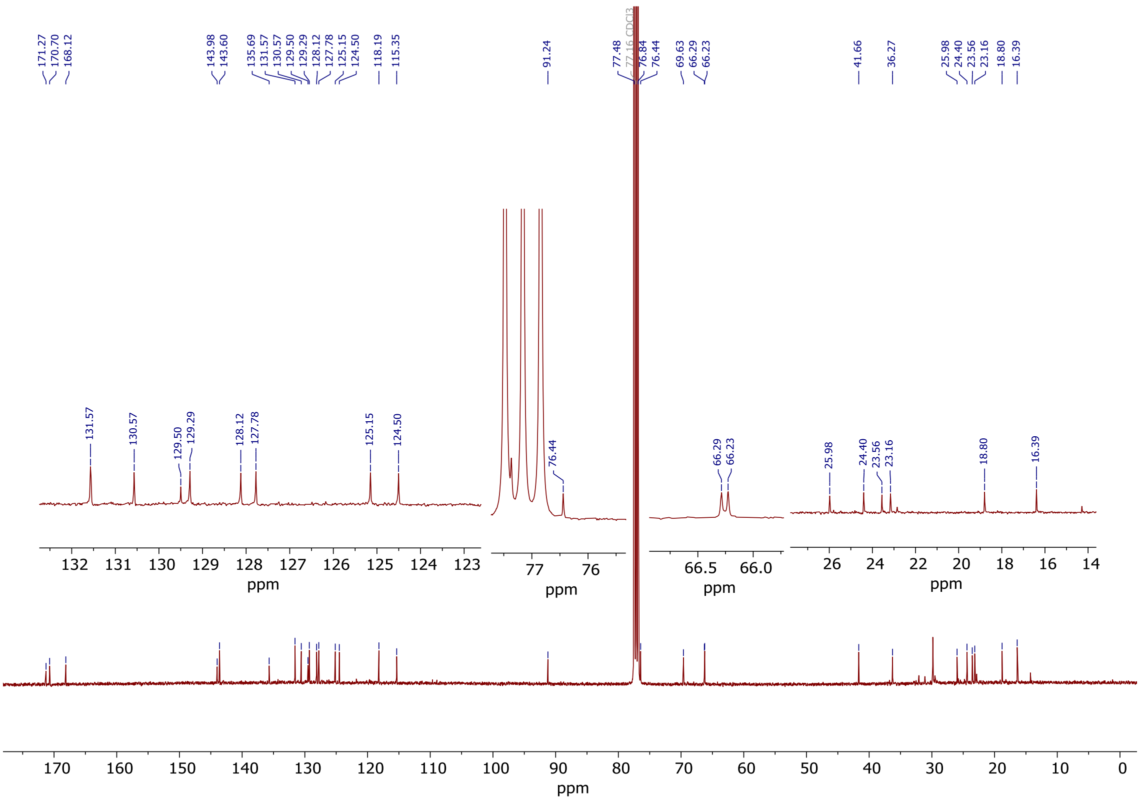

HSQC (100.62 MHz, $CDCl_3$)

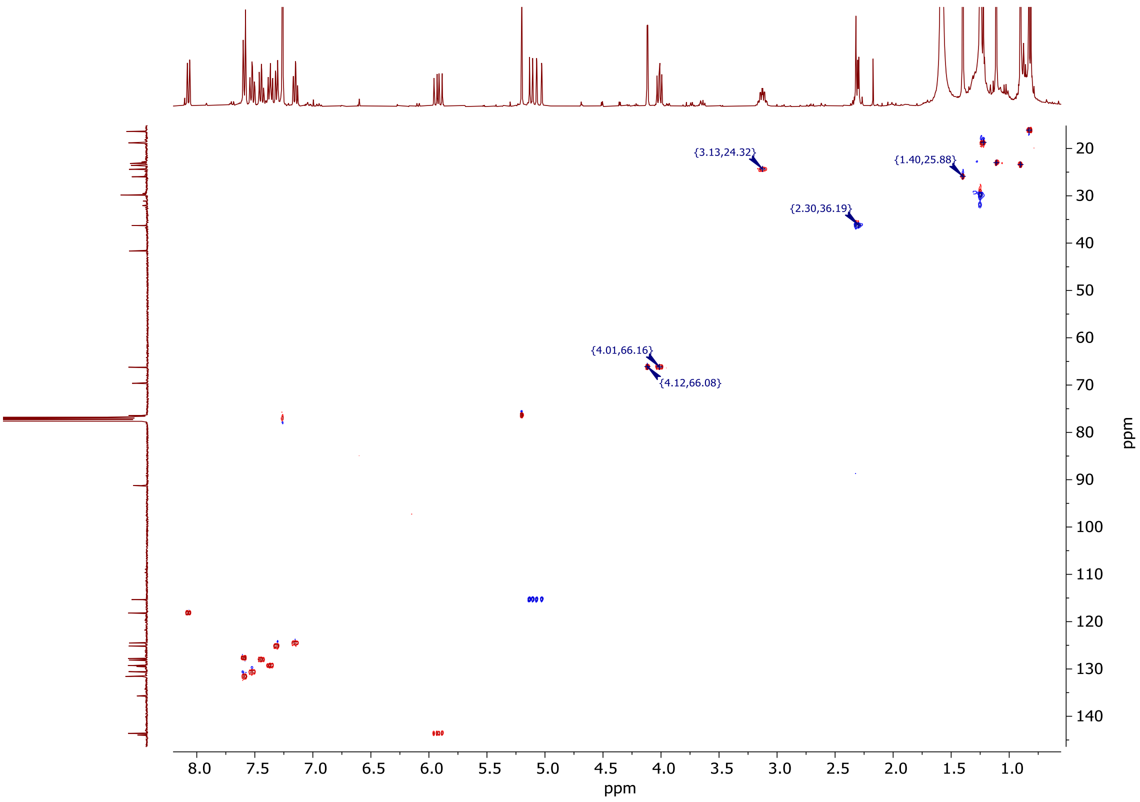

HMBC (100.62 MHz, $CDCl_3$)

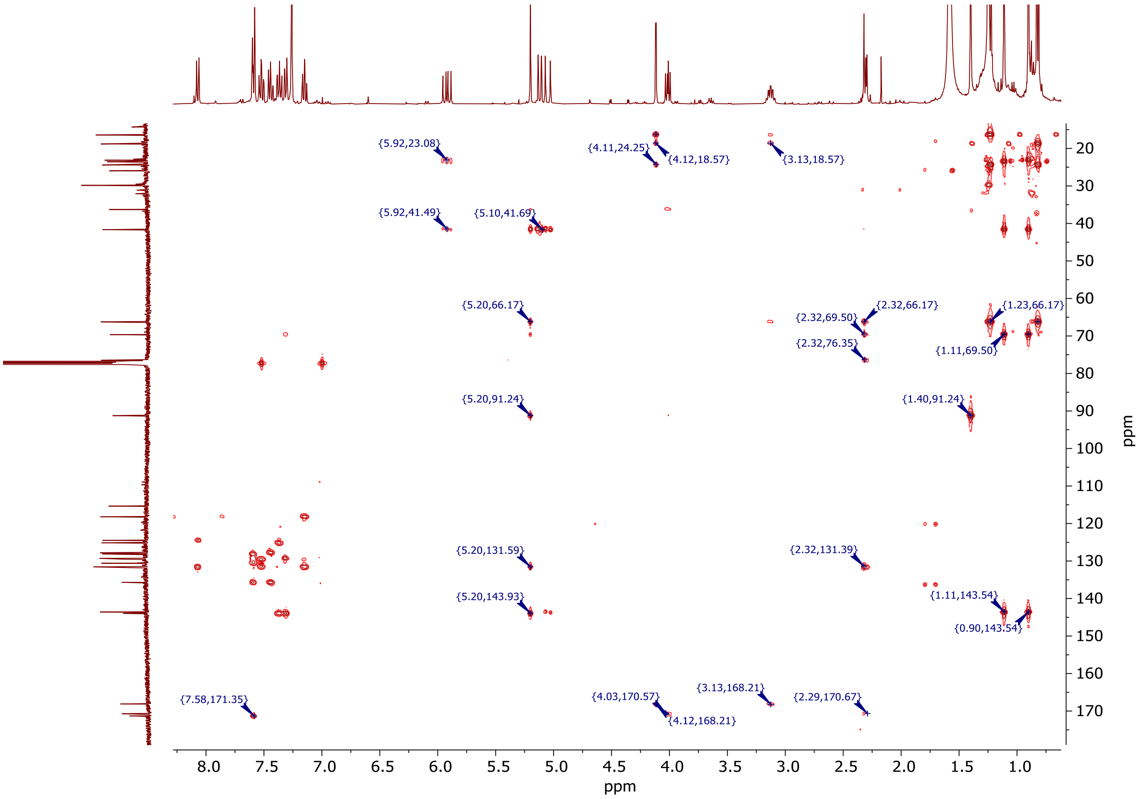

NOESY

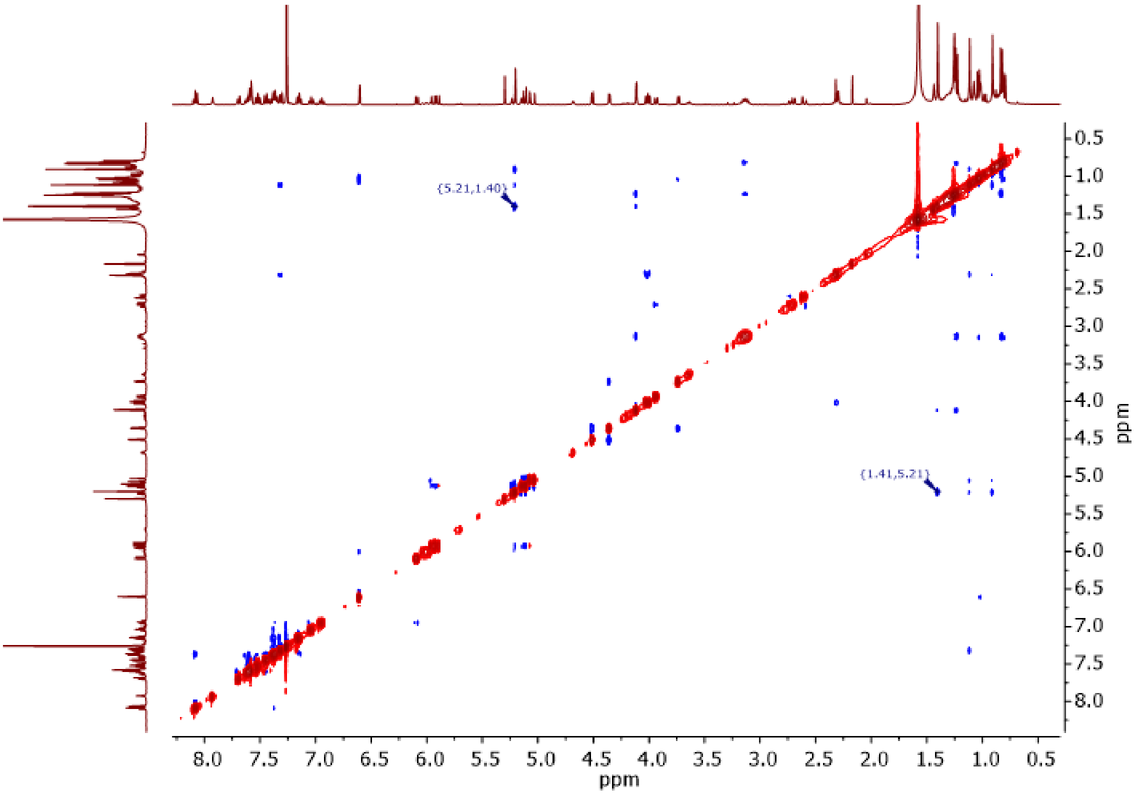

{5.21,1.40}
{1.41,5.21}
0.5
1.0
1.5
2.0
2.5
3.0
3.5
4.0
4.5
5.0
5.5
6.0
6.5
7.0
7.5
8.0
ppm
8.0
7.5
7.0
6.5
6.0
5.5
5.0
4.5
4.0
3.5
3.0
2.5
2.0
1.5
1.0
0.5
ppm

^{1}H NMR (400.16 MHz, DMSO-d_6, 343 K)

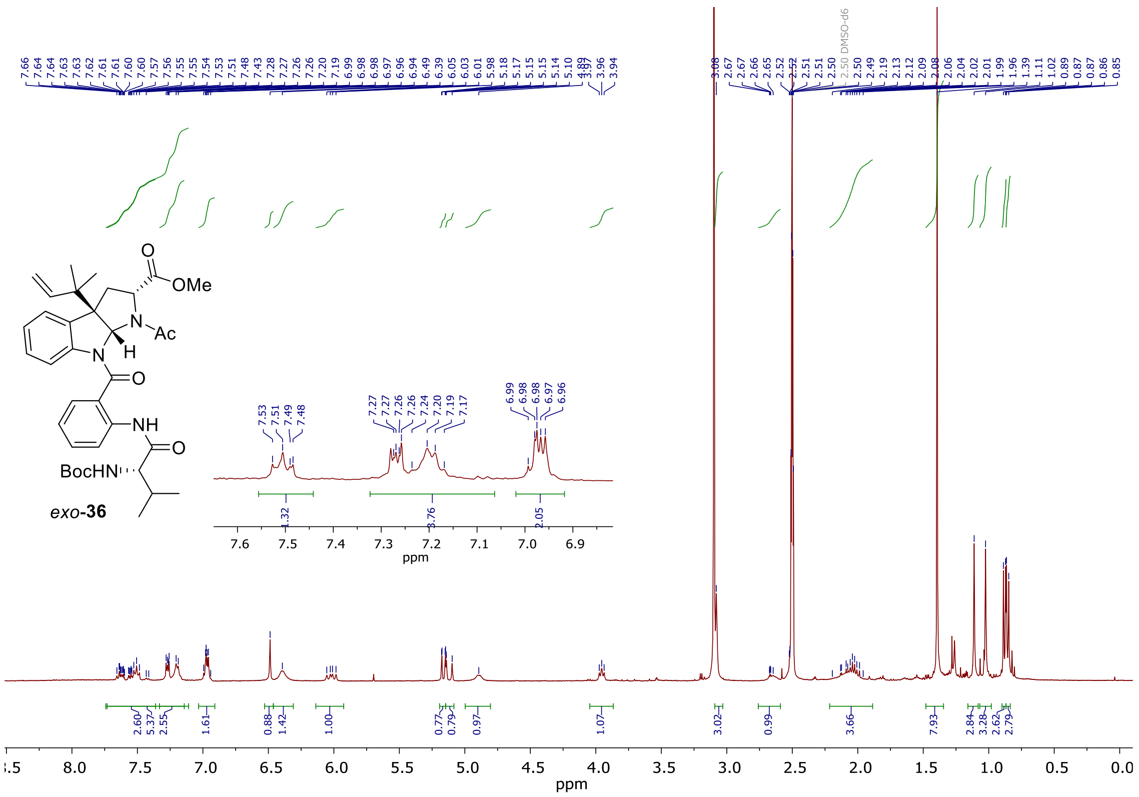

exo-**36**

$^{13}C\{^{1}H\}$ NMR (100.62 MHz, DMSO-d_6, 343 K)

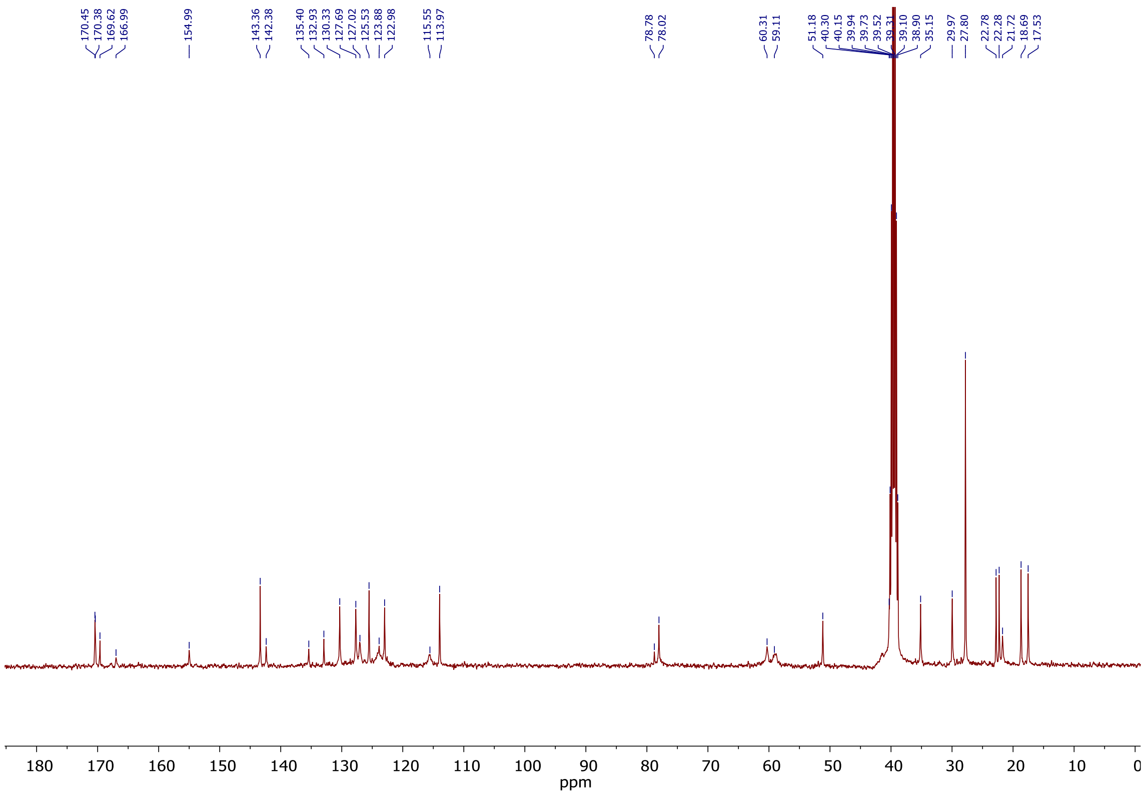

^{1}H NMR (400.16 MHz, $CDCl_3$)

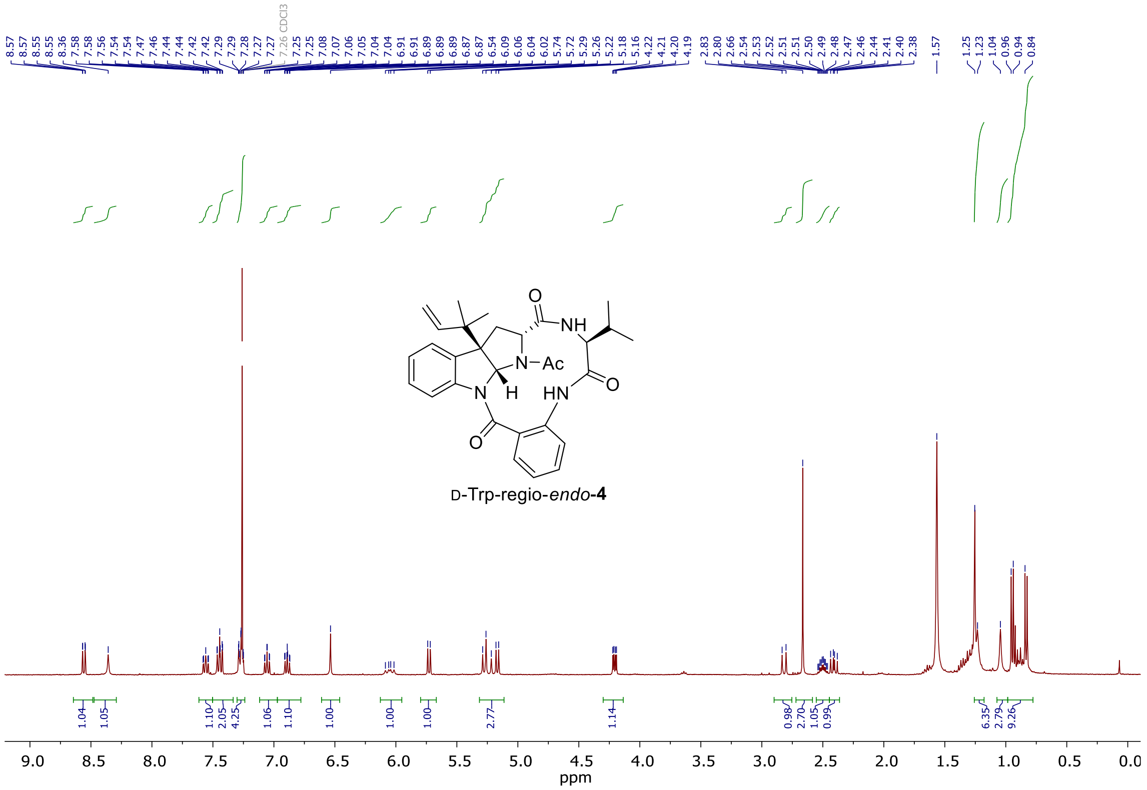

$^{13}C\{^{1}H\}$ NMR (100.62 MHz, $CDCl_3$)

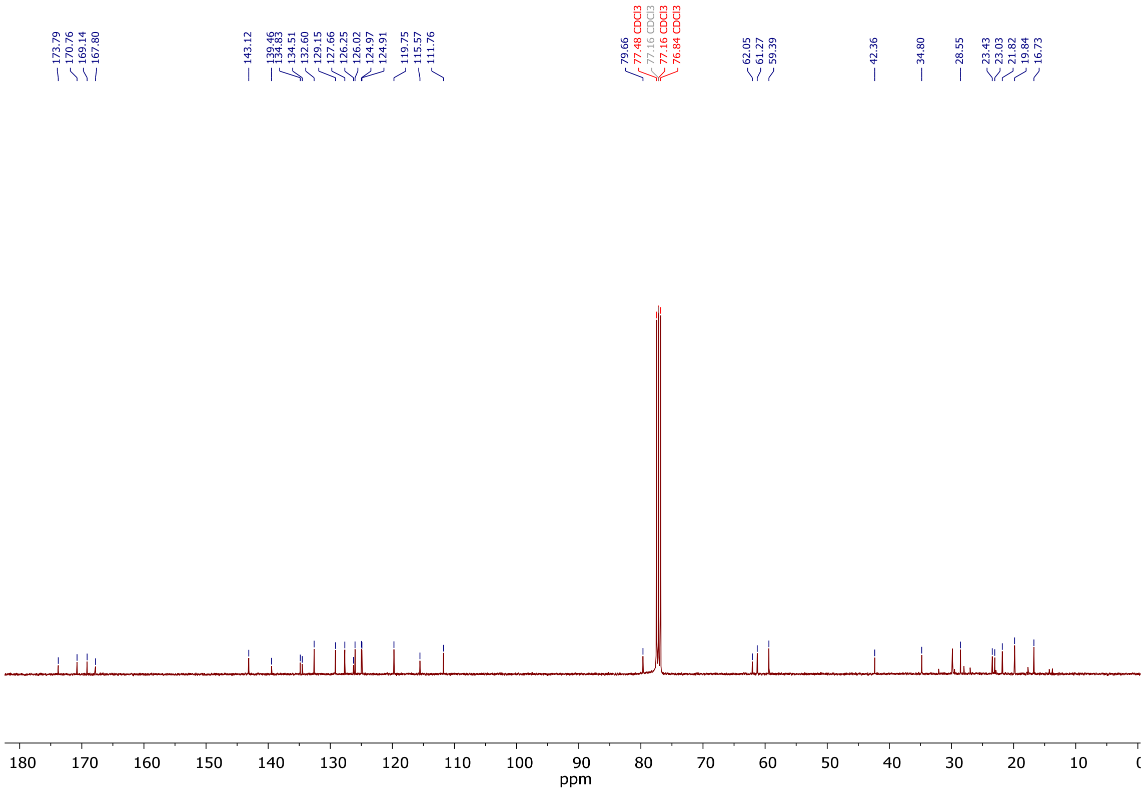

DEPT-135 (100.62 MHz, $CDCl_3$)

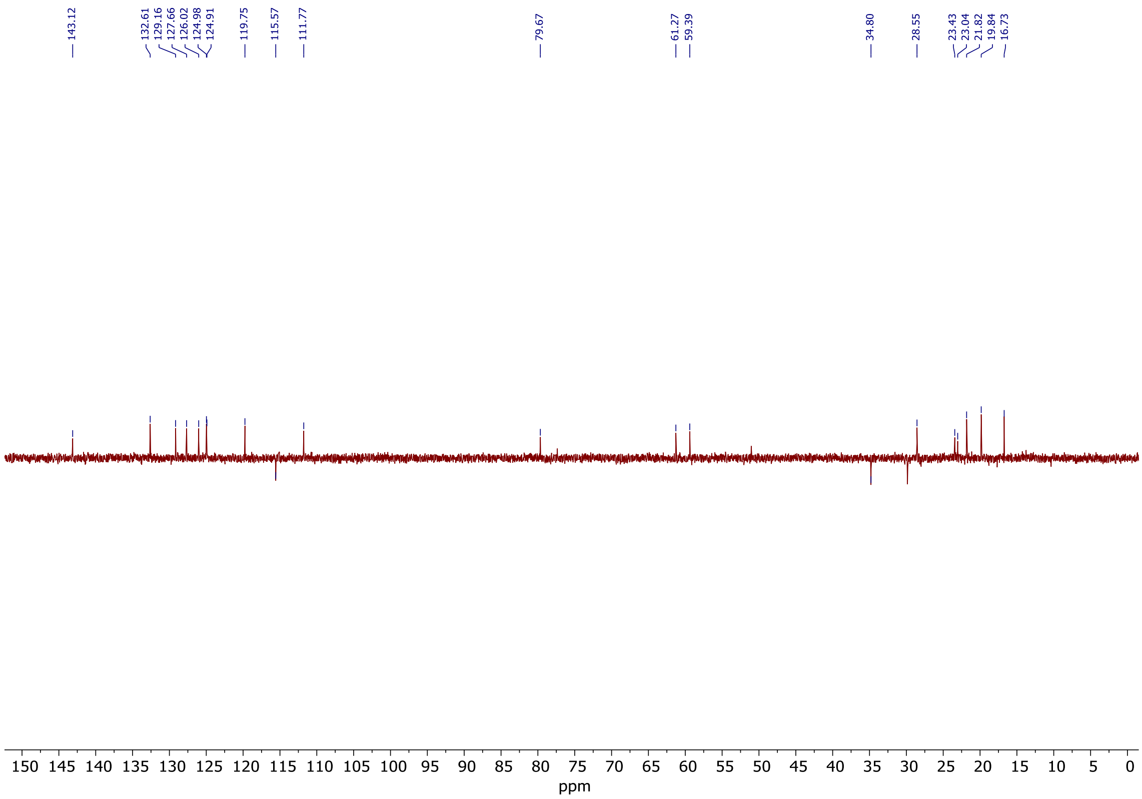

HSQC (100.62 MHz, $CDCl_3$)

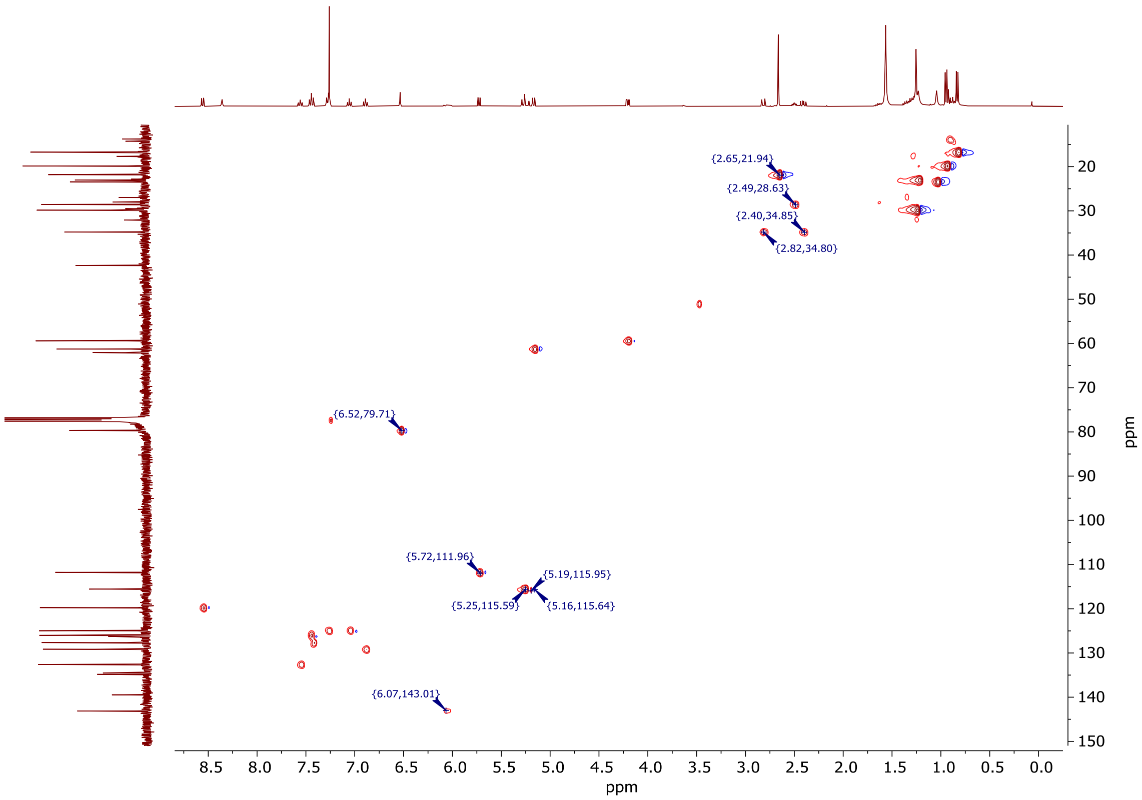

HMBC (100.62 MHz, $CDCl_3$)

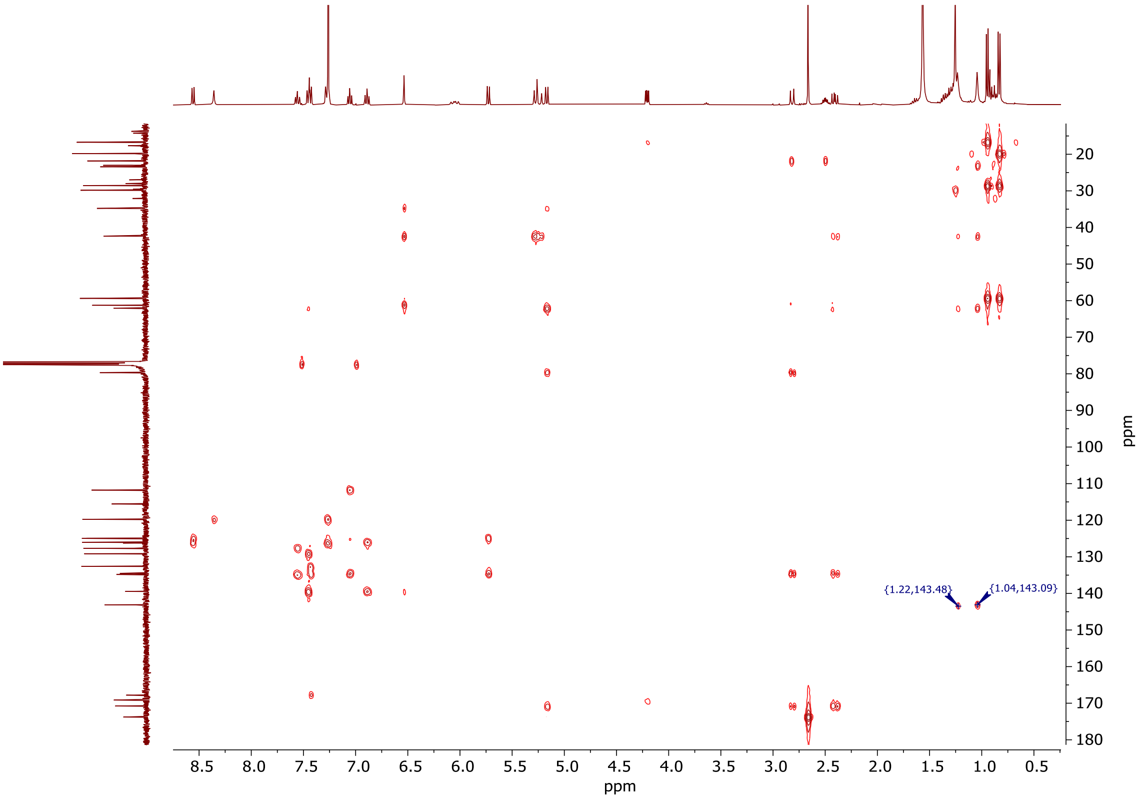

COSY (100.62 MHz, $CDCl_3$)

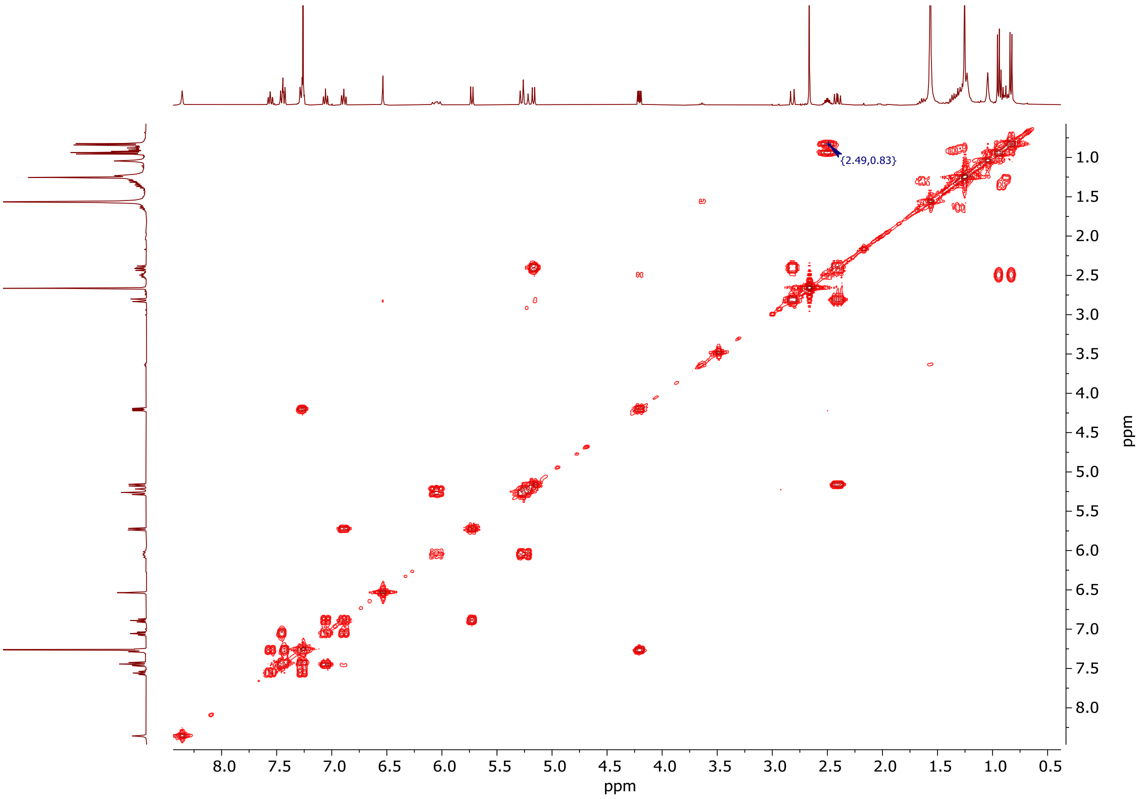

^{1}H NMR (400.16 MHz, DMSO-d_6, 343 K)

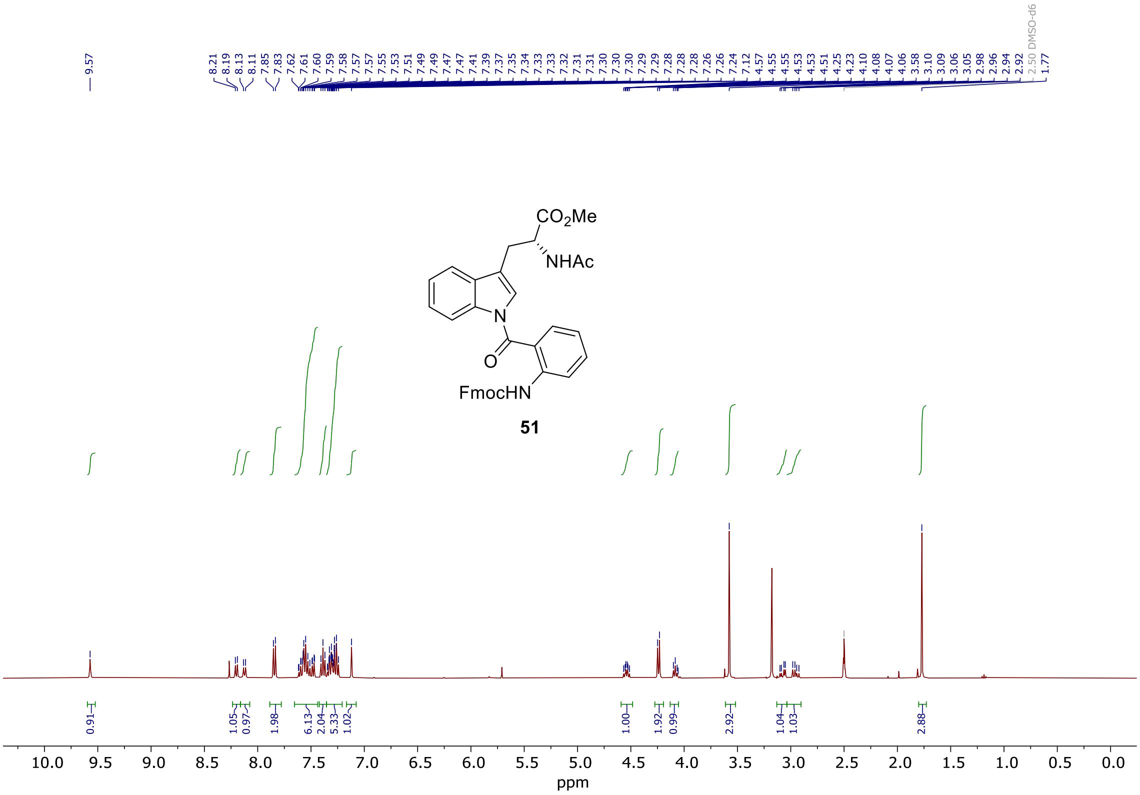

^{13}C{^{1}H} NMR (100.62 MHz, DMSO-d_6, 343 K)

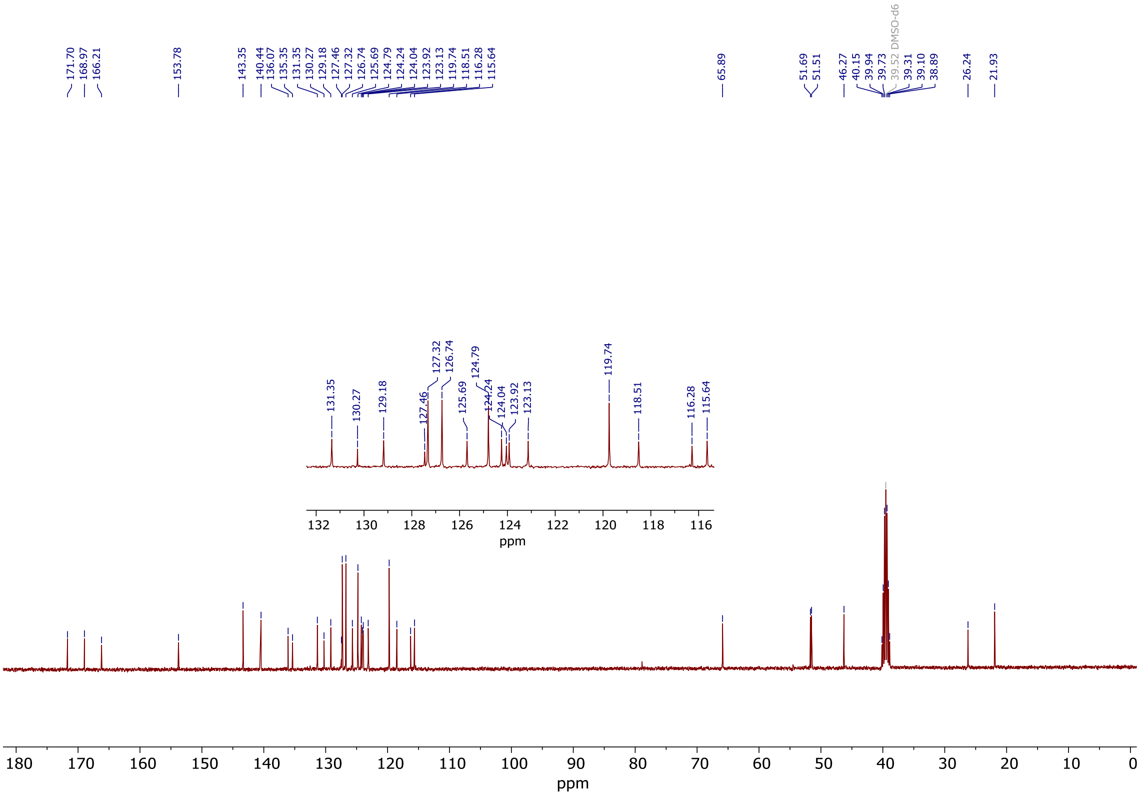

^{1}H NMR (400.16 MHz, DMSO-d_6, 343 K)

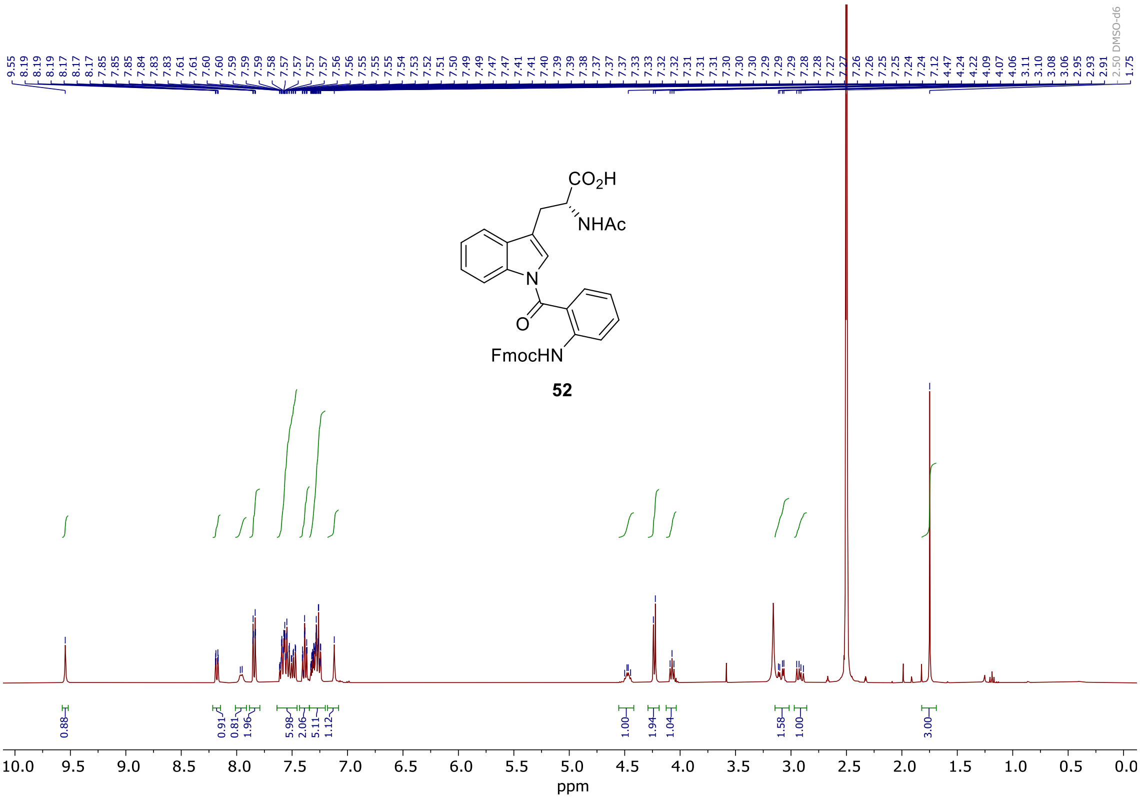

52

^{13}C{^{1}H} NMR (100.62 MHz, DMSO-d_6, 343 K)

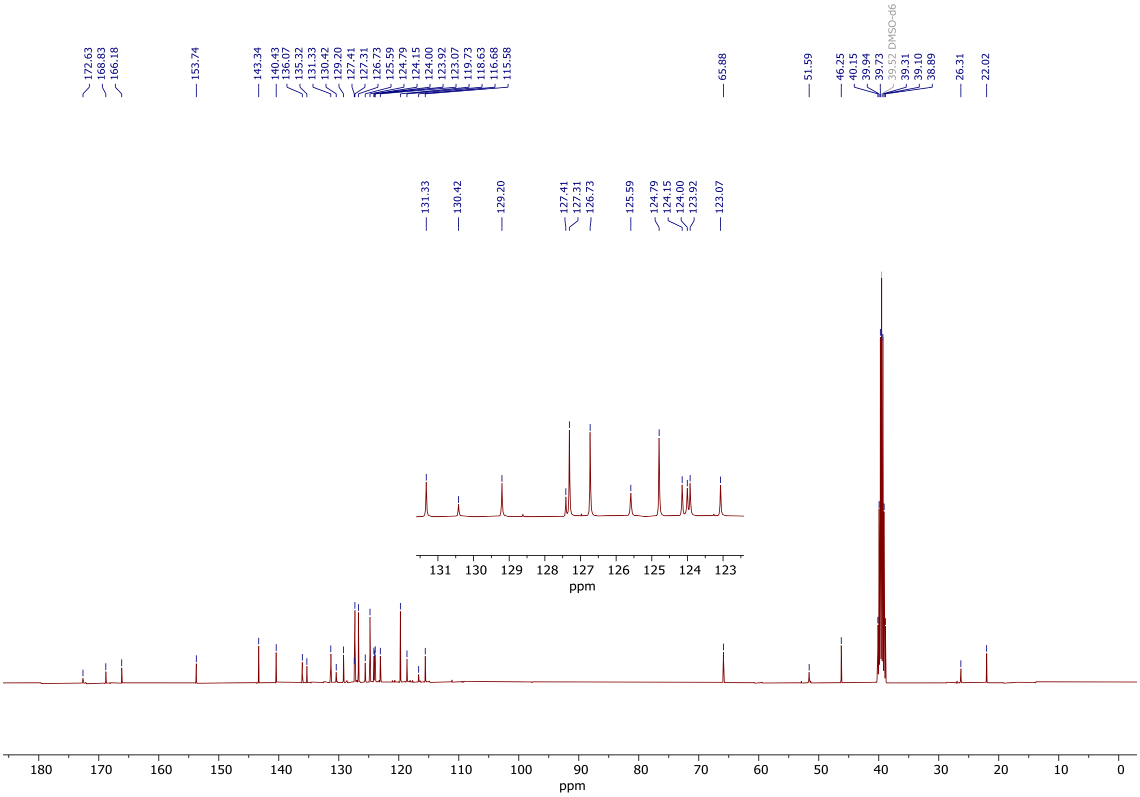

^{1}H NMR (400.16 MHz, DMSO-d_6, 343 K)

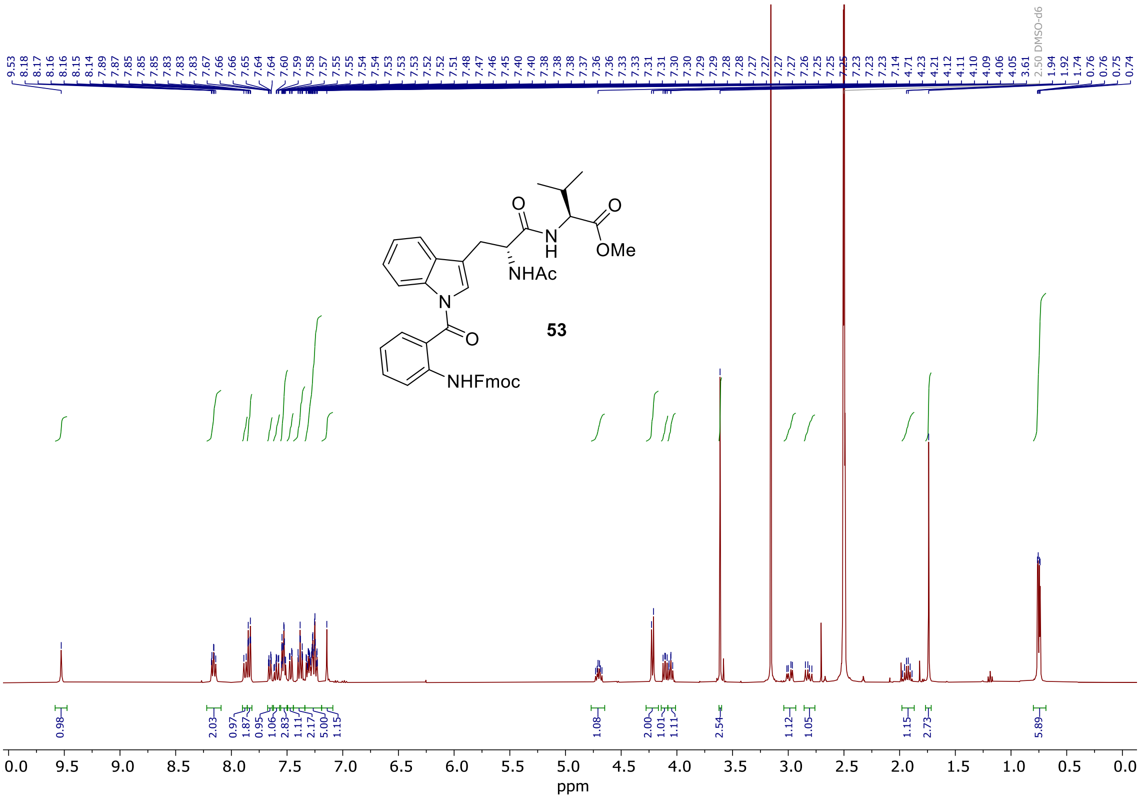

^{13}C{^{1}H} NMR (100.62 MHz, DMSO-d_6, 343 K)

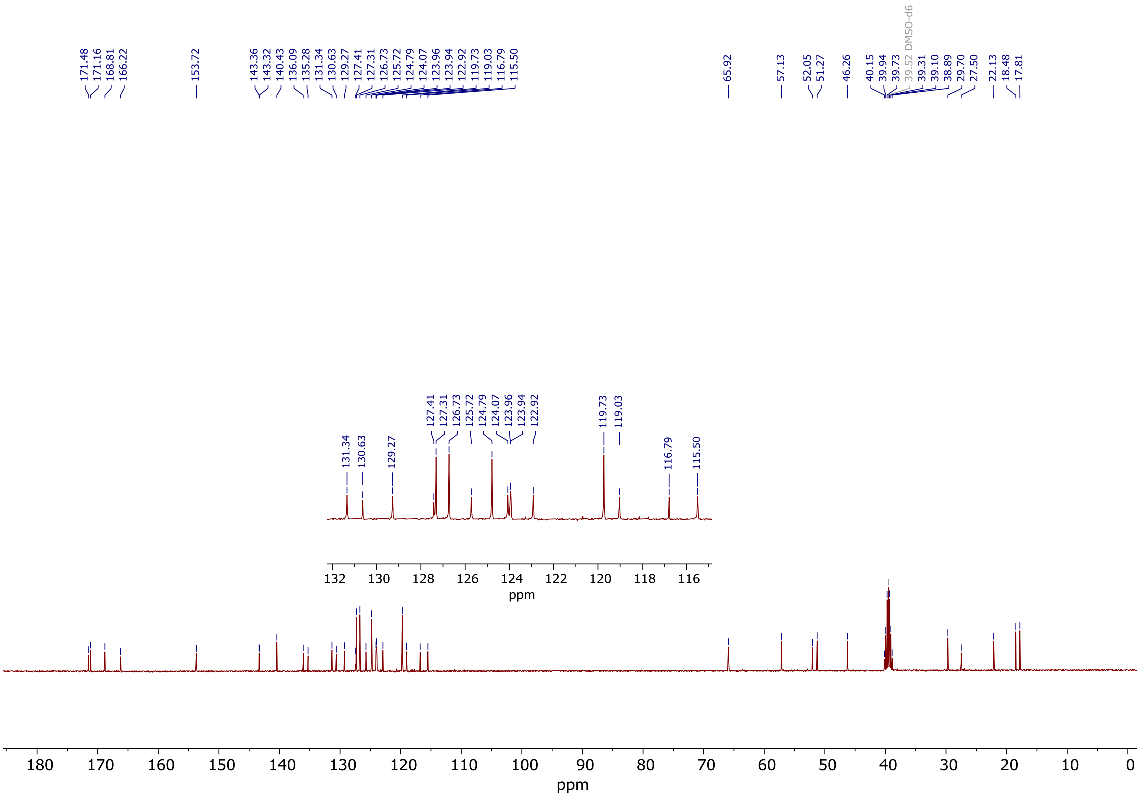

^{1}H NMR (400.16 MHz, DMSO-d_6, 343 K)

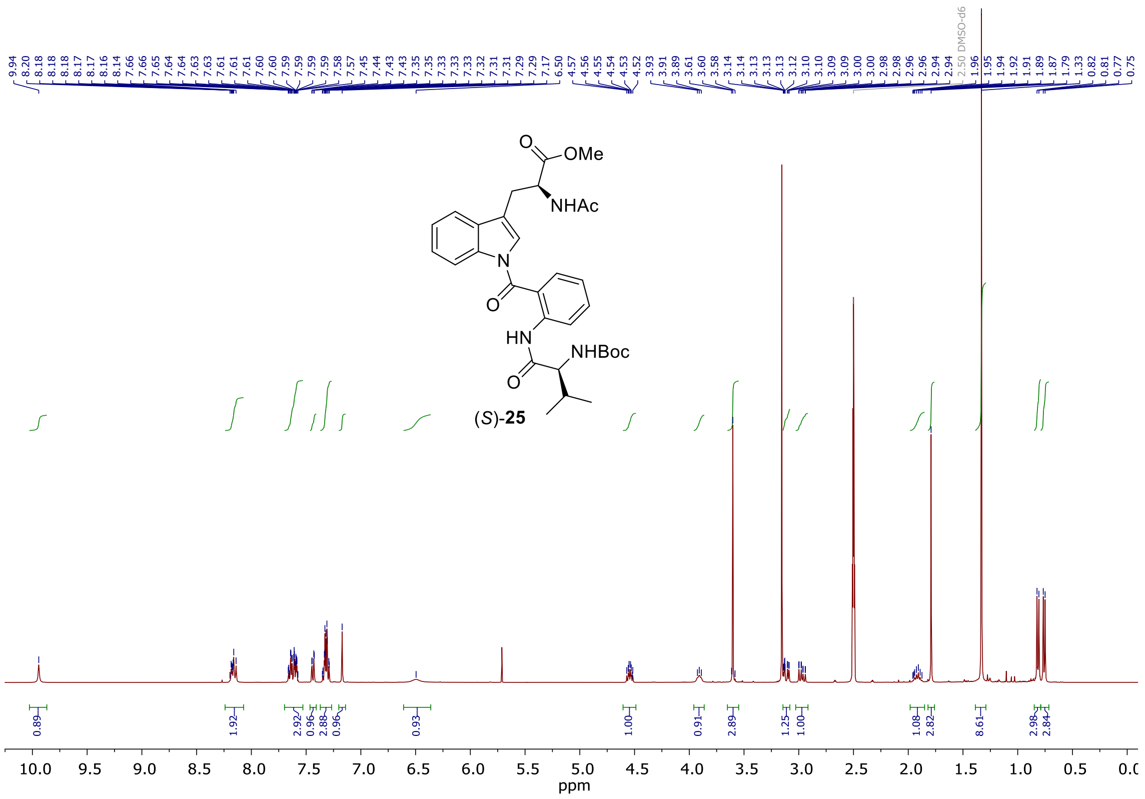

$^{13}C\{^{1}H\}$ NMR (100.62 MHz, DMSO-d_6, 343 K)

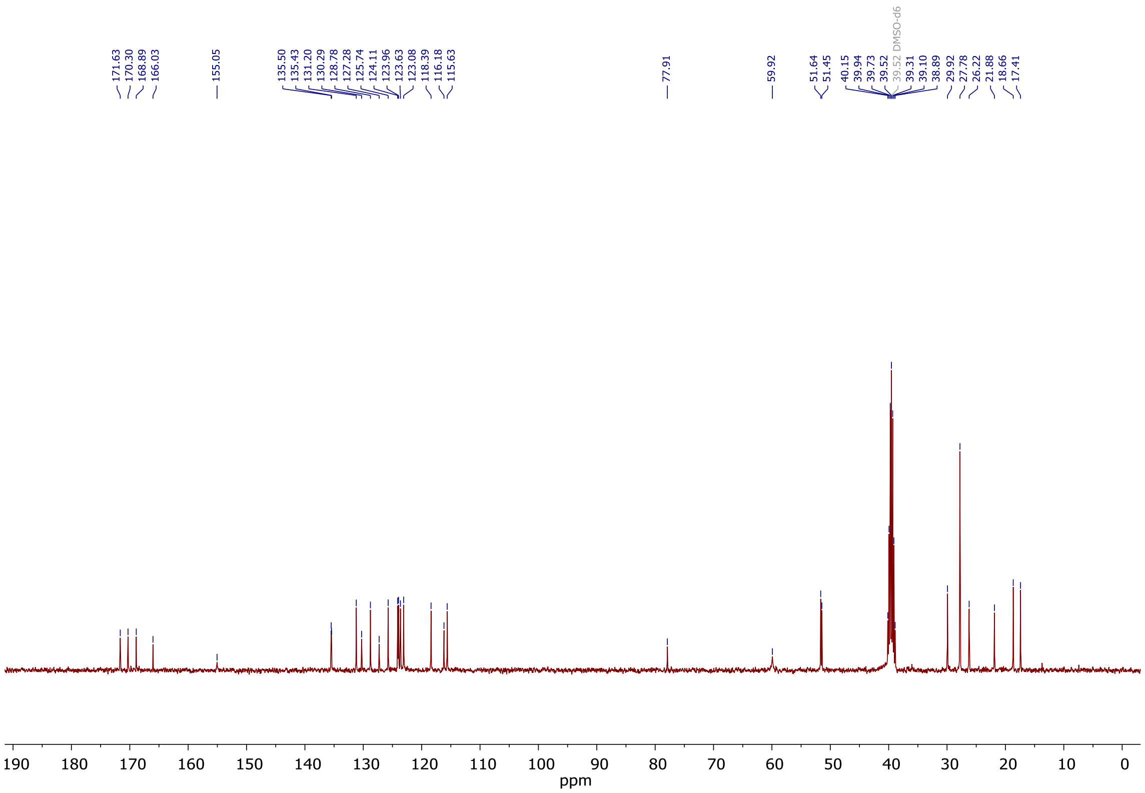

^{1}H NMR (400.16 MHz, DMSO-d_6, 343 K)

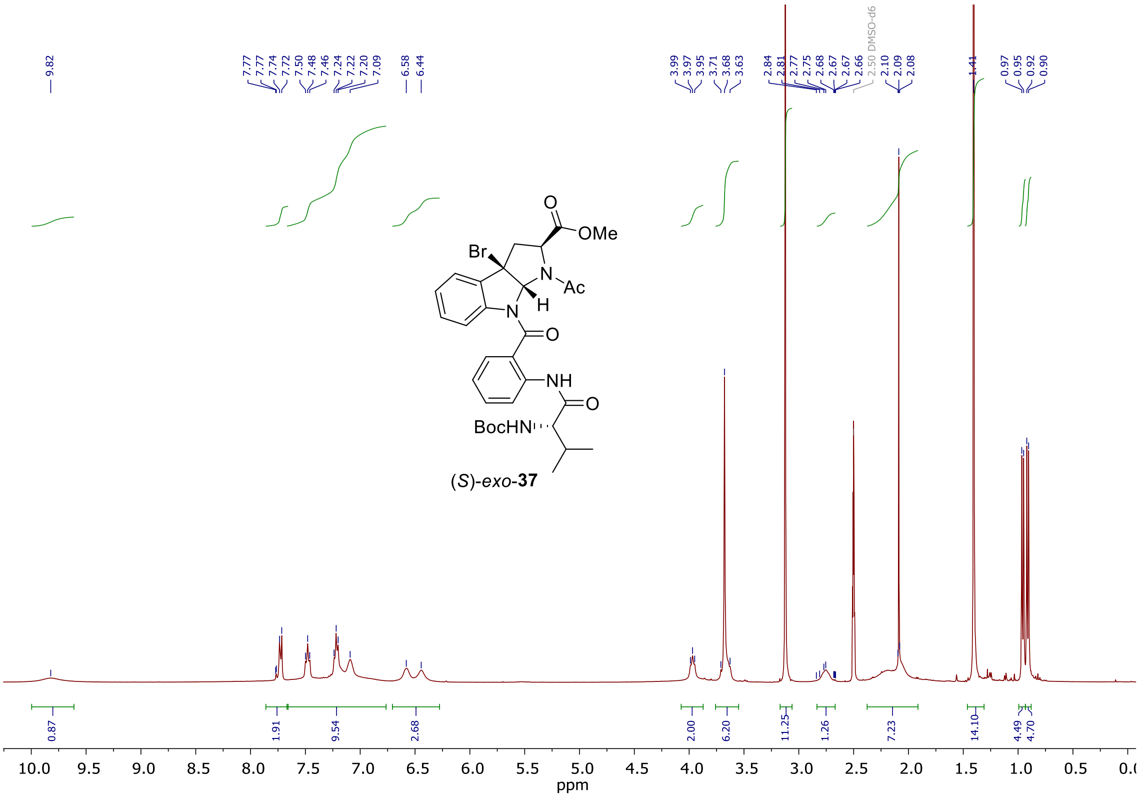

^{13}C{^{1}H} NMR (100.62 MHz, DMSO-d_6, 343 K)

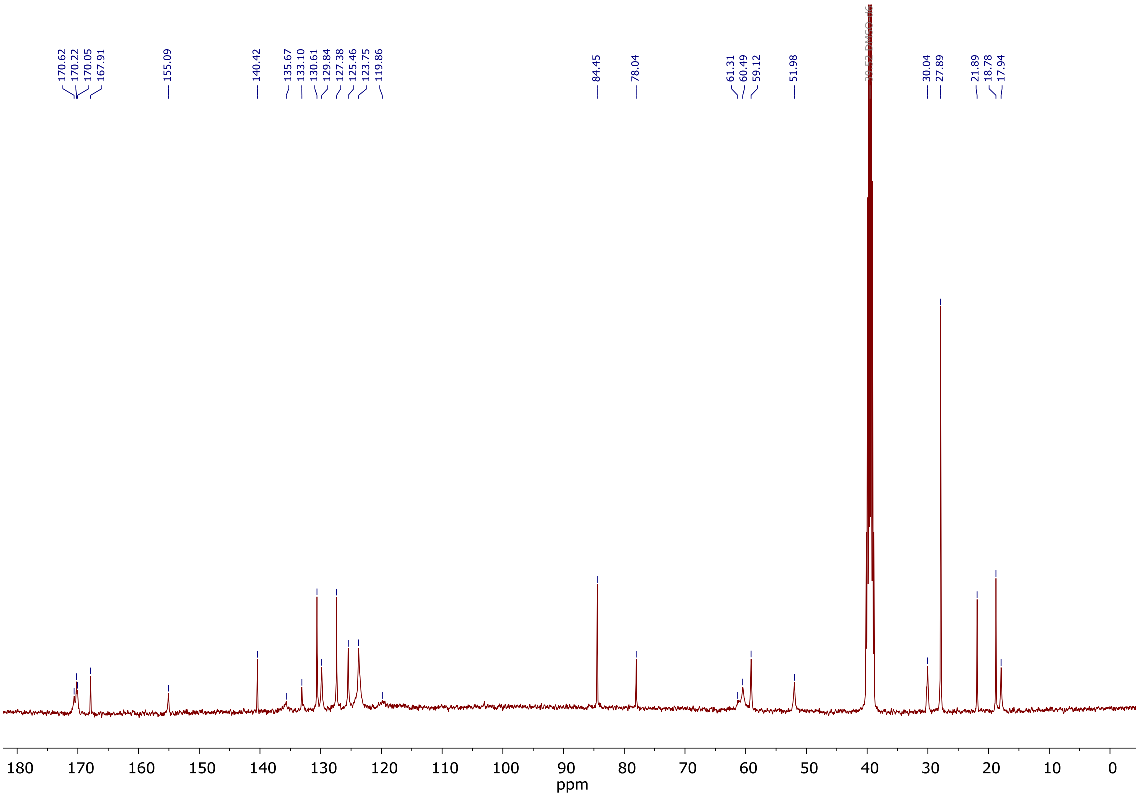

^{1}H NMR (400.16 MHz, DMSO-d_6, 343 K)

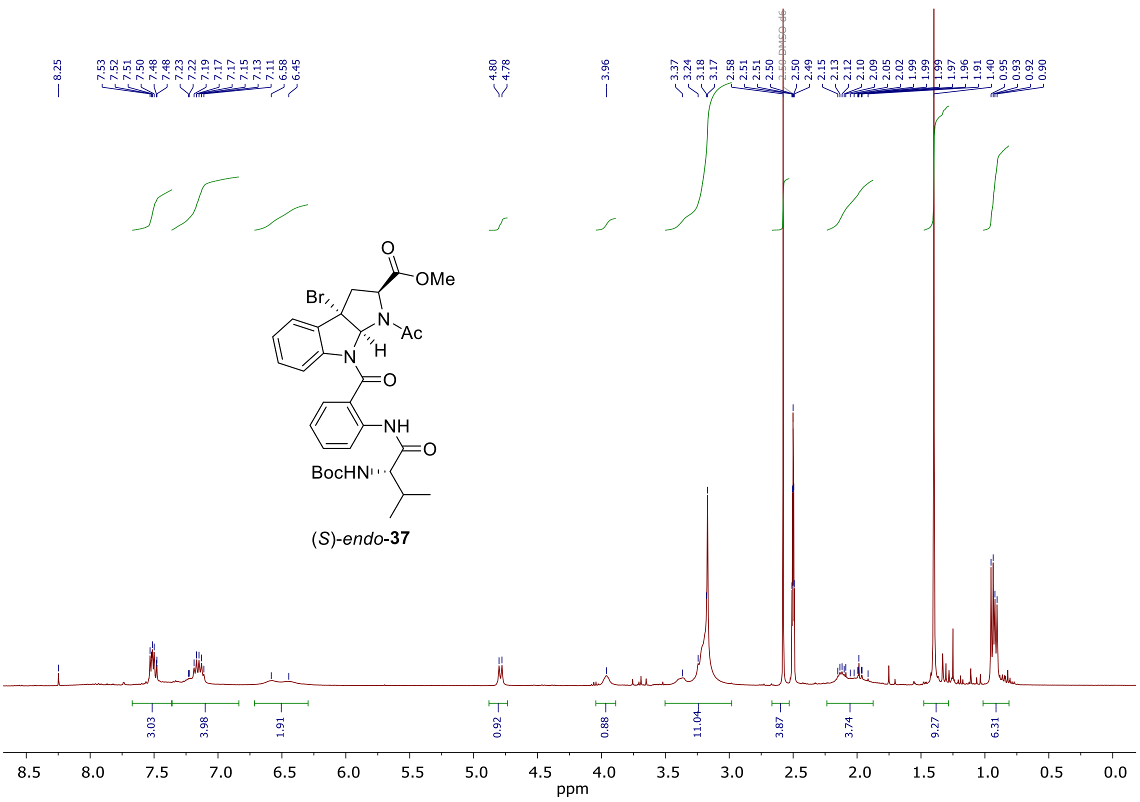

(*S*)-*endo*-**37**

$^{13}C\{^{1}H\}$ NMR (100.62 MHz, DMSO-d_6, 343 K)

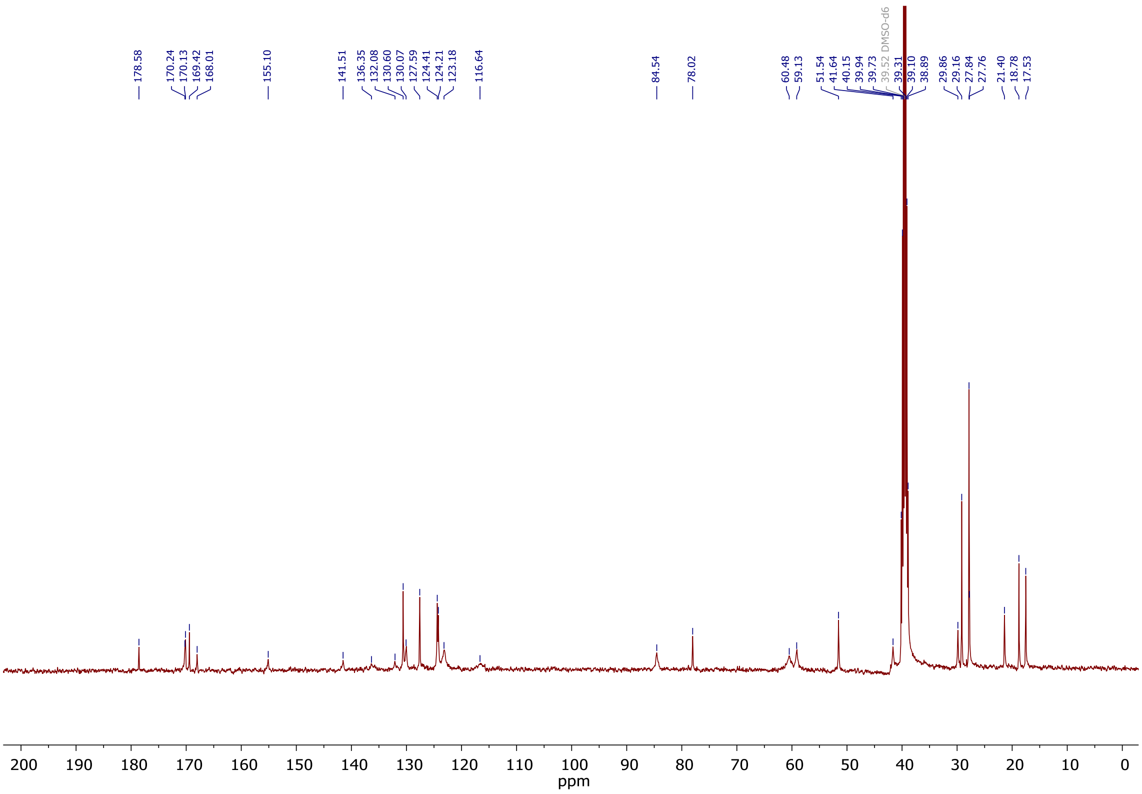

HPLC trace of a ~1:1 mixture of diastereomers (*R*)-*exo*-**37** and (*R*)-*endo*-**37** (from D-trp and L-val). Different conditions and columns were attempted, but no separation of the peaks was observed. Conditions used in the chromatogram shown below: Scharlau, C18 Kromaphase 100, 5 μm, 250 x 4.6 mm, gradient from CH_3CN/H_2O 30% to CH_3CN 100% in 20 min, 1.0 mL/min.

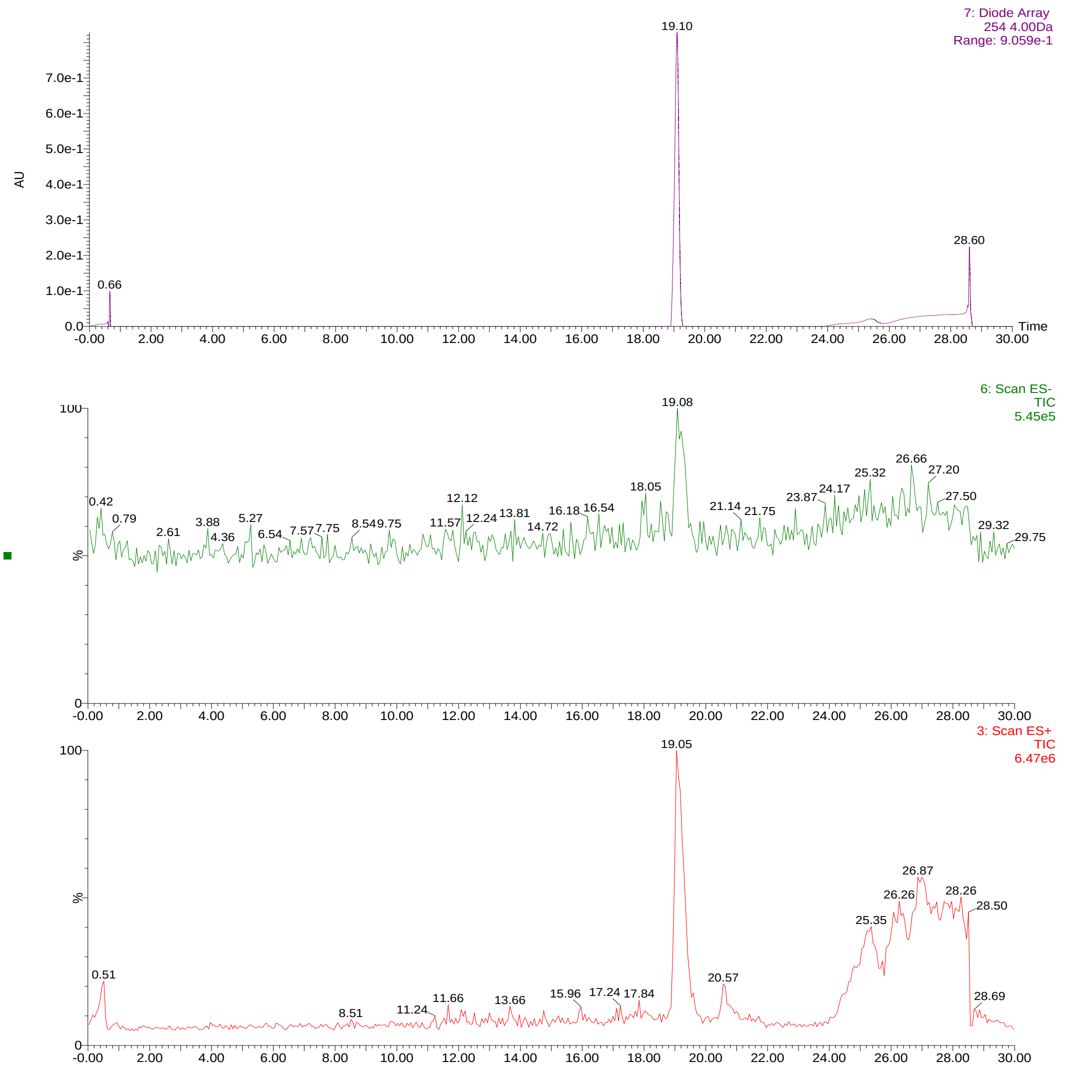

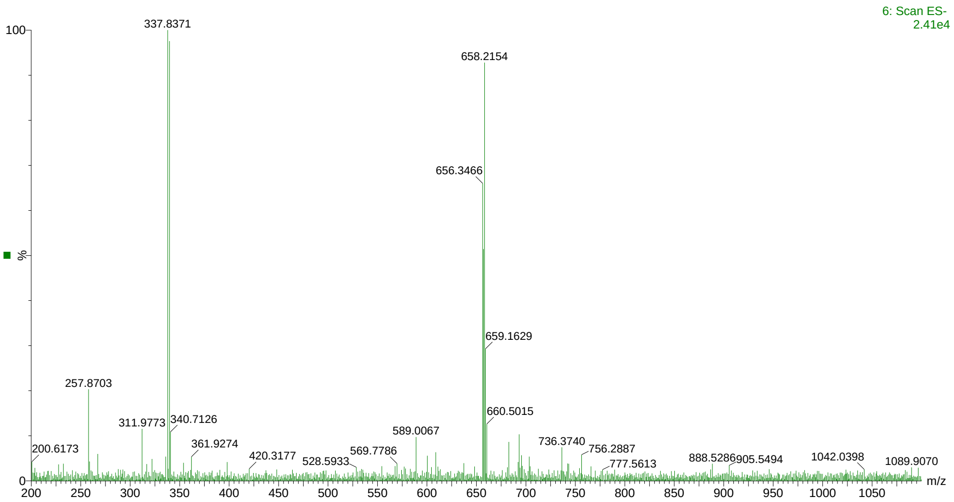

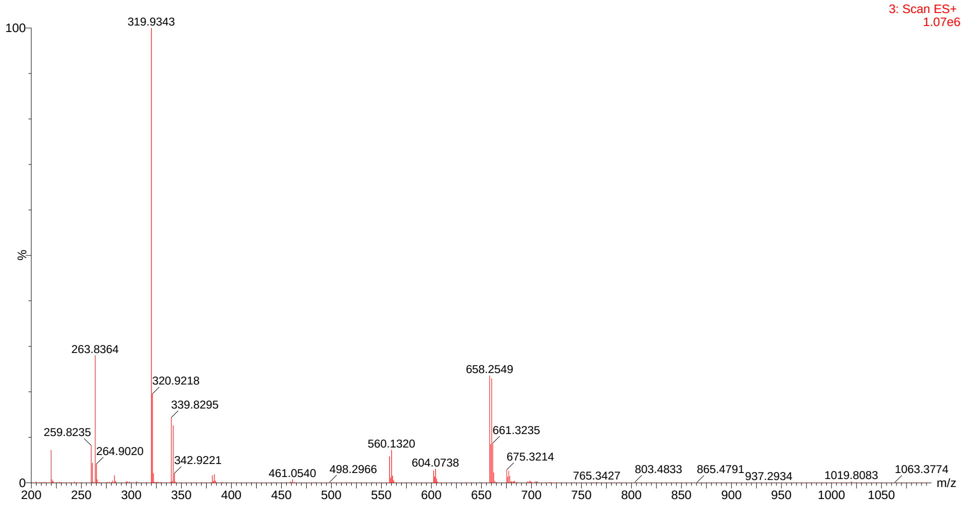

HPLC trace of a ~1:1 mixture of diastereomers (*S*)-*exo*-**37**and (*S*)-*endo*-**37** (from L-trp and L-val). Conditions used in the chromatogram shown below: Scharlau, C18 Kromaphase 100, 5 μm, 250 x 4.6 mm, gradient from CH_3CN/H_2O 30% to CH_3CN 100% in 20 min, 1.0 mL/min. In this case, separation of both diastereomers was observed.

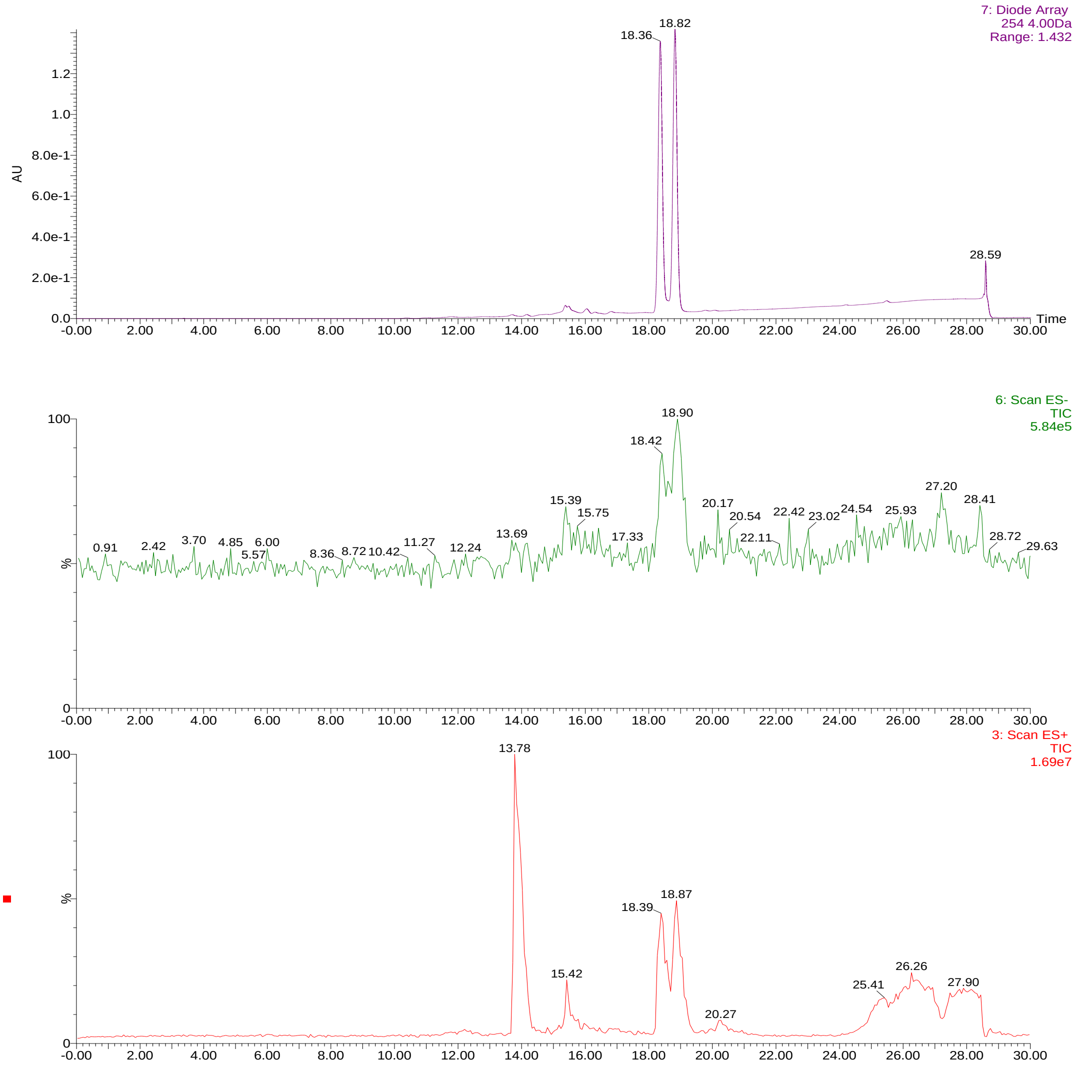

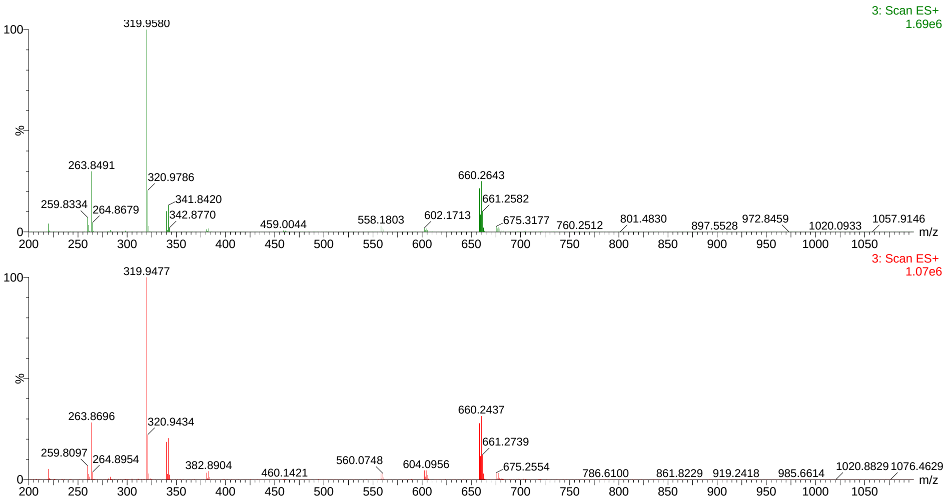

HPLC trace of a ~1:1:1:1 mixture of diastereomers (*R*)-*exo*-**37**/(*R*)-*endo*-**37**/(*S*)-*exo*-**37**/(*S*)-*endo*-**37**

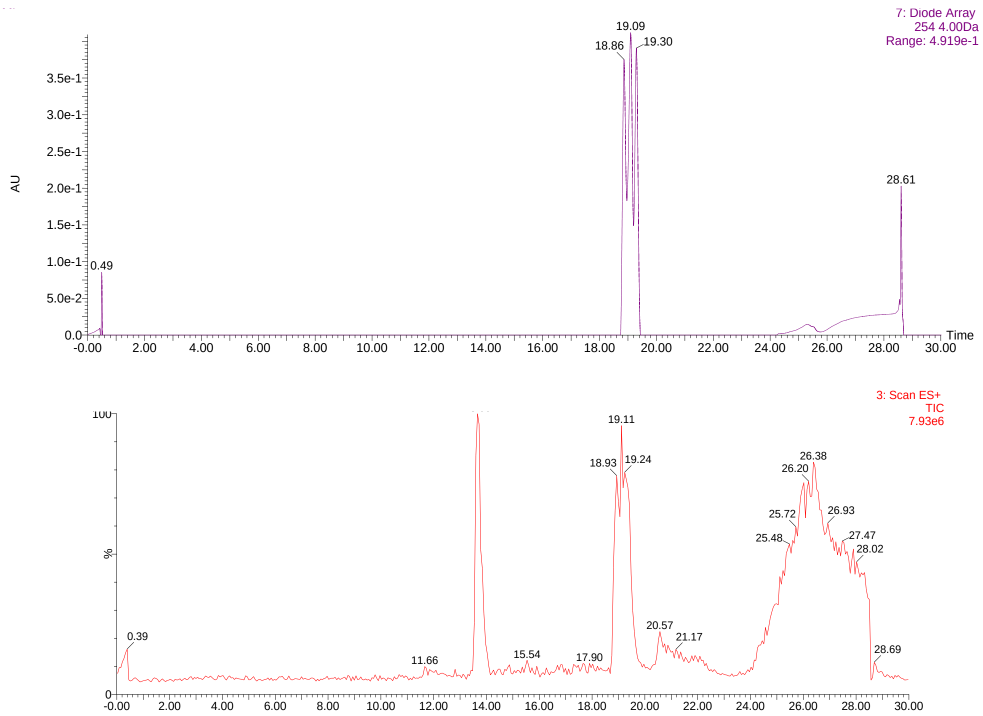

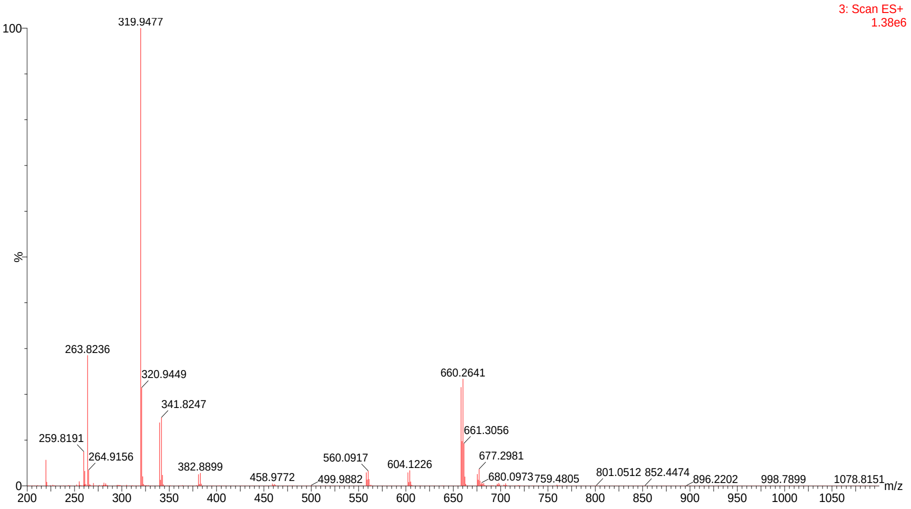
3: Scan ES+
1.38e6
100
%
0
319.9477
263.8236
320.9449
341.8247
259.8191
264.9156
382.8899
458.9772
499.9882
560.0917
604.1226
660.2641
661.3056
677.2981
680.0973
759.4805
801.0512
852.4474
896.2202
998.7899
1078.8151
m/z
200
250
300
350
400
450
500
550
600
650
700
750
800
850
900
950
1000
1050

^{1}H NMR (400.16 MHz, DMSO-d_6, 343 K)

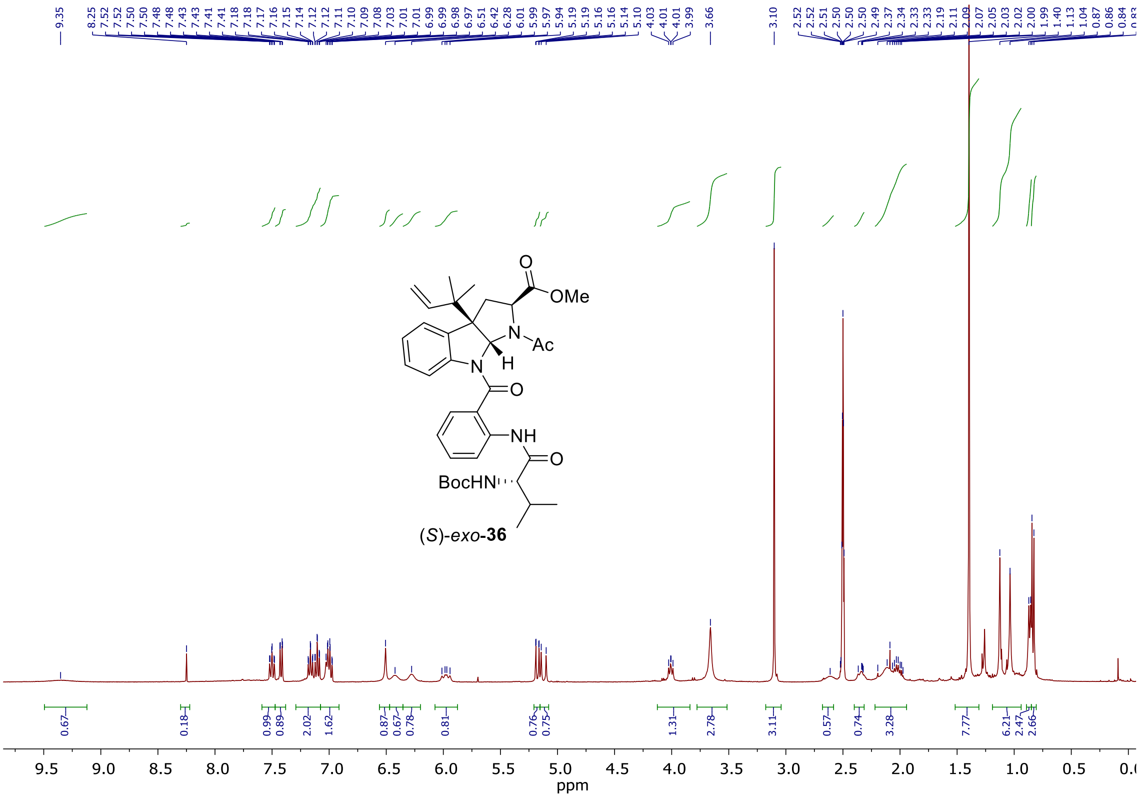

$^{13}C\{^{1}H\}$ NMR (100.62 MHz, DMSO-d_6, 343 K)

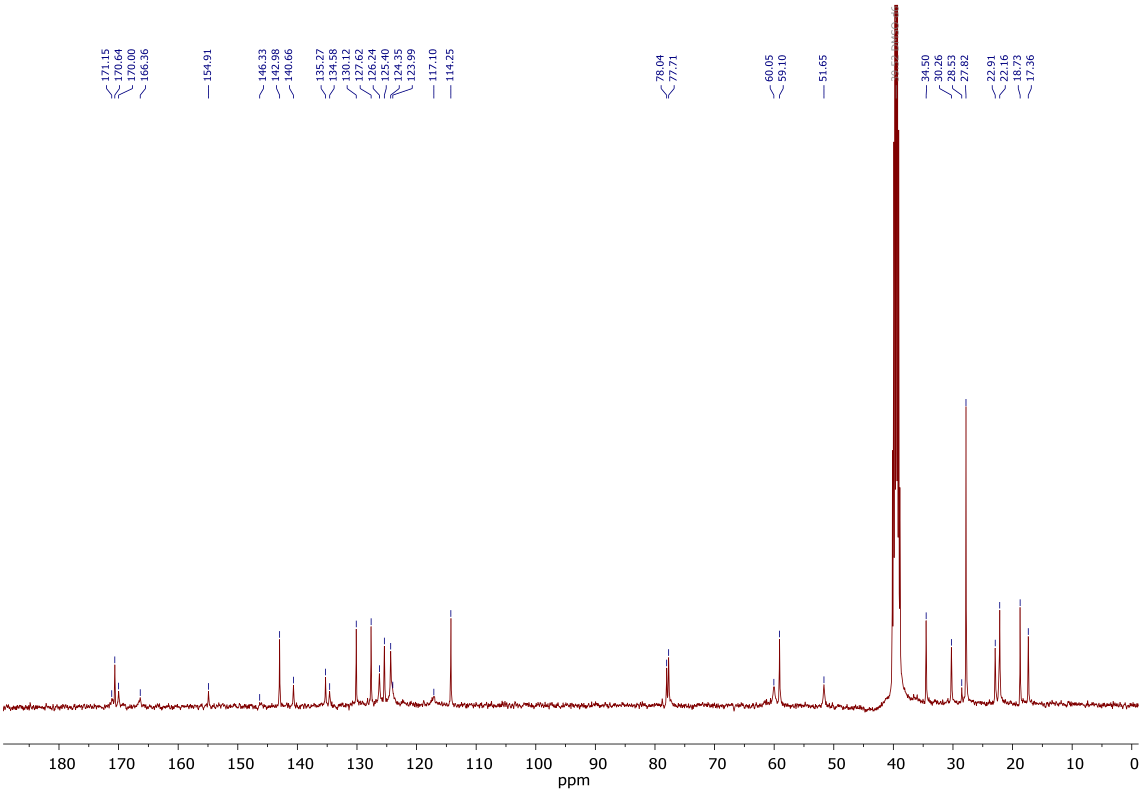

HPLC trace of the fraction of the column of *exo* isomer (*S*)-*exo*-**39** (Scharlau, C18 Kromaphase 100 Kromaphase 100, 5 µm, 250 x 4.6 mm, gradient from CH_3CN/H_2O 50% to CH_3CN 100% in 20 min, 1.0 mL/min).

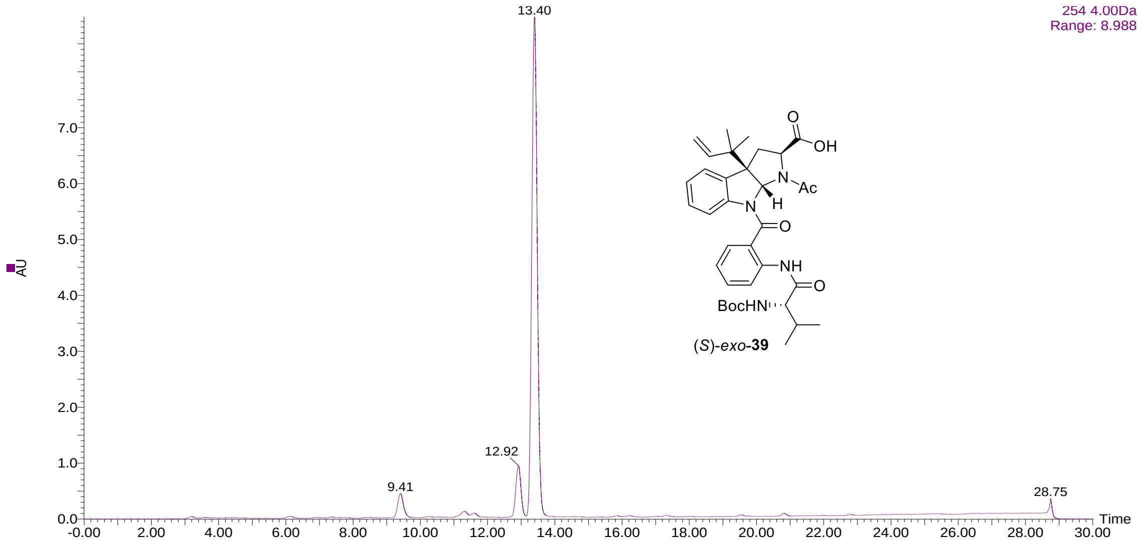

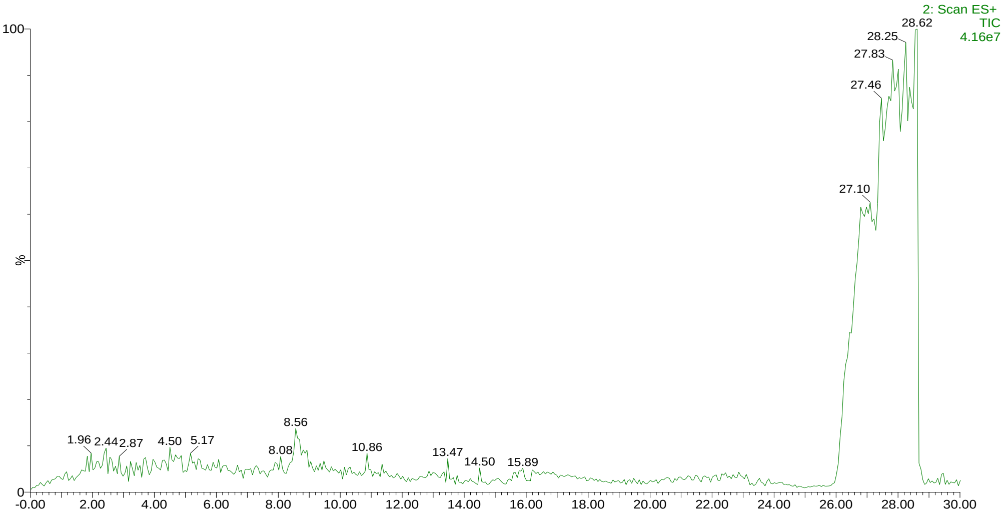

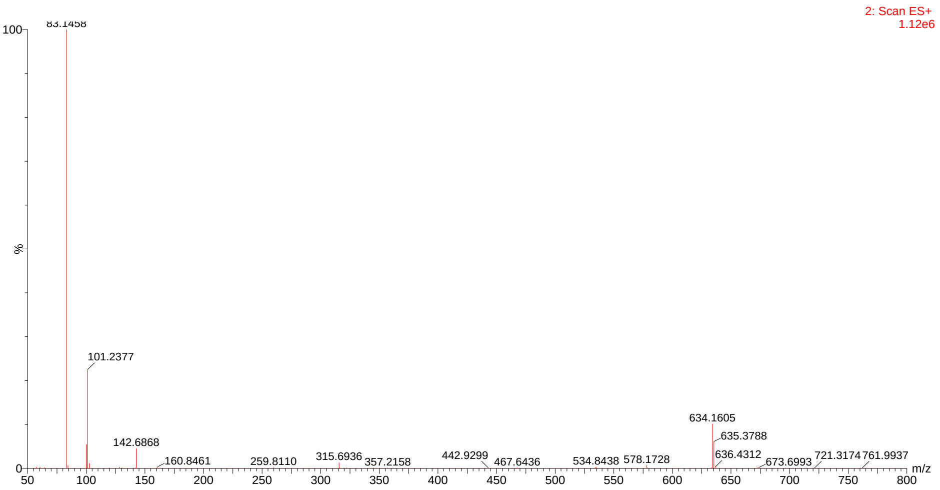

HPLC trace of the fraction of the column of the reaction to obtain **New synthetic novofumigatamide from L-Trp and L-Val** (L-Trp-regio-*exo*-**4**) (Scharlau, C18 Kromaphase 100 Kromaphase 100, 5 µm, 250 x 4.6 mm, gradient from CH_3CN/H_2O 50% to CH_3CN 100% in 20 min, 1.0 mL/min).

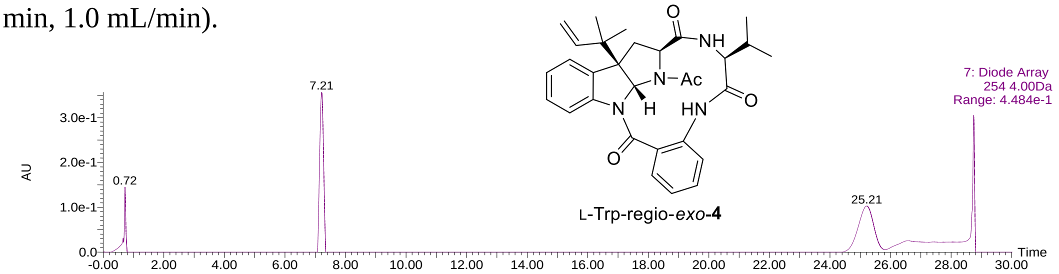

L-Trp-regio-*exo*-**4**

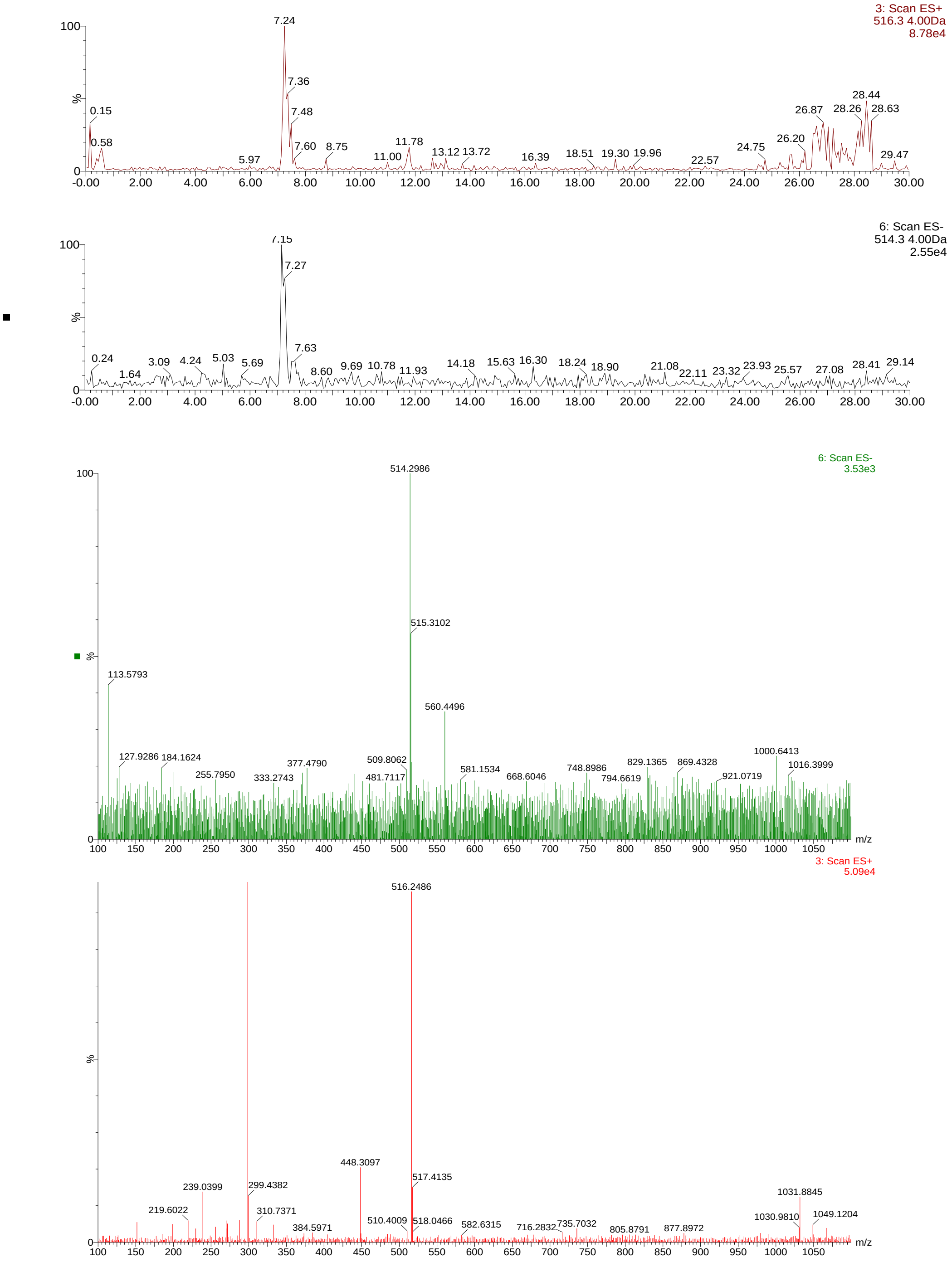

^{1}H NMR (400.16 MHz, CDCl$_3$)

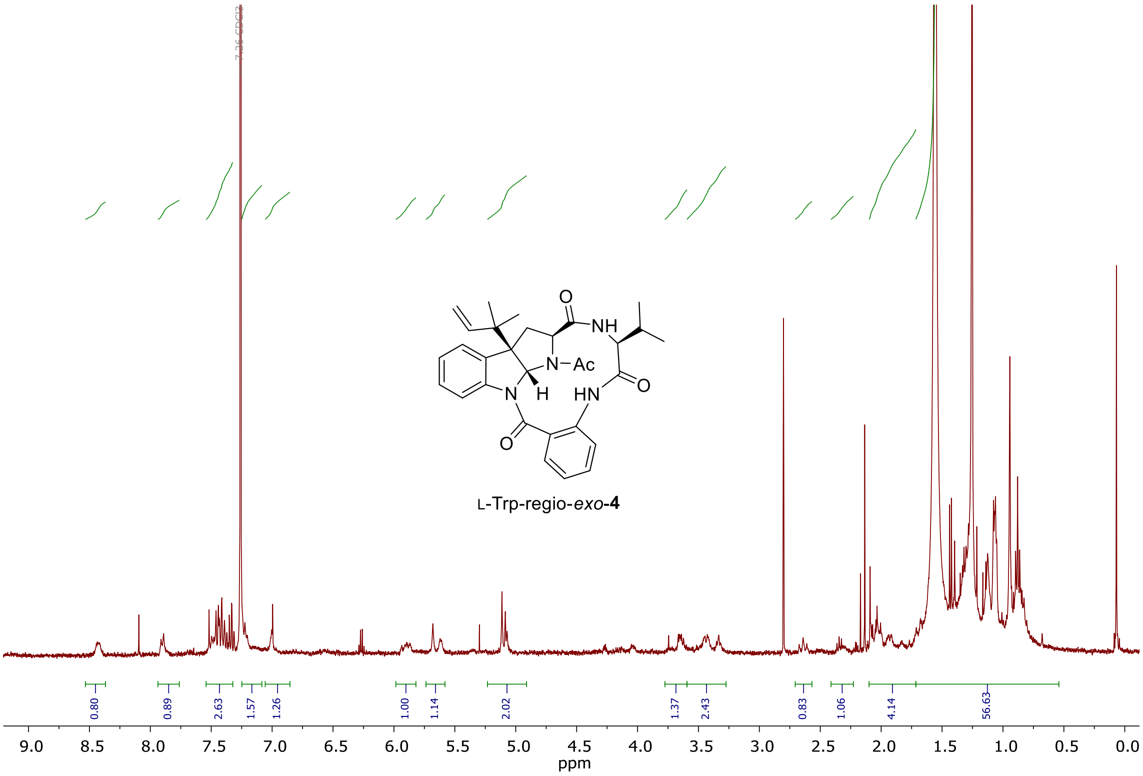

REMARK: this compound could not be fully characterized due to its fast decomposition (during purification by HPLC and acquisition of the NMR experiments).

^{1}H NMR (400.16 MHz, DMSO-d_6, 343 K)

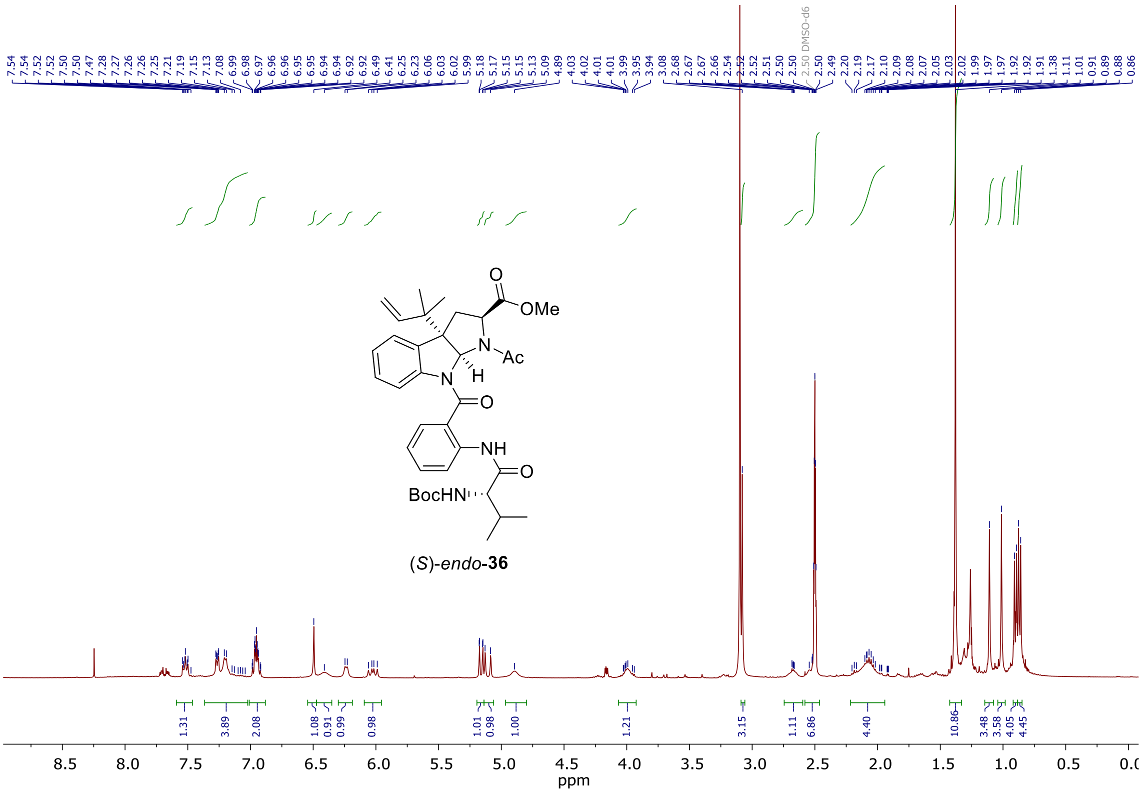

^{13}C{^{1}H} NMR (100.62 MHz, DMSO-d_6, 343 K)

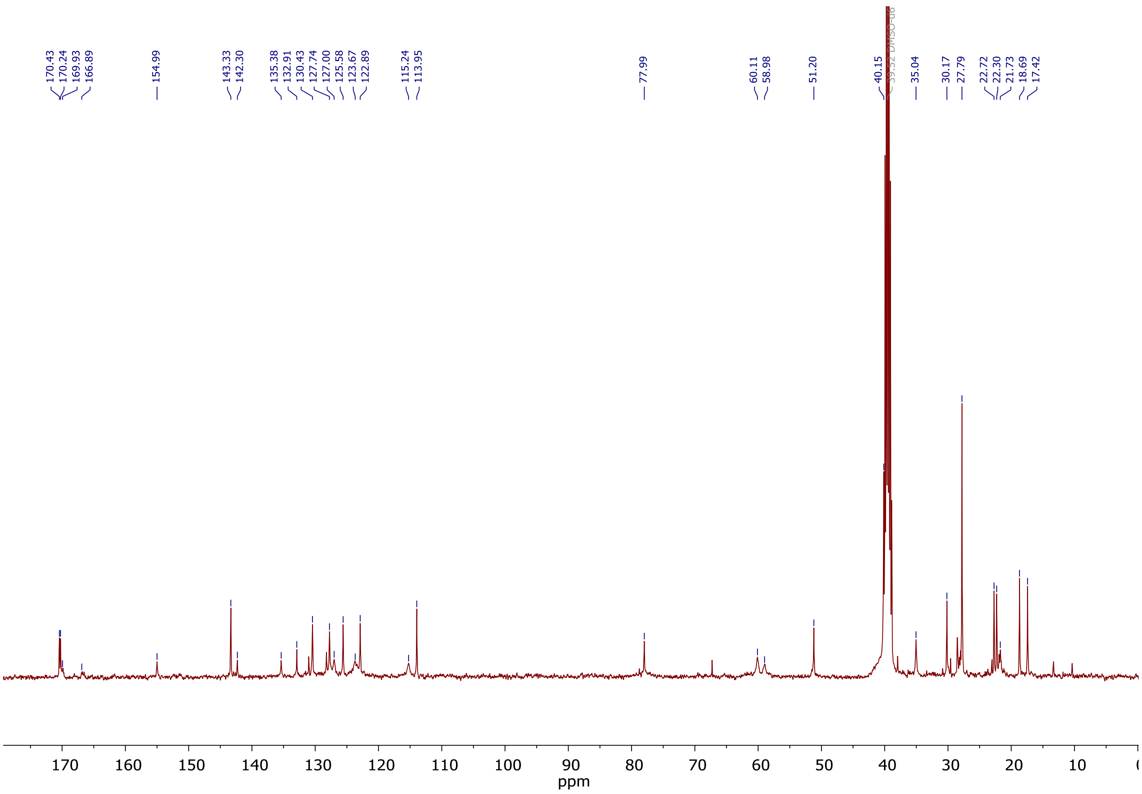

HPLC trace of the fraction of the column of *endo* isomer (*S*)-*endo*-**39** (Scharlau, C18 Kromaphase 100 Kromaphase 100, 5 µm, 250 x 4.6 mm, gradient from CH_3CN/H_2O 50% to CH_3CN 100% in 20 min, 1.0 mL/min).

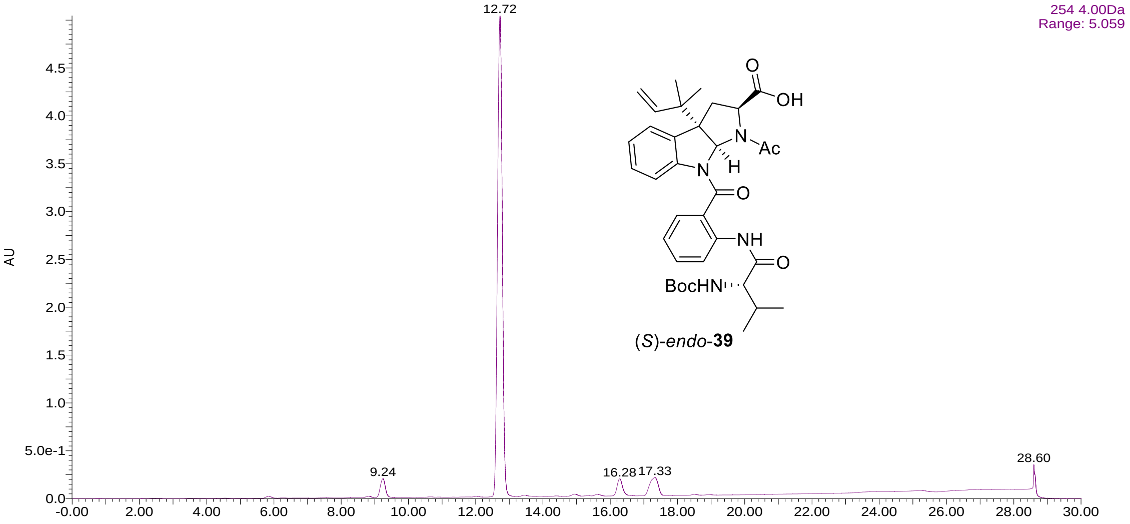

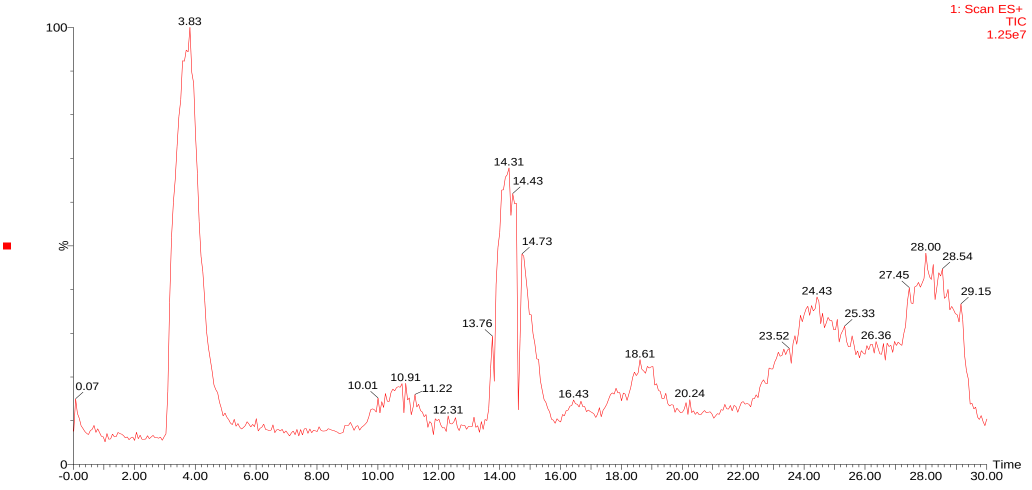

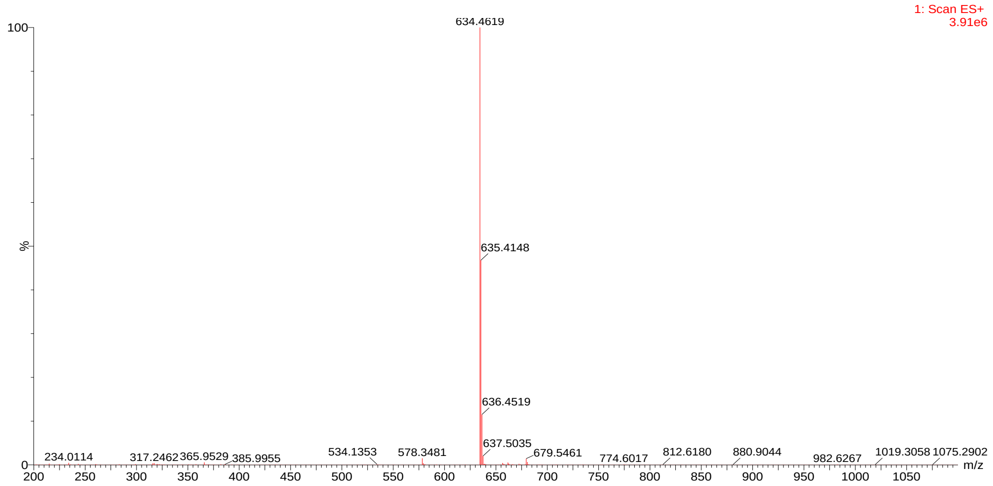

^{1}H NMR (400.16 MHz, $CDCl_3$)

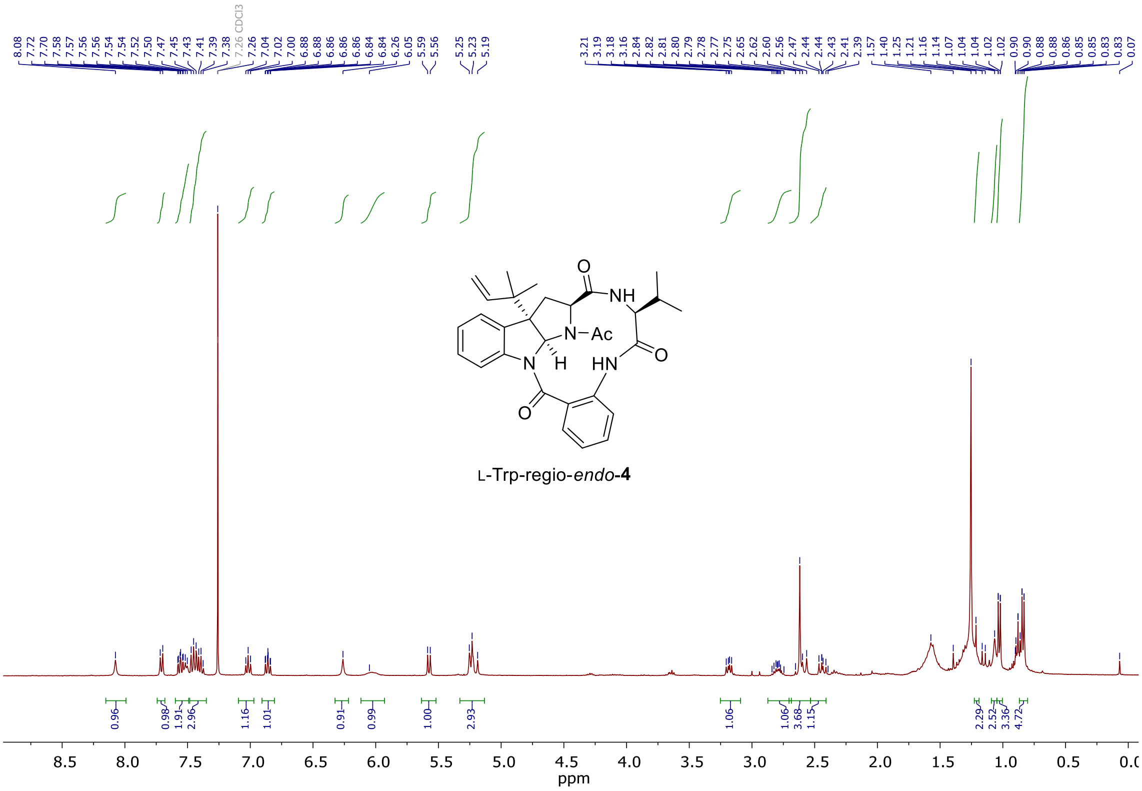

$^{13}C\{^{1}H\}$ NMR (100.62 MHz, $CDCl_3$)

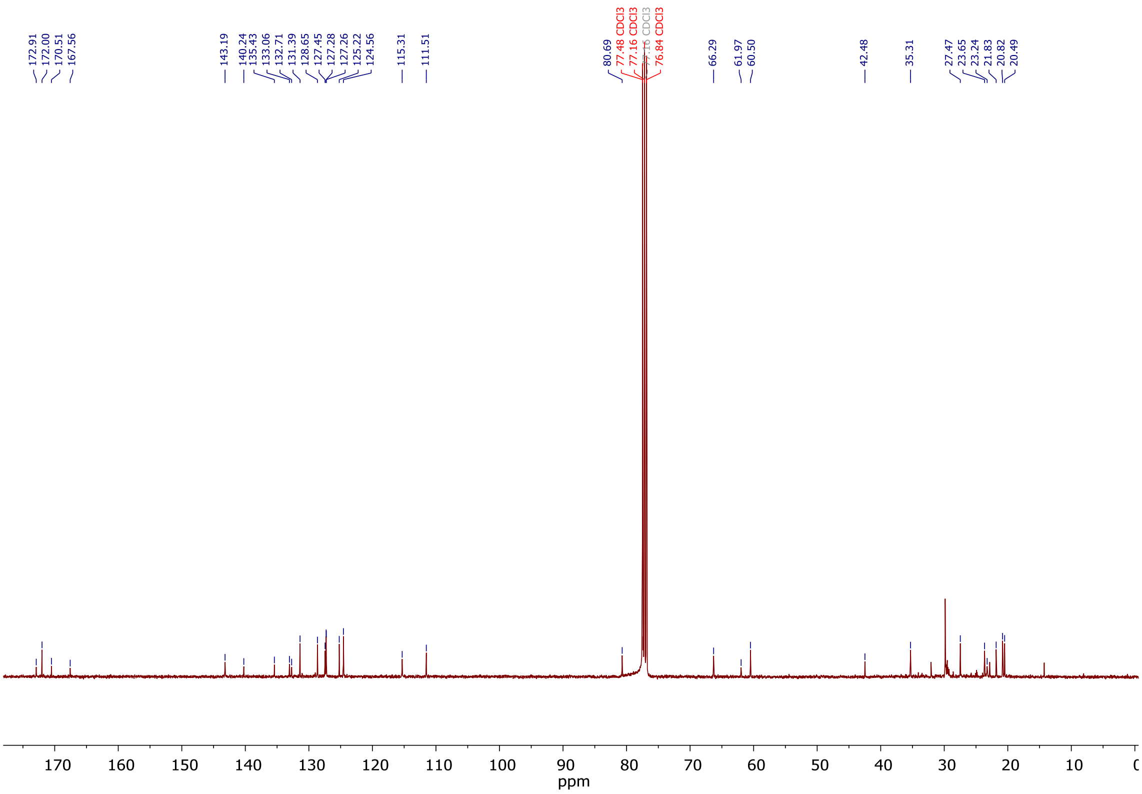

DEPT-135 (100.62 MHz, $CDCl_3$)

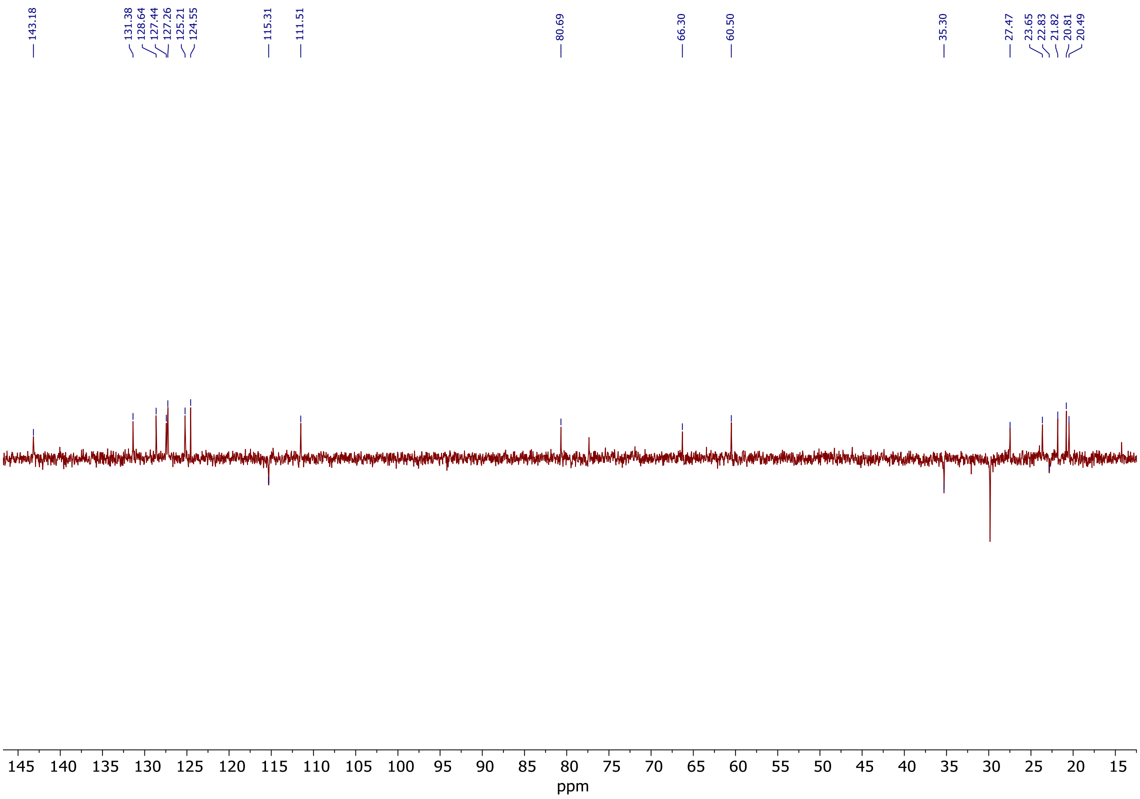

HSQC (100.62 MHz, $CDCl_3$)

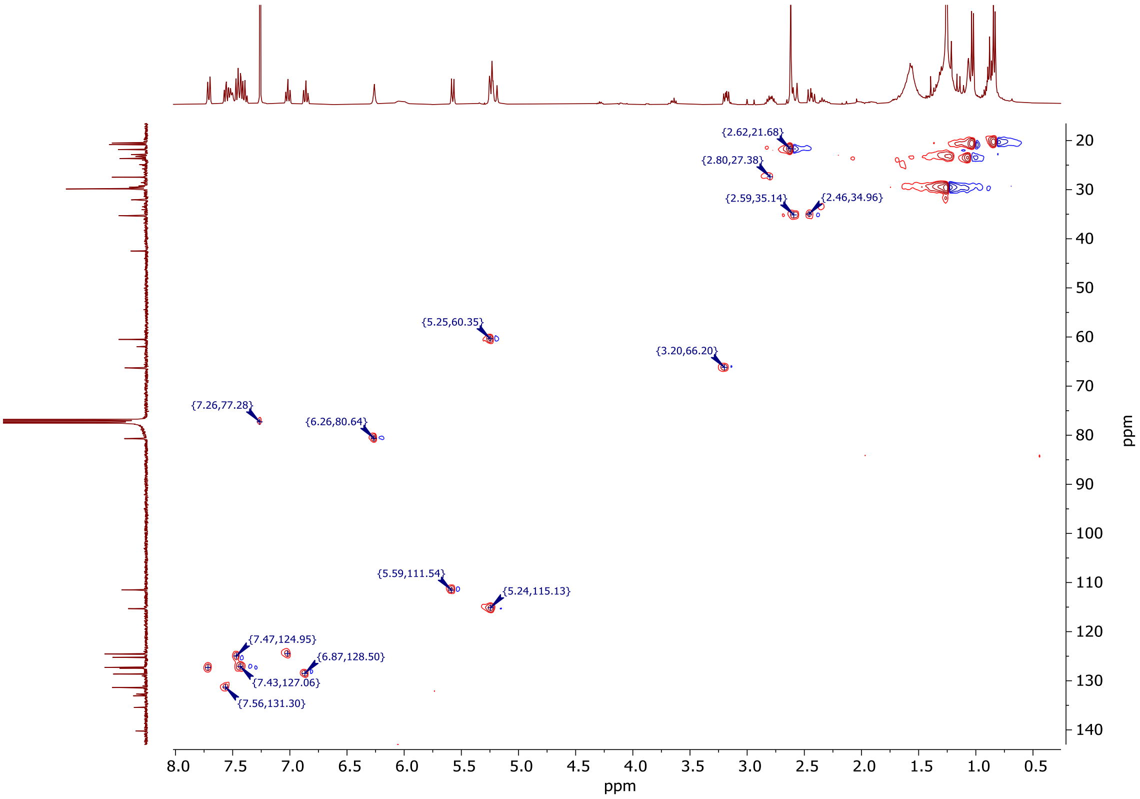

HMBC (100.62 MHz, $CDCl_3$)

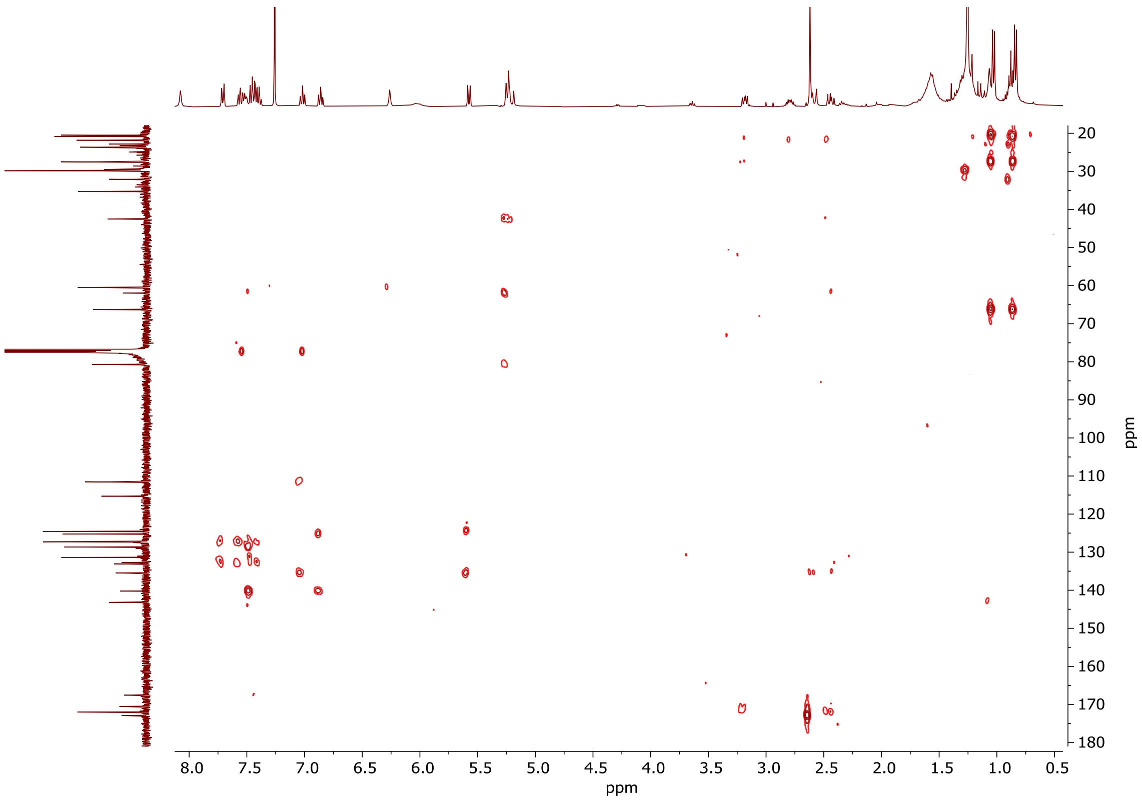

^{1}H NMR (400.16 MHz, $CDCl_3$, 298 K)

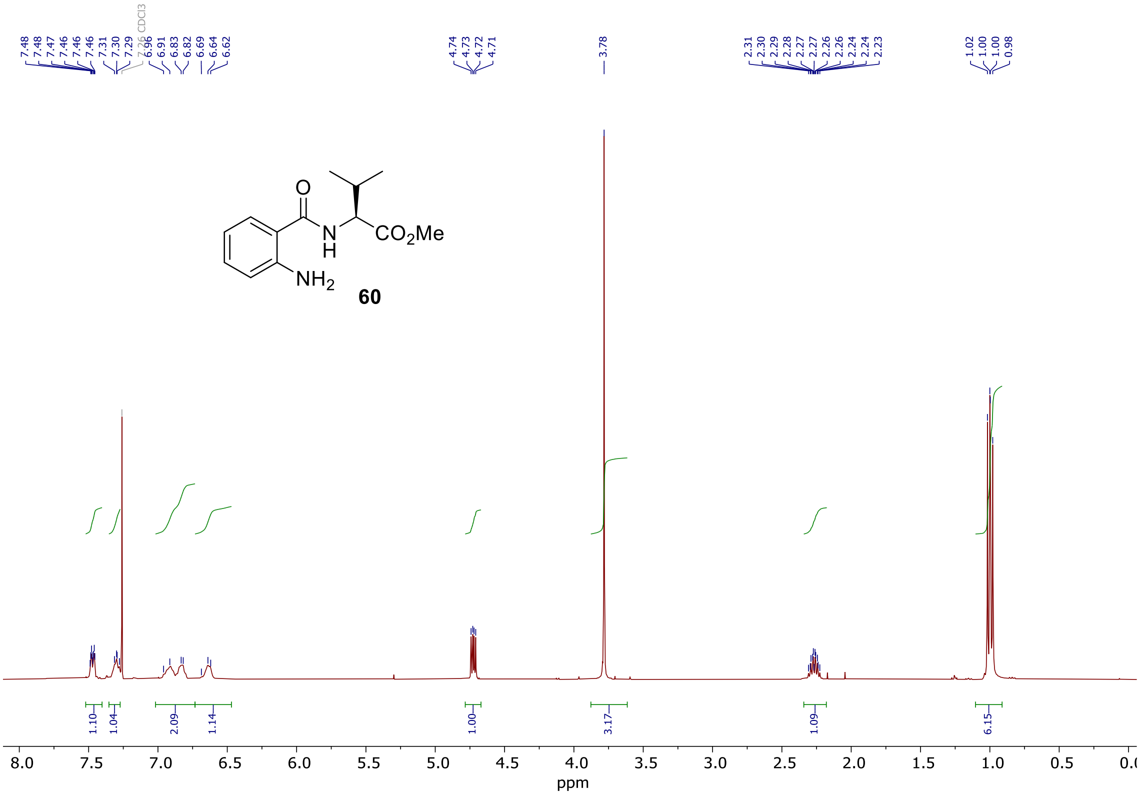

^{1}H NMR (400.16 MHz, DMSO-d_6, 343 K)

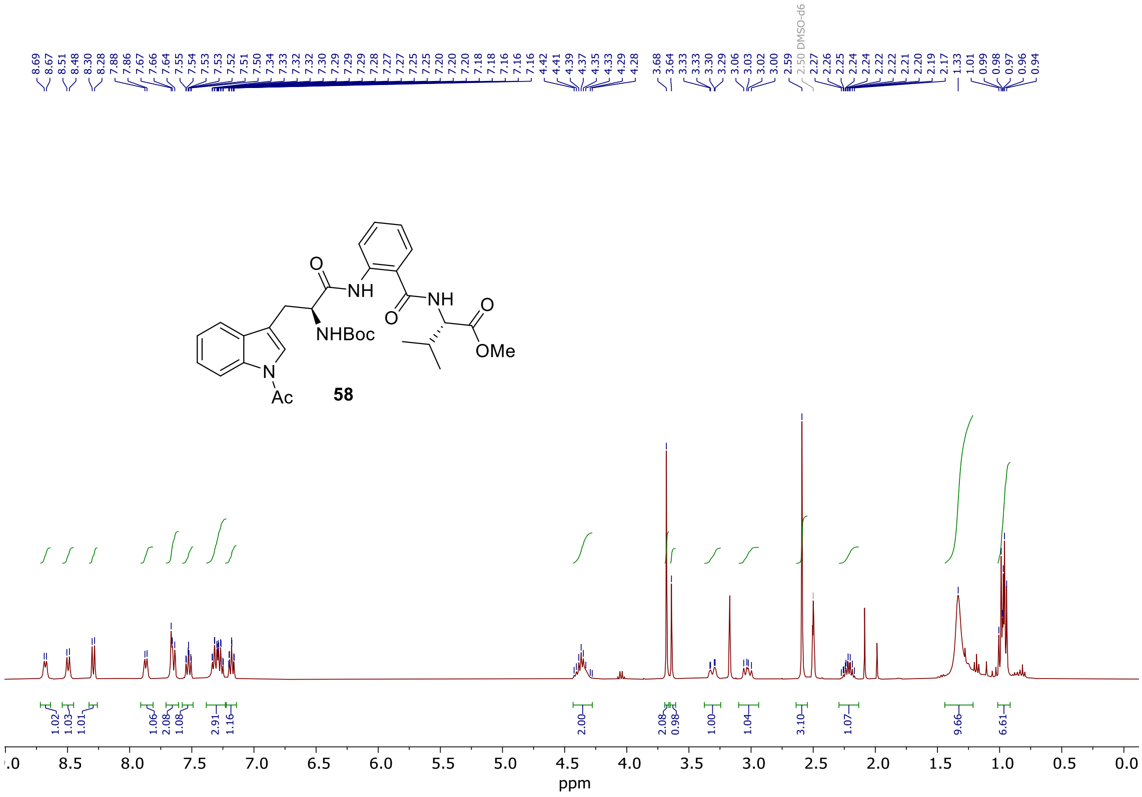

^{13}C{^{1}H} NMR (100.62 MHz, DMSO-d_6, 343 K)

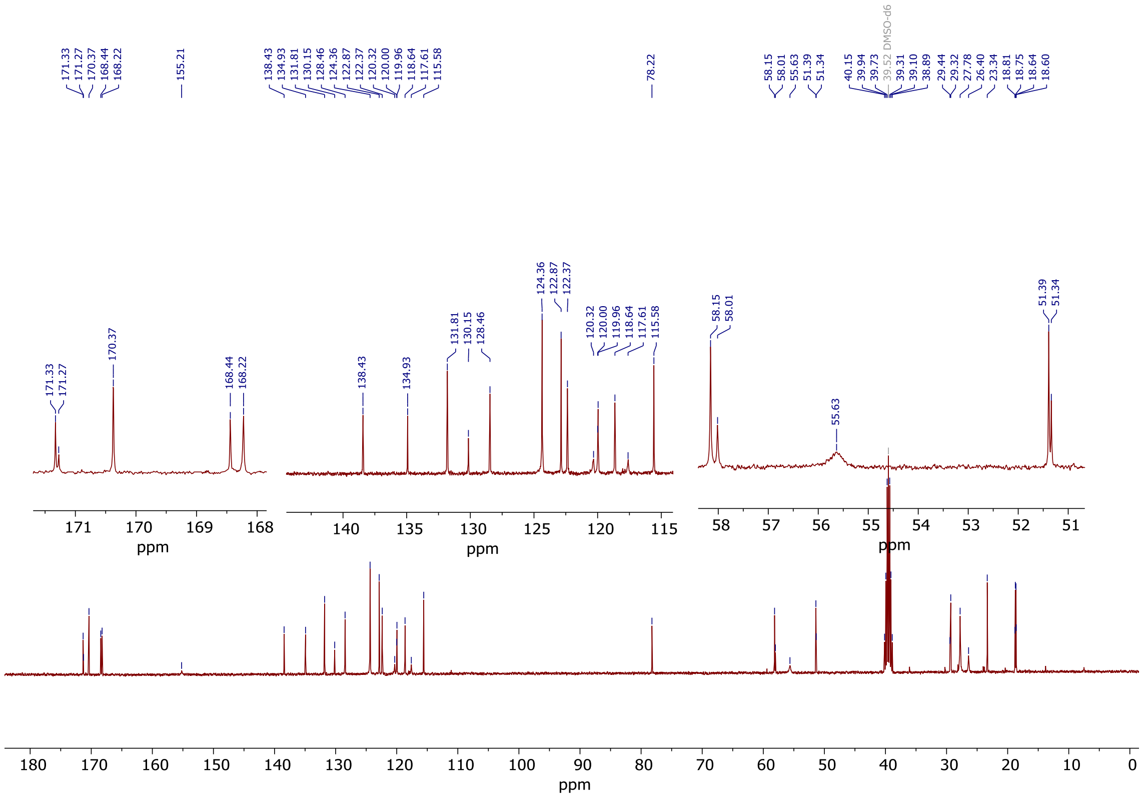

^{1}H NMR (400.16 MHz, DMSO-d_6, 343 K)

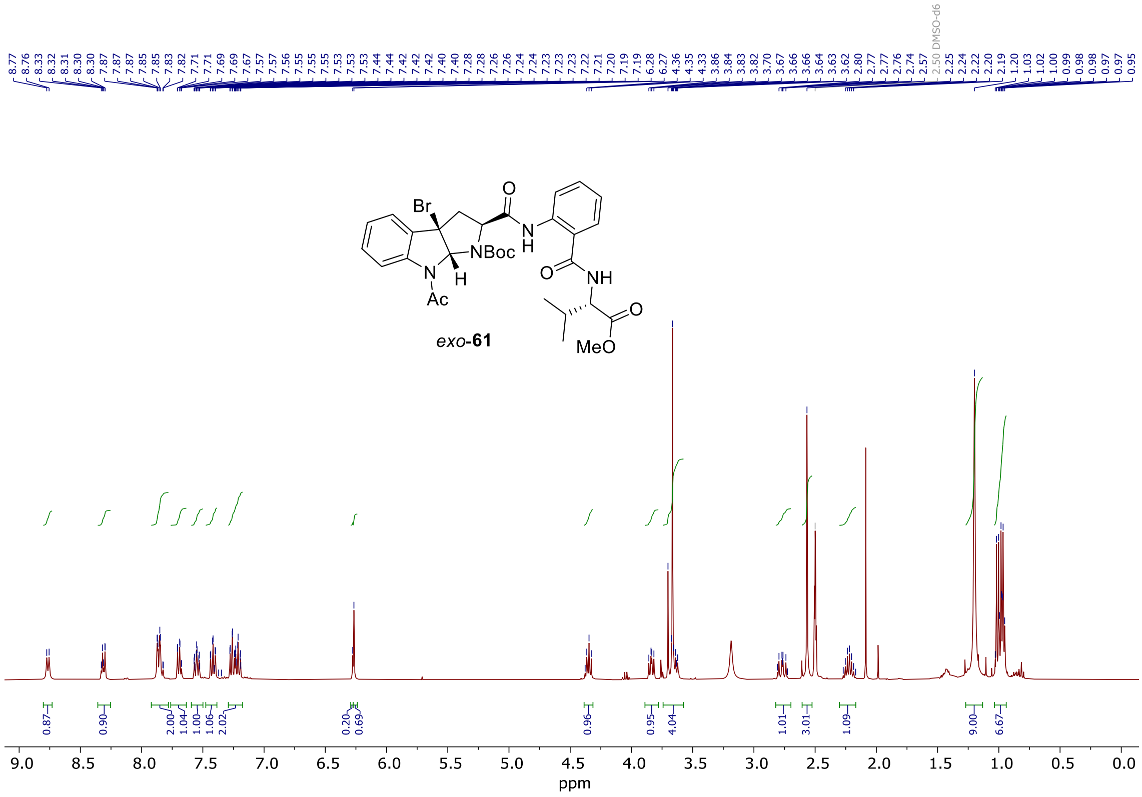

^{13}C{^{1}H} NMR (100.62 MHz, DMSO-d_6, 343 K)

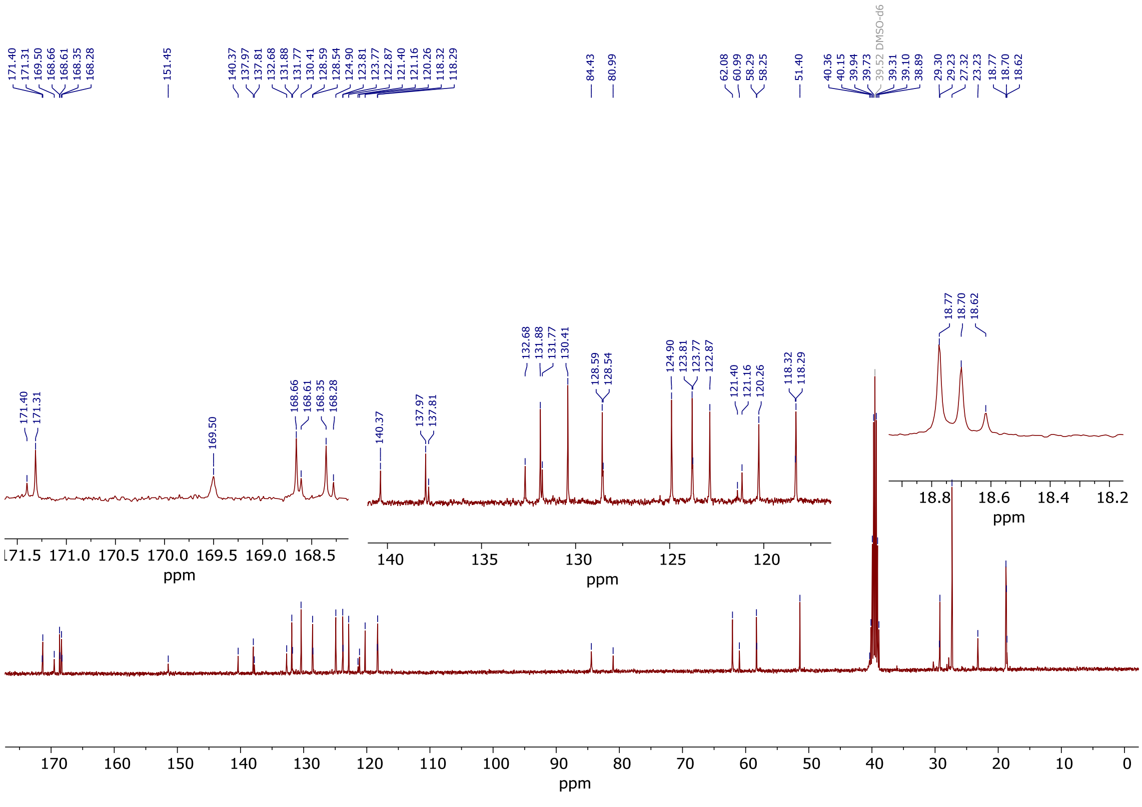

^{1}H NMR (400.16 MHz, DMSO-d_6, 343 K)

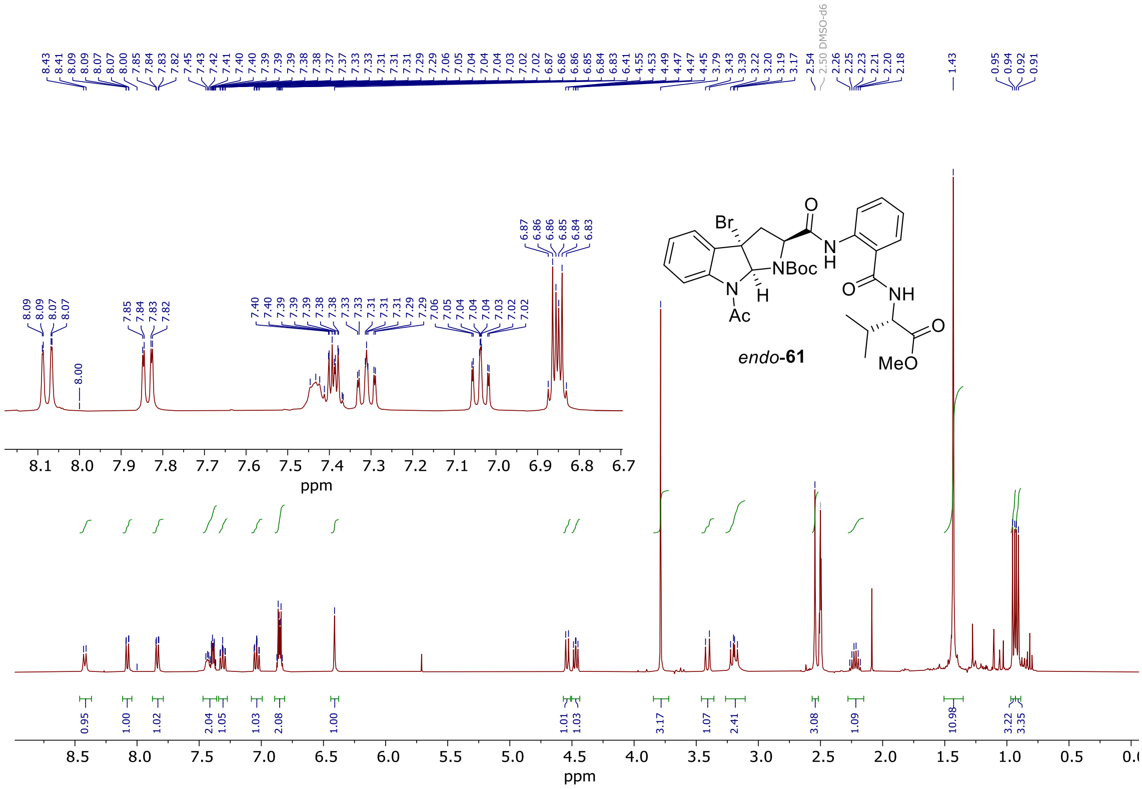

^{13}C{^{1}H} NMR (100.62 MHz, DMSO-d_6, 343 K)

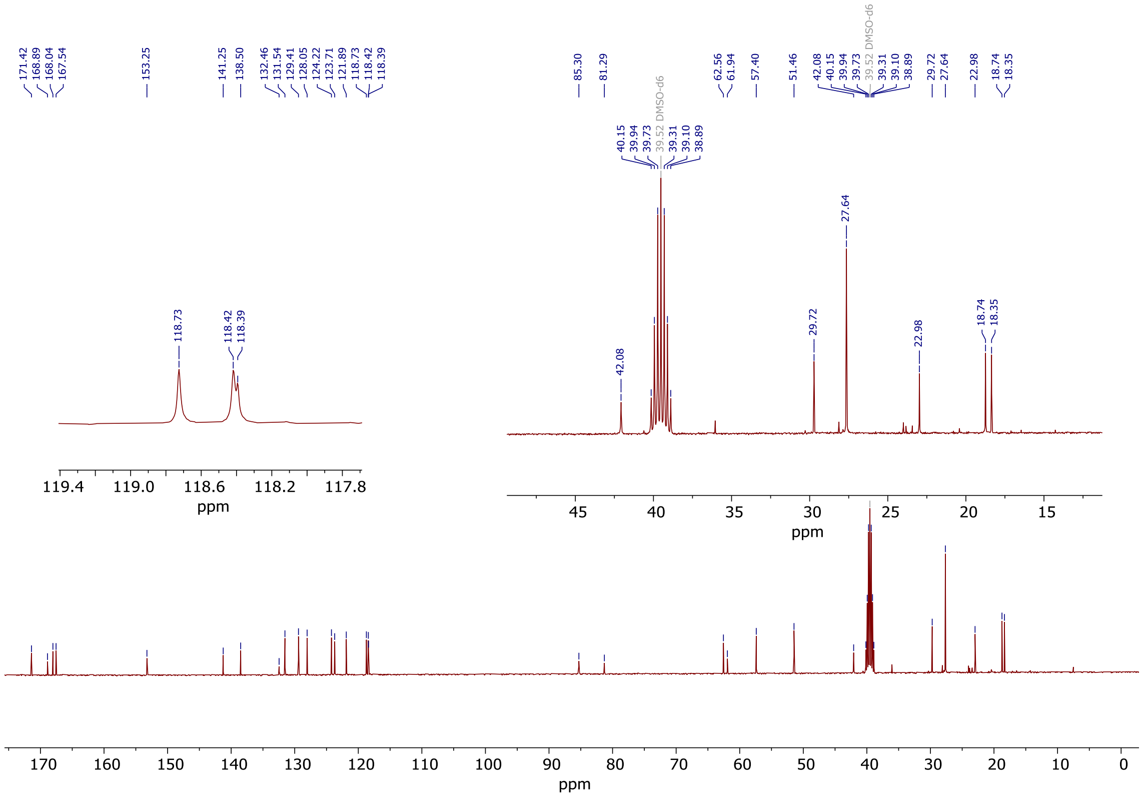

^{1}H NMR (400.16 MHz, DMSO-d_6, 343 K)

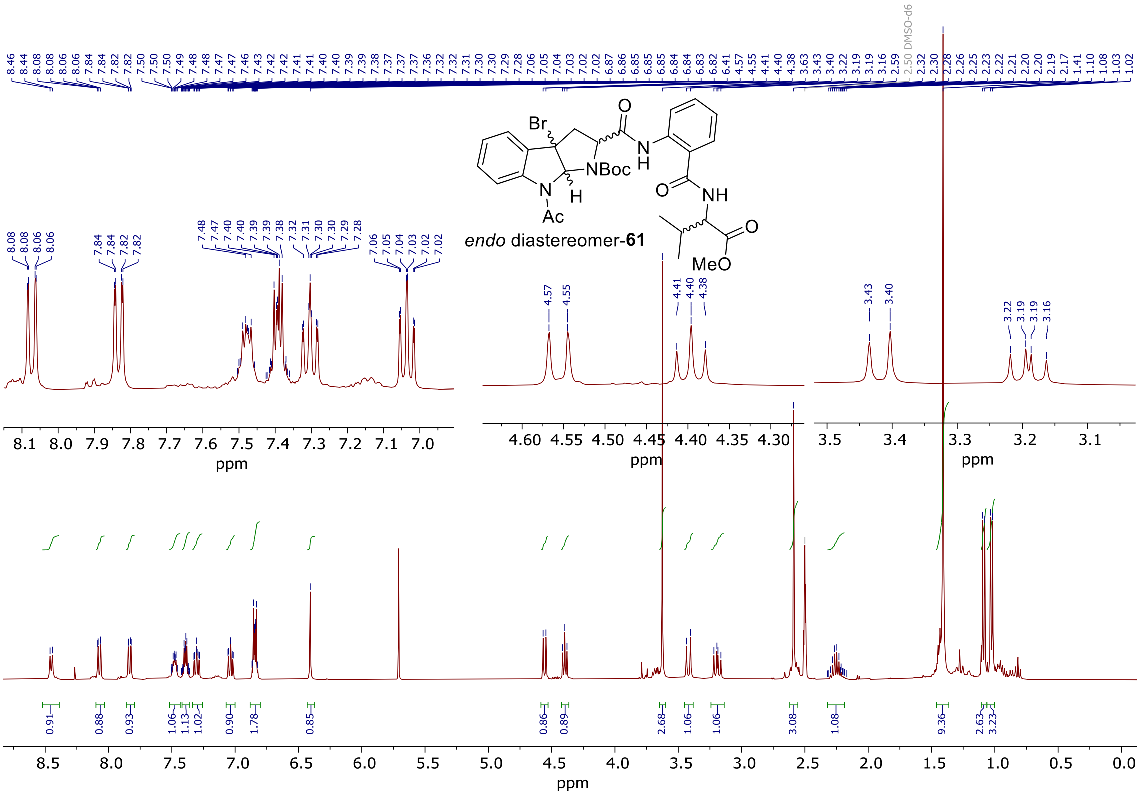

^{13}C{^{1}H} NMR (100.62 MHz, DMSO-d_6, 343 K)

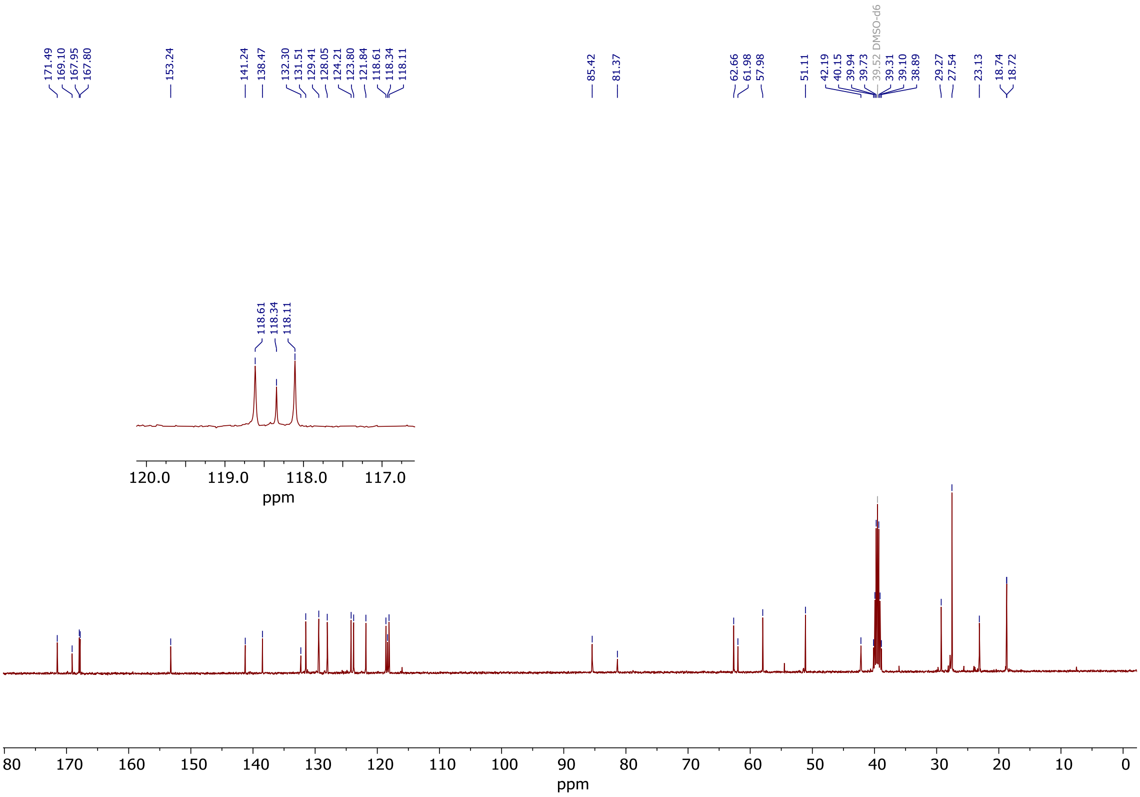

^{1}H NMR (400.16 MHz, DMSO-d_6, 343 K)

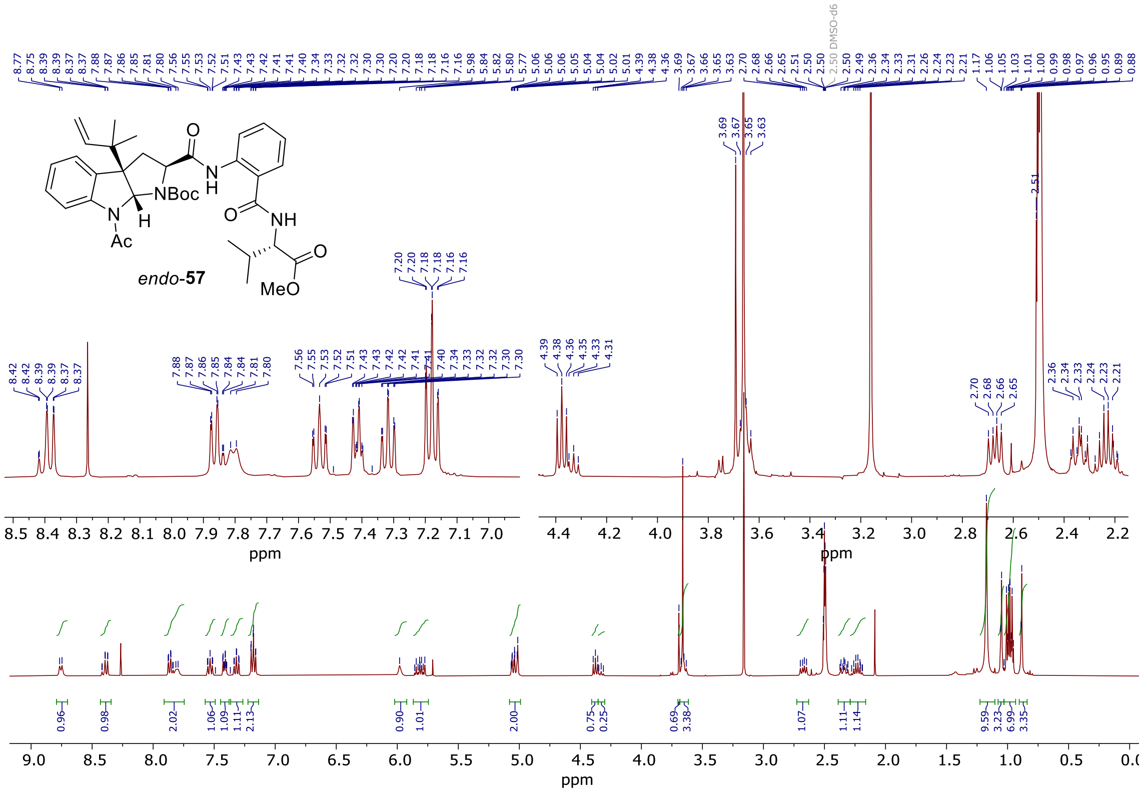

^{13}C{^{1}H} NMR (100.62 MHz, DMSO-d_6, 343 K)

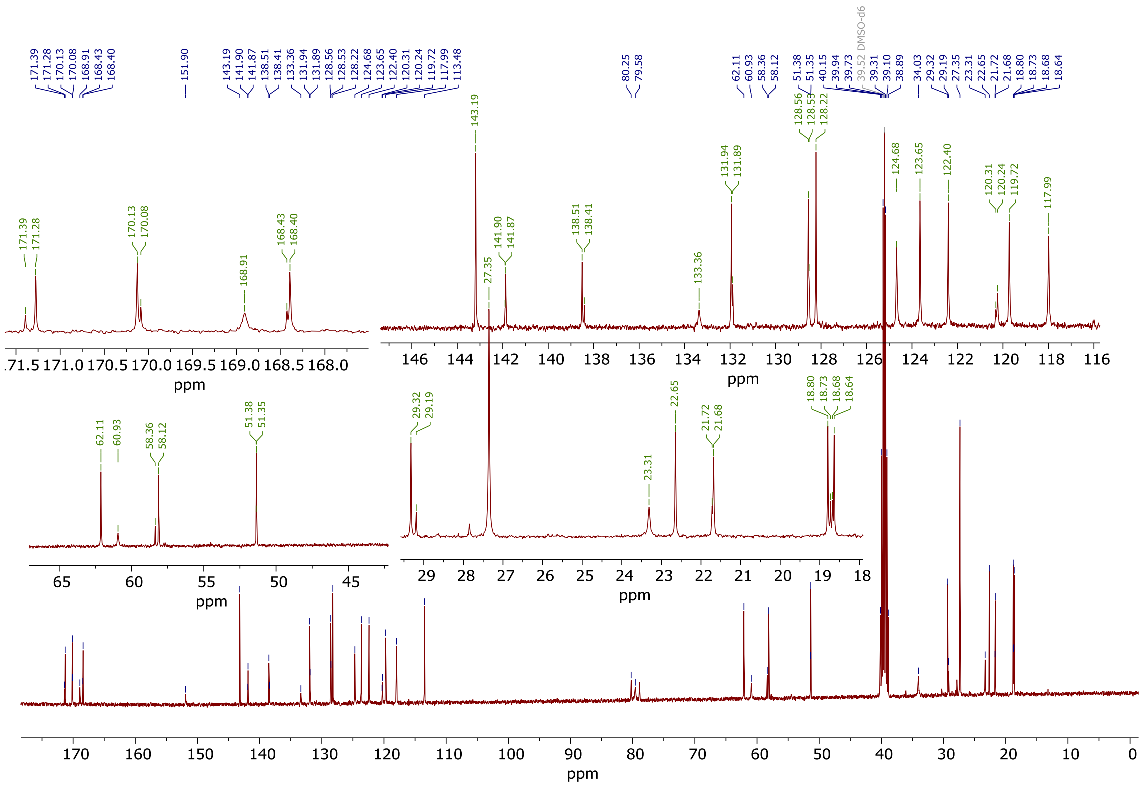

HMBC

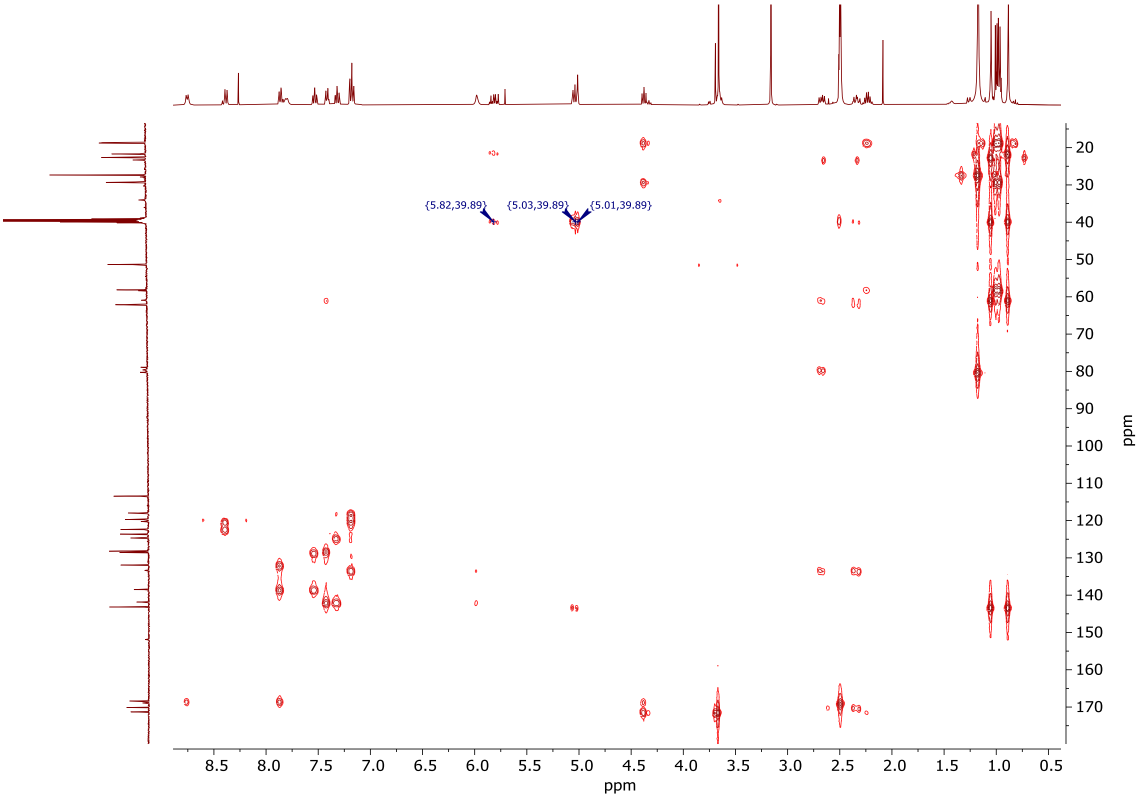

{5.82,39.89}
{5.03,39.89}
{5.01,39.89}
8.5
8.0
7.5
7.0
6.5
6.0
5.5
5.0
4.5
4.0
3.5
3.0
2.5
2.0
1.5
1.0
0.5
ppm
20
30
40
50
60
70
80
90
100
110
120
130
140
150
160
170
ppm

^{1}H NMR (400.16 MHz, DMSO-d_6, 343 K)

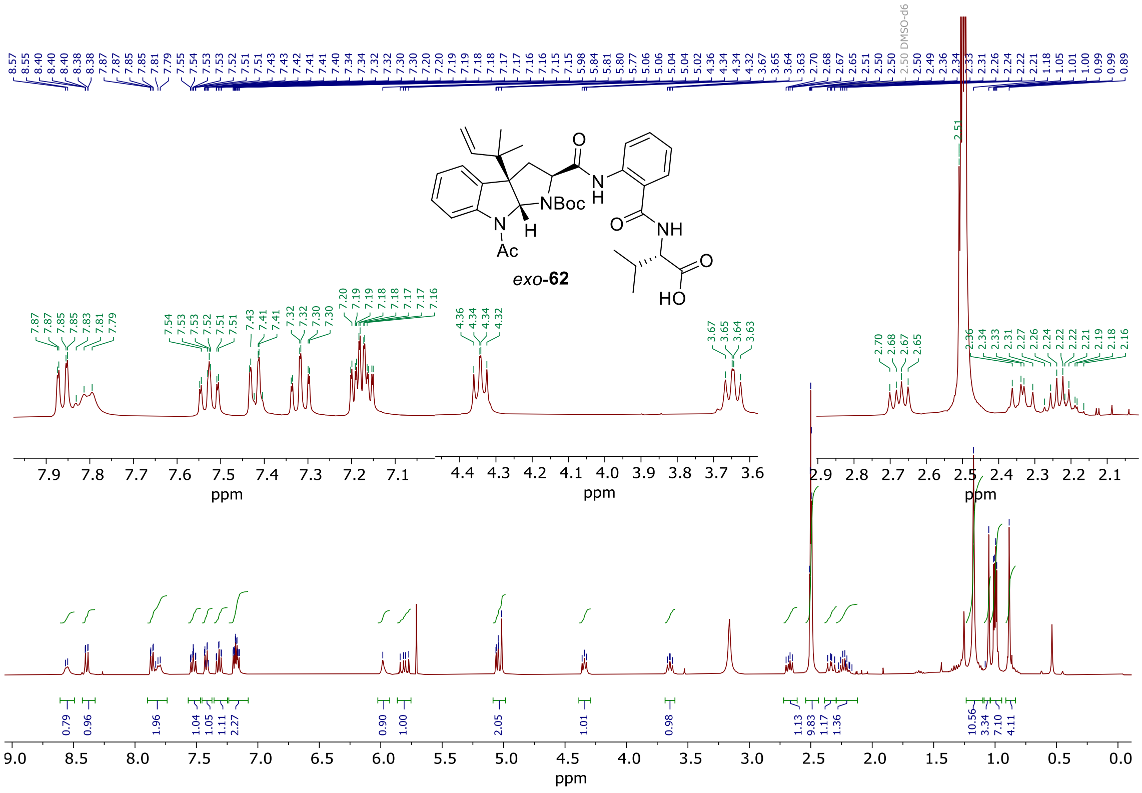

^{13}C{^{1}H} NMR (100.62 MHz, DMSO-d_6, 343 K)

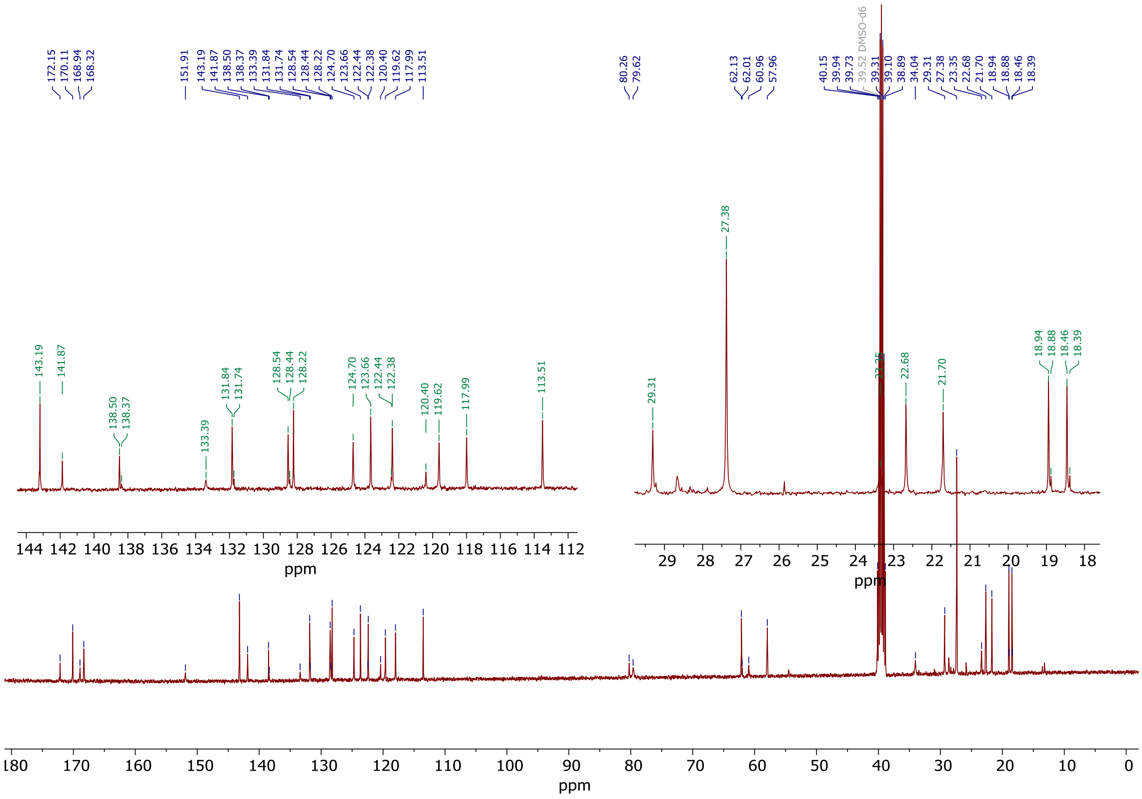

HMBC

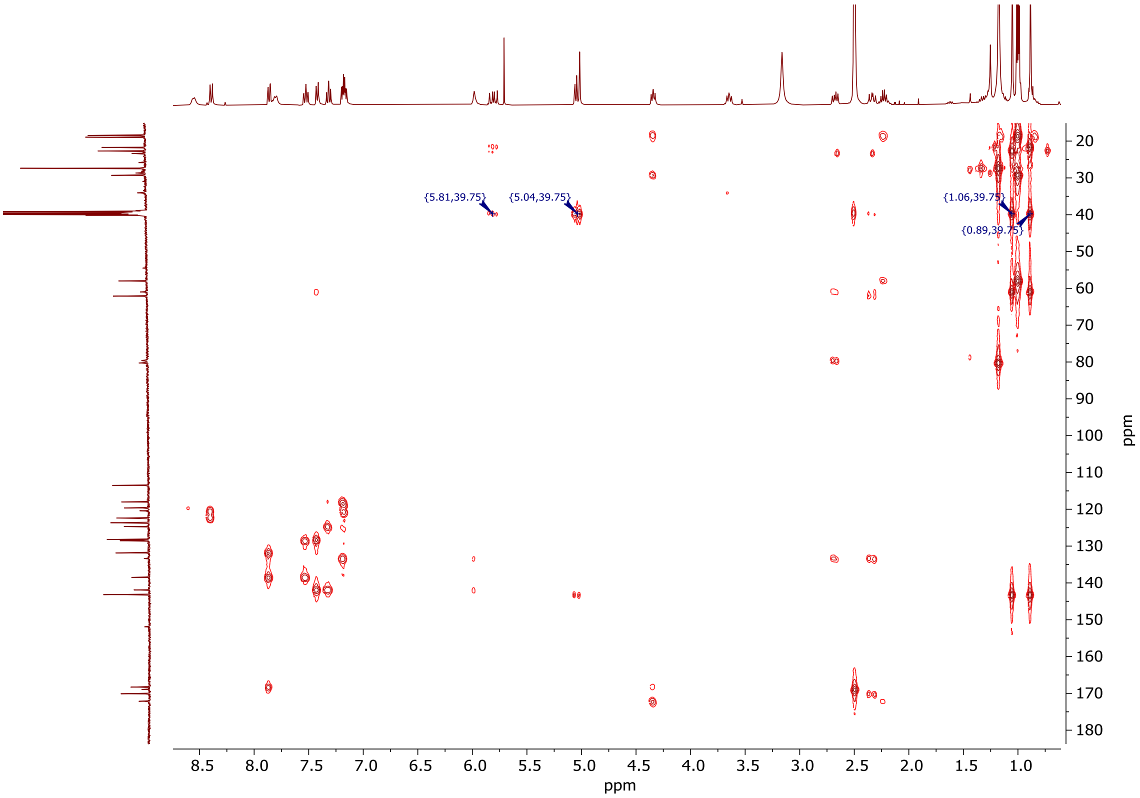

HPLC trace of the reaction progress to obtain *exo*-**63** (Scharlau, C18 Kromaphase 100 Kromaphase 100, 5 µm, 250 x 4.6 mm, gradient from CH_3CN/H_2O 50% to CH_3CN 100% in 20 min, 1.0 mL/min).

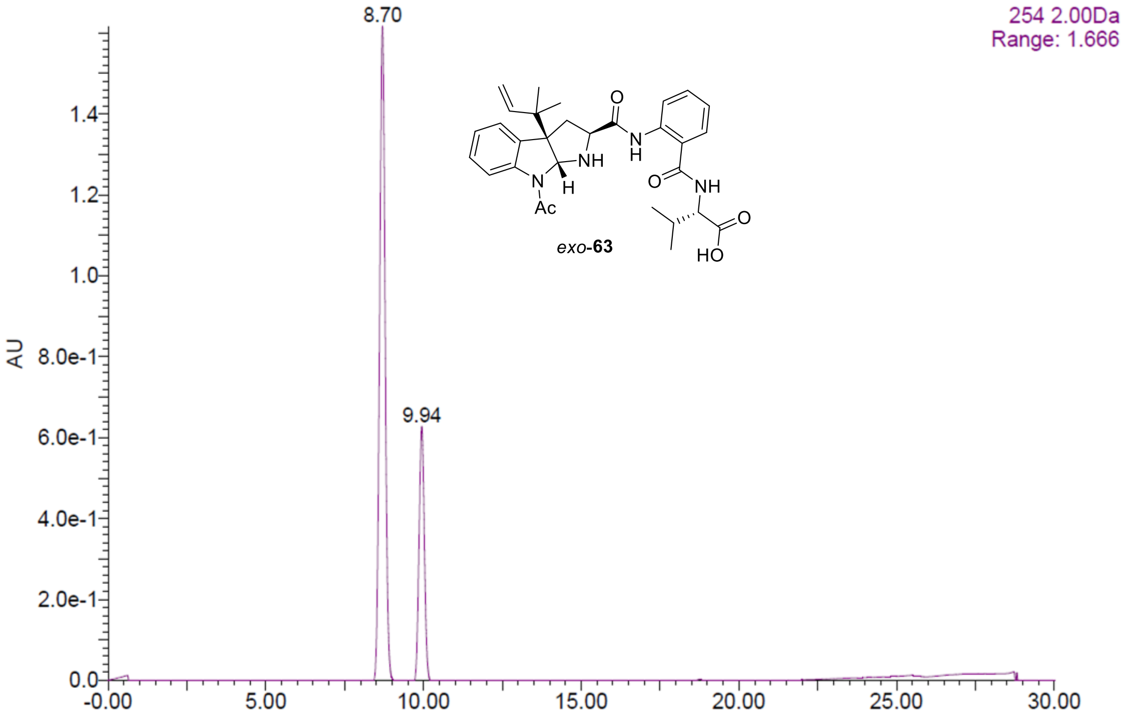

Scharlau_50_to_100_AcN_20min_ (8.734 min)

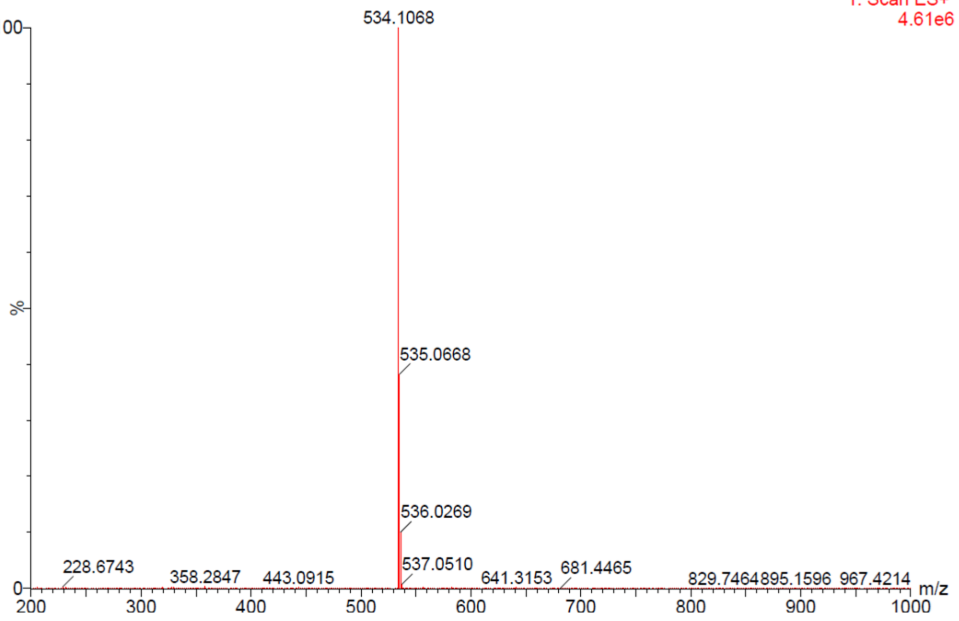

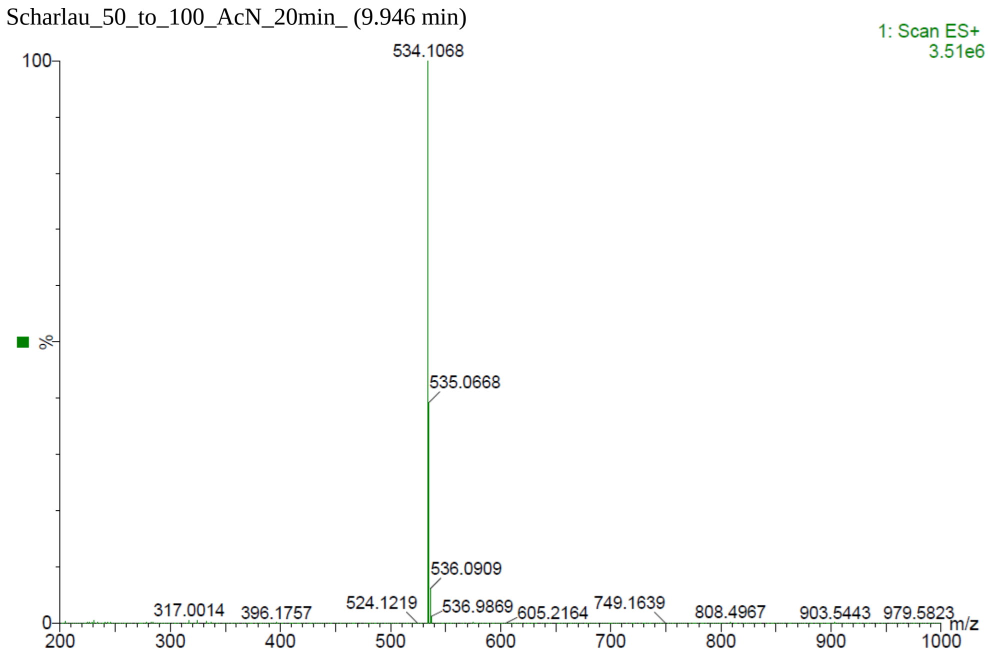

HPLC trace of the reaction progress to obtain **Synthetic novofumigatamide from L-Trp** (L-Trp-regio-*exo*-**56**) (Scharlau, C18 Kromaphase 100 Kromaphase 100, 5 µm, 250 x 4.6 mm, gradient from CH_3CN/H_2O 50% to CH_3CN 100% in 20 min, 1.0 mL/min).

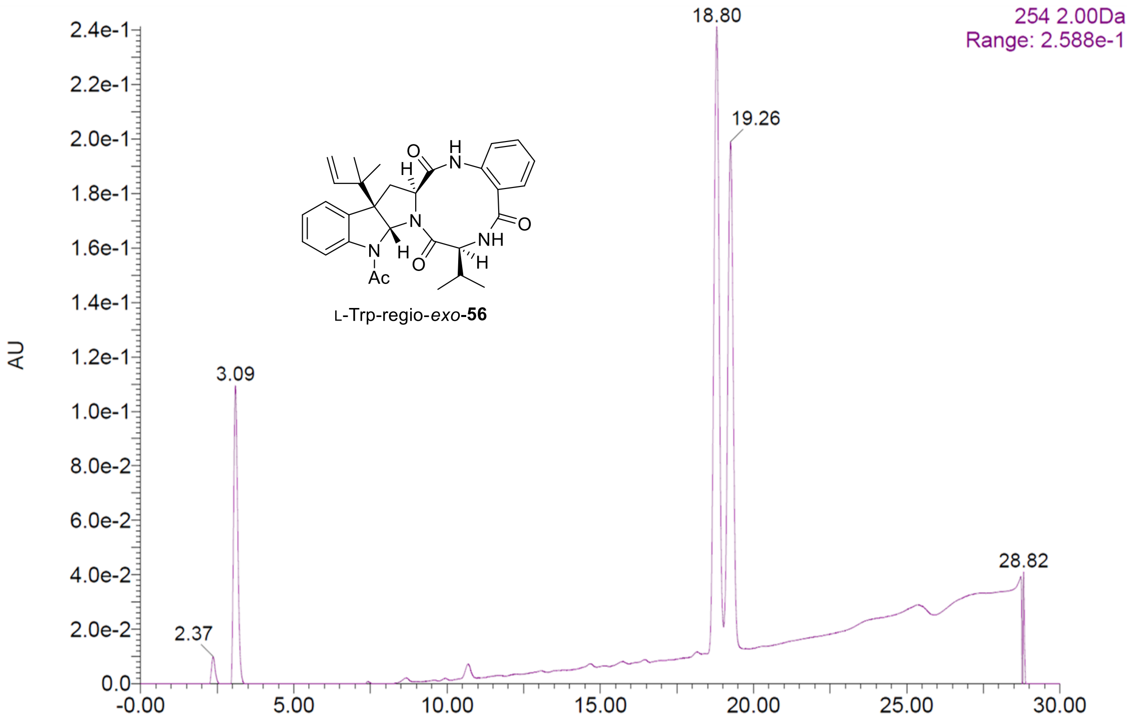

Scharlau_50_to_100_AcN_20min_ (18.730 min)

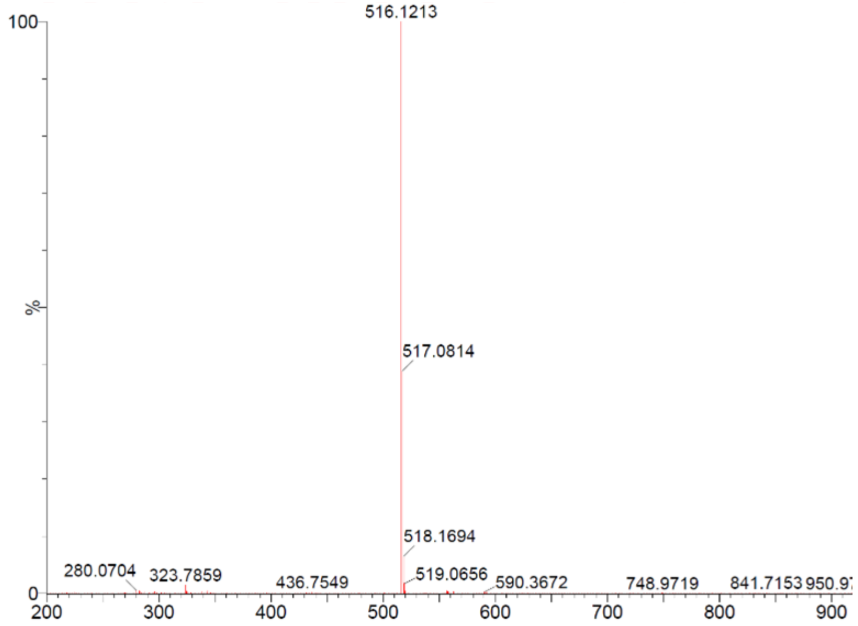

Scharlau_50_to_100_AcN_20min_ (19.275 min)

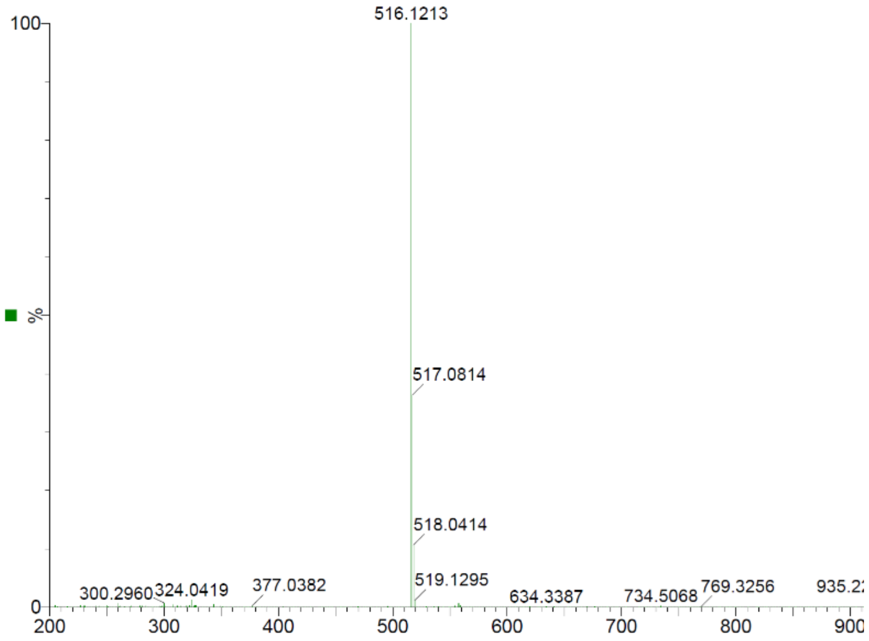

^{1}H NMR (400.16 MHz, DMSO-d_6, 343 K)

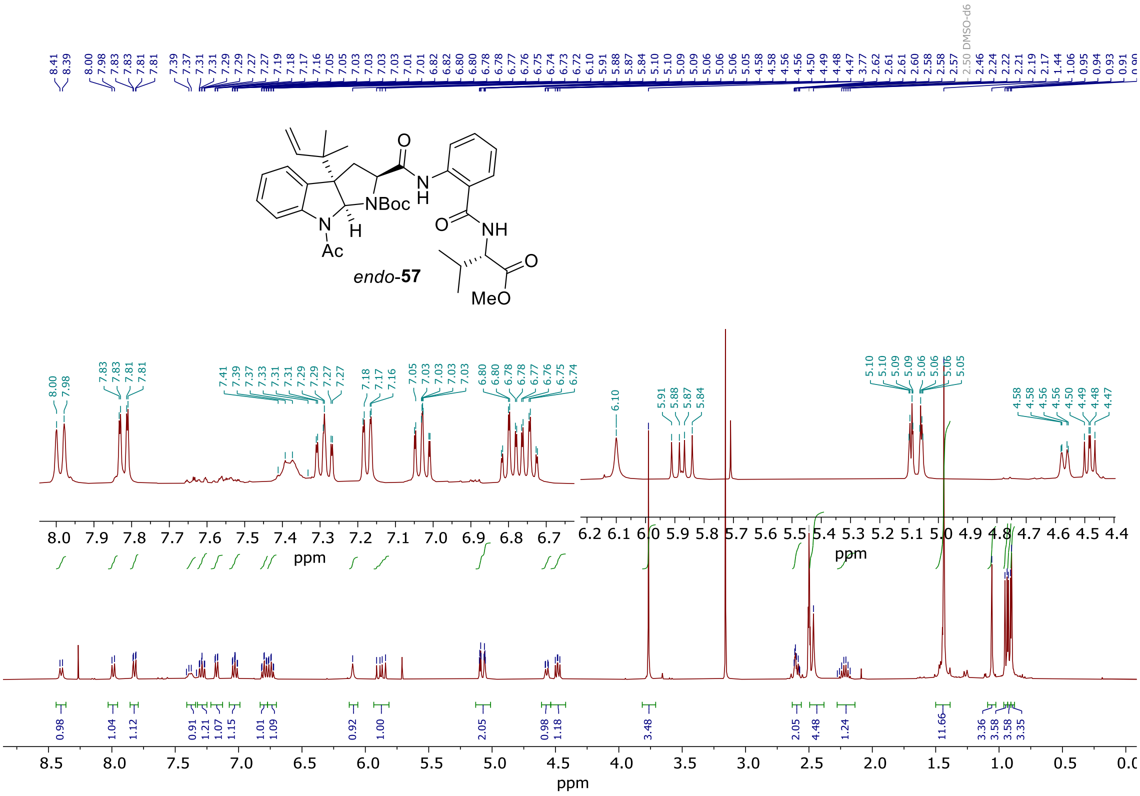

^{13}C{^{1}H} NMR (100.62 MHz, DMSO-d_6, 343 K)

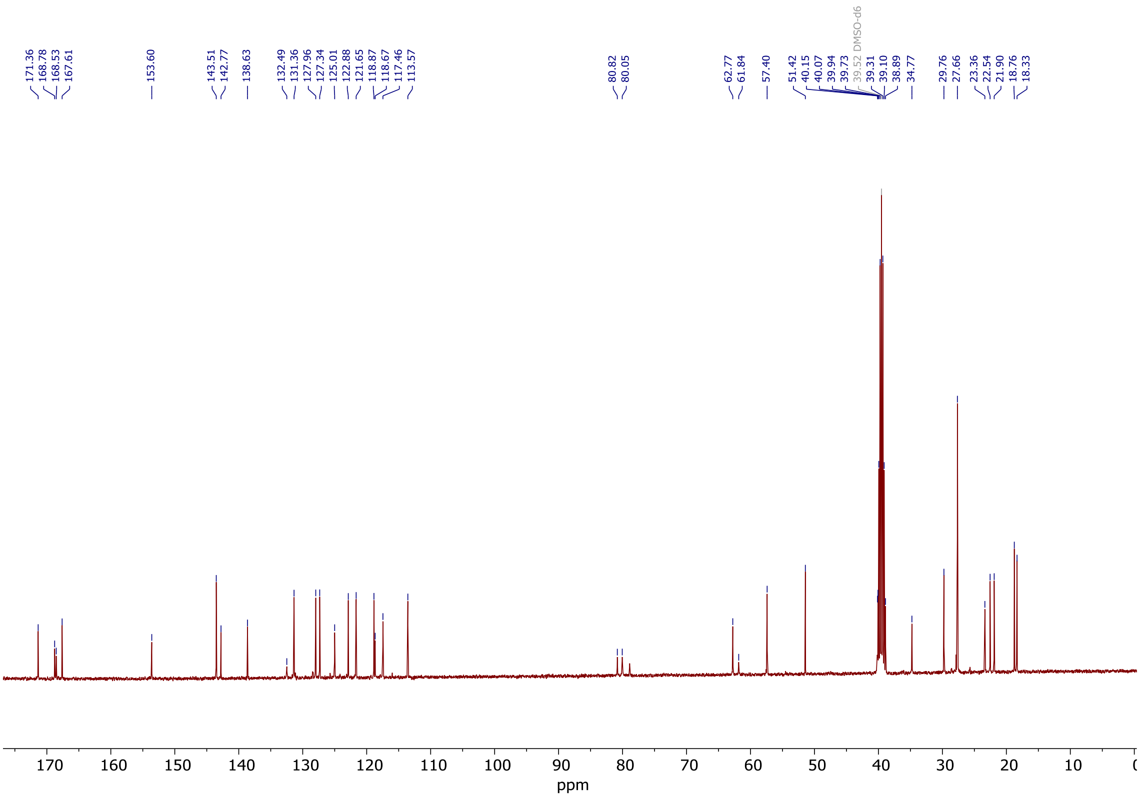

^{1}H NMR (400.16 MHz, DMSO-*d*$_6$, 343 K)

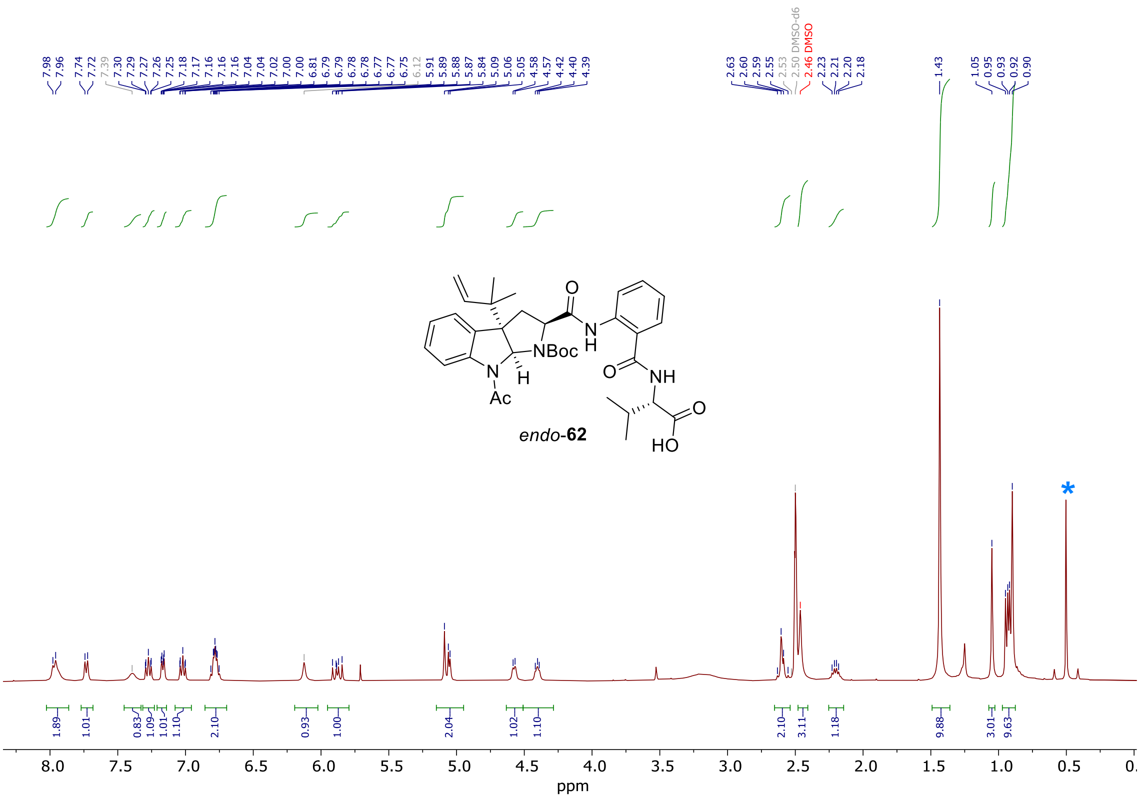

* ^{1}H NMR signals corresponding to remaining Me_3SnOH

^{13}C{^{1}H} NMR (100.62 MHz, DMSO-*d*$_6$, 343 K)

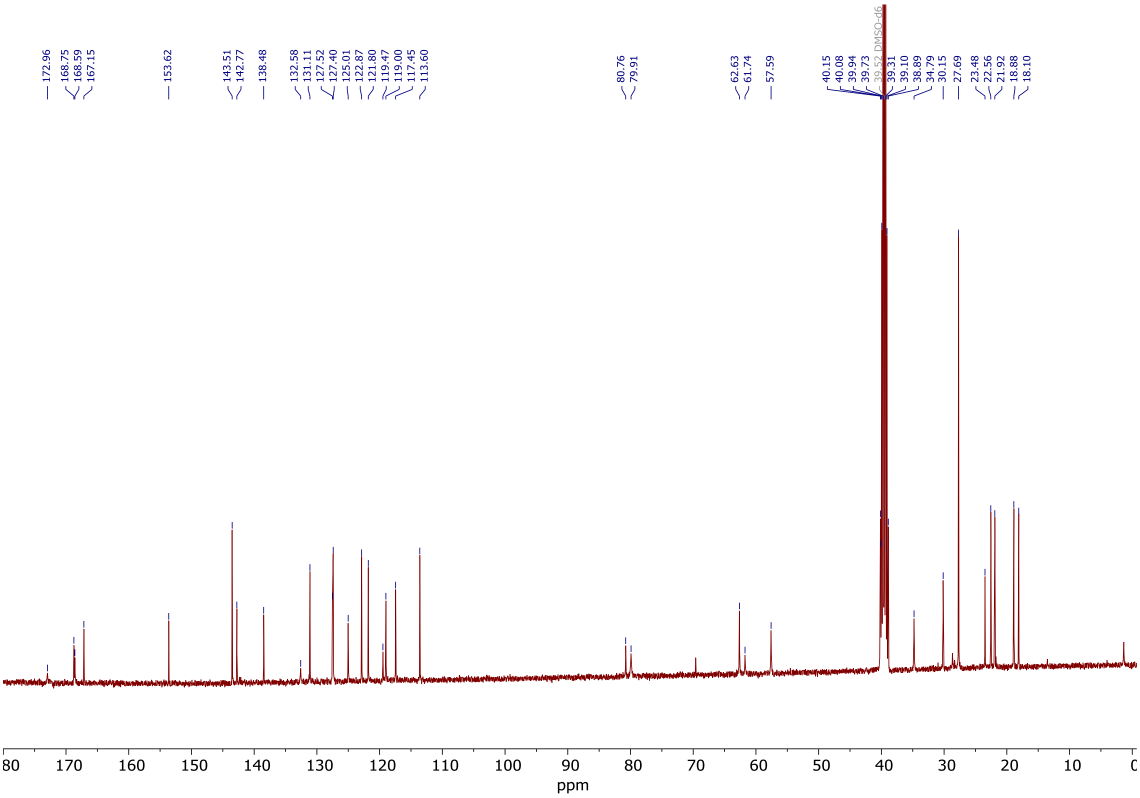

HPLC trace of the reaction progress to obtain *endo*-**63** (Scharlau, C18 Kromaphase 100 Kromaphase 100, 5 μm, 250 x 4.6 mm, gradient from CH_3CN/H_2O 50% to CH_3CN 100% in 20 min, 1.0 mL/min).

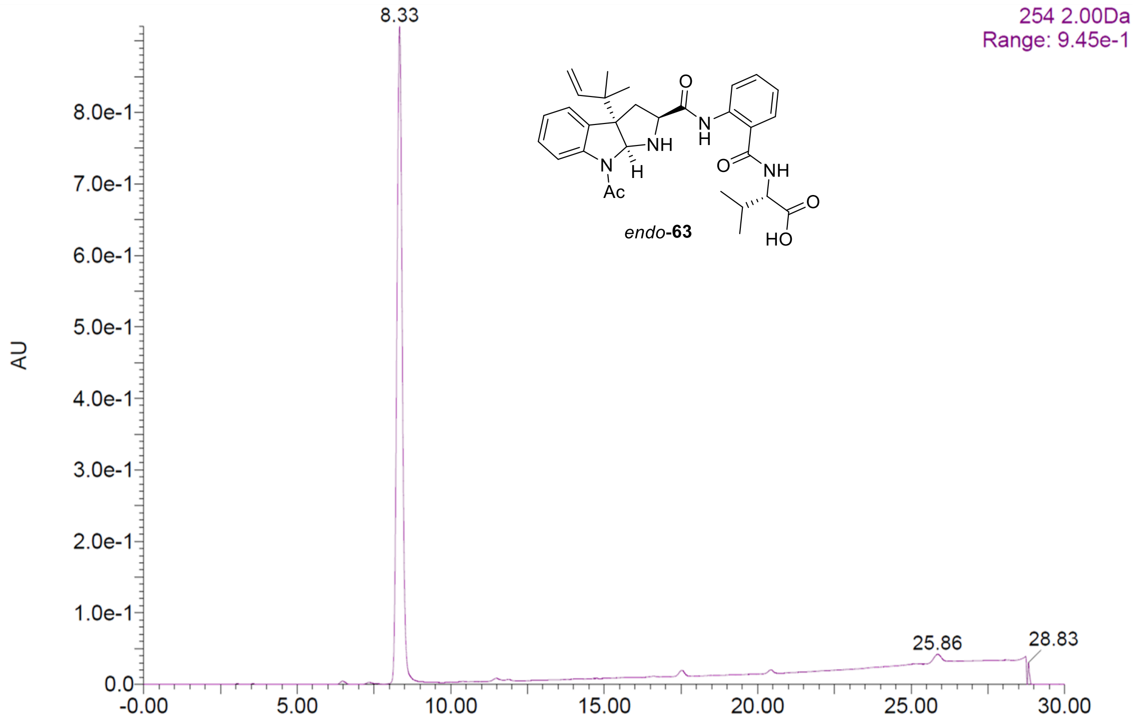

Scharlau_50_to_100_AcN_20min_ (8.370 min)

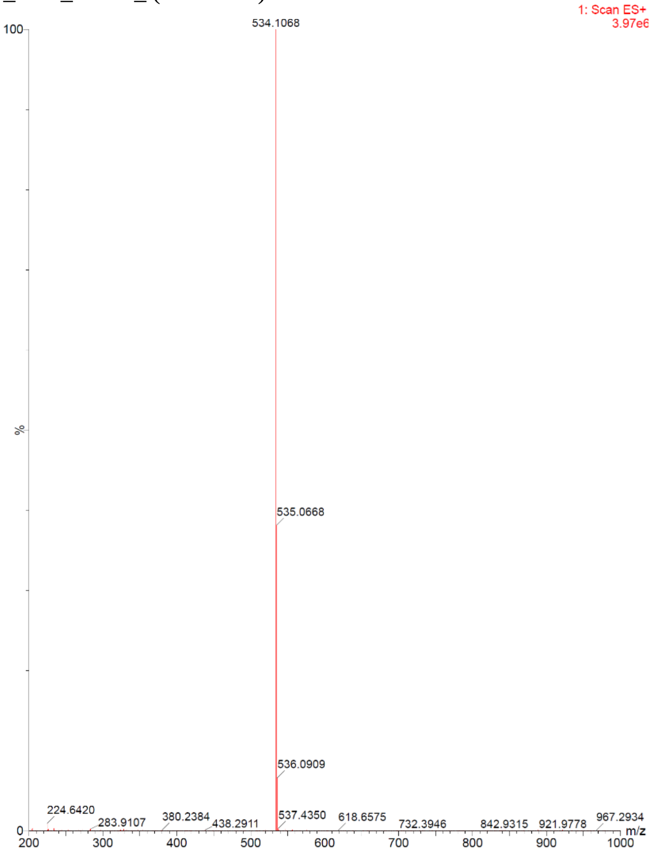

^{1}H NMR (400.16 MHz, $CDCl_3$)

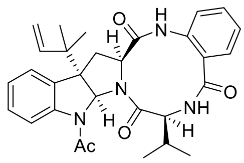

L-Trp-regio-*endo*-**56**

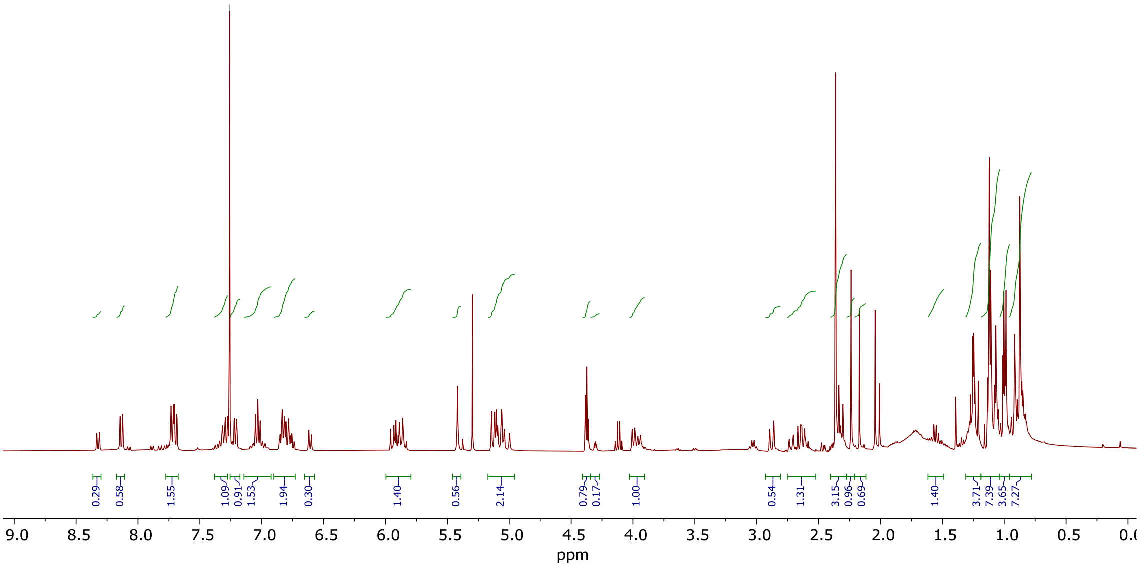

HPLC trace of the <u>reaction crude</u> of **Synthetic novofumigatamide from L-Trp** (L-Trp-regio-*endo*-**56**) (Scharlau, C18 Kromaphase 100 Kromaphase 100, 5 µm, 250 x 4.6 mm, gradient from CH_3CN/H_2O 50% to CH_3CN 100% in 20 min, 1.0 mL/min).

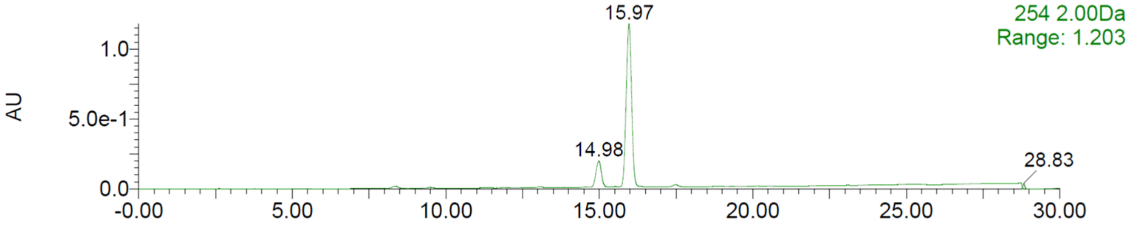

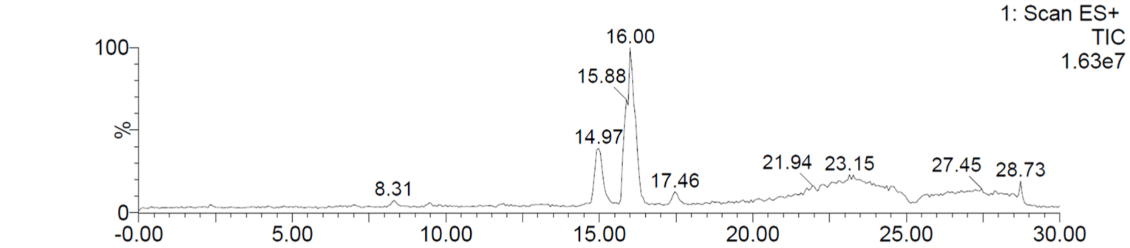

Scharlau_50_to_100_AcN_20min_ (14.974 min)

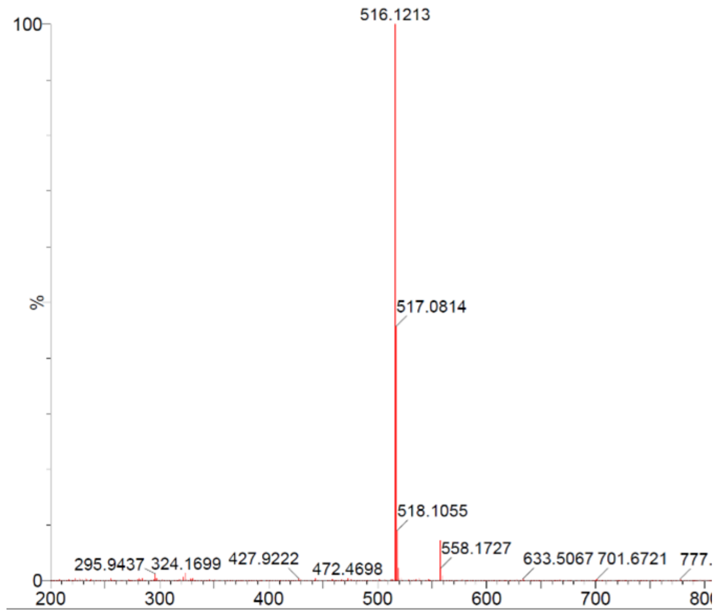

Scharlau_50_to_100_AcN_20min_ (15.943 min)

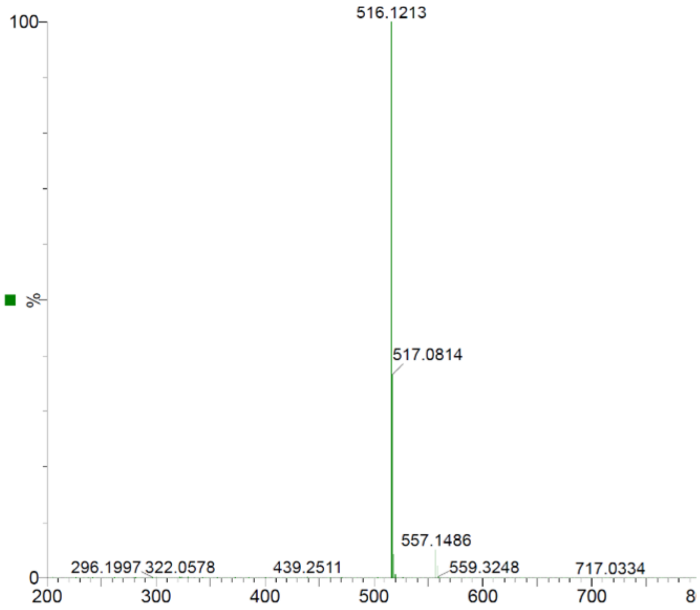

HPLC trace of the column fraction **Synthetic novofumigatamide from L-Trp** (L-Trp-regio-*endo*-**56**) (Scharlau, C18 Kromaphase 100 Kromaphase 100, 5 µm, 250 x 4.6 mm, gradient from CH_3CN/H_2O 50% to CH_3CN 100% in 20 min, 1.0 mL/min).

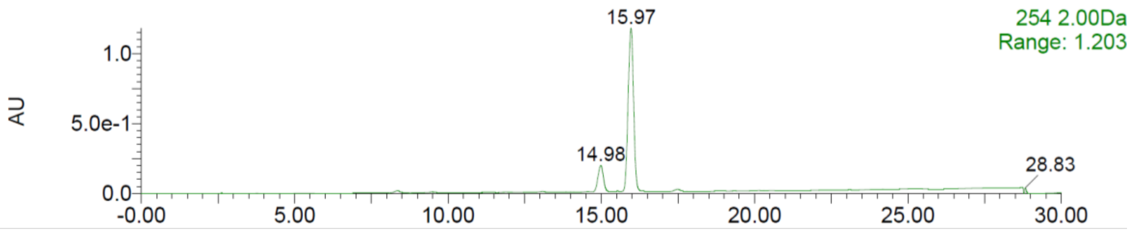

HPLC traces showing overtime decomposition of the product (L-Trp-regio-*endo*-**56**)

1. **After 1 day at - 30 ºC**

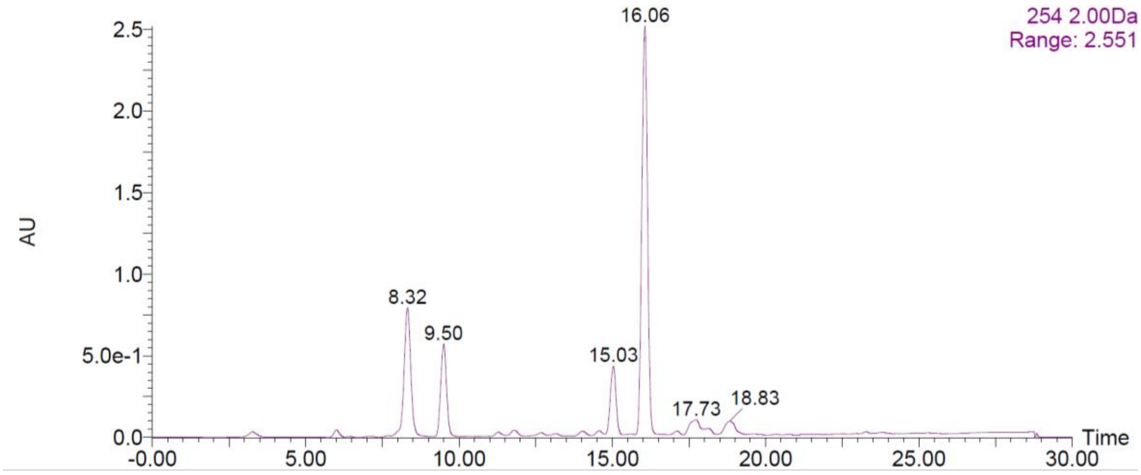

2. **After 6 days at - 30 ºC**

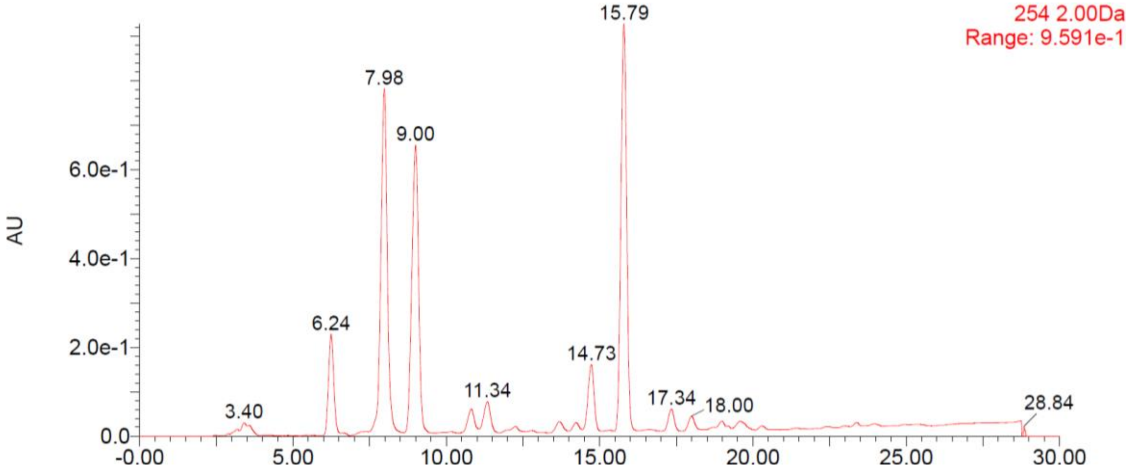

3. **After one week at - 30 ºC**

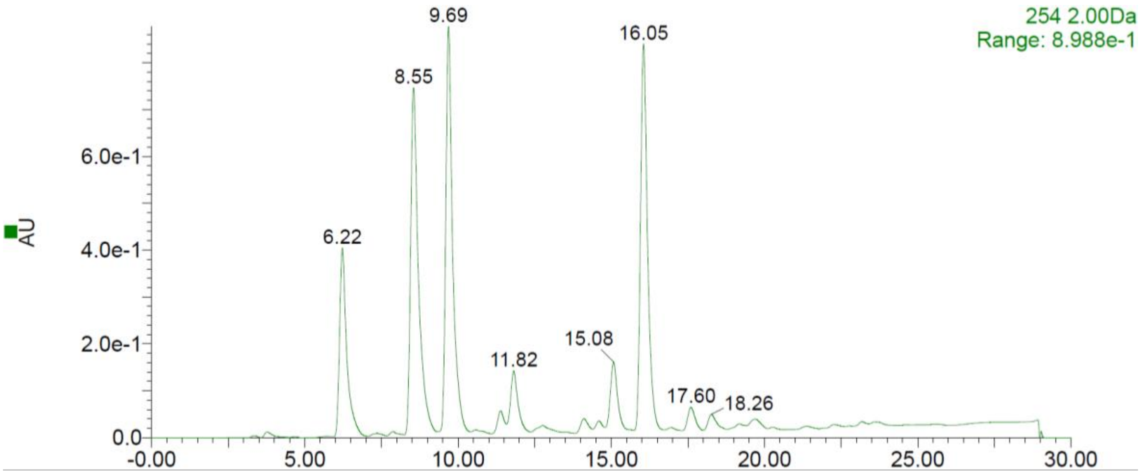

5. X-RAY Structures

(2*R*,3a*R*,8a*R*,2'*S*)-1-Acetyl-3a-bromo-8-{2'-[2"-(*tert*-butoxycarbonyl)amino-3''-methylbutanamido]benzoyl}-(1,2,3,3a,8,8a)-hexahydropyrrolo[2,3-*b*]indole-2-carboxylic Acid Methyl Ester (*endo*-37)

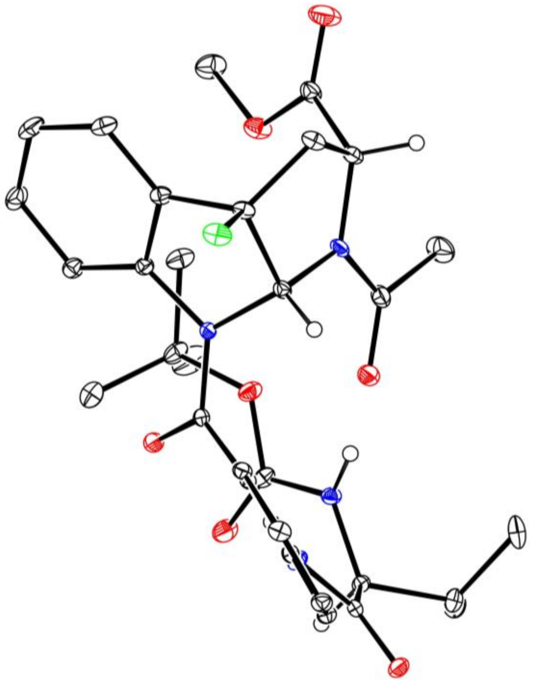

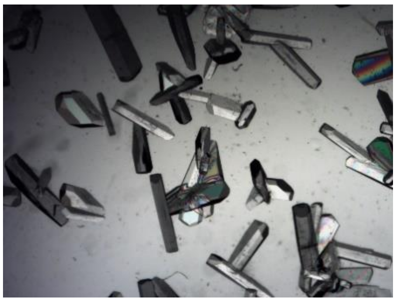

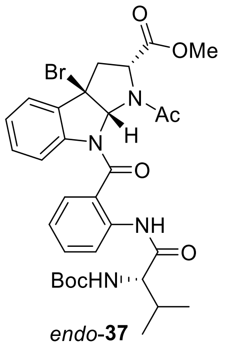

endo-**37**

Table 1. Crystal data and structure refinement for *endo*-**37**

Empirical formula	$C_{31}H_{37}BrN_4O_7$	
Formula weight	657.55	
Temperature	100.0 K	
Wavelength	0.71073 Å	
Crystal system	Orthorhombic	
Space group	$P2_12_12_1$	
Unit cell dimensions	a = 10.1429(5) Å	α= 90°.
	b = 16.6510(8) Å	β= 90°.
	c = 18.8633(10) Å	γ = 90°.
Volume	3185.8(3) $Å^3$	
Z	4	
Density (calculated)	1.371 Mg/m^3	
Absorption coefficient	1.342 mm^{-1}	
F(000)	1368	
Crystal size	0.257 x 0.175 x 0.153 mm^3	
Theta range for data collection	2.280 to 30.581°.	
Index ranges	-11<=h<=14, -23<=k<=23, -27<=l<=26	

Reflections collected	58020
Independent reflections	9770 [R(int) = 0.0394]
Completeness to theta = 25.242°	99.9 %
Absorption correction	Semi-empirical from equivalents
Max. and min. transmission	0.7461 and 0.5699
Refinement method	Full-matrix least-squares on F^2
Data / restraints / parameters	9770 / 2 / 401
Goodness-of-fit on F^2	1.035
Final R indices [I>2sigma(I)]	R1 = 0.0281, wR2 = 0.0618
R indices (all data)	R1 = 0.0368, wR2 = 0.0648
Absolute structure parameter	-0.010(2)
Extinction coefficient	n/a
Largest diff. peak and hole	0.401 and -0.572 e.Å^{-3}

Experimental report

Crystallization was achieved in toluene/hexane. A single crystal of *endo*-**37** was analysed by X-ray diffraction and a summary of the crystallographic data and the structure refinement parameters is reported in Table 1. Crystallographic data were collected at 100 K using a Bruker D8 Venture diffractometer with a Photon 100 CMOS detector and Mo-Kα radiation (λ = 0.71073 Å) generated by an Incoatec high brillance microfocus source equipped with Incoatec Helios multilayer optics. The software $APEX_3$[1] was used for collecting frames of data, indexing reflections, and the determination of lattice parameters, SAINT[2] for integration of intensity of reflections, and SADABS[3] for scaling and empirical absorption correction. The structure was solved by dualspace algorithm using the program SHELXT.[4] All non-hydrogen atoms were refined with anisotropic thermal parameters by full-matrix least-squares calculations on F2 using the program SHELXL[5] with $OLEX_2$.[6] Hydrogen atoms were inserted at calculated positions and constrained with isotropic thermal parameters except for the hydrogen atoms of the -NH groups, whose positions were located from a Fourier-difference map and refined isotropically restraining the N-H distances. The molecule crystallized in the chiral space group $P2_12_12_1$; the absolute configuration was established by anomalous dispersion effects in diffraction measurements on the crystal [Flack[7] parameter = -0.010(2)]. Drawings were produced with PLATON.[8]

---

[1] $APEX_3$ Version 2018.7-2 (Bruker AXS Inc., 2017).
[2] SAINT Version 8.38A (Bruker AXS Inc., 2017).
[3] SADABS Version 2016/2 (Krause, L.; Herbst-Irmer, R.; Sheldrick, G. M.; Stalke, D. *J. Appl. Cryst.* **2015**, *48*, 3).
[4] SHELXT Version 2014/5 (Sheldrick, G. M. *Acta Cryst.* **2015**, *A71*, 3).
[5] SHELXL Version 2018/3 (Sheldrick, G. M. *Acta Cryst.* **2015**, *C71*, 3).
[6] $OLEX_2$: A complete structure solution, refinement and analysis program (Dolomanov, O.V.; Bourhis, L. J.; Gildea, R. J.; Howard, J. A. K.; Puschmann, H. *J. Appl. Cryst.* **2009**, *42*, 339).
[7] Flack, H. D. *Acta Cryst.* **1983**, *A39*, 876.
[8] PLATON, *A Multipurpose Crystallographic Tool* (Spek, A. L. *J. Appl. Cryst.*, **2003**, *36*, 7).

(2*R*,3a*S*,8a*S*,2'*S*)-1-Acetyl-8-{2'-[2"-(*tert*-butoxycarbonyl)amino-3''-methylbutanamido]benzoyl}-3a-(3'''-methylbut-1-en-3'''-yl)-(1,2,3,3a,8,8a)-hexahydropyrrolo[2,3-*b*]indole-2-carboxylic Acid Methyl Ester (*exo*-36)

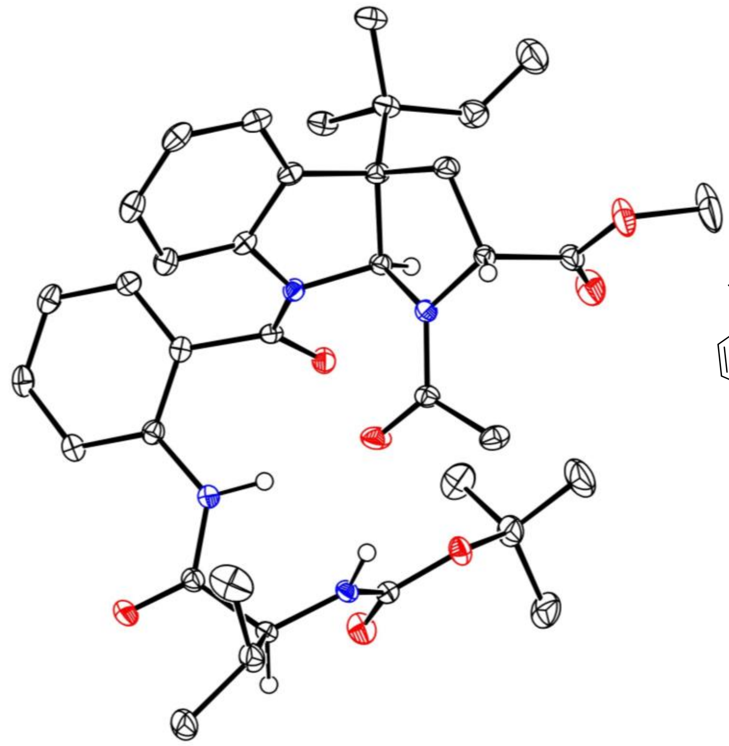

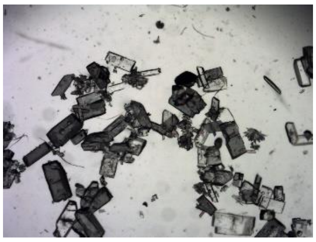

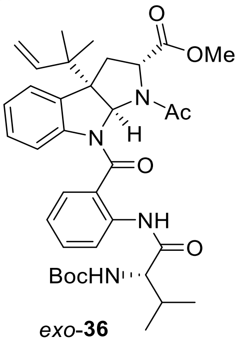

exo-**36**

Table 1. Crystal data and structure refinement for *exo*-**36**

Empirical formula	$C_{36}H_{46}N_4O_7$	
Formula weight	646.77	
Temperature	100.0 K	
Wavelength	1.54178 Å	
Crystal system	Monoclinic	
Space group	P 1 21 1	
Unit cell dimensions	a = 7.9557(6) Å	α= 90°.
	b = 20.5627(17) Å	β= 101.650(2)°.
	c = 10.7263(9) Å	γ = 90°.
Volume	1718.6(2) Å^3	
Z	2	
Density (calculated)	1.250 Mg/m^3	
Absorption coefficient	0.708 mm^{-1}	
F(000)	692	
Crystal size	0.231 x 0.156 x 0.148 mm^3	
Theta range for data collection	4.208 to 71.654°.	
Index ranges	-9<=h<=9, -25<=k<=24, -13<=l<=13	

Reflections collected	37508
Independent reflections	6623 [R(int) = 0.0304]
Completeness to theta = 67.679°	100.0 %
Absorption correction	Semi-empirical from equivalents
Max. and min. transmission	0.7535 and 0.6901
Refinement method	Full-matrix least-squares on F^2
Data / restraints / parameters	6623 / 3 / 439
Goodness-of-fit on F^2	1.031
Final R indices [I>2sigma(I)]	R1 = 0.0295, wR2 = 0.0788
R indices (all data)	R1 = 0.0295, wR2 = 0.0788
Absolute structure parameter	0.07(8)
Extinction coefficient	n/a
Largest diff. peak and hole	0.186 and -0.146 e.$Å^{-3}$

Experimental report

Crystallization was achieved in DMSO. A single crystal of *exo*-**36** was analysed by X-ray diffraction and a summary of the crystallographic data and the structure refinement parameters is reported in Table 1. Crystallographic data were collected at 100K using a Bruker Smart 6000 CCD detector and Cu-Kα radiation (λ = 1.54178 Å) generated by a Incoatec microfocus source equipped with Incoatec Quazar MX optics. The software $APEX_3$[1] was used for collecting frames of data, indexing reflections, and the determination of lattice parameters, SAINT[2] for integration of intensity of reflections, and SADABS[3] for scaling and empirical absorption correction. The structure was solved by dualspace algorithm using the program SHELXT.[4] All non-hydrogen atoms were refined with anisotropic thermal parameters by full-matrix least-squares calculations on F^2 using the program SHELXL[5] with $OLEX_2$.[6] Hydrogen atoms were inserted at calculated positions and constrained with isotropic thermal parameters except for the hydrogen atoms of the –NH groups, which were located from a Fourier-difference map and refined isotropically restraining the N-H distances. The sample has crystallized in the chiral space group $P2_1$. The absolute configuration has been established by anomalous dispersion effects in diffraction measurements on the crystal using Bayesian statistics on Bijvoet differences[7] [P_2(true) = 1.000, P_3(true) = 1.000, P_3(rac-twin) = 0.1 x 10^{-14} and P_3(false)=0.7 x 10^{-75}]. Drawings were produced with PLATON[8] and $OLEX_2$.[6]

[1] $APEX_3$ Version 2018.7-2 (Bruker AXS Inc., 2017).
[2] SAINT Version 8.38A (Bruker AXS Inc., 2017).
[3] SADABS Version 2016/2 (Krause, L.; Herbst-Irmer, R.; Sheldrick, G. M.; Stalke, D. *J. Appl. Cryst.* **2015**, *48*, 3).
[4] SHELXT Version 2014/5 (Sheldrick, G. M. *Acta Cryst.* **2015**, *A71*, 3).
[5] SHELXL Version 2018/3 (Sheldrick, G. M. *Acta Cryst.* **2015**, *C71*, 3).
[6] $OLEX_2$: A complete structure solution, refinement and analysis program (Dolomanov, O.V.; Bourhis, L. J.; Gildea, R. J.; Howard, J. A. K.; Puschmann, H. *J. Appl. Cryst.* **2009**, *42*, 339).
[7] Hooft, R. W. W.; Straver, L. H.; Spek, A. L. *J. Appl. Cryst.* **2008**, *41*, 96.
[8] PLATON, *A Multipurpose Crystallographic Tool* (Spek, A. L. *J. Appl. Cryst.*, **2003**, *36*, 7).

New synthetic novofumigatamide from D-Trp and L-Val xx (D-Trp-regio-*endo*-4)

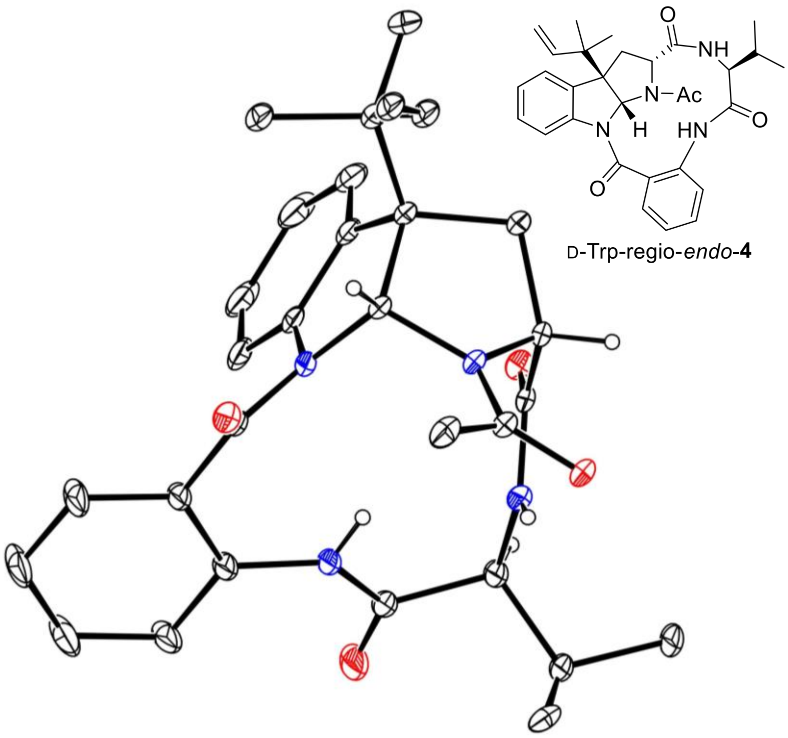

D-Trp-regio-*endo*-**4**

Table 1. Crystal data and structure refinement for **New synthetic novofumigatamide from D-Trp and L-Val** (D-Trp-regio-*endo*-**4**)

Empirical formula	$C_{30}H_{34}N_4O_4$	
Formula weight	514.61	
Temperature	100.0 K	
Wavelength	0.71073 Å	
Crystal system	Monoclinic	
Space group	$P2_1$	
Unit cell dimensions	a = 11.0322(6) Å	α= 90°.
	b = 9.2466(5) Å	β= 95.105(2)°.
	c = 13.0198(7) Å	γ = 90°.
Volume	1322.89(12) $Å^3$	
Z	2	
Density (calculated)	1.292 Mg/m^3	
Absorption coefficient	0.087 mm^{-1}	
F(000)	548	
Crystal size	0.281 x 0.135 x 0.077 mm^3	
Theta range for data collection	2.320 to 28.300°.	
Index ranges	-14<=h<=14, -12<=k<=12, -17<=l<=17	
Reflections collected	40413	
Independent reflections	6539 [R(int) = 0.0438]	
Completeness to theta = 25.242°	99.9 %	

Absorption correction	Semi-empirical from equivalents
Max. and min. transmission	0.7457 and 0.6911
Refinement method	Full-matrix least-squares on F^2
Data / restraints / parameters	6539 / 3 / 360
Goodness-of-fit on F^2	1.160
Final R indices [I>2sigma(I)]	R1 = 0.0682, wR2 = 0.1384
R indices (all data)	R1 = 0.0899, wR2 = 0.1462
Largest diff. peak and hole	0.302 and -0.222 e.Å^{-3}

Experimental report

Crystallization was achieved in CH_2Cl_2/pentane. A single crystal of **New synthetic novofumigatamide from D-Trp and L-Val** (D-Trp-regio-*endo*-**4**) was analysed by X-ray diffraction and a summary of the crystallographic data and the structure refinement parameters is reported in Table 1. Crystallographic data were collected at 100 K using a Bruker D8 Venture diffractometer with a_Photon 100 CMOS detector and Mo-Kα radiation (λ = 0.71073 Å) generated by an Incoatec high_brillance microfocus source equipped with Incoatec Helios multilayer optics. The software $APEX_3$[1] was used for collecting frames of data, indexing reflections, and the determination of lattice parameters, SAINT[2] for integration of intensity of reflections, and SADABS[3] for scaling and empirical absorption correction. The structure was solved by dualspace algorithm using the program SHELXT.[4] All non-hydrogen atoms were refined with anisotropic thermal parameters by full-matrix least-squares calculations on F^2 using the program SHELXL[5] with $OLEX_2$.[6] The vinyl group and a methyl group are disordered over two positions, the site occupations factors were refined converging to 67:33. Hydrogen atoms were inserted at calculated positions and constrained with isotropic thermal parameters except for the hydrogen atoms of the -NH groups, whose positions were located from a Fourierdifference map and refined isotropically restraining the N-H distances. The molecule crystallized in the chiral space group $P2_1$; but absolute configuration was not established by anomalous dispersion effects in diffraction measurements on the crystal. The enantiomer was assigned by reference to two unchanging chiral centers in the synthetic procedure. Drawings were produced with PLATON.[7]

[1] $APEX_3$ Version 2018.7-2 (Bruker AXS Inc., 2017).
[2] SAINT Version 8.38A (Bruker AXS Inc., 2017).
[3] SADABS Version 2016/2 (Krause, L.; Herbst-Irmer, R.; Sheldrick, G. M.; Stalke, D. *J. Appl. Cryst.* **2015**, *48*, 3).
[4] SHELXT Version 2014/5 (Sheldrick, G. M. *Acta Cryst.* **2015**, *A71*, 3).
[5] SHELXL Version 2018/3 (Sheldrick, G. M. *Acta Cryst.* **2015**, *C71*, 3).
[6] $OLEX_2$: A complete structure solution, refinement and analysis program (Dolomanov, O.V.; Bourhis, L. J.; Gildea, R. J.; Howard, J. A. K.; Puschmann, H. *J. Appl. Cryst.* **2009**, *42*, 339).
[7] PLATON, *A Multipurpose Crystallographic Tool* (Spek, A. L. *J. Appl. Cryst.*, **2003**, *36*, 7).

Bromine-containing precursor of regioisomer L-Trp-regio-56 (*endo*-61)

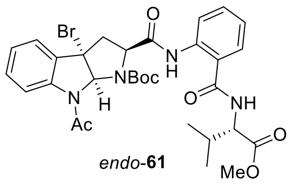

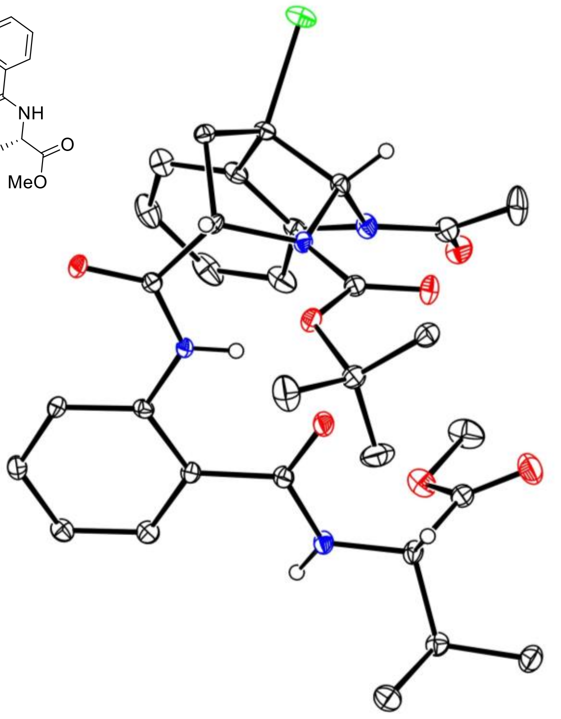

Table 1. Crystal data and structure refinement for *endo*-**61**

Empirical formula	$C_{31}H_{37}BrN_4O_7$	
Formula weight	657.55	
Temperature	100.0 K	
Wavelength	0.71073 Å	
Crystal system	Monoclinic	
Space group	P 1 21 1	
Unit cell dimensions	a = 9.4517(18) Å	α= 90°.
	b = 15.162(3) Å	β= 105.183(7)°.
	c = 11.6010(19) Å	γ = 90°.
Volume	1604.4(5) $Å^3$	
Z	2	
Density (calculated)	1.361 Mg/m^3	
Absorption coefficient	1.332 mm^{-1}	
F(000)	684	
Crystal size	0.226 x 0.214 x 0.036 mm^3	
Theta range for data collection	2.261 to 27.734°.	
Index ranges	-12<=h<=12, -19<=k<=19, -13<=l<=15	
Reflections collected	44372	
Independent reflections	7180 [R(int) = 0.0486]	
Completeness to theta = 25.242°	99.9 %	
Absorption correction	Semi-empirical from equivalents	

Max. and min. transmission	0.7456 and 0.5669
Refinement method	Full-matrix least-squares on F^2
Data / restraints / parameters	7180 / 1 / 403
Goodness-of-fit on F^2	1.052
Final R indices [I>2sigma(I)]	R1 = 0.0314, wR2 = 0.0686
R indices (all data)	R1 = 0.0349, wR2 = 0.0700
Absolute structure parameter	-0.007(3)
Extinction coefficient	n/a
Largest diff. peak and hole	0.506 and -0.419 e.Å^{-3}

Experimental report

Crystallization was achieved in DMSO. A single crystal of *endo*-**61** was analysed by X-ray diffraction and a summary of the crystallographic data and the structure refinement parameters is reported in Table 1. Crystallographic data were collected at 100 K using a Bruker D8 Venture diffractometer with a Photon II CMOS detector and Mo-Kα radiation (λ = 0.71073 Å) generated by an Incoatec high brillance microfocus source equipped with Incoatec Helios multilayer optics.
The software $APEX_3$[1] was used for collecting frames of data, indexing reflections, and determination of lattice parameters, SAINT[2] for integration of intensity of reflections, and SADABS[3] for scaling and empirical absorption correction. The structure was solved by dualspace algorithm using the program SHELXT.[4] All non-hydrogen atoms were refined with anisotropic displacement parameters by full-matrix least-squares calculations on F^2 using the program SHELXL[5] with $OLEX_2$.[6] Hydrogen atoms were inserted at calculated positions and constrained with isotropic displacement; except for the hydrogen atoms of the –NH groups, which were located from a Fourier-difference map and refined isotropically. The sample crystallized in the chiral space group $P2_1$; the absolute configuration was determined by anomalous dispersion effects in diffraction measurements on the crystal [Flack[7] parameter = -0.007(3)]. Drawings were produced with PLATON.[8]

[1] $APEX_3$ Version 2019.11-0 (Bruker AXS Inc., 2019).
[2] SAINT Version 8.40A (Bruker AXS Inc., 2019).
[3] SADABS Version 2016/2 (Krause, L.; Herbst-Irmer, R.; Sheldrick, G. M.; Stalke, D. *J. Appl. Cryst.* **2015**, *48*, 3).
[4] SHELXT Version 2018/2 (Sheldrick, G. M. *Acta Cryst.* **2015**, *A71*, 3).
[5] SHELXL Version 2018/3 (Sheldrick, G. M. *Acta Cryst.* **2015**, *C71*, 3).
[6] $OLEX_2$: A complete structure solution, refinement and analysis program (Dolomanov, O.V.; Bourhis, L. J.; Gildea, R. J.; Howard, J. A. K.; Puschmann, H. *J. Appl. Cryst.* **2009**, *42*, 339).
[7] Flack, H. D. *Acta Cryst.* **1983**, *A39*, 876; Flack, H. D.; Bernardinelli, G. *Acta Cryst.* **1999**, *A55*, 908.
[8] PLATON, *A Multipurpose Crystallographic Tool* (Spek, A. L. *J. Appl. Cryst.*, **2003**, *36*, 7).

6. References

1. ElHady, A. K.; Shih, S.-P.; Chen, Y.-C.; Liu, Y.-C.; Ahmed, N. S.; Keeton, A. B.; Piazza, G. A.; Engel, M.; Abadi, A. H.; Abdel-Halim, M. Extending the use of tadalafil scaffold: Development of novel selective phosphodiesterase 5 inhibitors and histone deacetylase inhibitors. *Bioorg. Chem.* **2020**, *98*, 103742.
2. Reay, A. J.; Williams, T. J.; Fairlamb, I. J. S. Unified mild reaction conditions for C2-selective Pd-catalysed tryptophan arylation, including tryptophan-containing peptides. *Org. Biomol. Chem.* **2015**, *13*, 8298-8309.
3. García-Domínguez, P.; Lorenzo, P.; Álvarez, R.; de Lera, A. R. Total Synthesis of the Proposed Structure of (-)-Novofumigatamide, Isomers Thereof and Analogues. Part I. *J. Org. Chem.* **2022**, DOI: 10.1021/acs.joc.2c01127.
4. Coste, A.; Toumi, M.; Wright, K.; Razafimahaleo, V.; Couty, F.; Marrot, J.; Evano, G. Copper-catalyzed cyclization of iodo-tryptophans: a straightforward synthesis of pyrroloindoles. *Org. Lett.* **2008**, *10*, 3841-4.
5. Engler, T. A.; Reddy, J. P.; Combrink, K. D.; Vander Velde, D. Formal 2 + 2 and 3 + 2 cycloaddition reactions of 2H-chromenes with 2-alkoxy-1,4-benzoquinones: regioselective synthesis of substituted pterocarpans. *J. Org. Chem.* **1990**, *55*, 1248-1254.
6. Naruta, Y.; Nishigaichi, Y.; Maruyama, K. Tributyl(3-methyl-2-butenyl)tin [Stannane, tributyl(3-methyl-2-butenyl)-]. *Org. Synth.* **1993**, *71*, 118.
7. Hédou, D.; Deau, E.; Harari, M.; Sanselme, M.; Fruit, C.; Besson, T. Rational multistep synthesis of a novel polyfunctionalized benzo[*d*]thiazole and its thiazolo[5,4-*b*]pyridine analogue. *Tetrahedron* **2014**, *70*, 5541-5549.
8. Gao, X.; Haynes, S. W.; Ames, B. D.; Wang, P.; Vien, L. P.; Walsh, C. T.; Tang, Y. Cyclization of fungal nonribosomal peptides by a terminal condensation-like domain. *Nat. Chem. Biol.* **2012**, *8*, 823-830.
9. McKay, C. S.; Finn, M. G. Polyvalent Catalysts Operating on Polyvalent Substrates: A Model for Surface-Controlled Reactivity. **2016**, *55*, 12643-12649.
10. Flores-López, L. Z.; Parra-Hake, M.; Somanathan, R.; Ortega, F.; Aguirre, G. Synthesis of Some New Chiral Sulfonamide Ligands. *Synth. Commun.* **2000**, *30*, 147-155.
11. Ishikawa, K.; Hosoe, T.; Itabashi, T.; Kayoko, T.; Takashi, Y.; Ken-ichi, K. A novofumigatamide, new cyclic tripeptide from aspergillus novofumigatus. *Heterocycles* **2010**, *81*, 2143-2148.
